# Genome-wide Mapping Reveals Conservation of Promoter DNA Methylation Following Chicken Domestication

**DOI:** 10.1038/srep08748

**Published:** 2015-03-04

**Authors:** Qinghe Li, Yuanyuan Wang, Xiaoxiang Hu, Yaofeng Zhao, Ning Li

**Affiliations:** 1 Institute of Animal Sciences, Chinese Academy of Agricultural Sciences, Beijing, 100193, China; 2 Department of Biological Sciences, Bengbu Medical College, Bengbu 233030, China; 3 State Key Laboratory for Agro-biotechnology, China Agricultural University, Beijing 100193, China; 4 National Engineering Laboratory for Animal Breeding, China Agricultural University, Beijing, China

## Abstract

It is well-known that environment influences DNA methylation, however, the extent of heritable DNA methylation variation following animal domestication remains largely unknown. Using meDIP-chip we mapped the promoter methylomes for 23,316 genes in muscle tissues of ancestral and domestic chickens. We systematically examined the variation of promoter DNA methylation in terms of different breeds, differentially expressed genes, SNPs and genes undergo genetic selection sweeps. While considerable changes in DNA sequence and gene expression programs were prevalent, we found that the inter-strain DNA methylation patterns were highly conserved in promoter region between the wild and domestic chicken breeds. Our data suggests a global preservation of DNA methylation between the wild and domestic chicken breeds in either a genome-wide or locus-specific scale in chick muscle tissues.

Epigenetic information, which exists as different kinds of modifications in DNA and histone proteins, is considered as a heritable genetic code in addition to the sequence of DNA^1^,^2^. The mechanisms of how epigenetic modifications and their interactions generate regulatory signals to modulate diverse biological processes have been intensively studied in recent years^3^,^4^,^5^,^6^,^7^. We have gained knowledge on the distribution of epigenetic modifications across the genome, however, little information is available regarding the dynamics of epigenetic modifications during development and disease, the contribution of epigenetic modifications to gene expression, and the stability of the epigenetic code under long-term environmental changes and selections.

Environmental factors affect phenotypes via both genetic and epigenetic mechanisms^8^,^9^. In mice, the epigenetic state of an intracisternal A-particle (IAP) retrotransposon inserted upstream of the *Agouti* coding region, and subsequently, the expression of the *Agouti* gene are influenced by the S-adenosylmethionine (SAM) content of maternal food intake^10^,^11^. Low degree of IAP methylation leads to overexpression of the *Agouti* gene and consequently ectopic phenotypes such as obesity, diabetes and increased susceptibility to tumors^10^,^11^. Furthermore, adaptive traits induced by the environment can be transmitted to the following generations through epigenetic inheritance mechanisms^12^. For example, the exposure of female rats to the antiandrogenic fungicide vinclozolin induces a female-specific mate preference after three generations^13^.

Above findings suggest that environmental factors could induce heritable epigenetic changes to influence phenotypes. An interesting question is whether epigenetic mechanism is involved in shaping traits of farm animals following domestication. Chicken was first domesticated from the red jungle fowl (*Gallus gallus,* RJF) in southeast Asia about 8,000 years ago^14^. Chahua chicken (CH) is a native breed in southwest China that displays many phenotypes and behaviors similar to RJF^15^. Avian broiler (AA) is a well-known breed used for meat production worldwide, and white leghorn (WL) has good performance in egg production. Both AA and WL have been subject to intensive human selection and have become accustomed to a man-made environment. Recently, extensive analyses have been done towards determining the genetic mechanisms underlying phenotypic diversity of farm animals^16^. Millions of DNA mutations have been identified in different chicken breeds^17^,^18^. However, the epigenetic variations in the process of animal domestication have been rarely studied.

In this study, we focused on the promoter DNA methylation and systematically analyzed the changes of DNA methylome following chicken domestication. With the development of DNA microarray and next-generation sequencing technologies, high-throughput methods such as the methylated DNA immunoprecipitation (meDIP) approach and bisulfite sequencing (bis-seq) assay can be combined to perform unbiased genome-wide analysis of DNA methylation profile^1^,^6^,^19^. MeDIP can specifically pull down the DNA stretches with methylated cytosines and quantify the methylation level by hybridizing DNA to a whole-genome promoter array. We applied the meDIP to quantify relative methylation level of the promoters for four chicken breeds using the MEDME routine (modeling experimental data with meDIP enrichment)^4^,^20^. Our results reveal a surprising conservation of DNA methylation between the wild (RJF) and domestic chicken breeds (CH, AA and WL) in either a genome-wide or locus-specific scale in chick muscle tissues. These findings shed novel insights into the influence of domestication on DNA methylation variation.

## Results

### Genome-wide mapping and characterization of promoter methylation in chickens

The MeDIP hybridization data was processed using MEDME to determine the relative methylation scores (RMS)^4^,^20^. RMS is an estimate of the relative probe-level methylation (mCpG/CpG) and is reported to be linearly correlated with that obtained using bis-seq^20^. We found the MeDIP results showed a high consistency to that of bis-seq method suggesting a high quality of DNA methylation data (Supplementary Fig. S1 and S2).

**Figure 1:**
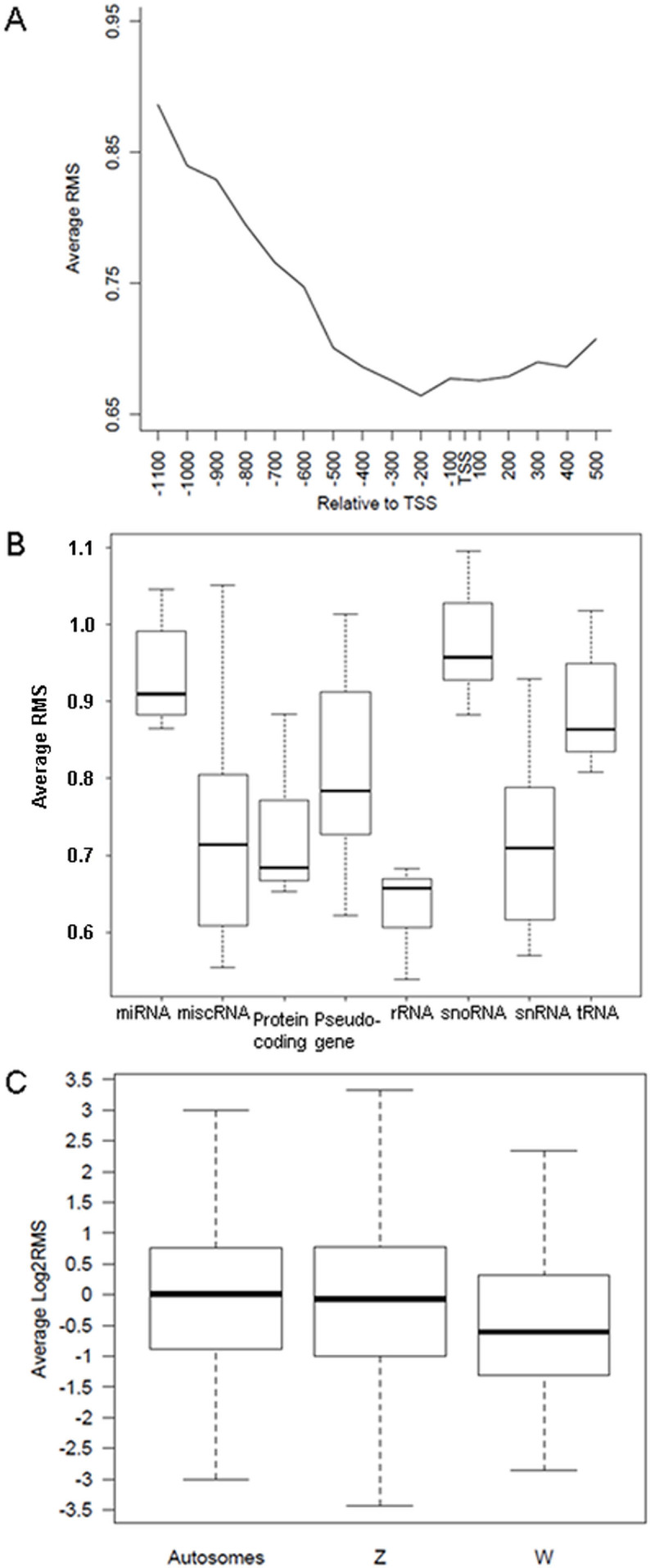
Characteristics of promoter DNA methylation in the chicken genome. (A) Distribution of DNA methylation around the TSS in the chicken genome. (B) Average relative methylation level of different gene categories in the chicken genome. Box plots showing the methylation level distribution of each gene category. Gene methylation levels of 25–75% were selectively used and the middle line represents the average methylation level. (C) Relative DNA methylation levels of autosomes, and Z and W chromosomes.

We first analyzed the distribution of DNA methylation around the TSS. We found the DNA methylation level decreased gradually towards the TSS and increased towards the gene body region, forming a valley at about 200 bp upstream of transcription start site (TSS) (Figure 1A). The depletion of DNA methylation immediately upstream of the TSS might be beneficial for the binding of transcription factors.

We then investigated the relative methylation level of promoters for different gene categories. We found that genes encoding miRNAs (*P* = 6.30 × e^−10^), pseudogenes (*P* = 0.019), snoRNAs (*P* = 2.39 × e^−11^) and tRNAs (*P* = 2.25 × e^−7^) showed relatively high levels of promoter methylation compared to the protein-encoding genes. The promoters of the miscRNA (*P* = 0.94) genes and snRNA (*P* = 0.79) genes showed similar methylation levels with the protein-encoding genes, while the rRNA gene promoters had a relatively lower methylation level (*P* = 0.0053) (Figure 1B). These results are consistent with previous studies on *Arabidopsis* and silkworms^21^,^22^. These studies reported that the promoters of miRNA genes were usually highly methylated, suggesting a conserved phenomenon among species.

One pair of chromosomes distinguishes the sex in birds, ZZ for males and ZW for females^23^. Although the avian ZW chromosomes have been suggested to be evolved from autosomes, recent studies have shown that Z chromosome is different from the autosomes in terms of gene content and expression^24^. Z chromosome is less gene-dense and contains more interspersed repeats compared to autosomes^23^,^24^. We compared the promoter methylation of sex chromosomal and autosomal genes and detected no significant difference in the average promoter methylation level between the Z chromosome and autosomes. However, the promoters on the W chromosome were significantly less methylated compared to Z chromosome and autosomes (*P* = 8.63 × e^−15^ between W and Z, *P* = 3.07 × e^−16^ between W and autosomes, Figure 1C).

Furthermore, we analyzed the enrichment of methylated and unmethylated genes in the chicken genome. We defined promoters with an RMS of >1.5 as highly methylated, an RMS of 0.8~1.5 as intermediately methylated, and an RMS of <0.8 as lowly methylated (the thresholds were defined empirically according to our bis-seq results, data not shown). Genes that were consistently methylated or lowly methylated in all four breeds were termed as conserved highly methylated genes (CHMGs) or lowly methylated genes (CLMGs), respectively. In total, we identified 299 CHMGs and 2,429 CLMGs (Supplementary Table 1). Four CHMGs and four CLMGs were randomly selected for bis-seq analysis. The results showed that four CHMGs were all highly methylated and four CLMGs were all sparsely methylated (Supplementary Fig. S3 and S4). Gene ontology (GO) analysis showed that CHMGs were enriched for genes involved in regulation of apoptosis, catecholamine metabolic, and lipid metabolic processes. In contrast, CLMGs were enriched for genes required for many basic biological processes, such as DNA-dependent regulation of transcription and the G-protein coupled receptor protein signaling pathway (Table 1).

### Global conservation of promoter DNA methylomes in chicken breeds

**Figure 2:**
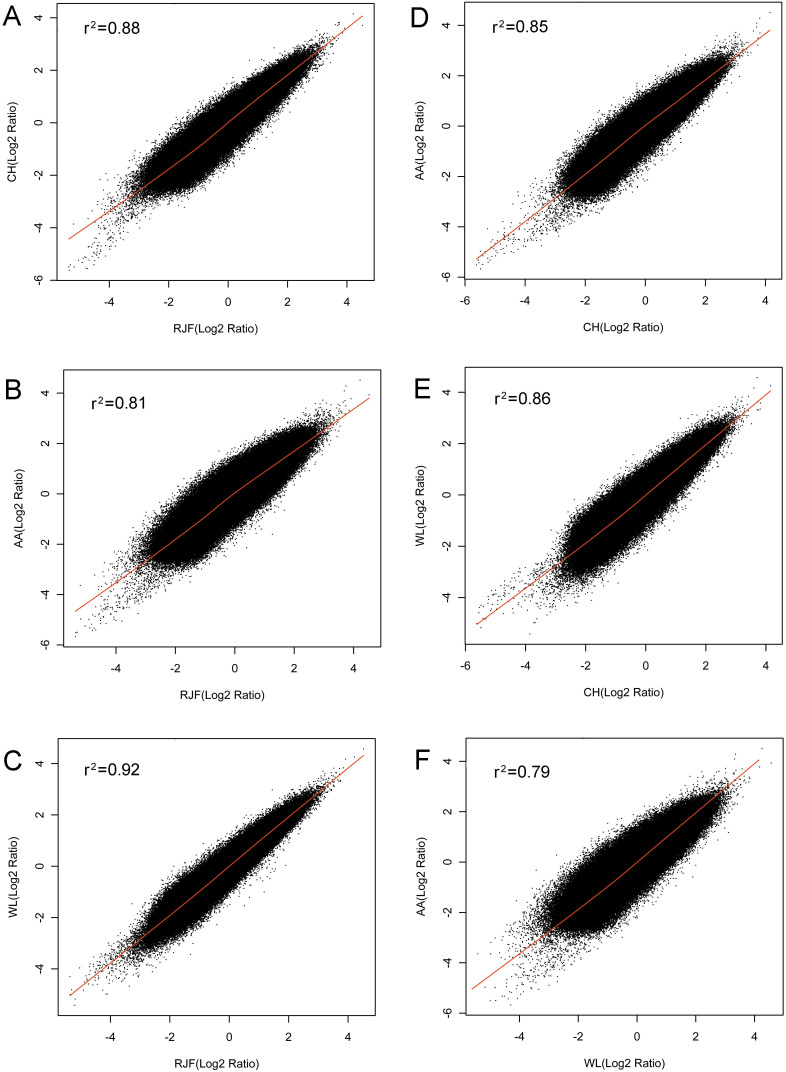
Correlation of global DNA methylation levels between chicken breeds. Scatter plots showing the correlation of DNA methylation level of all the 390,000 probes in the microarray between different breeds. The numbers in both axes represent the log2 ratio of probes. (A) RJF and CH. (B) RJF and AA. (C) RJF and WL. (D) CH and AA. (E) CH and WL. (F) AA and WL.

We compared the whole genome promoter methylomes between different chicken breed pairs. First, we analyzed the correlation between RJF and three domestic chicken breeds based on the signal density of all probes. The methylation of each chicken breed was represented by the average relative methylation level of all analyzed individuals. Scatter plots comparing the average DNA methylation level between RJF and CH, RJF and AA, or RJF and WL revealed strong correlations of promoter DNA methylation between the wild and domestic chicken breeds (Pearson's r: RJF and CH, r^2^ = 0.88; RJF and AA, r^2^ = 0.81; RJF and WL, r^2^ = 0.92. Figure 2A, 2B and 2C). Next, we compared the methylation profiles of three domestic chicken breeds and found that all the three breeds showed indistinguishable promoter methylation patterns and there was no obvious methylation difference between CH and AA, CH and WL, or AA and WL (Pearson's r: CH and AA, r^2^ = 0.85; CH and WL, r^2^ = 0.86; AA and WL, r^2^ = 0.79. Figure 2D, 2E and 2F). Finally, hierarchical clustering of the methylation level of all samples from the four breeds failed to distinguish samples from different breeds indicating a lack of breed-specific promoter methylation (Supplementary Fig. S5).

The above correlation analysis was based on the log2 ratio of all probes. To eliminate the variation potentially caused by the analysis method, we analyzed differentially methylated genes (DMGs) using the data generated by MEDME. Based on our criteria for DMGs in method section, we did not identify any DMG between RJF and CH, CH and AA, or CH and WL. There were 4 DMGs between RJF and AA, 1 gene between RJF and WL, and 35 DMGs between AA and WL (Supplementary Table 2). Seven of these genes (two between RJF and AA, five between AA and WL) were selected for bis-seq validation, and bis-seq results showed that they were all false positive (Supplementary Fig. S6). In addition, we randomly selected 795 CpG sites for bis-seq and quantified the methylation level in all CpG sites of the four samples. Again, no significant inter-breed differences were detected in these sites (Supplementary Fig. S7). Our results thus suggest that at least in the 23,316 promoter regions of the muscle tissues included in our array, there are no dramatic changes in promoter DNA methylation between the wild and domestic chicken breeds, or between the domestic chicken breeds.

### Changes in gene expression level between chicken breeds do not globally correlate with promoter methylation variations

Thousands of genes show differential expression levels between the wild and domestic chicken breeds^25^. Promoter DNA methylation is a well-studied suppressor of gene transcription^1^,^4^,^21^. Hence, we investigated whether DNA methylation contributes to the gene expression difference between chicken breeds.

First, we obtained gene expression profiles of RJF, CH, AA and WL using the RNA-seq assay, and defined differentially expressed genes (DEGs) using the criteria of >2-fold change, *P* < 0.05, and FDR < 0.05. In total, we identified 740, 1148, 2457, 168, 3791, and 4094 DEGs (Supplementary Table 3) between RJF and CH, RJF and AA, RJF and WL, CH and AA, CH and WL, and AA and WL, respectively.

**Figure 3:**
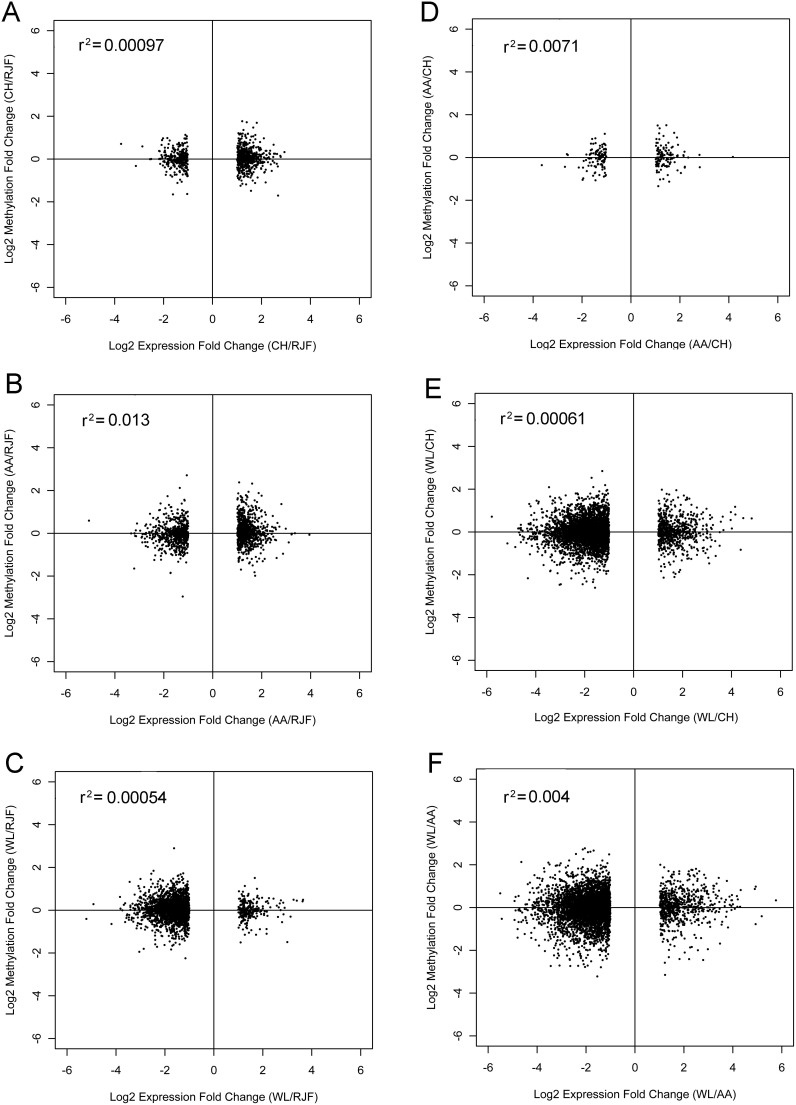
Correlation between differential gene expression and promoter DNA methylation. The X axis represents the fold change of gene expression between samples, and the y axis represents the fold change of methylation level. For each promoter, the average change in cytosine methylation is compared to the change in mRNA. (A) RJF and CH. (B) RJF and AA. (C) RJF and WL. (D) CH and AA. (E) CH and WL. (F) AA and WL.

**Figure 4:**
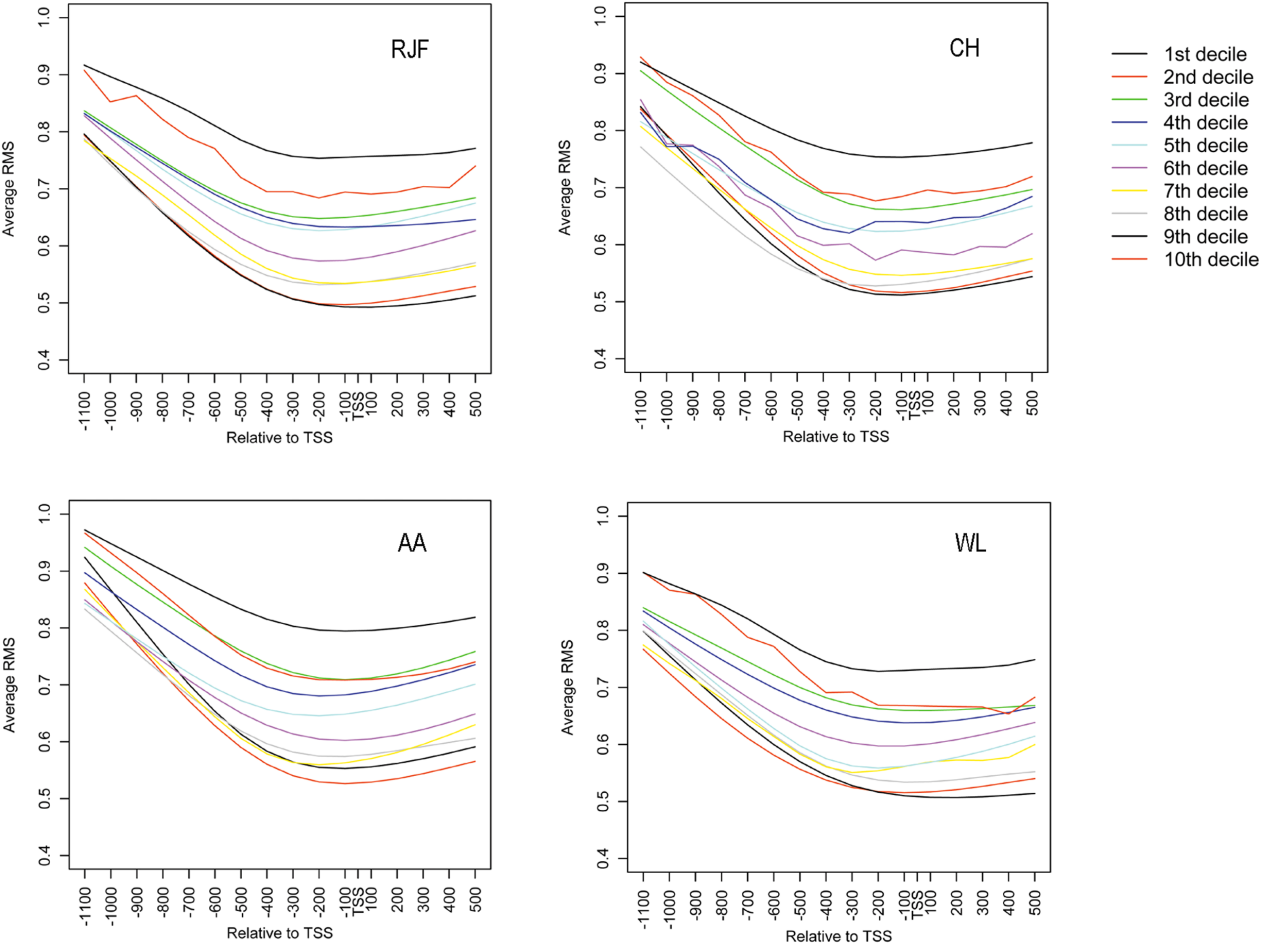
Relationship between promoter DNA methylation and expression levels of genes in chicken. Genes were classified into deciles based on the expression level, the 1st decile is the lowest and the 10th is the highest. Promoter regions of each gene probed by our array were divided into 100-bp fragments. Plots show the methylation level of each fragment.

Next, we analyzed the correlation between the changes in promoter methylation level and the changes in gene expression level in all chicken breeds. We found that the changes in promoter methylation correlated poorly with the changes in gene expression in a global level (Figure 3). Although genes in the second and fourth quadrants showed a mild negative correlation, none of them was DMG between the wild and domestic chicken breeds. In contrast, we observed a significant negative correlation between promoter methylation and gene expression level in all samples (Figure 4). Our results suggest that changes in gene expression during domestication could not be explained by the changes in promoter DNA methylation in a global scale.

### DNA mutations between chicken breeds show little influence on DNA methylation variations

Millions of single nucleotide polymorphisms (SNPs) have been identified between different chicken breeds at a frequency of five SNPs per kilobase between RJF and the domestic chicken lines^17^,^18^. We downloaded the SNP data of AA and WL, and analyzed the relationship between DNA mutations and DNA methylation. We did not perform pairwise comparison between RJF and CH due to the lack of re-sequencing data of CH.

**Figure 5:**
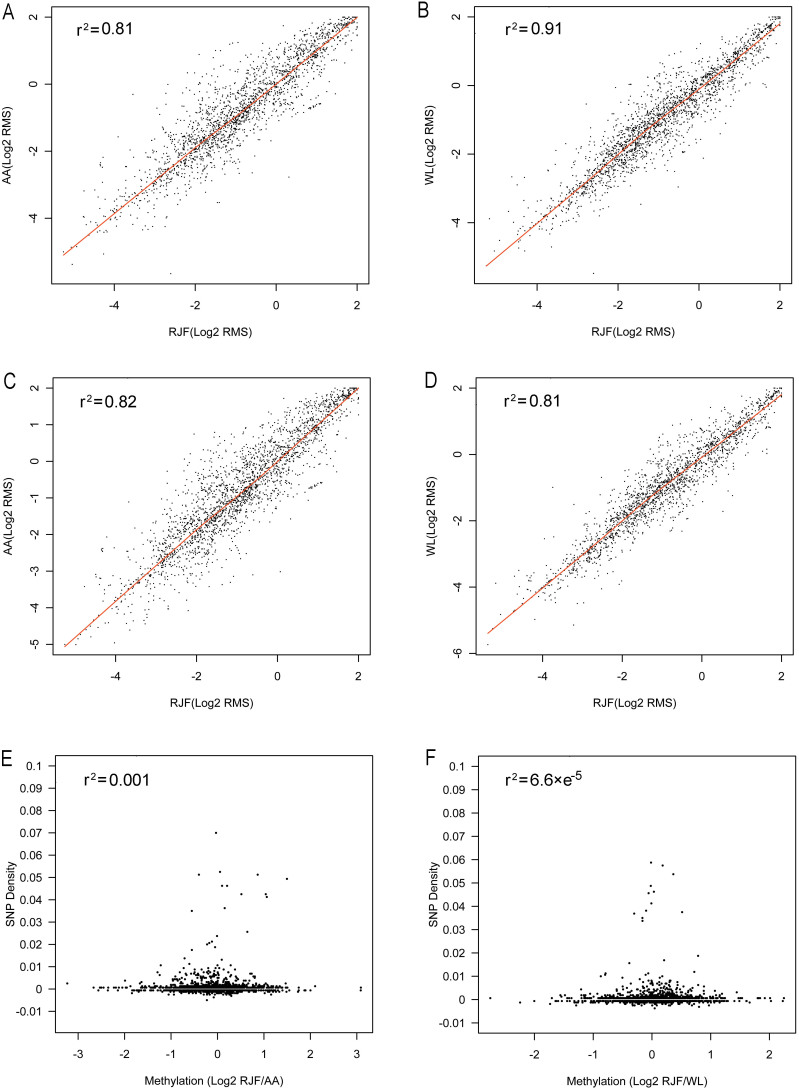
Effect of SNPs on DNA methylation level. Scatter plots showing the correlation of the DNA methylation level of probes to SNP. Each plot represents an SNP site, x and y axis represent RMS of domestic and RJF of corresponding probe. Loss of CG motif: (A) RJF and AA; (B) RJF and WL. Gain of CG motif: (C) RJF and AA; (D) RJF and WL. SNP density and DNA methylation changes. (E) RJF and AA; (F) RJF and WL.

By analyzing the RMS of probes which contained loss and gain of CG motif mutations, respectively, we found that the methylation levels of these probes were still highly correlated between RJF and AA, and between RJF and WL. This observation suggests that the genetic mutations which altered the potential methylation sites do not result in DNA methylation changes (Figure 5A, 5B, 5C and 5D). These SNPs did not result in changes in DNA methylation level of the corresponding probe region in the genome. One possible explanation is the loss or gain of a single methylated CG site is not sufficient to alter the affinity of the methylation antibody to DNA fragments.

Next, we asked whether a mass of SNPs would be able to affect DNA methylation. To this end, we examined the influence of SNP density on DNA methylation variation. All the SNPs that caused a gain or a loss of the CG motif in the 1.6 kb regions probed in the microarray were taken into consideration. SNP density was presented by the ratio of SNP number to the length of the proximal promoter. The methylation state of a promoter was calculated as the average methylation level of all the probes in promoter region. By comparing the correlation of SNP density to methylation level changes between RJF and AA, and between RJF and WL, we found that DNA methylation variations did not show a significant positive correlation with SNP density (Figure 5E and 5F. r^2^ = 0.001 between RJF and AA, r^2^ = 6.6 × e^−5^ between RJF and WL).

### Genes that undergo genetic selection stress show no variations in promoter methylation between the wild and domestic chicken breeds

**Figure 6:**
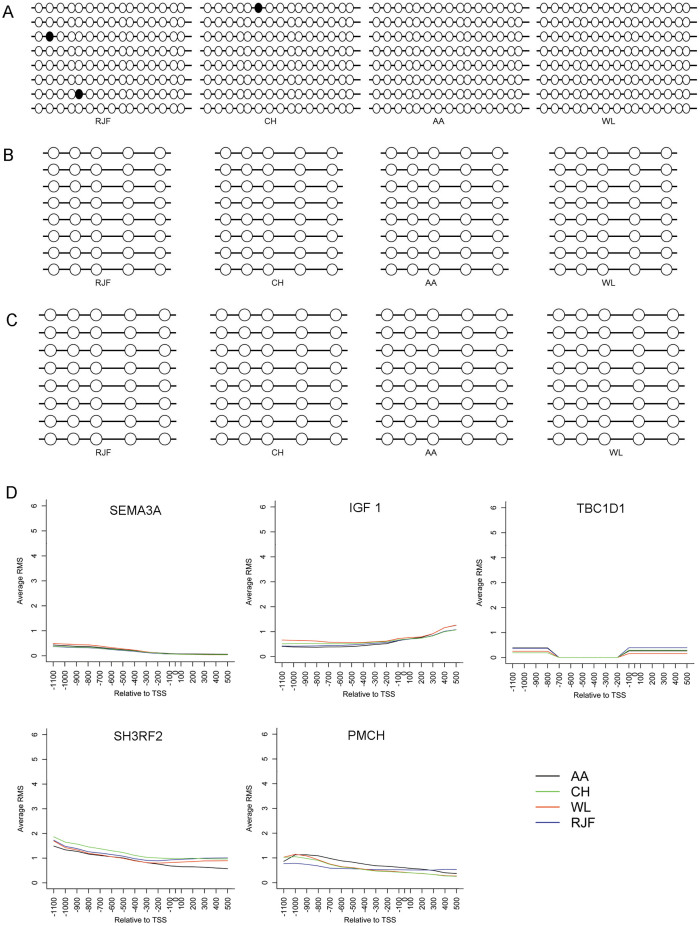
Methylation state of genes that have undergone genetic selection sweeps in four chicken breeds. (A) Bis-seq results of *TSHR* promoter. (B) Bis-seq results of *VSTM2A* promoter. (C) Bis-seq results of *GHR* promoter. (D) MeDIP-chip results of *SEM3A*, *IGF* I, *TBC1D*1, *SH3RF2* and *PMCH*.

A number of genes have been reported to suffer from intensively artificial selection stress during chicken domestication, such as thyroid stimulating hormone receptor (*TSHR*), V-set and transmembrane domain containing 2A (*VSTM2A*), semaphorin (*SEM3A*), insulin-like growth factor I (*IGF* I), and growth hormone receptor (*GHR*)^18^. Genetic selective sweeps have been detected in gene body or upstream region of these genes^18^. We asked if these genes underwent epigenetic selection stress. *TSHR*, *VSTM2A* and *GHR* were selected for bis-seq analysis, and both the meDIP-chip and bis-seq results showed no DNA methylation variations in their proximal promoter regions between the wild and domestic chicken breeds (Figure 6A, 6B, 6C and Supplementary Fig. S8). In addition, the meDIP-chip results showed that there was no obvious DNA methylation variation in promoter regions of genetically selected genes including *SEM3A*, *IGF* I, TBC1 domain family, member 1 (*TBC1D1*), SH3 Domain-Containing RING Finger (*SH3RF2*) and pro-melanin-concentrating hormone (*PMCH*) (Figure 6D).

## Discussion

In this study we report genome-wide promoter methylation patterns in the muscle tissue of the wild and domestic chicken breeds. We systematically analyzed the methylation characteristics of the chicken promoter regions. Importantly, we analyzed the DNA methylation variations between the wild and domestic chicken breeds, the influence of DNA methylation on gene expression changes and the impacts of SNPs on DNA methylation between these breeds. In our study we used meDIP for DNA methylation analysis. MeDIP is widely used in mapping of DNA methylation^1^,^6^,^19^, although it does not give enough resolution as that of single base bisulfite sequencing analysis, it can basically reflect the changes of DNA methylation. MeDIP has many disadvantages, including bias towards high CG content region, requirement of methylated CpG number in limited region, problems in evaluation of methylation level. The greatest concern is on if there is linear relationship between the meDIP value and DNA methylation level. Pelizzola revealed the nonlinear relationship between the meDIP value and DNA methylation level and proposed an analytical methodology called MEDMD^20^, which could improve the evaluation of DNA methylation level based on meDIP data and worked well in real-life data analysis. In our study we adopted this method for our meDIP data analysis and took stringent criteria for differential methylation analysis to avoid false positive results.

Domestication is an evolutionary process during which animals become accustomed to an artificial environment with the intention of selection for human-preferred traits^26^. When animals are moved from a natural to a man-made environment, many traits are undergoing changes including sources and components of feed, and general living conditions^25^. The cumulative influence of the environmental changes accompanying artificial human selection directly resulted in morphological and psychological changes in domestic animals compared with their wild ancestor, such as accelerated growth and development, plumage color variety, and reduced fearfulness towards humans^27^. For example, AA and WL both show higher levels of developmental and reproductive activities compared with RJF. While it is difficult to quantify the extent, the changes between the living environment of the wild and domestic chickens are very dramatic. An interesting question is whether such dramatic environmental changes affect the DNA methylomes of the domestic chickens? Our results reveal that the promoter methylomes are highly conserved between the wild and domestic chicken breeds, and between the domestic chicken breeds themselves.

As the basis of phenotypic variations, many genes are differentially expressed between diverse chicken breeds. Promoter DNA methylation is a well-characterized epigenetic modification that can suppress gene transcription^28^,^29^, raising the question as to whether the changes in DNA methylation contribute to differential gene expression. By analyzing all the DMGs in RJF, CH, AA and WL, we found that there was no obvious negative correlation, suggesting that DNA methylation plays a limited role in shaping the differential gene expression patterns.

DNA methylation occurs mainly in the CpG dinucleotides in animal genomes^28^. Alterations in DNA sequences especially those at CpG motifs may affect the number of methylated cytosines. Seven million SNPs were found in various domestic chicken breeds compared with RJF^18^, many of which are in the CG motifs. First, we found that neither loss nor gain of CG motifs could alter DNA methylation of the corresponding genome regions of the domestic chicken breeds. Second, DNA methylation remained unaltered with the increment of the SNP density in the promoter region. Finally, important growth and development traits related genes that have undergone genetic selection sweeps did not show DNA methylation changes in promoter regions of the studied chicken breeds.

Many reports showed the variations of epigenetic modifications in higher eukaryotes can be stably inherited by offspring, and environmental stimuli may induce epigenetic changes and subsequently have a long-term impact on gene expression^12^,^29^,^30^,^31^. We found only small differences in promoter DNA methylation between the wild and domestic chicken breeds that have been maintained in distinct environments for thousands of years. Furthermore, we showed the conservation of promoter DNA methylome among chicken breeds in several aspects. In our study, only the proximal promoter regions were probed in the microarray. Hence, it is difficult for us to draw a conclusion on the absence of DNA methylation changes on a genome-wide scale. However, methylation of the promoter regions, which are of great importance for gene expression regulation, remained unchanged. We speculate that there were no stable DNA methylation changes during the process of chicken domestication, at least in promoter regions of the muscle genome. Animals exposed to environmental factors may show alteration in the stability of their epigenomes throughout their lifetimes^8^. Previous studies suggested that environmental factors such as nutritional supplements^11^, behavioral cues^32^, reproductive method^33^ and radiation^34^ can induce epigenetic variations. Hence, it is possible that the environmental differences between the wild and domestic chicken breeds were not intense enough to alter the stability of the DNA methylome, or that the initial DNA methylation changes could not be stably inherited. In a broader context, promoter DNA methylation is only one type of the many epigenetic modifications that can regulate the gene expression level^35^,^36^,^37^. Further studies will be needed to elucidate the involvement of epigenetic mechanisms in animal domestication.

## Methods

### Ethics statement

All experiments of this study were carried out under the guidelines of animal welfare committee of China Agricultural University with approval number XK293.

### Animals

Five 7-day-old female chickens were used for each chicken breed in this study. Gastrocnemius taken from each animal was flash frozen in liquid nitrogen and then stored at −80°C.

We performed DNA methylation profiling of proximal promoter regions in the skeletal muscle tissue of chickens using the meDIP-chip assay. Four chicken breeds were profiled: RJF was used to represent the wild chicken breed, whereas CH, AA and WL were selected as representative domestic chicken breeds. Five individuals were analyzed for each breed, except that one individual of the WL was discarded due to the poor data quality.

### MeDIP assay

Genomic DNA was extracted using a DNeasy Blood & Tissue Kit (QIAGEN), and DNA was sheared into 400^–^1000 bp fragments by sonication (Bioruptor, Diagenode). The MeDIP assay was performed as described previously. Briefly, 2 μg sonicated DNA was denatured and incubated with 5 μg mouse monoclonal anti-5-methyl cytidine antibody (Diagenode) in 500 ml IP buffer (0.5% NP40; 1.1% Triton X-100; 1.5 mM EDTA; 50 mM Tris-HCl, 150 mM NaCl) at 4°C for 4 h on a rotating wheel. Then the mixture was incubated with 50 μl of magnetic beads coupled anti-mouse IgG (Bangs laboratories Inc) at 4°C for 2 h by end-over-end rotation, and washed three times with 700 ml IP buffer. Methylated DNA was recovered using proteinase K digestion at 50°C for 3 h, and purified using the phenol-chloroform extraction method followed by ethanol precipitation.

### Array design, data processing, and probe annotation of the arrays for detection of genome-wide promoter methylation

ArrayStar Custom Chicken promoter tiling arrays (Nimblegen) were designed based on the galGal3 genome release which including about 23000 transcripts. The array contains about 390,000 probes with an average length of 60 bp tiled in 62-bp steps along all the Ensemble promoter regions (from about 1.1 kb upstream to 500 bp downstream of the TSS). The MeDIP DNA was labeled by Cy5 and the input DNA was labeled by Cy3. The probe-level log ratio was determined as the log2 of the Cy5/Cy3 channels and used as a measure of MeDIP enrichment. We applied the standard normalization methods, median-centering and quantile normalization by Bioconductor packages Ringo and limma, for two-channel microarrays^38^. After normalization, the normalized log2-ratio data was created for each sample.

### Estimation of the absolute and relative DNA methylation levels, and identification of differentially methylated genes

The absolute and relative methylation score (AMS and RMS) were used to describe the relative methylation level of the proximal promoter regions, which was generated based on the log2 ratio of the probes. The detailed MEDME method for data processing was previously described by Pelizzola *et al*^20^.

The criteria for differentially methylated genes were as follows: 1) the promoter should be located in the high CpG density promoter (HCP) or intermediate CpG density promoter (ICP) (the definitions of HCP, ICP and low CpG density promoter (LCP) are provided in Ref. 1 there must be at least three continuous probes in the promoter region, with more than 2-fold RMS change between breeds, *P* < 0.05, and FDR < 0.05; 3) the average RMS should be >1.5 in one breed and <0.8 in the other breed, according to our definitions of the methylated genes and unmethylated genes.

### GO enrichment analysis

Gene information was downloaded from the public FTP site of Ensembl (ftp.ensembl.org/pub). The information about GO terms was downloaded from the UniProtKB-GOA database. We randomly selected samples of N_f_ different genes at each iteration, and calculated the *P* values for over-representation of the selected genes in all GO biological categories using Fisher's exact test. GO terms with *P* < 0.05 were considered significantly enriched.

### Bisulfite-sequencing

Bisulfite-converted DNA was obtained using the EZ DNA Methylation-Gold Kit^TM^ (Zymo Research) according to the manufacturer's instructions. Semi-nested PCR was carried out for the amplification of specified genomic regions. Primers for bis-seq analysis are available upon request.

### Total RNA isolation and RNA-Seq

A tissue block was ground in liquid nitrogen, and total RNA was extracted with TRIzol® Reagent (Invitrogen) according to the manufacturer's instructions. At least 4 individuals used for the meDIP-chip assay were used for RNA-seq analysis. mRNA was isolated from the total RNA using oligo(dT) magnetic beads. mRNA was interrupted to about 200 bp fragments in a fragmentation buffer. The first strand of cDNA was synthesized using the random hexamer-primer, and the second strand was subsequently synthesized based on the first strand. The double strand cDNA was purified with the QiaQuick PCR extraction kit (Qiagen) and washed with EB buffer for end repair and adenine addition. Finally, sequencing adaptors were ligated to the fragments. The required fragments were obtained using agarose gel electrophoresis and amplified using PCR. The library products were ready for sequencing analysis by Illumina HiSeq™ 2000.

### SNP data used in this study and SNP grouping

The SNP data of the AA and WL were downloaded from: http://www.ensembl.org/biomart/martview/aef928205e592369f2e599bfd31f23f8/aef928205e592369f2e599bfd31f23f8. SNPs were divided into two categories: SNPs which cause loss of CG dinucleotides and gain of CG dinucleotides. The SNPs that resulted in the loss or gain of CG dinucleotides referred to genomic sites that were immediately followed by a G and contained mutations of C to N or N to C (N = A, T, or G).

## Author Contributions

Q.L., Y.Z., X.H. and N.L. conceived and designed the experiments. Q.L. performed the experiments and prepared figures 1–6. Q.L. and Y.W. analyzed the data. Q.L. wrote the paper. All authors reviewed the manuscript.

## Supplementary Material

Supplementary Information Supplementary information file

## Figures and Tables

**Table 1:** GO enrichment of CHMGs and CLMGs

Gene group	GO terms	Number of genes in the gene group/all genes in the GO group	P-value for enrichment
Conserved highly methylated genes	regulation of apoptosis	5/14	0.0078
	catecholamine metabolic process	2/2	0.013
	lipid metabolic process	7/30	0.019
Conserved lowly methylated genes	regulation of transcription, DNA-dependent	182/248	4.59E-05
	G-protein coupled receptor protein signaling pathway	64/142	0.00017
	ubiquitin-dependent protein catabolic process	30/36	0.0067
	protein folding	29/35	0.0093
	vesicle-mediated transport	29/36	0.020
	protein amino acid phosphorylation	135/197	0.032
	post-translational protein modification	17/20	0.039
