# Supplementary material for: Genome-wide Mapping Reveals Conservation of Promoter DNA Methylation Following Chicken Domestication
Source: Sci Rep. 2015 Mar 4;5:8748. doi: 10.1038/srep08748 (PMC4348661; doi:10.1038/srep08748)
Supplement: Supplementary Information — file [file srep08748-s1.pdf]

Supplementary information files

Genome-wide Mapping Reveals Conservation of Promoter DNA Methylation Following Chicken Domestication

Qinghe Li[1,3*], Yuanyuan Wang[2,3 *], Xiaoxiang Hu[3], Yaofeng Zhao[3], and Ning Li[3,4]

[1] *Institute of Animal Sciences, Chinese Academy of Agricultural Sciences, Beijing, 100193, China;*

[2] *Department of Biological Sciences, Bengbu Medical College, Bengbu 233030, China;*

[3] *State Key Laboratory for Agro-biotechnology, China Agricultural University, Beijing 100193, China;* [4] *To whom correspondence should be addressed. E-mail: ninglcau@cau.edu.cn. Tel: 86-10-62731142. Fax: 86-10-62733904. Address: No2 Yuanmingyuan W Rd, Beijing 100193, China*

[*] These authors contributed equally to this work.

Supplemental Figure Legends

Figure S1. Bisulfite-sequencing validation of methylated genes detected by meDIP-chip. (A) Relative methylation scores of the proximal promoter of ENSGALT0000023309 and ENSGALT0000020243. (B) Bis-seq results of the proximal promoter of ENSGALT0000023309. (C) Bis-seq results of the proximal promoter of ENSGALT0000020243. The black circle represents the methylated CpG site and the white circle represents the unmethylated site.

Figure S2. Bisulfite-sequencing validation of unmethylated genes detected by meDIP-chip. (A) Relative methylation scores of the proximal promoter of ENSGALT0000016542 and ENSGALT0000008838. (B) Bis-seq results of the proximal promoter of ENSGALT0000016542. (C) Bis-seq results of the proximal promoter of ENSGALT0000008838.

Figure S3. Bisulfite-sequencing validation of CHMGs identified by meDIP-seq. (A) CAC1B. (B) PTPRU. (C) SCMH1. (D) Q5MB12.

Figure S4. Bisulfite-sequencing validation of CLMGs identified by meDIP-seq. (A) SLC25A39. (B) CDX4. (C) T22D1. (D) MAT3.

Figure S5. Clustering of promoter DNA methylation of all 19 chickens. The DNA methylation level was represented by relative methylation scores, and the RMSs of all genes were used for unsupervised hierarchical clustering.

Figure S6. Validation of differentially methylated genes which proved to be false

positive between various chicken breeds by bis-seq. Differentially methylated genes between the RJF and AA: (A) ENSGALT00000028623; (B) ENSGALT00000014058. Differentially methylated genes between the AA and WL: (C) ENSGALT00000000424; (D) ENSGALT00000031378; (E) ENSGALT00000010200; (F) ENSGALT00000036126; (G) ENSGALT00000040845.

Figure S7. Heatmap shows comparison of DNA methylation level among chicken breeds for 795 individual CpG sites selected randomly from the genome. The DNA methylation level was determined by sequencing 8 clones of bisulfite-treated DNA. Unmethylated CpG sites are shown in white; Methylated CpG sites are shown in red, with color intensity proportional to the methylation level; unavailable data is shown in gray. The pairwise correlation coefficient table shows the similarity of DNA methylation between chicken breeds.

Figure S8. Relative methylation scores of promoter of *TSHR*, *VSTM2A* and *GHR* in the RJF, CH, AA andWL by meDIP-chip.

Figure S1

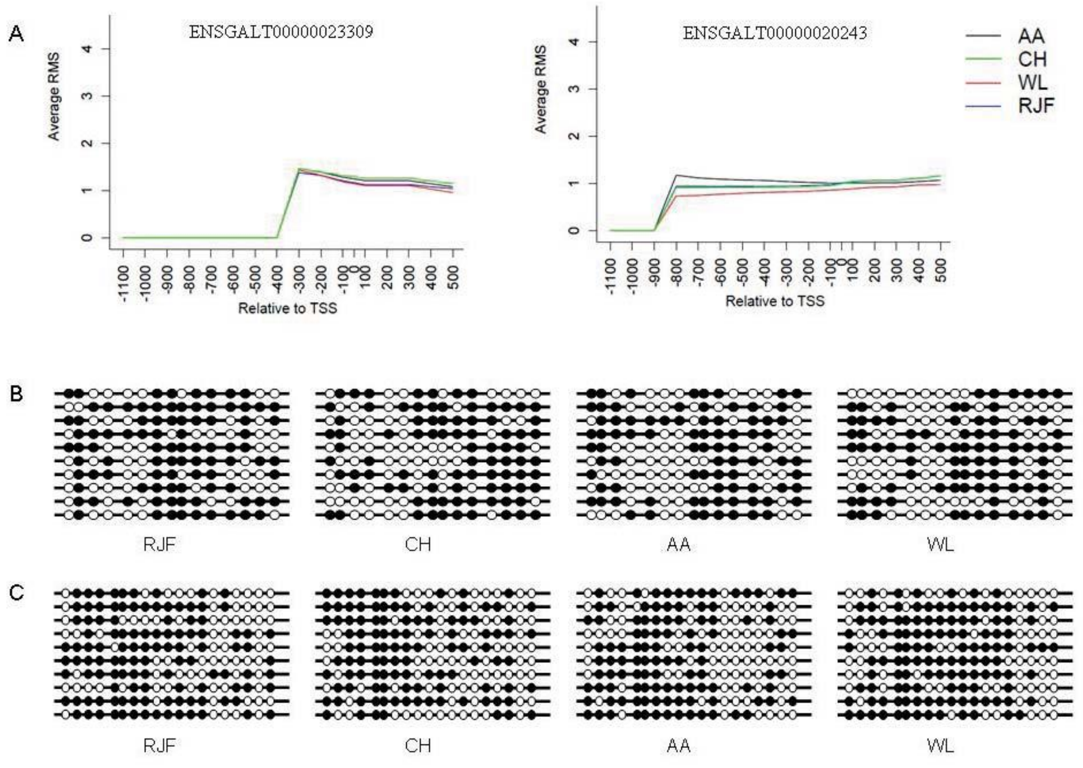

Figure S2

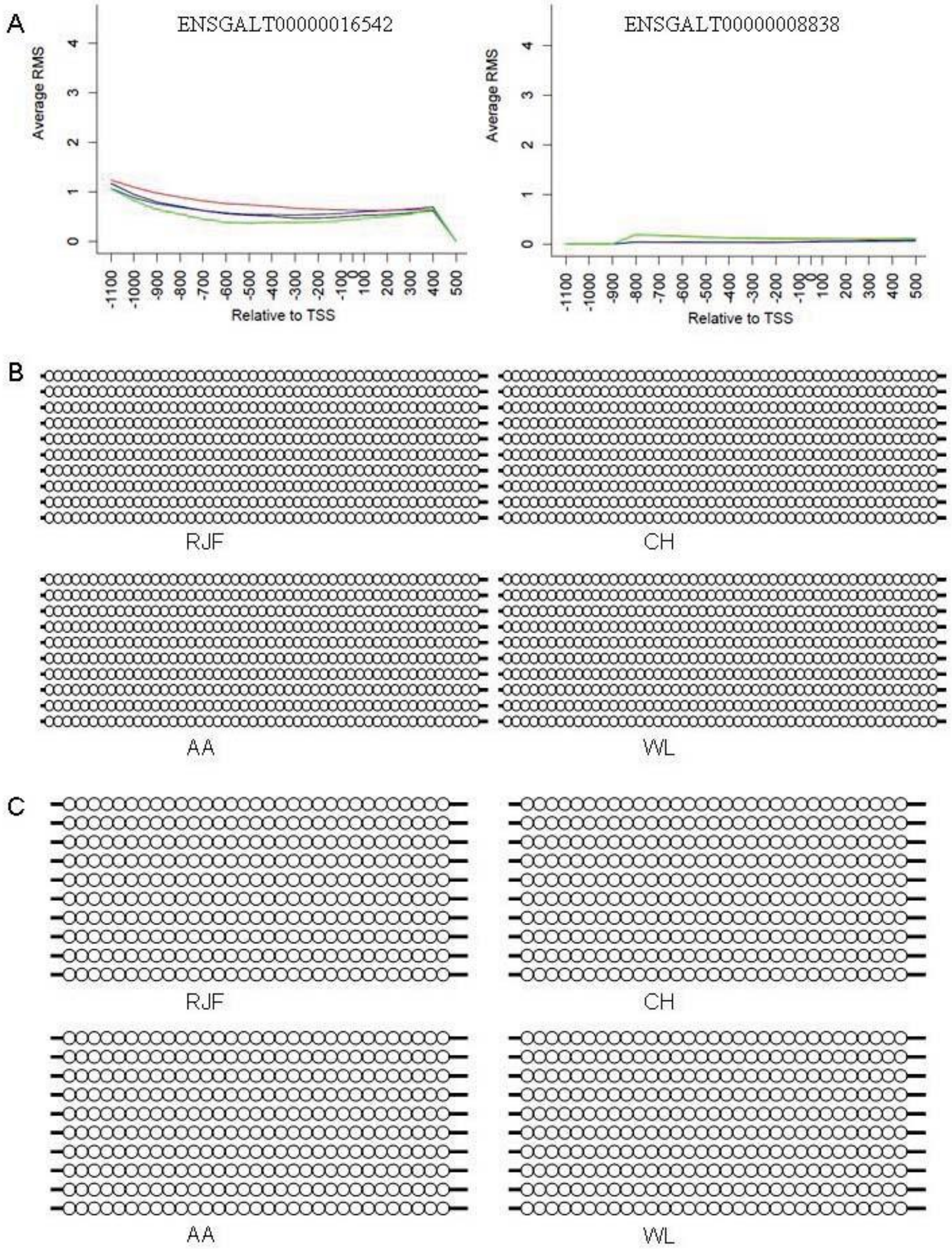
A
ENSGALT00000016542
Average RMS
Relative to TSS
ENSGALT00000008838
Average RMS
Relative to TSS
B
RJF
CH
AA
WL
C
RJF
CH
AA
WL

Figure S3

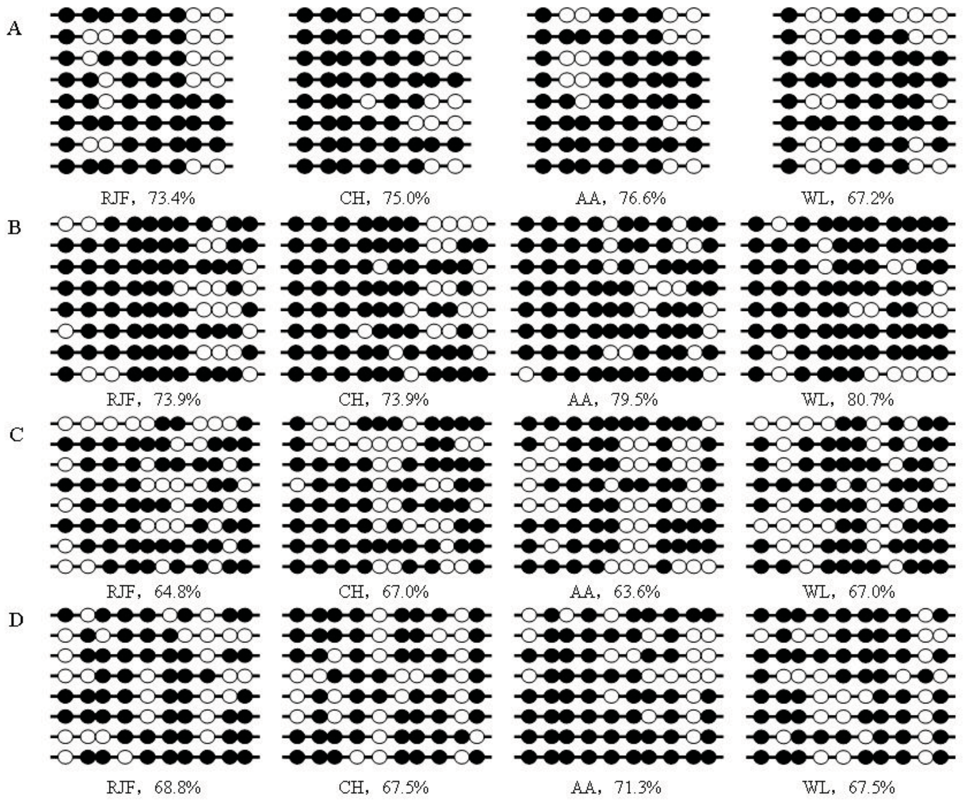
A
RJF，73.4%
CH，75.0%
AA，76.6%
WL，67.2%
B
RJF，73.9%
CH，73.9%
AA，79.5%
WL，80.7%
C
RJF，64.8%
CH，67.0%
AA，63.6%
WL，67.0%
D
RJF，68.8%
CH，67.5%
AA，71.3%
WL，67.5%

Figure S4

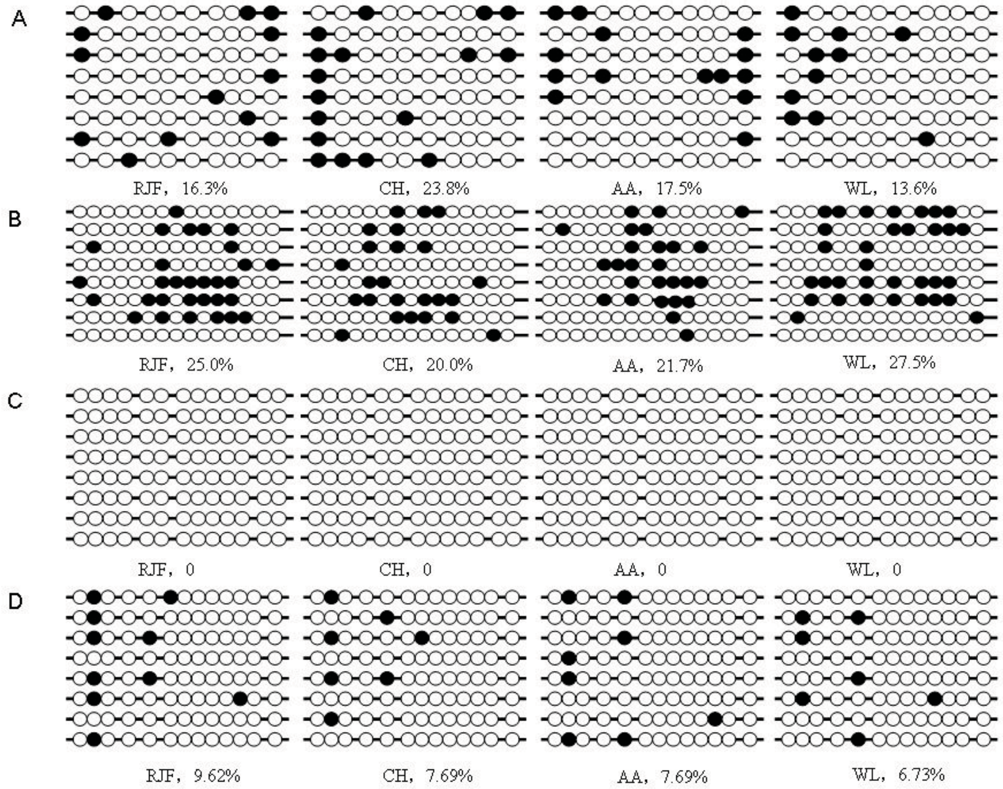
A
RJF，16.3%
CH，23.8%
AA，17.5%
WL，13.6%
B
RJF，25.0%
CH，20.0%
AA，21.7%
WL，27.5%
C
RJF，0
CH，0
AA，0
WL，0
D
RJF，9.62%
CH，7.69%
AA，7.69%
WL，6.73%

Figure S5

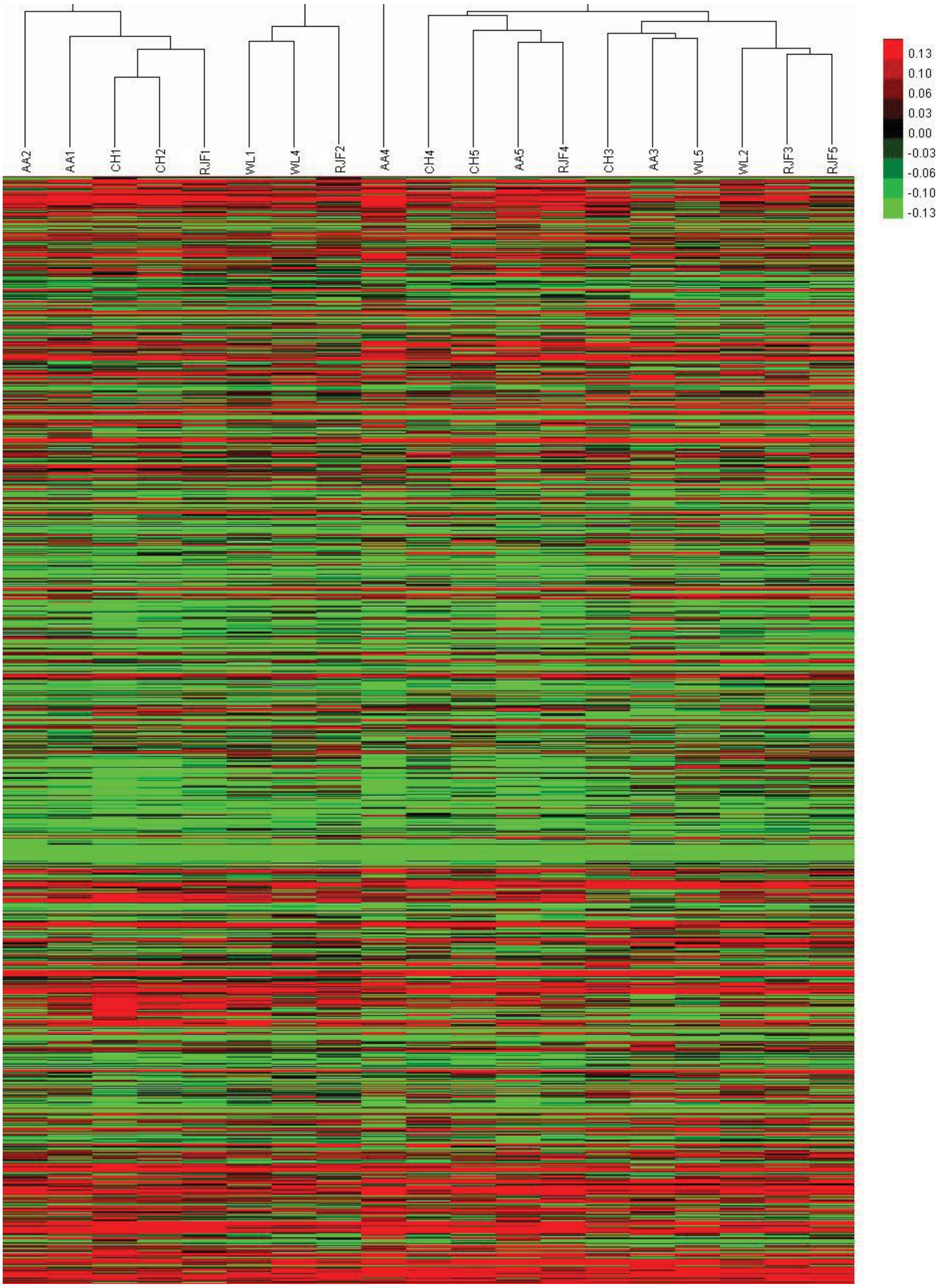

Figure S6

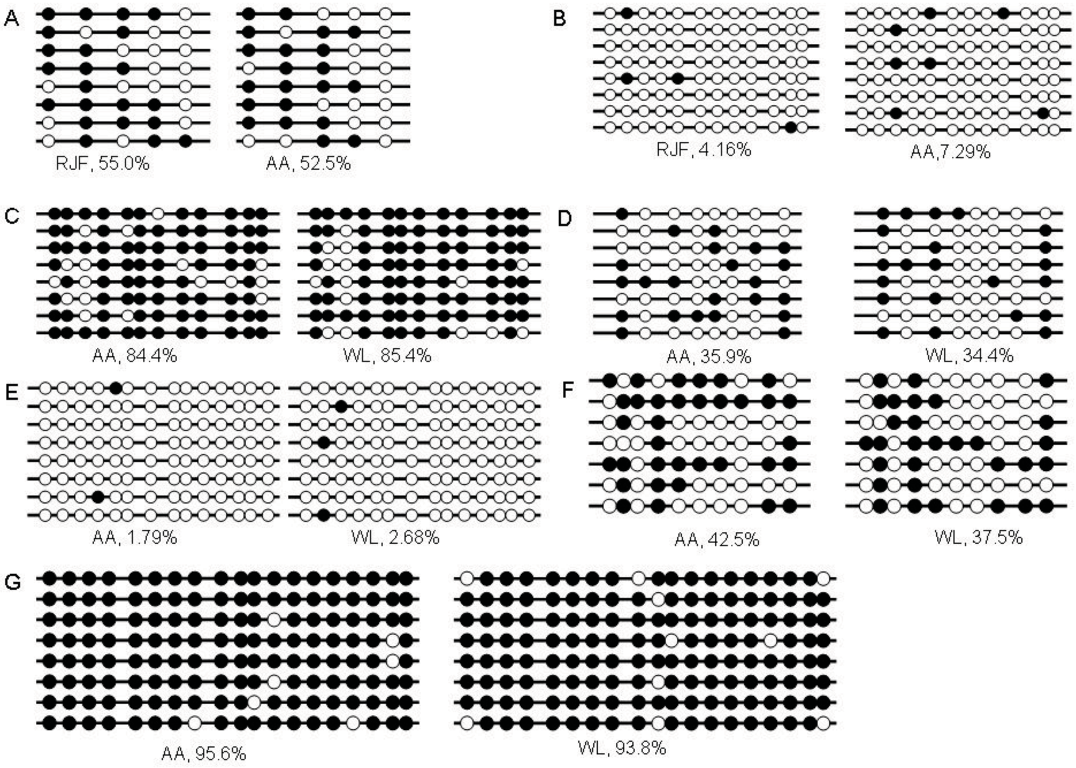

Figure S7

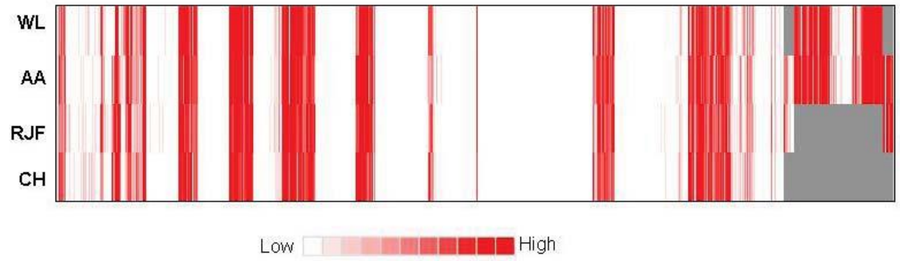

CH	RJF	AA	WL	
1	0.932	0.933	0.910	CH
	1	0.934	0.920	RJF
		1	0.937	AA
			1	WL

Figure S8

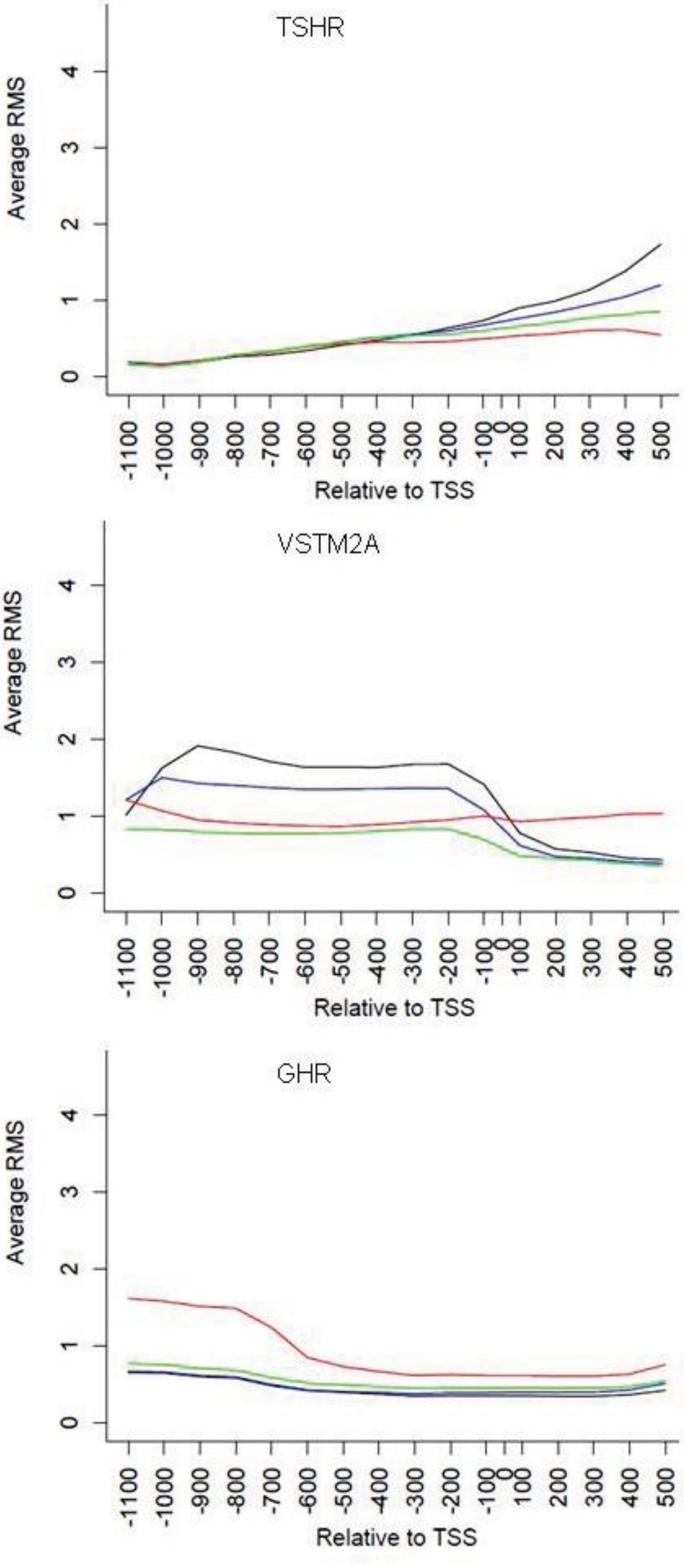
TSHR
Average RMS
0
1
2
3
4
-1100
-1000
-900
-800
-700
-600
-500
-400
-300
-200
-100
0
100
200
300
400
500
Relative to TSS
VSTM2A
Average RMS
Relative to TSS
GHR
Average RMS
Relative to TSS

Supplementary table 1. Conserved highly methylated genes and conserved lowly methylated genes between wild and domestic chicken breeds.

conserved highly methylated genes		conserved lowly methylated genes	
gene name		gene name	
IPI00582379.2	CAC1B_CHICK	NP_989455.1	IPI00595951.3
IPI00598325.2	CAC1S_CHICK	PAX6_CHICK	PTN1_CHICK
NP_990255.1	IPI00818883.1	MYCN_CHICK	IPI00821238.1
Q9W6F1_CHICK	IPI00602405.3	HSF2_CHICK	IPI00579178.2
IPI00601756.3	IPI00572260.3	IPI00684164.3	IPI00585584.1
Q9I9A1_CHICK	TBA5_CHICK	XR_026852.1	IPI00598654.2
IPI00581939.2	IPI00684141.2	NP_001026079.1	PTPRU_CHICK
IPI00819296.1	IPI00591213.3	IPI00684197.1	IPI00683221.2
LIPL_CHICK	IPI00818931.1	IPI00581259.3	IPI00596758.3
IPI00595677.2	IPI00679593.3	IPI00587069.3	O42253_CHICK
Q8UWH4_CHICK	IPI00588910.3	Q8UVD8_CHICK	IPI00598635.3
IPI00603812.3	IPI00923820.1	NP_001012900.1	IPI00592428.3
IPI00576588.3	IPI00582347.2	NP_001025507.1	IPI00578239.2
B3TZC0_CHICK	IPI00603026.3	MYF5_CHICK	NP_990738.1
XR_027237.1	IPI00818951.1	IPI00818973.1	Q91969_CHICK
IPI00599405.1	IPI00681245.2	Q9PTK0_CHICK	SMAD5_CHICK
IPI00576976.3	Q5ZJA5_CHICK	HXA9_CHICK	NP_001019997.1
41_CHICK	Q5ZJA5_CHICK	IPI00592130.3	NP_989892.1
IPI00575887.2	IPI00811176.2	IPI00593183.3	Q56IA0_CHICK
IPI00572954.3	IPI00574878.3	Q9YGP8_CHICK	TGFR2_CHICK
IPI00588910.3	GBRG4_CHICK	IPI00587855.3	IPI00572212.3
LOC429840	JAK2_CHICK	IPI00813273.1	XR_027234.1
IPI00588662.2	IPI00573310.3	IPI00593469.4	IPI00819554.1
ASSY_CHICK	IPI00598751.1	GATA6_CHICK	TDRD3_CHICK
NP_001012901.1	IPI00589711.3	NFKB1_CHICK	IPI00820435.1
IPI00883233.1	NP_989516.1	IPI00811057.2	NP_001026106.1
MUSK_CHICK	IPI00588585.3	IPI00573704.3	IPI00582553.2
NP_989516.1	Q9YI46_CHICK	NP_001032915.1	NP_990006.1
NP_990231.1	IPI00583429.3	NP_989557.1	NP_001032346.1
NP_990361.1	IPI00822099.1	IPI00681554.2	NP_001032351.1
IPI00598120.3	IPI00820240.1	REL_CHICK	Q3L248_CHICK
MOT3_CHICK	NP_001025776.1	NP_001025866.1	NP_989667.1
IPI00591373.2	NP_001026133.1	Q9YHW7_CHICK	Q3L250_CHICK
IPI00603080.3	IPI00823338.1	NP_990547.1	WNT9A_CHICK
IPI00581939.2	NP_990264.1	Q9DGG1_CHICK	IPI00588858.2
B3TZC0_CHICK	IPI00820356.1	IRF1_CHICK	Q5ZI90_CHICK

SPP24_CHICK	Q90715_CHICK	IPI00596933.2	IPI00587489.4
XR_026966.1	IPI00811921.2	IPI00587901.3	IPI00822922.1
Q9W6J0_CHICK	NP_989998.1	HHEX_CHICK	Q8AY19_CHICK
IPI00579614.2	IPI00583002.3	NP_990226.1	IPI00588817.2
IPI00601177.2	B3TZC0_CHICK	Q5ZMD5_CHICK	TRPC7
IPI00594166.3	Q9I8D3_CHICK	IPI00819883.1	CAC1B_CHICK
NP_001006325.1	NP_990533.1	SMAD5_CHICK	IPI00811977.2
XR_027135.1	CHST3_CHICK	IRF2_CHICK	IPI00595398.3
TEAD3_CHICK	IPI00811970.2	IPI00574747.3	ACHA5_CHICK
IPI00577484.3	Q5F3F8_CHICK	NP_990424.1	IPI00821979.1
STAT3_CHICK	IPI00684792.2	IPI00600306.2	Q9DEX2_CHICK
IPI00685033.2	IPI00583922.3	IPI00573114.3	NP_990545.1
IPI00579018.2	IPI00822779.1	IPI00820558.1	IPI00603425.3
IPI00589786.2	NP_989451.1	NP_989781.1	IPI00598119.3
NP_989444.1	IPI00598333.2	Q9YHW5_CHICK	A0M8U4_CHICK
IPI00594235.2	NP_001020005.1	HXA11_CHICK	IPI00819902.1
IPI00594192.2	IPI00821317.1	IPI00684025.2	Q8AV15_CHICK
NP_990051.1	IPI00588225.2	DMRT1_CHICK	IPI00820292.1
NP_989922.1	IPI00601442.4	IPI00680566.2	IPI00571508.3
IPI00600142.2	IPI00821827.1	IPI00584637.3	LOC430136
NP_001007079.1	NP_001009878.1	ANF1_CHICK	GBRB3_CHICK
CCL4_CHICK	IPI00579118.2	NP_001019997.1	IPI00575771.3
NP_998752.1	IPI00599589.2	XR_026766.1	Q90590_CHICK
IPI00822006.1	Q5ZMK6_CHICK	IPI00820569.1	IPI00589286.3
NP_989998.1	NP_001026661.1	NP_990429.1	NP_001004409.1
IPI00597131.2	NP_001034349.1	IPI00818195.1	NP_996862.1
IPI00582635.3	IPI00581601.3	IPI00597240.3	Q9PUI8_CHICK
IPI00810591.2	Q98SE8_CHICK	NP_997060.1	NP_989903.1
IPI00818613.1	IPI00588662.2	BMAL2_CHICK	IPI00602335.3
IPI00822606.1	IPI00820134.1	NPAS2_CHICK	IPI00812061.1
IPI00682695.2	B9VGZ4_CHICK	IPI00818178.1	O42421_CHICK
IPI00681326.2	IPI00603868.3	NP_001006192.1	CAC1C_CHICK
IPI00600235.3	IPI00603868.3	Q90692_CHICK	IPI00573170.2
IPI00585170.3	IPI00680595.1	HXB5_CHICK	NP_989657.1
XR_026898.1	IPI00579614.2	TCP4_CHICK	IPI00684904.2
IPI00573538.3	HBAD_CHICK	NP_001025509.1	NP_996757.1
NP_990255.1	IPI00683979.2	IPI00589286.3	IPI00587021.3
MOT3_CHICK	NP_001026461.1	NP_989784.1	IPI00600977.2
IPI00598902.3	IPI00814019.2	NP_990248.1	IPI00821254.1
IPI00596658.3	IPI00813937.2	IPI00591458.2	NP_001001774.1
IPI00820646.1	IPI00581849.2	IPI00577997.3	ACHB4_CHICK
NP_001007083.1	IPI00813977.2	MTA3	GBRG2_CHICK

LOC429840	IPI00822658.1	IPI00581941.2	IPI00599050.2
NP_001012901.1	IPI00573310.3	IPI00822713.1	IPI00600831.3
NP_990023.1	IPI00582347.2	Q9I9A0_CHICK	IPI00579185.1
XR_026842.1	IPI00812731.2	IPI00581321.3	O42418_CHICK
XR_026797.1	NP_001026044.1	Q8UVS4_CHICK	IPI00592966.3
IPI00592554.3	IPI00602372.2	IPI00595711.3	NP_989622.1
CAC1S_CHICK	Q6EE28_CHICK	LHX9_CHICK	IPI00571830.2
IPI00580553.3	IPI00574878.3	FOXP1_CHICK	IPI00682164.2
IPI00578050.2	IPI00601317.2	NP_001007082.1	IPI00683221.2
CAC1B_CHICK	Q5MB12_CHICK	Q5ZJU1_CHICK	IPI00578166.3
XR_027000.1	STXB1_CHICK	NP_989947.1	IPI00575434.2
IPI00585829.3	IPI00822695.1	IPI00571967.3	IPI00819156.1
IPI00684598.2	NP_001026133.1	IPI00588986.3	IPI00573257.4
NP_990449.1	IPI00819296.1	PIT1_CHICK	IPI00594975.3
Q5F3F8_CHICK	IPI00581109.2	IPI00584692.3	IPI00581122.2
Q9I9L4_CHICK	Q9I9A1_CHICK	NP_990075.1	IPI00680807.1
PRKDC_CHICK	IPI00680595.1	IPI00586440.3	IPI00581122.2
NP_001019999.1	CP19A_CHICK	TBX5_CHICK	IPI00680807.1
DNAS1_CHICK	NP_001001879.1	XR_026691.1	IPI00581122.2
IPI00581264.3	IPI00586199.3	IPI00581805.2	IPI00680807.1
B9VGZ4_CHICK	IPI00820998.1	IPI00599426.3	IPI00813164.1
Q9I9L4_CHICK	P4HA1_CHICK	PITX1_CHICK	IPI00578253.2
IPI00915707.1	NP_001006539.1	NP_990529.1	MYF5_CHICK
IPI00818883.1	IPI00596674.2	RX1_CHICK	MYF6_CHICK
IPI00818883.1	IPI00818131.1	IPI00599092.3	NP_989749.1
PTPRU_CHICK	IPI00883262.1	NP_990468.1	Q8AV15_CHICK
IPI00596340.2	IPI00580553.3	IPI00595893.3	XR_026692.1
IPI00580149.4	IPI00819512.1	IPI00820211.1	O42421_CHICK
NP_990738.1	MUSK_CHICK	IPI00583810.3	AT1B1_CHICK
NP_001026046.1	IPI00821654.1	NP_990083.1	NP_001038116.1
IPI00593651.3	IPI00683578.2	HOXC10	O42418_CHICK
IPI00581849.2	IPI00600691.2	IPI00585593.3	IPI00600831.3
NP_990071.1	IPI00583816.3	DBX2_CHICK	IPI00579948.2
IPI00813977.2	XR_026966.1	IPI00822726.1	NTCP7_CHICK
Q9W6S4_CHICK	NP_001034685.1	NP_001012899.1	Q5ZKL3_CHICK
IPI00822658.1	PRPF3_CHICK	IPI00588048.1	IPI00579184.3
IPI00574878.3	SIAT2_CHICK	ZFHX4_CHICK	IPI00582562.3
NP_989615.1	IPI00578091.2	T22D1_CHICK	FMN_CHICK
NP_990121.1	IPI00582425.3	IPI00589167.2	IPI00818552.1
IPI00583816.3	IPI00602405.3	IPI00883106.1	IPI00820183.1
IPI00680647.2	XR_026961.1	GATA2_CHICK	NP_989541.1
IPI00811921.2	GBRG4_CHICK	NP_001008480.1	IPI00819058.1

Q9YI46_CHICK	NP_998737.1	IPI00603116.2	NP_001012572.1
IPI00575389.3	GLL9_CHICK	NP_989892.1	NP_001026249.1
IPI00581147.3	NP_001025825.1	Q5IHK1_CHICK	IPI00571442.3
IPI00580198.3	IPI00585123.2	NP_001026113.1	IPI00684538.1
IPI00604389.4	IPI00581147.3	IPI00822861.1	NP_001026375.1
XR_026966.1	IPI00594309.3	IPI00582267.3	IPI00680633.1
IPI00820646.1	IPI00576188.3	TBXT_CHICK	IPI00818548.1
IPI00574878.3	EYA3_CHICK	Q804C5_CHICK	LOC768699
Q9I9A1_CHICK	IPI00819539.1	RABL3_CHICK	PIMT_CHICK
IPI00570973.3	NP_001026044.1	PO2F1_CHICK	IPI00588987.3
IPI00573310.3	Q9I9L4_CHICK	IPI00600145.2	NP_001034387.1
IPI00683578.2	IPI00822849.1	IPI00684173.2	IPI00593095.4
IPI00602313.2	IPI00585123.2	NP_001012932.1	IPI00593079.4
NP_001025776.1	IPI00594309.3	ATF2_CHICK	IPI00602488.2
MUSK_CHICK	IPI00582425.3	MYF6_CHICK	IPI00581174.1
JAK2_CHICK	IPI00820202.1	Q6DV99_CHICK	NP_001026138.1
IPI00572942.2	STXB1_CHICK	THB_CHICK	Q98SE8_CHICK
IPI00582379.2	IPI00820134.1	Q98TX8_CHICK	IPI00593770.1
IPI00589446.2	IPI00570702.1	NP_001012971.1	IPI00604167.3
IPI00821654.1	IPI00820202.1	LOC777298	O42347_CHICK
IPI00588134.2	DJC16_CHICK	JUN_CHICK	IPI00587316.3
Q6EE28_CHICK	TBA5_CHICK	HMGX7_CHICK	IPI00821253.1
IPI00598902.3	CAC1S_CHICK	RARB_CHICK	IPI00601634.2
IPI00602664.3	CAC1B_CHICK	IPI00600721.3	Q5ZK22_CHICK
LOC429840	IPI00598329.3	HMX1_CHICK	IPI00682165.2
NP_001007083.1	IPI00822606.1	IPI00591858.4	IPI00574019.3
GBRG4_CHICK	IPI00603812.3	NP_989437.1	IPI00595617.2
NP_990023.1	IPI00820913.1	IRF3_CHICK	IPI00595358.4
IPI00580553.3	IPI00602405.3	IPI00579574.1	IPI00602532.4
		MEF2A_CHICK	Q9YH83_CHICK
		Q5ZJZ7_CHICK	NP_001006408.1
		HMX3_CHICK	NP_001034361.1
		IPI00582563.4	NP_001026517.1
		IPI00819684.1	NP_001026111.1
		IPI00585822.3	NP_001006178.1
		IPI00598714.3	IPI00823190.1
		HIRA_CHICK	IPI00822156.1
		Q56IA0_CHICK	NP_001034378.1
		DLX5_CHICK	IPI00586051.2
		HSF3_CHICK	Q5ZMQ6_CHICK
		HES1_CHICK	IPI00592219.2
		IPI00818474.1	NP_001029995.1

IPI00682833.1	IPI00592615.3
Q9W6V3_CHICK	IPI00584834.3
NP_001012867.1	ERCC3_CHICK
IPI00590021.3	Q5ZJA5_CHICK
IPI00684235.2	FRIH_CHICK
NP_989873.1	NP_990538.1
IPI00588035.3	NP_001012931.1
NP_001025747.1	IPI00598923.3
NP_001026089.1	NP_001026165.1
HMD2_CHICK	IPI00587790.2
COT2_CHICK	IPI00812875.2
XR_027111.1	IPI00596143.2
VAX1_CHICK	NP_989717.1
NP_001039307.2	IPI00600118.2
IPI00822649.1	Q9DGH8_CHICK
IPI00579183.2	IPI00811977.2
TEAD4_CHICK	IPI00602335.3
IPI00591072.2	IPI00812061.1
NP_990134.1	GBRG2_CHICK
IPI00819473.1	NP_001025543.1
IPI00598916.2	JAK2_CHICK
NP_001026301.1	IPI00814205.2
IPI00819110.1	NP_990201.1
GNAI1_CHICK	IPI00596042.3
IPI00812390.2	HMCS1_CHICK
IPI00594142.3	IPI00572129.2
Q8QGC2_CHICK	NP_989816.1
IPI00592368.2	IPI00572039.3
IPI00602218.2	VATB_CHICK
IPI00599896.3	NP_989617.1
IPI00818522.1	IPI00599296.1
IPI00811726.2	NP_001006281.1
LOC776109	IPI00577304.2
IPI00820253.1	IPI00574801.3
NP_001026186.1	BLM_CHICK
IPI00820683.1	NP_989631.1
IPI00818964.1	IPI00591558.3
IPI00590976.3	NP_001006183.1
IPI00821858.1	IPI00571304.3
B8YLW8_CHICK	IPI00602959.3
NP_001012626.1	IPI00575167.3
IPI00573243.1	Q98SN6_CHICK

IPI00597246.2	Q98SN5_CHICK
IPI00821150.1	NP_001012846.1
IPI00814458.2	IPI00818080.1
IPI00811748.2	IPI00819012.1
IPI00595552.2	IPI00573005.3
IPI00822075.1	IPI00819012.1
IPI00822483.1	IPI00573005.3
B4X7J8_CHICK	IPI00680942.2
IPI00584101.1	IPI00683861.2
IPI00590757.2	IPI00813935.2
IPI00604290.2	NP_001006128.1
IPI00812092.1	XR_027146.1
OLF6_CHICK	NP_989634.1
IPI00680065.2	PUR2_CHICK
IPI00813698.2	Q9I9L4_CHICK
IPI00811681.2	IPI00604192.3
IPI00599818.3	PYRG2_CHICK
IPI00814014.2	IPI00587904.2
IPI00681022.2	Q9DEI0_CHICK
IPI00812432.2	KU70_CHICK
IPI00591114.1	PRKDC_CHICK
XR_026927.1	IPI00588538.3
IPI00594533.1	IPI00603535.1
IPI00594681.3	SYIM_CHICK
IPI00683370.2	IPI00581192.3
IPI00813684.2	IPI00574953.3
IPI00586811.3	IPI00595228.3
IPI00819799.1	NP_001039306.1
IPI00822579.1	NP_001026419.1
Q9PTB1_CHICK	IPI00595672.2
IPI00578651.1	IPI00599090.4
IPI00598001.2	NP_001006449.1
IPI00585336.3	IPI00581279.1
IPI00818625.1	IPI00574749.2
IPI00818636.1	Q5F3K9_CHICK
IPI00597885.2	NP_001028816.1
IPI00599070.1	IPI00590522.1
IPI00822268.1	IPI00571526.3
IPI00572187.3	IPI00684413.2
IPI00680211.2	Q9IAA6_CHICK
NP_990267.1	GEPH_CHICK
IPI00594769.2	RAN_CHICK

NP_001006530.1	IPI00820543.1
IPI00819354.1	IPI00573393.3
IPI00584907.3	IPI00598758.2
IPI00680945.2	IPI00584639.3
IPI00681234.2	FAK1_CHICK
NP_001026578.1	XR_026865.1
IPI00582057.3	IPI00578493.3
UBP7_CHICK	DMRT1_CHICK
NP_989539.1	IPI00581941.2
IPI00579016.3	IPI00582808.4
NP_001006130.1	KIT_CHICK
IPI00582957.1	FGFR2_CHICK
LOC771424	FGFR3_CHICK
NP_997702.1	IPI00574892.2
XR_027094.1	IPI00587195.3
IPI00819480.1	PUR2_CHICK
NP_001026141.1	Q9I9L4_CHICK
IPI00574592.3	IPI00812569.2
NP_001006134.1	IPI00572041.2
IPI00592203.4	IPI00577839.1
NP_001026250.1	PRPS2_CHICK
IPI00598742.2	MTDC_CHICK
IPI00590577.3	XR_027102.1
XR_027086.1	XR_027140.1
IPI00573052.3	IPI00682187.2
Q8JHZ7_CHICK	NP_001006294.1
PSA1_CHICK	NP_001006202.1
Q9DFY9_CHICK	Q91429_CHICK
NP_001026294.1	XR_027092.1
IPI00591077.3	Q91429_CHICK
HS90B_CHICK	XR_027092.1
PPIB_CHICK	NP_001026732.1
ENPL_CHICK	IPI00588119.1
IPI00600671.2	NP_001025711.1
IPI00581078.3	LDHA_CHICK
IPI00575389.3	IPI00820902.1
IPI00598319.2	IPI00592631.1
NP_001006250.1	IPI00821229.1
NP_001012581.1	IPI00595685.3
IPI00602969.1	Q91429_CHICK
IPI00579432.3	XR_027092.1
NP_001025723.1	VATB_CHICK

Q90634_CHICK	NP_989617.1
IPI00594320.3	NP_990363.1
IPI00603058.5	NP_001026601.1
IPI00571105.1	IPI00591811.3
NP_001026546.1	NP_001026125.1
NP_001026395.1	IPI00578026.1
NP_990398.1	IPI00592265.3
IPI00591376.3	NP_990190.1
IPI00572589.2	NP_990189.1
TCPQ_CHICK	NP_001012626.1
IPI00822858.1	IPI00596756.3
NP_001020780.1	NP_989772.1
IPI00821442.1	LOC768848
NP_001005841.1	IPI00597292.2
IPI00603388.4	Q5ZJE8_CHICK
NP_001005431.1	IPI00598554.3
NP_989972.1	IPI00581647.4
NP_001025780.1	IPI00590695.2
Q5ZIM9_CHICK	NP_001006496.1
IPI00598641.3	NP_001025536.1
NP_001026088.1	Q98946_CHICK
IPI00596524.2	IPI00819162.1
NP_001026764.1	Q91015_CHICK
NP_001025179.1	NP_001006390.1
IPI00592452.3	IPI00603013.3
NP_001012914.1	FYN_CHICK
NP_001012877.1	IPI00579079.2
NP_001012961.1	IPI00580681.2
IPI00575458.1	IPI00582590.2
IPI00578203.2	RAB10_CHICK
NP_001006261.1	IPI00572853.2
NP_001034575.1	IPI00589508.2
IPI00577874.1	IPI00594950.3
XR_026901.1	IPI00582273.3
IPI00589670.2	Q90YX6_CHICK
NP_001006586.1	IPI00589108.3
Q5ZMT6_CHICK	RAB6A_CHICK
IPI00813204.2	IPI00601244.1
IPI00604093.1	IPI00582335.1
IPI00597800.2	IPI00594309.3
IPI00597412.2	CHMP7_CHICK
IPI00586708.3	IPI00603242.2

IPI00596855.3	IPI00821845.1
LOC769161	NP_001026240.1
IPI00572599.2	IPI00601632.3
IPI00598430.2	IPI00570706.3
Q91969_CHICK	IPI00579759.1
IPI00571773.2	XR_026693.1
NP_001025543.1	NP_989543.1
IPI00814085.1	NP_989760.1
IPI00818166.1	NP_001026187.1
IPI00591988.3	IPI00820960.1
NP_990688.1	IPI00591992.3
IPI00590707.3	NP_001033782.1
KC1A_CHICK	IPI00583618.3
NP_001004368.1	XR_026928.1
NP_989979.1	IPI00818895.1
IPI00588613.2	NP_001026163.1
IPI00594257.3	IPI00592525.3
FGFR2_CHICK	NP_989432.1
XR_027234.1	IPI00598807.3
IPI00588803.3	IPI00594788.2
NP_990386.1	IPI00575586.3
Q5ZHL2_CHICK	Q5ZMV1_CHICK
IPI00813807.2	IPI00591196.2
JAK2_CHICK	IPI00821350.1
IPI00596460.3	XR_026654.1
IPI00587022.3	IPI00573182.3
NP_001012805.1	IPI00602389.3
NP_989691.1	NP_001026554.1
Q9PT87_CHICK	IPI00597610.2
IPI00822302.1	XR_027032.1
IPI00820353.1	FMN_CHICK
NP_989481.1	CAZA2_CHICK
IPI00591806.3	IPI00818552.1
IPI00598794.2	Q5ZL50_CHICK
KIT_CHICK	IPI00820183.1
IPI00596027.2	XIRP1_CHICK
IPI00821165.1	NP_989541.1
IPI00819902.1	IPI00819058.1
IPI00577164.3	IPI00681698.1
NP_001020782.1	CAZA1_CHICK
XR_026953.1	IPI00680520.2
IPI00588347.3	NP_001039306.1

IPI00575500.2	IPI00593676.2
IPI00814205.2	Q2WBI1_CHICK
IPI00589314.3	PXDNL
IPI00681290.2	IPI00822052.1
IPI00810781.2	NP_001029998.1
NP_001012547.1	IPI00590608.2
IPI00585752.1	PARP1_CHICK
IPI00572427.3	XR_026755.1
NP_001026490.1	IPI00684065.2
FGFR3_CHICK	IPI00683320.2
IPI00683858.2	IPI00823170.1
NP_989733.1	IPI00579011.2
IPI00578060.3	IPI00580979.2
NP_001026435.1	IPI00572821.1
EPHA3_CHICK	NP_001026764.1
VGFR1_CHICK	IPI00582590.2
NP_001026508.1	IPI00589670.2
IPI00594674.3	IPI00813204.2
IPI00582628.1	Q5ZIM9_CHICK
IPI00573527.3	IPI00583677.3
NP_001026042.1	HS90B_CHICK
NP_001006133.1	NP_989979.1
Q8QGH1_CHICK	ENPL_CHICK
IPI00595478.3	IPI00579557.1
IPI00573170.2	FZD6_CHICK
IPI00598376.2	SMO_CHICK
NP_001025988.1	FZD3_CHICK
KC1G1_CHICK	FZD10_CHICK
EPHA5_CHICK	FZD1_CHICK
XR_027077.1	IPI00583166.3
NP_001026111.1	IPI00820886.1
Q98SN6_CHICK	NP_001026363.1
TYRO3_CHICK	IPI00820659.1
IPI00591467.2	IPI00587190.3
Q5ZL78_CHICK	CRYAA_CHICK
NP_001034694.1	IPI00584799.1
IPI00578943.3	IPI00811415.2
IPI00571400.3	NP_001005841.1
XR_027075.1	HSPB1_CHICK
IPI00587876.2	NP_001007976.1
IPI00599490.2	IPI00819528.1
IPI00820514.1	NP_001026053.1

NP_990201.1	IPI00822695.1
NP_001006227.1	IPI00585641.3
Q5ZLJ0_CHICK	CHD7_CHICK
IPI00582579.3	ASF1_CHICK
IPI00579085.2	IPI00598956.2
NP_001025909.1	NP_001012920.1
IPI00587758.3	NP_989974.1
Q98SN5_CHICK	NP_990272.1
IPI00603018.4	NP_989537.1
IPI00593292.3	ACAP2_CHICK
NP_990521.1	SMAP2_CHICK
NP_989626.1	NP_001012557.1
IPI00822237.2	IPI00594909.3
IPI00822035.1	IPI00600935.3
NP_001025941.1	NP_001025725.1
IPI00575631.2	LOC776461
IPI00596751.3	IPI00592666.3
NP_001026372.1	IPI00594463.3
XR_026673.1	NM_001004400.2
BRAF1_CHICK	IPI00575200.3
CHK1_CHICK	IPI00598915.2
IPI00602834.3	IPI00592961.3
IPI00818448.1	TBA2_CHICK
Q90960_CHICK	IPI00576041.1
IPI00580228.3	IPI00822232.1
IPI00572424.4	IPI00603435.3
KCC2D_CHICK	Q2MHD5_CHICK
IPI00597506.2	IPI00600743.2
Q8AV70_CHICK	IPI00593687.3
NP_001026288.1	TBA4_CHICK
IPI00596668.3	XR_027195.1
IPI00584639.3	IPI00590371.3
IPI00582379.2	IPI00590953.2
IPI00596416.3	NP_001025793.1
NP_001026568.1	Q6V1L3_CHICK
BMR1B_CHICK	IPI00575336.2
IPI00574949.2	TBA1_CHICK
MK06_CHICK	IPI00822617.1
IPI00582849.1	IPI00589956.4
FAK1_CHICK	IPI00594667.3
IPI00571158.3	LOC776808
TGFR2_CHICK	IPI00821841.1

IPI00818088.1	TBB3_CHICK
IPI00577961.3	IPI00684333.2
STRAA_CHICK	IPI00821488.1
NP_989732.2	NP_001006320.1
IPI00587675.2	IPI00586977.4
IPI00683739.3	IPI00821889.1
IPI00585611.3	NP_001034355.1
IPI00576002.3	Q7T191_CHICK
PGFRA_CHICK	IPI00597302.3
IPI00596551.4	IPI00592228.3
LOC769294	IPI00579895.3
NTRK2_CHICK	IPI00601180.3
IPI00587477.3	LAP4A_CHICK
UBE2W	Q5F386_CHICK
XR_026727.1	IPI00573257.4
IPI00600590.1	IPI00599306.1
IPI00572628.2	IPI00594975.3
NP_001005838.1	IPI00588817.2
IPI00680215.2	IPI00818234.1
Q5ZLH8_CHICK	IPI00582494.3
NP_001012828.1	IPI00819404.1
IPI00681498.2	NM_204467.1
IPI00572066.2	IPI00822922.1
NP_001006180.1	TRPC7
IPI00576435.4	MFSD1_CHICK
NP_001026711.1	CAC1B_CHICK
UBC9_CHICK	KCAB1_CHICK
IPI00593939.1	IPI00580635.1
IPI00819923.1	IPI00581435.3
IPI00578886.3	IPI00598611.1
IPI00818733.1	Q9DEX2_CHICK
IPI00598641.3	S35B1_CHICK
IPI00582808.4	IPI00820629.1
NP_001026306.1	IPI00599491.3
IPI00572560.3	LOC416599
NP_001025179.1	IPI00603425.3
NP_001025792.1	IPI00822252.1
TOM1_CHICK	A0M8U4_CHICK
NP_001012914.1	Q9YH83_CHICK
IPI00584385.2	IPI00820292.1
IPI00577557.3	IPI00598119.3
NP_001012877.1	IPI00595217.2

RAN_CHICK	IPI00603440.2
NM_001030621.1	IPI00596696.1
IPI00577874.1	IPI00819902.1
XR_026901.1	NP_001025750.1
NP_001006261.1	LOC430136
Q5ZKZ1_CHICK	Q8AV15_CHICK
IPI00578318.2	IPI00820495.1
NP_001006586.1	IPI00589286.3
IPI00813364.2	IPI00571508.3
IPI00602929.3	IPI00582510.3
IPI00603305.2	IPI00577173.3
IPI00596397.1	IPI00586650.2
XPO7_CHICK	IPI00821160.1
IPI00681387.2	IPI00819069.1
STAM2_CHICK	NP_001004409.1
Q5ZIV8_CHICK	IPI00603919.1
Q5ZMT6_CHICK	XR_026692.1
IPI00604093.1	IPI00571180.3
IPI00584954.1	NP_989903.1
IPI00586708.3	Q9W6Q6_CHICK
XR_027204.1	NP_996757.1
SPN1_CHICK	O42421_CHICK
IPI00596855.3	GTR3_CHICK
IPI00584207.3	NP_989657.1
IPI00572599.2	NP_001012967.1
LOC769161	B9VMA9_CHICK
IPI00572136.3	IPI00821254.1
IPI00593619.2	IPI00578369.2
XR_026868.1	IPI00823435.1
NP_001026290.1	IPI00589034.2
IPI00588757.3	CAC1C_CHICK
IPI00598430.2	IPI00573170.2
NP_001025780.1	IPI00684904.2
NP_001026088.1	IPI00587021.3
IPI00596524.2	IPI00598220.3
XR_027190.1	IPI00600977.2
NP_989710.1	NP_001006236.1
Q6X3Y9_CHICK	LOC776399
NP_989484.1	Q8AV27_CHICK
IPI00589837.2	IPI00600831.3
HB2L_CHICK	IPI00599050.2
IPI00819331.1	IPI00604213.3

IPI00822732.1	B5G557_CHICK
NP_990372.1	NP_001029041.1
Q9DGH8_CHICK	IPI00579185.1
NP_989902.1	O42418_CHICK
NP_989717.1	IPI00571830.2
A5HUL8_CHICK	IPI00592966.3
IPI00588912.2	Q9YGQ9_CHICK
NP_989959.1	IPI00575434.2
NP_989740.1	Q5ZKG6_CHICK
NP_001007080.1	NP_990072.1
IPI00604344.2	IPI00571078.3
IPI00578232.1	NCKX1_CHICK
RAB10_CHICK	IPI00683221.2
IPI00586006.3	IPI00578166.3
IPI00577052.2	IPI00579948.2
MIRO2_CHICK	SPX3_CHICK
IPI00572853.2	IPI00584476.2
IPI00592157.2	XR_027154.1
IPI00813592.2	IPI00819156.1
IPI00591419.2	MRP1_CHICK
NP_001025831.1	Q8AY19_CHICK
GLI3_CHICK	IPI00579342.3
IPI00585089.1	ARHG6_CHICK
XR_026953.1	Q5ZJC0_CHICK
IPI00581726.2	IPI00575349.3
IPI00579307.3	IPI00587973.2
IPI00590321.1	IPI00586607.2
IPI00822164.1	IPI00601481.3
Q5F337_CHICK	IPI00580588.2
NP_989792.1	NP_001025819.1
IPI00601852.2	IPI00596688.3
Q90YX6_CHICK	NP_001010841.1
RHOC_CHICK	IPI00586462.3
ARL8A_CHICK	IPI00582840.2
CDC42_CHICK	IPI00599886.3
IPI00819159.1	IPI00580739.3
RAB6A_CHICK	IPI00580030.3
NP_001026498.1	NP_001025536.1
NM_205017.1	Q9PT87_CHICK
IPI00810723.2	KIT_CHICK
IPI00603978.1	IPI00821165.1
IPI00577366.2	EPHA3_CHICK

IPI00580160.2	IPI00819162.1
RABL3_CHICK	Q91015_CHICK
XR_026673.1	EPHA5_CHICK
IPI00601244.1	Q8AV70_CHICK
ARF5_CHICK	NTRK2_CHICK
IPI00594309.3	PGFRA_CHICK
IPI00576555.1	IPI00814048.2
IPI00573678.2	AT2B1_CHICK
NP_001012590.1	IPI00571180.3
IPI00822572.1	Q9W6Q6_CHICK
IPI00602093.1	IPI00593562.2
XR_027123.1	IPI00587053.2
NP_001006207.1	IPI00579948.2
IPI00597909.1	AT2A2_CHICK
DICER_CHICK	IPI00597186.2
IPI00598672.2	IPI00599427.2
IPI00571045.3	CASP2_CHICK
Q5ZIT4_CHICK	NP_001038154.1
IPI00598251.3	Q90WU0_CHICK
IPI00591557.2	IPI00597741.3
NP_989911.1	Q9W6F1_CHICK
IPI00580554.2	IPI00582379.2
NP_001030000.1	IPI00574950.3
IPI00579410.4	XR_026722.1
IPI00822376.1	IPI00591557.2
NP_001026505.1	IPI00823339.1
IPI00822637.1	Q5ZI29_CHICK
IPI00575056.1	DHSA_CHICK
IPI00820185.1	NP_001026001.1
NP_001012879.1	IPI00593517.1
IPI00821103.1	IPI00682714.2
NP_989849.1	XR_027204.1
IPI00572844.3	XR_026708.1
IPI00819262.1	IPI00588757.3
NP_989848.1	IPI00580186.3
IPI00572549.1	IPI00602002.2
NP_001012577.1	IPI00577577.3
IPI00586871.1	XR_027198.1
IPI00594378.3	IPI00819020.1
SEPT2_CHICK	PA24A_CHICK
IPI00576004.3	IPI00576464.3
NP_001025825.1	NP_996864.1

NP_001026296.1	NP_001025899.1
IPI00683568.2	IPI00602611.3
IPI00578880.1	NP_001026484.1
IPI00819156.1	SPY2_CHICK
Q8AY19_CHICK	IPI00822337.1
IPI00600162.2	IPI00820913.1
IPI00814048.2	HIRA_CHICK
CAC1B_CHICK	Q6X3Y9_CHICK
AT2A2_CHICK	XR_027190.1
TRPC7	HB2L_CHICK
Q9DEX2_CHICK	IPI00822732.1
Q9YH83_CHICK	A5HUL8_CHICK
AT2B1_CHICK	MYBB_CHICK
IPI00820292.1	IPI00820062.1
NP_001004409.1	NP_989568.1
NP_996757.1	NP_001026530.1
CAC1C_CHICK	XR_027180.1
NP_989463.1	IPI00818817.1
IPI00818851.1	NP_001007947.1
IPI00587053.2	IPI00823375.1
IPI00592966.3	IPI00589002.1
IPI00680945.2	TAD2A_CHICK
O42347_CHICK	IPI00598956.2
SEM3E_CHICK	IPI00572917.2
IPI00596873.3	IPI00820922.1
SEM3C_CHICK	POLR2L
Q9DEC9_CHICK	IPI00597464.4
NP_990006.1	MYSM1_CHICK
IPI00602611.3	IPI00581796.3
NP_001032346.1	IPI00588780.3
NP_001032351.1	LOC425967
Q3L248_CHICK	IPI00820822.1
IPI00591458.2	IPI00820399.1
SEM4D_CHICK	IPI00822104.1
PITX1_CHICK	NP_990124.1
RX1_CHICK	IPI00581170.3
IPI00822726.1	NP_989755.1
NP_989667.1	IPI00818020.1
IPI00588048.1	IPI00582485.3
SPY2_CHICK	IPI00590108.3
IPI00822237.2	IPI00821485.1
IPI00604365.3	IPI00573333.2

LDB1_CHICK	IPI00598838.3
Q3L250_CHICK	IPI00811256.2
PROX1_CHICK	PAX9_CHICK
IPI00582719.3	IPI00681690.2
LDB2_CHICK	IPI00589286.3
Q8JHZ7_CHICK	IPI00571950.3
WNT9A_CHICK	IPI00575965.1
CRY2_CHICK	POTE1_CHICK
NP_989514.1	NP_989607.1
Q5ZJP8_CHICK	IPI00586551.3
CRY1_CHICK	NP_001006389.1
NP_989607.1	Q91015_CHICK
IPI00820410.1	CRIM1_CHICK
IPI00579558.3	Q5ZI90_CHICK
RAD51_CHICK	IPI00581162.3
XR_026809.1	G3P_CHICK
IPI00572701.2	NP_001025728.1
IPI00573333.2	XR_027032.1
BLM_CHICK	IPI00591196.2
IPI00818388.1	NP_001026323.1
ITA1_CHICK	IPI00821707.1
Q6V0P4_CHICK	NP_001032348.1
IPI00598938.3	IPI00592525.3
CAD13_CHICK	NP60_CHICK
ITA6_CHICK	NP_001006502.1
TSP2_CHICK	IPI00577906.1
IPI00598904.3	IPI00576005.2
NP_001025954.1	IPI00596046.3
NEL_CHICK	IPI00821264.1
IPI00594966.2	IPI00603973.4
NP_989495.1	FANCJ_CHICK
Q9YGQ6_CHICK	IPI00819732.1
Q91415_CHICK	IPI00593059.1
IPI00581215.3	IPI00579558.3
IPI00601647.3	NP_001006451.1
Q9YGR4_CHICK	IPI00573852.2
Q6V0P5_CHICK	NP_001026692.1
CO9A1_CHICK	IPI00822678.1
Q91015_CHICK	IPI00822678.1
Q804X4_CHICK	Q5ZMV6_CHICK
IPI00594139.2	NP_001026253.1
TYRO3_CHICK	NP_001026308.1

COCA1_CHICK	NP_001006550.1
NCAM1_CHICK	Q9DEI0_CHICK
IPI00588351.3	IPI00570850.2
IPI00586914.3	IPI00593118.1
IPI00820415.1	DNA2L_CHICK
NP_001008459.1	BLM_CHICK
IPI00600729.3	NP_001006456.1
A6YJX3_CHICK	DPOE2_CHICK
Q6R0I0_CHICK	NP_001006181.1
Q6R0H8_CHICK	NP_001026308.1
IPI00579481.2	PCNA_CHICK
Q9YGQ9_CHICK	NP_001026178.1
CADH6_CHICK	NP_990024.1
IPI00579675.2	IPI00821549.1
IPI00592078.2	IPI00592615.3
NP_001039292.1	XR_027212.1
IPI00588122.2	XR_026668.1
CTNA2_CHICK	IPI00822608.1
VINC_CHICK	IPI00594951.3
ITAV_CHICK	MRE11_CHICK
Q6R0I1_CHICK	NP_989616.1
Q6V0P1_CHICK	NP_989607.1
Q90796_CHICK	BLM_CHICK
CADH1_CHICK	IPI00822162.1
IPI00683898.2	IPI00821036.1
IPI00679350.2	NP_001006387.1
NP_001026448.1	IPI00596200.1
IPI00590518.2	NP_001006387.1
IPI00819700.1	XR_027012.1
IPI00820436.1	IPI00597194.3
IPI00812156.2	IPI00591892.2
MRE11_CHICK	IPI00573277.2
IPI00572549.1	NP_001026273.1
IPI00599306.1	NP_001026378.1
IPI00811977.2	IPI00604168.3
IPI00575771.3	IPI00583886.2
IPI00812061.1	IPI00588415.1
IPI00602335.3	IPI00586261.2
Q9PUI8_CHICK	NP_001026227.1
IPI00578369.2	IPI00597775.1
GBRG2_CHICK	IPI00587216.3
Q5ZIV4_CHICK	IPI00587216.3

IPI00821707.1	SYCC_CHICK
IPI00600071.3	IPI00812698.2
NP_001032348.1	NP_001026681.1
NP_001025814.1	NP_001006144.1
IPI00595677.2	IPI00592258.2
IPI00580632.3	NP_001025754.1
NP_990221.1	NP_001026618.1
IPI00598167.3	IPI00586842.1
IPI00820880.1	IF5A2_CHICK
IPI00581980.3	IPI00594164.2
IPI00819571.1	NP_001005802.1
IPI00574970.2	NP_001006298.1
XR_027198.1	IPI00580971.2
O93586_CHICK	IPI00592455.3
CATL1_CHICK	IPI00594760.1
IPI00683486.2	Q5ZIX9_CHICK
IPI00593121.2	GCSP_CHICK
Q75W80_CHICK	GCSP_CHICK
Q788Q2_CHICK	IPI00577411.1
IPI00587812.2	IPI00586982.2
XR_026893.1	IPI00579350.3
IPI00602573.3	IPI00575843.1
IPI00599900.3	NP_001025974.1
NP_001025528.1	IPI00597595.2
IPI00596075.2	IPI00584385.2
IPI00594977.2	IPI00591857.3
NP_001026507.1	SPN1_CHICK
Q9PT87_CHICK	IPI00818733.1
IPI00599675.2	IPI00595042.1
Q5ZK89_CHICK	IPI00593517.1
Q5QHS0_CHICK	IPI00585331.3
IPI00595126.3	Q5ZMD8_CHICK
IPI00597186.2	IPI00578808.3
IPI00575822.3	PHS2_CHICK
IPI00573750.2	IPI00821761.1
NP_001026683.1	HEMH_CHICK
IPI00821811.1	SODC_CHICK
IPI00818613.1	NOS2_CHICK
IPI00573332.2	IPI00822117.1
IPI00575762.3	IPI00585469.3
NP_001005802.1	IPI00811126.1
Q5ZKL3_CHICK	NP_001012917.1

IPI00580247.3	Q9IAS3_CHICK
CASP2_CHICK	IPI00597270.1
IPI00820111.1	IPI00590384.3
IPI00588768.3	IPI00583651.3
IPI00819331.1	IPI00579058.1
IPI00603980.3	IPI00572599.2
NP_001038154.1	IPI00601567.3
IPI00604346.1	IPI00683274.2
IPI00821288.1	IPI00598611.1
TRY2_CHICK	NP_001025750.1
IPI00583927.3	IPI00589610.3
CAN2_CHICK	NP_996757.1
IPI00576308.1	Q5ZJA5_CHICK
IPI00819302.1	FRIH_CHICK
IPI00603559.2	NP_990538.1
IPI00574554.2	NP_001012820.1
Q90WU0_CHICK	NP_001026306.1
IPI00583043.3	NP_001006320.1
NP_001006385.1	IPI00584954.1
O93363_CHICK	Q90765_CHICK
IPI00596756.3	BECN1_CHICK
IPI00590280.2	CALD1_CHICK
IPI00602580.4	IPI00847062.1
IPI00579987.2	NP_001026326.1
IPI00592378.3	IPI00586551.3
IPI00585115.1	IPI00820719.1
IPI00585847.3	IPI00588496.3
IPI00820386.1	IPI00585380.3
NP_990544.1	IPI00819403.1
IPI00684448.2	IPI00594950.3
NP_001019746.1	IPI00570986.2
NP_998751.1	NP_989607.1
NP_989915.1	CADH1_CHICK
IPI00581140.3	Q6V0P4_CHICK
IPI00588781.2	CAD13_CHICK
NP_989772.1	Q9DDD3_CHICK
IPI00821300.1	Q9YGQ6_CHICK
LOC423941	IPI00581215.3
IPI00572749.3	Q9YGR4_CHICK
IPI00812503.2	Q6V0P5_CHICK
IPI00819885.1	NP_990521.1
IPI00819244.1	A6YJX3_CHICK

GARL3_CHICK	IPI00680581.2
IPI00595342.3	Q6R0I0_CHICK
IPI00820946.1	Q6R0H8_CHICK
IPI00586607.2	Q9YGQ9_CHICK
IPI00679865.2	CADH6_CHICK
NP_001026442.1	Q6R0I1_CHICK
IPI00582549.4	NP_001039292.1
NF1_CHICK	Q6V0P1_CHICK
IPI00823417.1	XR_027037.1
IPI00603663.2	Q91415_CHICK
XR_027072.1	IPI00586914.3
IPI00583672.3	IPI00680879.2
IPI00588554.3	IPI00595617.2
IPI00582840.2	IPI00599567.2
IPI00600532.3	IPI00822459.1
Q5F3V4_CHICK	NP_001026242.1
NP_001026390.1	IPI00595390.2
IPI00595657.2	Q5J7V0_CHICK
NP_990221.1	IPI00594712.3
IPI00822349.1	Q9YGQ9_CHICK
NP_990816.1	XR_027090.1
IPI00596551.4	Q91415_CHICK
IPI00576418.2	IPI00586914.3
IPI00583166.3	IPI00581795.2
IPI00589384.3	SPINZ_CHICK
IPI00579342.3	IPI00682552.2
STMN2_CHICK	NP_989739.1
IPI00600071.3	IPI00598916.2
IPI00575349.3	NP_001012949.1
ARHG6_CHICK	SAT1_CHICK
IPI00596330.2	IPI00593233.3
IPI00820886.1	NP_001025728.1
IPI00587022.3	IPI00571235.3
IPI00583699.1	XR_027152.1
NP_001026363.1	IPI00596042.3
IPI00594255.2	API5_CHICK
IPI00598794.2	IPI00819675.1
IPI00577164.3	LOC776105
NP_990203.1	IPI00580338.3
IPI00577693.3	IPI00584421.3
IPI00820659.1	IPI00818371.1
IPI00602356.2	IPI00819020.1

IPI00582549.4	ACOC_CHICK
NP_001026490.1	IPI00570827.1
IPI00596688.3	NP_001026053.1
IPI00592419.3	IPI00581886.3
IPI00587034.1	IPI00584832.3
NP_001006133.1	IPI00592113.2
IPI00574944.3	IPI00814180.2
NP_001025819.1	IPI00819440.1
STMN1_CHICK	O93363_CHICK
Q98SN6_CHICK	PA24A_CHICK
IPI00821902.1	NP_001006385.1
IPI00603962.3	IPI00585847.3
IPI00592526.3	IPI00820386.1
IPI00596984.2	IPI00584061.2
NP_001026601.1	IPI00818816.1
IPI00596422.2	IPI00578405.3
NP_989962.1	NP_001006578.2
IPI00582579.3	GSTA2_CHICK
IPI00590422.3	IPI00587689.1
NP_001006390.1	IPI00594164.2
IPI00580632.3	IPI00589655.1
IPI00587190.3	IF5A2_CHICK
IPI00582840.2	IPI00586982.2
IPI00576389.4	IPI00600585.3
IPI00593292.3	NP_001001301.1
Q98SN5_CHICK	IPI00594760.1
NP_001026372.1	IPI00601144.1
IPI00581980.3	Q5ZIX9_CHICK
IPI00592402.3	IPI00819871.1
IPI00590903.4	IPI00576005.2
BRAF1_CHICK	PRPS2_CHICK
IPI00579857.1	IPI00596630.1
XR_027198.1	IPI00577839.1
Q5F3V4_CHICK	PRPS2_CHICK
IPI00580030.3	IPI00596630.1
NP_989666.1	NP_001025718.1
IPI00602518.3	IPI00602356.2
IPI00574970.2	IPI00592526.3
GLL7_CHICK	NP_989962.1
IFNA3_CHICK	IPI00593770.1
IPI00597741.3	ERP29_CHICK
IPI00592671.1	PSME3_CHICK

IPI00572674.2	IPI00598371.3
XR_027181.1	IPI00683045.1
NP_989914.1	NP_001006389.1
IPI00591919.1	IPI00682509.2
IPI00595277.3	NP_001025942.1
IPI00586396.1	IPI00571346.1
NP_989805.1	IPI00680502.3
NP_001012580.1	IPI00579585.3
XR_026841.1	IPI00819611.1
NP_001026304.1	IPI00821995.1
IPI00588800.3	IPI00580880.2
IPI00588418.3	IPI00820109.1
IPI00596433.3	NP_001026167.1
IPI00577372.3	IPI00822283.1
IPI00818548.1	NP_989816.1
XR_026754.1	IPI00599653.3
IPI00604234.3	IPI00593511.2
IPI00591077.3	IPI00590995.2
RS15_CHICK	TAF3_CHICK
IPI00574602.2	IPI00601501.3
IPI00599862.2	IPI00593935.2
SYCC_CHICK	IPI00580182.1
RL30_CHICK	IPI00594088.1
IPI00603744.2	VPP1_CHICK
RL35_CHICK	VATB_CHICK
IPI00572512.1	NP_989617.1
Q6EE61_CHICK	IPI00588938.1
IPI00589221.2	NP_001028816.1
RS27A_CHICK	IPI00590522.1
NP_001026100.1	PAFA_CHICK
RS13_CHICK	TGFB2_CHICK
IPI00598304.2	TGFB3_CHICK
RM37_CHICK	IPI00820145.1
IPI00811129.1	IPI00588858.2
IPI00586190.2	IPI00577942.3
RL37A_CHICK	IPI00589597.2
Q98TF9_CHICK	IPI00594818.3
PELO_CHICK	IPI00595603.1
IPI00572788.1	Q5F446_CHICK
RS4_CHICK	NCOA7_CHICK
IPI00679673.2	IPI00576281.1
NP_001026227.1	IPI00591205.1

RL26_CHICK	DPH2_CHICK
RL7_CHICK	LOC776686
IPI00598672.2	IPI00821813.1
IPI00580519.1	IPI00847048.1
RL39_CHICK	IPI00600705.1
NP_001006241.1	NP_001026639.1
NP_001026075.1	NP_996864.1
IPI00597958.3	Q5ZK89_CHICK
NP_001026007.1	IPI00570838.2
NP_001035503.1	GCSH_CHICK
IPI00682237.2	IPI00681843.1
IPI00819932.1	IPI00820140.1
IPI00822922.1	IPI00595373.3
IPI00592126.2	IPI00601901.2
IPI00573257.4	IPI00593376.1
KCAB1_CHICK	Q9DEC9_CHICK
IPI00588817.2	IPI00590849.2
IPI00581191.3	IPI00600757.3
IPI00574468.3	XR_027181.1
IPI00574526.2	IPI00822433.1
IPI00820101.1	IPI00574267.4
IPI00590944.2	IPI00682607.2
NP_989749.1	IPI00573965.3
LOC430136	IPI00820707.1
O42508_CHICK	RRMJ3_CHICK
IPI00589286.3	IPI00587903.2
IPI00883188.1	IPI00583911.3
IPI00571508.3	IPI00600577.2
IPI00594138.1	IPI00578132.3
Q5ZLI4_CHICK	RBG1L_CHICK
NP_989657.1	Q5ZMK6_CHICK
AT1B1_CHICK	IPI00598715.2
IPI00594219.1	IPI00600450.3
IPI00821254.1	IPI00810892.2
NP_001038116.1	NP_001026011.1
IPI00587021.3	LOC768562
LOC429723	IPI00599518.2
IPI00570900.2	IPI00579593.3
IPI00576169.3	IPI00577808.3
IPI00599050.2	IPI00586533.2
IPI00574389.1	IPI00821761.1
IPI00573547.3	NEUM_CHICK

IPI00579185.1	IPI00812943.2
XR_026889.1	IPI00589610.3
IPI00588354.1	IPI00684160.2
XR_027154.1	IPI00683898.2
IPI00600568.2	IPI00600575.3
IPI00596200.1	IPI00683580.2
IPI00589574.3	NP_001001301.1
IPI00589137.3	ARLY2_CHICK
NP_001006448.1	NP_001026227.1
IPI00820874.1	Q9PVL2_CHICK
IPI00578643.1	NP_001026290.1
IPI00684089.1	IPI00593403.3
IPI00578749.2	IPI00574302.1
IPI00595896.2	IPI00820385.1
O73841_CHICK	NP_001026484.1
IPI00820385.1	PRPS2_CHICK
IPI00577405.2	IPI00573434.1
IPI00580368.2	IPI00680633.1
Q5ZLM6_CHICK	IPI00595304.2
IPI00579063.1	NP_001034693.1
IPI00572554.1	IF5A2_CHICK
NP_001006281.1	IF5A2_CHICK
IPI00597762.1	IPI00578781.1
IPI00604003.3	IPI00582433.3
Q90750_CHICK	IPI00587336.1
NP_001008789.1	XR_026853.1
IPI00575822.3	IPI00819350.1
IPI00821288.1	IPI00582397.3
IPI00579948.2	NP_001025728.1
IPI00811057.2	IPI00597631.2
IPI00584637.3	NP_001004410.1
IPI00590401.2	NP_001006578.2
IPI00819684.1	MIRO2_CHICK
IPI00682833.1	NP_990363.1
IPI00818474.1	NP_989691.1
IPI00683486.2	IPI00597631.2
NP_001026683.1	NP_001004410.1
PRS4_CHICK	NP_001012969.1
NP_001006225.1	Q9DEC9_CHICK
NP_001006494.1	ANXA5_CHICK
NP_001026361.1	IPI00577751.1
AT2A2_CHICK	IPI00821872.1

IPI00814048.2	SMC5_CHICK
IPI00819611.1	IPI00592666.3
NP_989749.1	IPI00575200.3
IPI00821995.1	NM_001004400.2
IPI00580880.2	TBA2_CHICK
IPI00820109.1	IPI00599285.3
AT2B1_CHICK	TBA4_CHICK
NP_001026167.1	TBA1_CHICK
AT1B1_CHICK	TBB3_CHICK
NP_001038116.1	IPI00823339.1
NP_001026052.1	NP_001025772.1
IPI00587053.2	KCAB1_CHICK
IPI00593562.2	CP17A_CHICK
IPI00822283.1	IPI00819230.1
IPI00822117.1	IPI00819650.1
IPI00570706.3	IPI00587812.2
Q8AYP9_CHICK	IPI00818136.1
IPI00580338.3	IPI00596674.2
NP_989543.1	IPI00570838.2
NP_001006128.1	IPI00572932.3
KPYK_CHICK	IPI00603009.3
NP_989432.1	Q2WBI1_CHICK
LDHA_CHICK	ACD11_CHICK
IPI00574366.3	G3P_CHICK
IPI00584839.3	FRIH_CHICK
IPI00821466.1	IOD3_CHICK
IPI00592592.3	P4HA1_CHICK
NP_001034359.1	IPI00590263.3
PIGM_CHICK	IPI00823200.1
IPI00604182.2	IPI00820140.1
IPI00819794.1	Q5ZI29_CHICK
IPI00603017.2	NP_001026173.1
IPI00684277.1	IPI00593182.3
IPI00589653.3	LOC776686
IPI00593042.1	IPI00576072.2
IPI00819543.1	NP_989910.1
IPI00592918.3	IPI00588258.1
IPI00679931.2	NP_001025970.1
PRDX6_CHICK	IPI00576752.1
IPI00584442.2	IPI00818809.1
IPI00682783.2	IPI00599090.4
IPI00603388.4	IPI00680586.2

NP_001026581.1	PXDNL
NHLC2_CHICK	NP_989634.1
Q5F472_CHICK	IPI00822678.1
H11L_CHICK	PSME3_CHICK
H2B1_CHICK	NP60_CHICK
H2AY_CHICK	NP_001025801.1
H2A4_CHICK	IPI00586982.2
H2AZ_CHICK	NP_001025731.1
IPI00575038.3	NP_001001301.1
H4_CHICK	Q5ZM68_CHICK
H2B5_CHICK	IPI00819543.1
H2AJ_CHICK	IPI00575843.1
IPI00582485.3	Q804X4_CHICK
IPI00582392.1	NP_001026323.1
H32_CHICK	IPI00821374.1
IPI00593450.3	IPI00598031.3
NP_989842.1	SODC_CHICK
Q5TIL8_CHICK	NP_001006136.1
NP_001026684.1	IPI00593517.1
NP_001025989.1	XDH_CHICK
Q6ZYP0_CHICK	IPI00574953.3
IPI00820237.1	IPI00593019.3
NP_001026658.1	Q5ZLS9_CHICK
IPI00583912.1	NP_001025966.1
NP_990339.1	CP1A4_CHICK
IPI00573090.3	IPI00822448.1
Q5QQ39_CHICK	Q5ZHU7_CHICK
IPI00600935.3	AL1A1_CHICK
NP_001012590.1	GCSP_CHICK
Q9DEQ8_CHICK	Q5MQR0_CHICK
B1B565_CHICK	XR_026694.1
IPI00600071.3	IPI00595228.3
IPI00589384.3	NP_989816.1
IPI00575349.3	XR_026853.1
RHG15_CHICK	IPI00820230.1
IPI00591811.3	IPI00593755.1
IPI00578610.3	NP_990221.1
NFKB1_CHICK	LDHA_CHICK
IPI00586048.3	IPI00579957.1
IPI00821531.1	IPI00579350.3
IPI00587022.3	IPI00590366.3
IPI00593231.3	IPI00598301.2

IPI00820946.1	IPI00584645.1
UNC5C_CHICK	IPI00822433.1
IPI00573855.1	IPI00581141.3
IPI00591419.2	IPI00582397.3
IPI00822692.1	IPI00822052.1
IPI00585441.2	IPI00599576.2
IPI00578474.1	DHSA_CHICK
LOC769442	IPI00601047.3
IPI00594148.3	HPGD
IPI00598277.3	NP_001006539.1
RAN_CHICK	IPI00813416.2
NP_001026055.1	CP2H1_CHICK
KAP0_CHICK	NOS2_CHICK
IPI00822349.1	ADH1_CHICK
IPI00812213.1	FAS_CHICK
GLI3_CHICK	XR_027146.1
NP_001026442.1	Q9PVL2_CHICK
Q98946_CHICK	Q9I8T6_CHICK
IPI00571290.2	BMP4_CHICK
IPI00591456.3	NP_001026578.1
IPI00572578.2	IPI00574247.2
IPI00819040.1	PSA1_CHICK
IPI00810781.2	NP_001025961.1
IPI00583262.3	IPI00599101.1
IPI00579307.3	IPI00581182.4
Q90717_CHICK	NP_989685.1
IPI00601852.2	Q90947_CHICK
IPI00578103.2	IPI00819764.1
IPI00823338.1	Q9PSC7_CHICK
IPI00580275.3	NP_001026240.1
NP_989733.1	NP_989748.1
IPI00600847.3	IPI00812448.2
NP_001026746.1	IPI00589207.2
IPI00823417.1	NP_989453.1
IPI00582549.4	IPI00587481.4
IPI00813205.2	IPI00598546.3
NP_001026517.1	IPI00588538.3
NF1_CHICK	IPI00821671.1
IPI00595478.3	IPI00685014.2
NP_001026396.1	IPI00681062.2
NP_989717.1	IPI00587429.3
2ABD_CHICK	IPI00581980.3

TEN1_CHICK	IPI00572538.4
Q98SN6_CHICK	IPI00588638.3
IPI00575093.1	NP_001026057.1
Q9DGH8_CHICK	IPI00819991.1
IPI00598376.2	NP_001025775.1
IPI00822290.1	IPI00591902.2
IPI00820388.1	FAS_CHICK
IPI00821008.1	XR_027102.1
Q802E5_CHICK	PUR2_CHICK
IPI00820370.1	IPI00587812.2
IPI00596984.2	IPI00576564.3
NP_001026498.1	XR_027124.1
LOC429923	IPI00573009.3
IPI00583672.3	IPI00576464.3
IPI00810723.2	IPI00594760.1
IPI00585593.3	IPI00582476.2
IPI00603978.1	IPI00821761.1
IPI00580632.3	NP_001026451.1
IPI00579856.3	IPI00589694.1
NP_990816.1	IPI00570829.3
ANGPTL5	Q9I9L4_CHICK
NP_001026125.1	IPI00577509.1
NP_001025909.1	IPI00812213.1
IPI00587190.3	KAP0_CHICK
IPI00599632.1	IPI00599632.1
Q98SN5_CHICK	NP_989760.1
IPI00571872.3	XR_026654.1
NP_001004410.1	IPI00597610.2
IPI00585822.3	NP_001006181.1
IPI00587779.3	TOP2A_CHICK
BRAF1_CHICK	TOP2B_CHICK
AB1IP_CHICK	NP_001026129.1
IPI00578929.3	CI006_CHICK
IPI00576555.1	IPI00588243.2
IPI00592402.3	IPI00599886.3
IPI00580030.3	Q90716_CHICK
NP_001029041.1	Q9DEC9_CHICK
IPI00598371.3	IPI00582433.3
NP_990072.1	LOC419482
NP_989587.1	IPI00603402.4
IPI00820606.1	IPI00584919.4
IPI00584425.3	IPI00582510.3

O42347_CHICK	Q9YGQ9_CHICK
IPI00823282.1	IPI00571078.3

Supplementary table 2. Differentially methylated genes between chicken breeds.

Pairwise Comparison	Transcript ID
RJF vs CH	No
RJF vs AA	ENSGALT00000028623
	ENSGALT00000014058
	ENSGALT00000016875
	ENSGALT00000035558
RJF vs WL	ENSGALT00000036045
CH vs AA	No
CH vs WL	No
AA vs WL	ENSGALT00000030604
	ENSGALT00000021191
	ENSGALT00000028031
	ENSGALT00000040394
	ENSGALT00000040845
	ENSGALT00000010200
	ENSGALT00000005397
	ENSGALT00000038997
	ENSGALT00000038443
	ENSGALT00000031378
	ENSGALT00000039071
	ENSGALT00000037637
	ENSGALT00000037636
	ENSGALT00000035375
	ENSGALT00000036610
	ENSGALT00000036593
	ENSGALT00000040769
	ENSGALT00000000424
	ENSGALT00000031934
	ENSGALT00000026577
	ENSGALT00000018724
	ENSGALT00000008285
	ENSGALT00000006744
	ENSGALT00000014058
	ENSGALT00000032637
	ENSGALT00000037801
	ENSGALT00000029498
	ENSGALT00000016875
	ENSGALT00000020492

ENSGALT00000023582
ENSGALT00000036126
ENSGALT00000035645
ENSGALT00000035558
ENSGALT00000002697

Supplementary table 3. DEGs between different chicken breeds.

DEGs between RJF and CH		DEGs between RJF and AA	
**Ensembl Transcript ID**	**HGNC symbol**	**Ensembl Transcript ID**	**HGNC symbol**
ENSGALT00000000023		ENSGALT00000000026	AAK1
ENSGALT00000000026	AAK1	ENSGALT00000000084	
ENSGALT00000000084		ENSGALT00000000104	NRBP2
ENSGALT00000000104	NRBP2	ENSGALT00000000128	INTS4
ENSGALT00000000216	ZNF638	ENSGALT00000000136	ANTXR1
ENSGALT00000000221	CDS2	ENSGALT00000000162	ZMAT3
ENSGALT00000000238		ENSGALT00000000216	ZNF638
ENSGALT00000000243	MTCH1	ENSGALT00000000221	CDS2
ENSGALT00000000312	SP2	ENSGALT00000000227	
ENSGALT00000000385	ARID4B	ENSGALT00000000237	
ENSGALT00000000407		ENSGALT00000000238	
ENSGALT00000000417	SIK2	ENSGALT00000000296	
ENSGALT00000000422		ENSGALT00000000312	SP2
ENSGALT00000000548	LOXL2	ENSGALT00000000353	C2CD2L
ENSGALT00000000627	MRC2	ENSGALT00000000385	ARID4B
ENSGALT00000000700	KCTD20	ENSGALT00000000407	
ENSGALT00000000716		ENSGALT00000000417	SIK2
ENSGALT00000000721	STK38	ENSGALT00000000518	SLC25A37
ENSGALT00000000739	ERRFI1	ENSGALT00000000548	LOXL2
ENSGALT00000000779	PI16	ENSGALT00000000627	MRC2
ENSGALT00000000797	PER3	ENSGALT00000000716	
ENSGALT00000000806	RANBP3	ENSGALT00000000721	STK38
ENSGALT00000000842	KANSL1	ENSGALT00000000739	ERRFI1
ENSGALT00000000878		ENSGALT00000000779	PI16
ENSGALT00000000950	TMCC2	ENSGALT00000000797	PER3
ENSGALT00000000994	ELK4	ENSGALT00000000806	RANBP3
ENSGALT00000001004		ENSGALT00000000864	PPP1R15B
ENSGALT00000001018	SLC41A1	ENSGALT00000000910	CIRH1A
ENSGALT00000001027		ENSGALT00000000950	TMCC2
ENSGALT00000001029		ENSGALT00000000994	ELK4
ENSGALT00000001098	TBC1D22B	ENSGALT00000001004	

ENSGALT00000001106		ENSGALT00000001018	SLC41A1
ENSGALT00000001189	SRGAP2	ENSGALT00000001027	
ENSGALT00000001203	MAPK14	ENSGALT00000001029	
ENSGALT00000001233	KDM1A	ENSGALT00000001093	CAPN5
ENSGALT00000001297	MAPKAPK2	ENSGALT00000001106	
ENSGALT00000001392	WDTC1	ENSGALT00000001149	
ENSGALT00000001407	TEAD3	ENSGALT00000001193	
ENSGALT00000001409	TMEM222	ENSGALT00000001199	
ENSGALT00000001481		ENSGALT00000001203	MAPK14
ENSGALT00000001489	MMP15	ENSGALT00000001233	KDM1A
ENSGALT00000001507		ENSGALT00000001289	PHLPP2
ENSGALT00000001527	SCAMP4	ENSGALT00000001297	MAPKAPK2
ENSGALT00000001535	SCAI	ENSGALT00000001398	
ENSGALT00000001662	MIER2	ENSGALT00000001401	
ENSGALT00000001752		ENSGALT00000001409	TMEM222
ENSGALT00000001766		ENSGALT00000001481	
ENSGALT00000001774	SEMA6B	ENSGALT00000001514	GOLGA1
ENSGALT00000001776		ENSGALT00000001527	SCAMP4
ENSGALT00000001786	CD34	ENSGALT00000001603	ELN
ENSGALT00000001814		ENSGALT00000001666	
ENSGALT00000001841	MSL2	ENSGALT00000001706	WBSCR17
ENSGALT00000001850	PIGU	ENSGALT00000001720	ZNF346
ENSGALT00000001925	GTF2I	ENSGALT00000001776	
ENSGALT00000001983		ENSGALT00000001786	CD34
ENSGALT00000001988		ENSGALT00000001790	KCNJ5
ENSGALT00000002003	UBE2Z	ENSGALT00000001801	DHX8
ENSGALT00000002011	CAMK1G	ENSGALT00000001841	MSL2
ENSGALT00000002047	ZBTB44	ENSGALT00000001850	PIGU
ENSGALT00000002082		ENSGALT00000001852	HERPUD1
ENSGALT00000002090	ADAMTS8	ENSGALT00000001897	
ENSGALT00000002140	ADAMTS15	ENSGALT00000001916	CLIC4
ENSGALT00000002157	ADRA1B	ENSGALT00000001925	GTF2I
ENSGALT00000002165		ENSGALT00000001947	HOXB9
ENSGALT00000002191	RANBP10	ENSGALT00000001984	
ENSGALT00000002223	ANKFY1	ENSGALT00000001988	
ENSGALT00000002306	FHL3	ENSGALT00000002047	ZBTB44
ENSGALT00000002314	PHF20	ENSGALT00000002068	MORN5
ENSGALT00000002321	VWA1	ENSGALT00000002090	ADAMTS8
ENSGALT00000002335		ENSGALT00000002121	
ENSGALT00000002495	DUSP26	ENSGALT00000002140	ADAMTS15
ENSGALT00000002498	EDC4	ENSGALT00000002165	
ENSGALT00000002567		ENSGALT00000002191	RANBP10

ENSGALT00000002623	MED1	ENSGALT00000002218	PDIK1L
ENSGALT00000002654	DGKD	ENSGALT00000002223	ANKFY1
ENSGALT00000002657	CDK12	ENSGALT00000002230	JAM3
ENSGALT00000002661	LRIG2	ENSGALT00000002268	PPM1F
ENSGALT00000002706		ENSGALT00000002291	
ENSGALT00000002733	MFSD11	ENSGALT00000002299	CNTRL
ENSGALT00000002757	ADPGK	ENSGALT00000002314	PHF20
ENSGALT00000002971	DNAJB5	ENSGALT00000002321	VWA1
ENSGALT00000003000	SLC25A24	ENSGALT00000002333	STAB1
ENSGALT00000003002	HIPK1	ENSGALT00000002335	
ENSGALT00000003005	SPDL1	ENSGALT00000002409	
ENSGALT00000003045	INPP5E	ENSGALT00000002416	
ENSGALT00000003064	PRPF38B	ENSGALT00000002422	MLLT1
ENSGALT00000003093		ENSGALT00000002469	OGFOD3
ENSGALT00000003103	LIMS2	ENSGALT00000002498	EDC4
ENSGALT00000003133	MMRN2	ENSGALT00000002508	
ENSGALT00000003142	CAPN15	ENSGALT00000002514	
ENSGALT00000003148	COMT	ENSGALT00000002516	
ENSGALT00000003180		ENSGALT00000002609	
ENSGALT00000003276	PFKFB4	ENSGALT00000002623	MED1
ENSGALT00000003315	PLEKHN1	ENSGALT00000002654	DGKD
ENSGALT00000003339	PGAM5	ENSGALT00000002657	CDK12
ENSGALT00000003495	RERE	ENSGALT00000002661	LRIG2
ENSGALT00000003506	C3orf18	ENSGALT00000002706	
ENSGALT00000003543		ENSGALT00000002716	ACAP3
ENSGALT00000003559	DCUN1D3	ENSGALT00000002729	CEP250
ENSGALT00000003570		ENSGALT00000002733	MFSD11
ENSGALT00000003571	UNK	ENSGALT00000002757	ADPGK
ENSGALT00000003716	CMTR2	ENSGALT00000002758	HECTD2
ENSGALT00000003726	SEC16A	ENSGALT00000002781	IFT74
ENSGALT00000003737		ENSGALT00000002784	
ENSGALT00000003784	ULK1	ENSGALT00000002785	IWS1
ENSGALT00000003831		ENSGALT00000003000	SLC25A24
ENSGALT00000003844	FAM69B	ENSGALT00000003002	HIPK1
ENSGALT00000003846	BDP1	ENSGALT00000003005	SPDL1
ENSGALT00000003980	TMEM201	ENSGALT00000003038	
ENSGALT00000003987		ENSGALT00000003045	INPP5E
ENSGALT00000004014	CSGALNACT2	ENSGALT00000003064	PRPF38B
ENSGALT00000004041	GPR133	ENSGALT00000003067	GNL2
ENSGALT00000004086	MSS51	ENSGALT00000003093	
ENSGALT00000004111		ENSGALT00000003103	LIMS2

ENSGALT00000004115		ENSGALT00000003133	MMRN2
ENSGALT00000004144	TMEM248	ENSGALT00000003142	CAPN15
ENSGALT00000004204		ENSGALT00000003145	ZC3H12A
ENSGALT00000004213	GRPEL2	ENSGALT00000003148	COMT
ENSGALT00000004255		ENSGALT00000003157	AHCY
ENSGALT00000004258	ATP8B2	ENSGALT00000003180	
ENSGALT00000004357		ENSGALT00000003192	
ENSGALT00000004394	PITPNA	ENSGALT00000003246	MMP19
ENSGALT00000004469	SH3PXD2B	ENSGALT00000003296	TSTD2
ENSGALT00000004481	GRM4	ENSGALT00000003315	PLEKHN1
ENSGALT00000004554	CASZ1	ENSGALT00000003339	PGAM5
ENSGALT00000004596		ENSGALT00000003364	CAMSAP2
ENSGALT00000004598		ENSGALT00000003449	
ENSGALT00000004650	PRPF8	ENSGALT00000003506	C3orf18
ENSGALT00000004669		ENSGALT00000003529	AGO1
ENSGALT00000004844	EZH1	ENSGALT00000003543	
ENSGALT00000004906	AMFR	ENSGALT00000003570	
ENSGALT00000004933	NCOR2	ENSGALT00000003612	TMEM132E
ENSGALT00000005129	RAD54L2	ENSGALT00000003644	SORD
ENSGALT00000005131	LSM1	ENSGALT00000003716	CMTR2
ENSGALT00000005219	ADAMTS12	ENSGALT00000003726	SEC16A
ENSGALT00000005236	CRTC1	ENSGALT00000003831	
ENSGALT00000005327	KAT2A	ENSGALT00000003844	FAM69B
ENSGALT00000005338		ENSGALT00000003846	BDP1
ENSGALT00000005386		ENSGALT00000003906	B3GALT2
ENSGALT00000005407	ZBTB8A	ENSGALT00000003918	SFSWAP
ENSGALT00000005424		ENSGALT00000003944	
ENSGALT00000005581	ARRDC2	ENSGALT00000003954	COL5A2
ENSGALT00000005592	KLHL11	ENSGALT00000003980	TMEM201
ENSGALT00000005608		ENSGALT00000003987	
ENSGALT00000005617	PRELP	ENSGALT00000004032	
ENSGALT00000005643		ENSGALT00000004041	GPR133
ENSGALT00000005655		ENSGALT00000004086	MSS51
ENSGALT00000005662	RNF19B	ENSGALT00000004113	U2SURP
ENSGALT00000005664		ENSGALT00000004144	TMEM248
ENSGALT00000005721	DARS2	ENSGALT00000004183	
ENSGALT00000005731		ENSGALT00000004187	
ENSGALT00000005844	PAPSS2	ENSGALT00000004201	OLFML2B
ENSGALT00000005921	UCK1	ENSGALT00000004204	
ENSGALT00000005938	CPAMD8	ENSGALT00000004213	GRPEL2
ENSGALT00000005939	VPS37B	ENSGALT00000004246	FAM149B1
ENSGALT00000005982	SIN3B	ENSGALT00000004255	

ENSGALT00000005992	PRRC2B	ENSGALT00000004258	ATP8B2
ENSGALT00000006018	SLC35E1	ENSGALT00000004357	
ENSGALT00000006028	PABPC4	ENSGALT00000004469	SH3PXD2B
ENSGALT00000006051	NT5C1A	ENSGALT00000004489	NEURL1B
ENSGALT00000006133		ENSGALT00000004494	
ENSGALT00000006155		ENSGALT00000004499	SARDH
ENSGALT00000006164		ENSGALT00000004517	ERGIC1
ENSGALT00000006179	MTMR10	ENSGALT00000004533	ZBTB38
ENSGALT00000006182	SGCD	ENSGALT00000004538	
ENSGALT00000006265		ENSGALT00000004555	
ENSGALT00000006362		ENSGALT00000004573	ALPK2
ENSGALT00000006375		ENSGALT00000004598	
ENSGALT00000006417	PHF12	ENSGALT00000004728	SERPINF2
ENSGALT00000006422	GALNT10	ENSGALT00000004808	SRM
ENSGALT00000006478	TET1	ENSGALT00000004815	
ENSGALT00000006504	FNBP1	ENSGALT00000004844	EZH1
ENSGALT00000006533	CCAR1	ENSGALT00000004876	
ENSGALT00000006584		ENSGALT00000005064	TRADD
ENSGALT00000006652	NSRP1	ENSGALT00000005088	TSPO2
ENSGALT00000006657		ENSGALT00000005135	
ENSGALT00000006663	SLTM	ENSGALT00000005192	COMP
ENSGALT00000006691		ENSGALT00000005219	ADAMTS12
ENSGALT00000006699	ABL2	ENSGALT00000005224	
ENSGALT00000006741	TRAF3IP1	ENSGALT00000005236	CRTC1
ENSGALT00000006768	SNX8	ENSGALT00000005261	TBC1D2B
ENSGALT00000006838	CPD	ENSGALT00000005262	OSGIN1
ENSGALT00000006896		ENSGALT00000005305	RAI14
ENSGALT00000006907	ABCA5	ENSGALT00000005327	KAT2A
ENSGALT00000006909		ENSGALT00000005338	
ENSGALT00000006945	CCDC69	ENSGALT00000005348	LSM4
ENSGALT00000006957	MCM3AP	ENSGALT00000005353	DNAJC21
ENSGALT00000006970		ENSGALT00000005373	
ENSGALT00000007036	ASCC1	ENSGALT00000005381	ZNF385C
ENSGALT00000007195	TRPV2	ENSGALT00000005391	SOGA1
ENSGALT00000007219	DCTN4	ENSGALT00000005397	RALGDS
ENSGALT00000007279	LRRC8A	ENSGALT00000005424	
ENSGALT00000007292	NPLOC4	ENSGALT00000005561	AGPAT6
ENSGALT00000007303	CLCN6	ENSGALT00000005592	KLHL11
ENSGALT00000007311		ENSGALT00000005608	
ENSGALT00000007316	ZBTB37	ENSGALT00000005617	PRELP
ENSGALT00000007398		ENSGALT00000005643	
ENSGALT00000007437	SMG7	ENSGALT00000005664	

ENSGALT00000007475	NLE1	ENSGALT00000005683	
ENSGALT00000007675	EDEM3	ENSGALT00000005708	
ENSGALT00000007728	APLNR	ENSGALT00000005731	
ENSGALT00000007743	ZMIZ1	ENSGALT00000005824	PRKAR1B
ENSGALT00000007760	FICD	ENSGALT00000005832	
ENSGALT00000007838		ENSGALT00000005902	CHD9
ENSGALT00000007851		ENSGALT00000005921	UCK1
ENSGALT00000007950		ENSGALT00000005924	
ENSGALT00000008038		ENSGALT00000005938	CPAMD8
ENSGALT00000008172	SLC25A25	ENSGALT00000005939	VPS37B
ENSGALT00000008174	B9D1	ENSGALT00000005940	
ENSGALT00000008181	GDPD1	ENSGALT00000005967	
ENSGALT00000008222	FBLN2	ENSGALT00000005982	SIN3B
ENSGALT00000008232	LSM14B	ENSGALT00000005992	PRRC2B
ENSGALT00000008249	KCTD10	ENSGALT00000006018	SLC35E1
ENSGALT00000008264	PDP2	ENSGALT00000006033	DAB2
ENSGALT00000008397	MYOZ1	ENSGALT00000006051	NT5C1A
ENSGALT00000008452		ENSGALT00000006070	NUP214
ENSGALT00000008454	SEC22C	ENSGALT00000006125	PAPD5
ENSGALT00000008481	TRMT13	ENSGALT00000006133	
ENSGALT00000008492	BCAS3	ENSGALT00000006164	
ENSGALT00000008503		ENSGALT00000006179	MTMR10
ENSGALT00000008587	ZBTB5	ENSGALT00000006182	SGCD
ENSGALT00000008746	CCDC66	ENSGALT00000006206	MPHOSPH10
ENSGALT00000008792		ENSGALT00000006228	CAP1
ENSGALT00000008794		ENSGALT00000006230	EPS15L1
ENSGALT00000008940	NOLC1	ENSGALT00000006250	
ENSGALT00000009006		ENSGALT00000006252	MYPN
ENSGALT00000009044	GBF1	ENSGALT00000006317	
ENSGALT00000009068		ENSGALT00000006358	RUFY2
ENSGALT00000009079	FOXO4	ENSGALT00000006362	
ENSGALT00000009084		ENSGALT00000006374	LARP1
ENSGALT00000009103	TTC28	ENSGALT00000006422	GALNT10
ENSGALT00000009141	HIF1AN	ENSGALT00000006424	
ENSGALT00000009190	CSF1R	ENSGALT00000006478	TET1
ENSGALT00000009240		ENSGALT00000006533	CCAR1
ENSGALT00000009267	HMGXB3	ENSGALT00000006540	
ENSGALT00000009269		ENSGALT00000006583	SH3BP4
ENSGALT00000009283		ENSGALT00000006584	
ENSGALT00000009328	SEPT2	ENSGALT00000006652	NSRP1
ENSGALT00000009350	FAM219A	ENSGALT00000006663	SLTM
ENSGALT00000009355		ENSGALT00000006691	

ENSGALT00000009408		ENSGALT00000006699	ABL2
ENSGALT00000009410	TBC1D9B	ENSGALT00000006741	TRAF3IP1
ENSGALT00000009416	ALOX5	ENSGALT00000006768	SNX8
ENSGALT00000009484	USP8	ENSGALT00000006793	ASB1
ENSGALT00000009494	PCMTD2	ENSGALT00000006838	CPD
ENSGALT00000009591	SEC61A1	ENSGALT00000006867	AGGF1
ENSGALT00000009600	RIC3	ENSGALT00000006945	CCDC69
ENSGALT00000009602	KBTBD12	ENSGALT00000006957	MCM3AP
ENSGALT00000009653	ADAMTS2	ENSGALT00000006978	
ENSGALT00000009656	PRPF6	ENSGALT00000007014	
ENSGALT00000009741	FAM64A	ENSGALT00000007036	ASCC1
ENSGALT00000009806		ENSGALT00000007061	LHFPL2
ENSGALT00000009845	ZNF644	ENSGALT00000007150	FAM73B
ENSGALT00000009867		ENSGALT00000007193	PARP3
ENSGALT00000009889		ENSGALT00000007219	DCTN4
ENSGALT00000009913	GGA2	ENSGALT00000007279	LRRC8A
ENSGALT00000009976	SLC9A6	ENSGALT00000007290	NDST1
ENSGALT00000009988	FHL1	ENSGALT00000007292	NPLOC4
ENSGALT00000010006	TBC1D20	ENSGALT00000007303	CLCN6
ENSGALT00000010117	KIRREL	ENSGALT00000007316	ZBTB37
ENSGALT00000010138		ENSGALT00000007345	
ENSGALT00000010215		ENSGALT00000007398	
ENSGALT00000010255		ENSGALT00000007431	ZDHHC12
ENSGALT00000010304	LIPA	ENSGALT00000007437	SMG7
ENSGALT00000010311		ENSGALT00000007475	NLE1
ENSGALT00000010312	UBXN7	ENSGALT00000007484	PDAP1
ENSGALT00000010319	CRKL	ENSGALT00000007499	CSE1L
ENSGALT00000010339	DDX46	ENSGALT00000007566	
ENSGALT00000010372	PAK2	ENSGALT00000007571	NOL8
ENSGALT00000010426		ENSGALT00000007588	CEP89
ENSGALT00000010518	CLMP	ENSGALT00000007592	DHRS7B
ENSGALT00000010594	MCF2	ENSGALT00000007608	C1QA
ENSGALT00000010602	UBASH3B	ENSGALT00000007637	
ENSGALT00000010619		ENSGALT00000007643	TNFAIP8L3
ENSGALT00000010635	PLAGL2	ENSGALT00000007656	MPRIP
ENSGALT00000010676		ENSGALT00000007669	EPB42
ENSGALT00000010751	DUSP8	ENSGALT00000007675	EDEM3
ENSGALT00000010812	ABHD2	ENSGALT00000007729	NT5M
ENSGALT00000010834		ENSGALT00000007734	SEMA6D
ENSGALT00000010909	DET1	ENSGALT00000007743	ZMIZ1
ENSGALT00000010917		ENSGALT00000007760	FICD
ENSGALT00000010935		ENSGALT00000007838	

ENSGALT00000010949		ENSGALT00000007851	
ENSGALT00000010963	KLHDC8B	ENSGALT00000007937	GLA
ENSGALT00000010978	CCDC71	ENSGALT00000007950	
ENSGALT00000011032	USP19	ENSGALT00000007962	SLC27A4
ENSGALT00000011062	TSPAN4	ENSGALT00000007980	ALKBH5
ENSGALT00000011145	SLC38A10	ENSGALT00000007982	
ENSGALT00000011206	BAIAP2	ENSGALT00000007990	CERCAM
ENSGALT00000011255		ENSGALT00000008018	
ENSGALT00000011260	CHD2	ENSGALT00000008038	
ENSGALT00000011440		ENSGALT00000008115	PPM1E
ENSGALT00000011465	CCNY	ENSGALT00000008172	SLC25A25
ENSGALT00000011484	LYSMD4	ENSGALT00000008174	B9D1
ENSGALT00000011502	MLEC	ENSGALT00000008185	TPR
ENSGALT00000011547		ENSGALT00000008219	SWT1
ENSGALT00000011568	RNF10	ENSGALT00000008222	FBLN2
ENSGALT00000011657		ENSGALT00000008243	
ENSGALT00000011691	HGS	ENSGALT00000008249	KCTD10
ENSGALT00000011727		ENSGALT00000008264	PDP2
ENSGALT00000011730		ENSGALT00000008293	
ENSGALT00000011799		ENSGALT00000008325	SEC24C
ENSGALT00000011867	ABAT	ENSGALT00000008407	USP54
ENSGALT00000011871		ENSGALT00000008474	LRRC39
ENSGALT00000011897	MAP3K8	ENSGALT00000008481	TRMT13
ENSGALT00000011900	CILP	ENSGALT00000008490	PPFIBP2
ENSGALT00000011962		ENSGALT00000008492	BCAS3
ENSGALT00000011996		ENSGALT00000008503	
ENSGALT00000012011	PPRC1	ENSGALT00000008519	SRFBP1
ENSGALT00000012079		ENSGALT00000008552	SNCAIP
ENSGALT00000012083	PCYT1A	ENSGALT00000008553	
ENSGALT00000012148		ENSGALT00000008587	ZBTB5
ENSGALT00000012192	ANKRD2	ENSGALT00000008738	
ENSGALT00000012431		ENSGALT00000008746	CCDC66
ENSGALT00000012457	BCL9L	ENSGALT00000008792	
ENSGALT00000012479		ENSGALT00000008794	
ENSGALT00000012623		ENSGALT00000008805	
ENSGALT00000012655		ENSGALT00000008859	DYNLL2
ENSGALT00000012737	ATRX	ENSGALT00000008940	NOLC1
ENSGALT00000012797		ENSGALT00000008944	AKAP1
ENSGALT00000012884		ENSGALT00000009029	
ENSGALT00000012901		ENSGALT00000009037	MRVI1
ENSGALT00000012929	AHCYL2	ENSGALT00000009044	GBF1
ENSGALT00000012936	ALG9	ENSGALT00000009068	

ENSGALT00000012996	CAPN6	ENSGALT00000009079	FOXO4
ENSGALT00000013006		ENSGALT00000009090	
ENSGALT00000013018	KIAA0195	ENSGALT00000009103	TTC28
ENSGALT00000013063	MTMR3	ENSGALT00000009141	HIF1AN
ENSGALT00000013071	B4GALT5	ENSGALT00000009190	CSF1R
ENSGALT00000013077	PAK3	ENSGALT00000009192	PDGFRB
ENSGALT00000013128		ENSGALT00000009240	
ENSGALT00000013157	CCDC88A	ENSGALT00000009266	
ENSGALT00000013221	COL4A5	ENSGALT00000009267	HMGXB3
ENSGALT00000013245	SRPK2	ENSGALT00000009269	
ENSGALT00000013265		ENSGALT00000009283	
ENSGALT00000013322		ENSGALT00000009327	KREMEN1
ENSGALT00000013428		ENSGALT00000009328	SEPT2
ENSGALT00000013447	COL4A6	ENSGALT00000009335	
ENSGALT00000013512		ENSGALT00000009337	BCAR3
ENSGALT00000013524		ENSGALT00000009355	
ENSGALT00000013539	SLK	ENSGALT00000009407	CLUH
ENSGALT00000013587	ATG13	ENSGALT00000009408	
ENSGALT00000013683	ANKRD12	ENSGALT00000009410	TBC1D9B
ENSGALT00000013704	DVL3	ENSGALT00000009467	
ENSGALT00000013753		ENSGALT00000009484	USP8
ENSGALT00000013794	SLC35B4	ENSGALT00000009494	PCMTD2
ENSGALT00000013841		ENSGALT00000009591	SEC61A1
ENSGALT00000013858	THOC2	ENSGALT00000009602	KBTBD12
ENSGALT00000013962		ENSGALT00000009612	KIAA1107
ENSGALT00000013983	GPR1	ENSGALT00000009653	ADAMTS2
ENSGALT00000014012	TMEM255A	ENSGALT00000009656	PRPF6
ENSGALT00000014023		ENSGALT00000009723	
ENSGALT00000014035		ENSGALT00000009734	
ENSGALT00000014106		ENSGALT00000009741	FAM64A
ENSGALT00000014151		ENSGALT00000009742	
ENSGALT00000014170		ENSGALT00000009845	ZNF644
ENSGALT00000014182	ABI2	ENSGALT00000009846	ZNF644
ENSGALT00000014184	FLRT3	ENSGALT00000009861	FAM122B
ENSGALT00000014239	DPH7	ENSGALT00000009864	
ENSGALT00000014277	CTBS	ENSGALT00000009867	
ENSGALT00000014329	LRRN4	ENSGALT00000009909	DIP2B
ENSGALT00000014340	IQUB	ENSGALT00000009913	GGA2
ENSGALT00000014420	PLCB1	ENSGALT00000009976	SLC9A6
ENSGALT00000014545	NEXN	ENSGALT00000009993	
ENSGALT00000014566	C10orf118	ENSGALT00000010006	TBC1D20
ENSGALT00000014643		ENSGALT00000010046	ANKRD11

ENSGALT00000014661	SMG5	ENSGALT00000010117	KIRREL
ENSGALT00000014665	TRAF2	ENSGALT00000010129	
ENSGALT00000014710	CLDN12	ENSGALT00000010215	
ENSGALT00000014731		ENSGALT00000010255	
ENSGALT00000014752		ENSGALT00000010272	
ENSGALT00000014767	CD99L2	ENSGALT00000010280	
ENSGALT00000014770	GALNT14	ENSGALT00000010304	LIPA
ENSGALT00000014774	MAN1B1	ENSGALT00000010311	
ENSGALT00000014779	FAM160B1	ENSGALT00000010312	UBXN7
ENSGALT00000014827		ENSGALT00000010319	CRKL
ENSGALT00000014832		ENSGALT00000010339	DDX46
ENSGALT00000015009		ENSGALT00000010372	PAK2
ENSGALT00000015084		ENSGALT00000010376	
ENSGALT00000015117		ENSGALT00000010411	
ENSGALT00000015176		ENSGALT00000010484	RPP30
ENSGALT00000015190		ENSGALT00000010518	CLMP
ENSGALT00000015206		ENSGALT00000010562	TBC1D12
ENSGALT00000015328	MYNN	ENSGALT00000010596	
ENSGALT00000015350	KIAA0247	ENSGALT00000010602	UBASH3B
ENSGALT00000015385		ENSGALT00000010607	TM9SF4
ENSGALT00000015414		ENSGALT00000010635	PLAGL2
ENSGALT00000015427		ENSGALT00000010641	
ENSGALT00000015464	TMA16	ENSGALT00000010650	ZMYND11
ENSGALT00000015642		ENSGALT00000010676	
ENSGALT00000015678		ENSGALT00000010684	FBXO3
ENSGALT00000015681	KCTD12	ENSGALT00000010687	FRA10AC1
ENSGALT00000015751		ENSGALT00000010760	DIP2C
ENSGALT00000015787		ENSGALT00000010812	ABHD2
ENSGALT00000015887		ENSGALT00000010817	UPF2
ENSGALT00000015940		ENSGALT00000010834	
ENSGALT00000015941	SETD7	ENSGALT00000010845	
ENSGALT00000015973	CCDC173	ENSGALT00000010875	
ENSGALT00000015996	PPIG	ENSGALT00000010889	
ENSGALT00000015998	NKTR	ENSGALT00000010901	PVRL1
ENSGALT00000016025		ENSGALT00000010909	DET1
ENSGALT00000016037		ENSGALT00000010917	
ENSGALT00000016039	RNF150	ENSGALT00000010935	
ENSGALT00000016236	ARHGAP5	ENSGALT00000010949	
ENSGALT00000016289	BAZ1A	ENSGALT00000010978	CCDC71
ENSGALT00000016303		ENSGALT00000011032	USP19
ENSGALT00000016376	KDM4A	ENSGALT00000011059	
ENSGALT00000016432	DMAP1	ENSGALT00000011145	SLC38A10

ENSGALT00000016441		ENSGALT00000011162	ITIH5
ENSGALT00000016446	SCLT1	ENSGALT00000011184	
ENSGALT00000016472	PLK3	ENSGALT00000011226	
ENSGALT00000016480	ZFAND3	ENSGALT00000011260	CHD2
ENSGALT00000016488		ENSGALT00000011292	CDK5RAP2
ENSGALT00000016531		ENSGALT00000011460	ATP13A3
ENSGALT00000016540	SLC35B2	ENSGALT00000011465	CCNY
ENSGALT00000016569	FBXO33	ENSGALT00000011502	MLEC
ENSGALT00000016584		ENSGALT00000011547	
ENSGALT00000016627	TOE1	ENSGALT00000011621	
ENSGALT00000016690	LTBP2	ENSGALT00000011657	
ENSGALT00000016725	TMEM63B	ENSGALT00000011691	HGS
ENSGALT00000016788	MAST2	ENSGALT00000011713	
ENSGALT00000016792	NEK9	ENSGALT00000011730	
ENSGALT00000016835		ENSGALT00000011752	
ENSGALT00000016854	POMGNT1	ENSGALT00000011799	
ENSGALT00000016864		ENSGALT00000011867	ABAT
ENSGALT00000016897	FAM13A	ENSGALT00000011871	
ENSGALT00000016916	TJAP1	ENSGALT00000011900	CILP
ENSGALT00000016934		ENSGALT00000011913	RBFOX1
ENSGALT00000016991	CP	ENSGALT00000011962	
ENSGALT00000017142		ENSGALT00000012038	IFT46
ENSGALT00000017215	STON2	ENSGALT00000012079	
ENSGALT00000017430	SDCCAG8	ENSGALT00000012083	PCYT1A
ENSGALT00000017485		ENSGALT00000012140	
ENSGALT00000017502	TRIP11	ENSGALT00000012148	
ENSGALT00000017560		ENSGALT00000012192	ANKRD2
ENSGALT00000017630	BTBD7	ENSGALT00000012207	
ENSGALT00000017703	ANTXR2	ENSGALT00000012208	TPCN2
ENSGALT00000017844		ENSGALT00000012273	C7orf26
ENSGALT00000017890	NID1	ENSGALT00000012281	LRCH3
ENSGALT00000017959	CACHD1	ENSGALT00000012479	
ENSGALT00000017964	RAVER2	ENSGALT00000012554	CASS4
ENSGALT00000017973	AK4	ENSGALT00000012618	
ENSGALT00000017992	AFF1	ENSGALT00000012623	
ENSGALT00000018051		ENSGALT00000012655	
ENSGALT00000018061	CCSAP	ENSGALT00000012660	GXYLT2
ENSGALT00000018137		ENSGALT00000012664	VASN
ENSGALT00000018147	LIN54	ENSGALT00000012737	ATRX
ENSGALT00000018212	WIPF3	ENSGALT00000012759	
ENSGALT00000018221	CEP290	ENSGALT00000012805	PAMR1
ENSGALT00000018228	FAM120B	ENSGALT00000012808	

ENSGALT00000018324	GALNT15	ENSGALT00000012856	C15orf61
ENSGALT00000018386		ENSGALT00000012861	PRR5L
ENSGALT00000018392		ENSGALT00000012901	
ENSGALT00000018413		ENSGALT00000012936	ALG9
ENSGALT00000018542	TTLL4	ENSGALT00000012996	CAPN6
ENSGALT00000018652		ENSGALT00000013011	EXT2
ENSGALT00000018657		ENSGALT00000013018	KIAA0195
ENSGALT00000018669		ENSGALT00000013037	TMEM209
ENSGALT00000018681	CCNI	ENSGALT00000013051	CORO2B
ENSGALT00000018727	CMTM6	ENSGALT00000013071	B4GALT5
ENSGALT00000018770	ANO10	ENSGALT00000013107	AMMECR1
ENSGALT00000018812	EAF1	ENSGALT00000013128	
ENSGALT00000019023	ANKRD17	ENSGALT00000013157	CCDC88A
ENSGALT00000019060		ENSGALT00000013221	COL4A5
ENSGALT00000019108		ENSGALT00000013227	UACA
ENSGALT00000019136		ENSGALT00000013245	SRPK2
ENSGALT00000019144		ENSGALT00000013281	
ENSGALT00000019223	PDE4DIP	ENSGALT00000013284	FAM208B
ENSGALT00000019230	APOLD1	ENSGALT00000013336	
ENSGALT00000019301	PLBD1	ENSGALT00000013374	ANXA4
ENSGALT00000019340		ENSGALT00000013401	
ENSGALT00000019343	C7orf55	ENSGALT00000013428	
ENSGALT00000019409	SLC38A6	ENSGALT00000013447	COL4A6
ENSGALT00000019464	DESI1	ENSGALT00000013464	TMED3
ENSGALT00000019482	PMM1	ENSGALT00000013519	OBFC1
ENSGALT00000019514	ZC3H7B	ENSGALT00000013522	
ENSGALT00000019566	SYNPO2	ENSGALT00000013539	SLK
ENSGALT00000019581	SEC24D	ENSGALT00000013555	RSBN1L
ENSGALT00000019679	LARP7	ENSGALT00000013567	
ENSGALT00000019698	OSBPL11	ENSGALT00000013568	MAN2A2
ENSGALT00000019783	KIAA0895	ENSGALT00000013587	ATG13
ENSGALT00000019854		ENSGALT00000013608	TPCN1
ENSGALT00000019895	PLA2G12A	ENSGALT00000013664	PPIP5K1
ENSGALT00000019967	PPP1R17	ENSGALT00000013673	CREB3L1
ENSGALT00000020072	L2HGDH	ENSGALT00000013679	MPP4
ENSGALT00000020115	MANBA	ENSGALT00000013683	ANKRD12
ENSGALT00000020165	SASH1	ENSGALT00000013704	DVL3
ENSGALT00000020337	SBF1	ENSGALT00000013753	
ENSGALT00000020354	MIS18BP1	ENSGALT00000013794	SLC35B4
ENSGALT00000020391		ENSGALT00000013808	PLXND1
ENSGALT00000020402	KLHL28	ENSGALT00000013813	
ENSGALT00000020442	BRD9	ENSGALT00000013841	

ENSGALT00000020444	TPPP	ENSGALT00000013844	
ENSGALT00000020552	GKAP1	ENSGALT00000013858	THOC2
ENSGALT00000020591		ENSGALT00000013896	
ENSGALT00000020595	C9orf3	ENSGALT00000013924	
ENSGALT00000020625		ENSGALT00000013962	
ENSGALT00000020628	MTERFD3	ENSGALT00000013979	
ENSGALT00000020679	NRSN1	ENSGALT00000013983	GPR1
ENSGALT00000020743		ENSGALT00000014023	
ENSGALT00000020783	NT5DC3	ENSGALT00000014035	
ENSGALT00000021086		ENSGALT00000014115	
ENSGALT00000021202	B4GALNT3	ENSGALT00000014117	
ENSGALT00000021213	ERC1	ENSGALT00000014170	
ENSGALT00000021258	MARCH6	ENSGALT00000014182	ABI2
ENSGALT00000021282	BCL2L13	ENSGALT00000014184	FLRT3
ENSGALT00000021403	RERGL	ENSGALT00000014195	KIF16B
ENSGALT00000021448	TMEM245	ENSGALT00000014214	PCSK2
ENSGALT00000021521	BCAT1	ENSGALT00000014239	DPH7
ENSGALT00000021578	SLC39A6	ENSGALT00000014244	
ENSGALT00000021611		ENSGALT00000014256	FAM171A1
ENSGALT00000021647	LOXL3	ENSGALT00000014329	LRRN4
ENSGALT00000021658	ARHGEF11	ENSGALT00000014381	TTLL7
ENSGALT00000021722		ENSGALT00000014420	PLCB1
ENSGALT00000021812		ENSGALT00000014440	ZNF618
ENSGALT00000021871	TMF1	ENSGALT00000014450	
ENSGALT00000022020	TBC1D1	ENSGALT00000014481	
ENSGALT00000022062		ENSGALT00000014545	NEXN
ENSGALT00000022096		ENSGALT00000014566	C10orf118
ENSGALT00000022114		ENSGALT00000014574	DBF4
ENSGALT00000022138	FBXO5	ENSGALT00000014612	GNB4
ENSGALT00000022352	GFPT2	ENSGALT00000014643	
ENSGALT00000022435	HECA	ENSGALT00000014649	CPED1
ENSGALT00000022454		ENSGALT00000014661	SMG5
ENSGALT00000022505	PCYOX1	ENSGALT00000014694	CCDC141
ENSGALT00000022698	WASF2	ENSGALT00000014731	
ENSGALT00000022738	SSPN	ENSGALT00000014750	CDK14
ENSGALT00000022808	TM7SF3	ENSGALT00000014752	
ENSGALT00000022891	FCGBP	ENSGALT00000014767	CD99L2
ENSGALT00000022946		ENSGALT00000014774	MAN1B1
ENSGALT00000022979	GPR155	ENSGALT00000014776	ZNF106
ENSGALT00000023065		ENSGALT00000014779	FAM160B1
ENSGALT00000023144		ENSGALT00000014791	
ENSGALT00000023157	NECAP1	ENSGALT00000014827	

ENSGALT00000023233		ENSGALT00000014832	
ENSGALT00000023323		ENSGALT00000014902	
ENSGALT00000023325	EVI5L	ENSGALT00000014913	CHGB
ENSGALT00000023478		ENSGALT00000014951	FMR1
ENSGALT00000023483		ENSGALT00000014990	
ENSGALT00000023495		ENSGALT00000015009	
ENSGALT00000023620		ENSGALT00000015057	PLD1
ENSGALT00000023630	NR2F6	ENSGALT00000015070	
ENSGALT00000023648		ENSGALT00000015071	TMEM62
ENSGALT00000023686	CLCN1	ENSGALT00000015124	TELO2
ENSGALT00000023700	DHX29	ENSGALT00000015176	
ENSGALT00000023728		ENSGALT00000015190	
ENSGALT00000023780	CWC27	ENSGALT00000015198	
ENSGALT00000023833		ENSGALT00000015203	
ENSGALT00000023839	SREK1	ENSGALT00000015210	TRIM63
ENSGALT00000023918	HOMER1	ENSGALT00000015276	
ENSGALT00000023934	TTC33	ENSGALT00000015328	MYNN
ENSGALT00000023962	PLCXD3	ENSGALT00000015332	BROX
ENSGALT00000023965	TRDN	ENSGALT00000015343	RGS10
ENSGALT00000023976		ENSGALT00000015350	KIAA0247
ENSGALT00000023982		ENSGALT00000015375	INPP5F
ENSGALT00000023984	SMPDL3A	ENSGALT00000015385	
ENSGALT00000023995		ENSGALT00000015418	FNIP2
ENSGALT00000024082	ENC1	ENSGALT00000015427	
ENSGALT00000024095	ABHD3	ENSGALT00000015431	
ENSGALT00000024112	GTF2E1	ENSGALT00000015464	TMA16
ENSGALT00000024149	LRRC58	ENSGALT00000015496	
ENSGALT00000024191	LYAR	ENSGALT00000015511	TMEM192
ENSGALT00000024248	KIAA1919	ENSGALT00000015526	
ENSGALT00000024341		ENSGALT00000015687	
ENSGALT00000024372		ENSGALT00000015751	
ENSGALT00000024400		ENSGALT00000015787	
ENSGALT00000024545	DTNA	ENSGALT00000015796	
ENSGALT00000024590	CD164	ENSGALT00000015841	PCDH18
ENSGALT00000024600	GPR161	ENSGALT00000015853	
ENSGALT00000024611	COL8A1	ENSGALT00000015878	
ENSGALT00000024628	RB1CC1	ENSGALT00000015887	
ENSGALT00000024644	CMSS1	ENSGALT00000015909	NAA15
ENSGALT00000024648	FILIP1L	ENSGALT00000015916	PTPN14
ENSGALT00000024669	RGMB	ENSGALT00000015940	
ENSGALT00000024692	TFG	ENSGALT00000015941	SETD7
ENSGALT00000024704	ABI3BP	ENSGALT00000015973	CCDC173

ENSGALT00000024761	RTN4IP1	ENSGALT00000015996	PPIG
ENSGALT00000024772	CBLB	ENSGALT00000015998	NKTR
ENSGALT00000024777	BBX	ENSGALT00000016025	
ENSGALT00000024828	HOOK3	ENSGALT00000016037	
ENSGALT00000024907	FKTN	ENSGALT00000016100	
ENSGALT00000024944	IGSF3	ENSGALT00000016160	
ENSGALT00000024957		ENSGALT00000016212	SLC3A1
ENSGALT00000024998	GGH	ENSGALT00000016236	ARHGAP5
ENSGALT00000025000		ENSGALT00000016239	AKAP6
ENSGALT00000025048	CSPP1	ENSGALT00000016289	BAZ1A
ENSGALT00000025061	TADA2B	ENSGALT00000016303	
ENSGALT00000025114	HTRA3	ENSGALT00000016329	SUPT7L
ENSGALT00000025119		ENSGALT00000016376	KDM4A
ENSGALT00000025131	SLCO5A1	ENSGALT00000016420	MBIP
ENSGALT00000025157	ZCCHC9	ENSGALT00000016432	DMAP1
ENSGALT00000025160	BACH2	ENSGALT00000016435	ERI3
ENSGALT00000025170		ENSGALT00000016441	
ENSGALT00000025199	EDIL3	ENSGALT00000016446	SCLT1
ENSGALT00000025304	KIAA0368	ENSGALT00000016472	PLK3
ENSGALT00000025330		ENSGALT00000016500	SEC23A
ENSGALT00000025350	SVEP1	ENSGALT00000016532	PNN
ENSGALT00000025447		ENSGALT00000016540	SLC35B2
ENSGALT00000025465	ADAMTS1	ENSGALT00000016545	ZFC3H1
ENSGALT00000025470	ADAMTS5	ENSGALT00000016548	RFX3
ENSGALT00000025493		ENSGALT00000016551	SLC29A1
ENSGALT00000025494	USP16	ENSGALT00000016584	
ENSGALT00000025499	LRRCC1	ENSGALT00000016625	
ENSGALT00000025551	PRSS35	ENSGALT00000016627	TOE1
ENSGALT00000025559	PGM3	ENSGALT00000016690	LTBP2
ENSGALT00000025624	SENP6	ENSGALT00000016704	AREL1
ENSGALT00000025653		ENSGALT00000016725	TMEM63B
ENSGALT00000025692		ENSGALT00000016788	MAST2
ENSGALT00000025750	PANK2	ENSGALT00000016792	NEK9
ENSGALT00000025780		ENSGALT00000016835	
ENSGALT00000025788	ITSN1	ENSGALT00000016850	GPATCH2L
ENSGALT00000025829	ATRN	ENSGALT00000016854	POMGNT1
ENSGALT00000025839		ENSGALT00000016888	ESRRB
ENSGALT00000025855	RRM2B	ENSGALT00000016892	VASH1
ENSGALT00000025999		ENSGALT00000016897	FAM13A
ENSGALT00000026040	TSNARE1	ENSGALT00000016908	
ENSGALT00000026043	PTP4A3	ENSGALT00000016916	TJAP1
ENSGALT00000026044	GPR20	ENSGALT00000016923	

ENSGALT00000026110	CBS	ENSGALT00000016945	TMEM63C
ENSGALT00000026117		ENSGALT00000016973	POMT2
ENSGALT00000026131		ENSGALT00000016977	ZNF318
ENSGALT00000026133		ENSGALT00000016985	TM4SF18
ENSGALT00000026230	PHF3	ENSGALT00000017131	ALDH18A1
ENSGALT00000026264	DST	ENSGALT00000017142	
ENSGALT00000026295		ENSGALT00000017184	
ENSGALT00000026321		ENSGALT00000017211	
ENSGALT00000026331	ICK	ENSGALT00000017215	STON2
ENSGALT00000026335		ENSGALT00000017218	STRN
ENSGALT00000026351	NDUFB9	ENSGALT00000017221	SEL1L
ENSGALT00000026385		ENSGALT00000017295	ZCCHC11
ENSGALT00000026475		ENSGALT00000017317	ENPP6
ENSGALT00000026492	TAF2	ENSGALT00000017359	TDP1
ENSGALT00000026827	ZNF395	ENSGALT00000017393	SECISBP2
ENSGALT00000026905	TRAM2	ENSGALT00000017399	
ENSGALT00000026925		ENSGALT00000017485	
ENSGALT00000027052		ENSGALT00000017502	TRIP11
ENSGALT00000027119	FHL2	ENSGALT00000017630	BTBD7
ENSGALT00000027129	GCC2	ENSGALT00000017687	ASB2
ENSGALT00000027171	CUL4A	ENSGALT00000017695	PAQR3
ENSGALT00000027237		ENSGALT00000017703	ANTXR2
ENSGALT00000027373		ENSGALT00000017844	
ENSGALT00000027403	RGCC	ENSGALT00000017877	
ENSGALT00000027416	EPSTI1	ENSGALT00000017890	NID1
ENSGALT00000027460		ENSGALT00000017907	IRF2BP2
ENSGALT00000027514		ENSGALT00000017973	AK4
ENSGALT00000027526	NHLRC3	ENSGALT00000017992	AFF1
ENSGALT00000027571		ENSGALT00000018021	COBLL1
ENSGALT00000027598		ENSGALT00000018034	
ENSGALT00000027738	GUCY1A2	ENSGALT00000018056	KCNH7
ENSGALT00000027822	PANX1	ENSGALT00000018061	CCSAP
ENSGALT00000027863	NOX4	ENSGALT00000018067	
ENSGALT00000027945	RAD51AP1	ENSGALT00000018125	SLC35D1
ENSGALT00000027995	FAM168A	ENSGALT00000018137	
ENSGALT00000028002	P2RY2	ENSGALT00000018177	
ENSGALT00000028035	STIM1	ENSGALT00000018181	HHIPL1
ENSGALT00000029632	DDI2	ENSGALT00000018196	
ENSGALT00000029722	RNF41	ENSGALT00000018212	WIPF3
ENSGALT00000029784		ENSGALT00000018221	CEP290
ENSGALT00000029798	KNOP1	ENSGALT00000018228	FAM120B
ENSGALT00000029889	NRGN	ENSGALT00000018257	TMTC3

ENSGALT00000030355	HLCS	ENSGALT00000018268	
ENSGALT00000030387		ENSGALT00000018296	SLC25A29
ENSGALT00000030592	PICK1	ENSGALT00000018346	
ENSGALT00000030620		ENSGALT00000018348	PLCL2
ENSGALT00000031095		ENSGALT00000018374	
ENSGALT00000031228	ACBD7	ENSGALT00000018386	
ENSGALT00000031915	PIGF	ENSGALT00000018404	EEA1
ENSGALT00000031945	TGFB2	ENSGALT00000018414	UBE2E1
ENSGALT00000032260		ENSGALT00000018417	DACT2
ENSGALT00000032288	MAP9	ENSGALT00000018542	TTLL4
ENSGALT00000032362		ENSGALT00000018582	MSH4
ENSGALT00000032399		ENSGALT00000018598	
ENSGALT00000032414	VSIG4	ENSGALT00000018603	SLC4A7
ENSGALT00000032565		ENSGALT00000018652	
ENSGALT00000032587		ENSGALT00000018662	MFHAS1
ENSGALT00000032650	MYBPC3	ENSGALT00000018666	TNFAIP2
ENSGALT00000032683		ENSGALT00000018667	GADL1
ENSGALT00000032690	EFCAB4A	ENSGALT00000018669	
ENSGALT00000032718	KCNJ11	ENSGALT00000018681	CCNI
ENSGALT00000032836	KDM4C	ENSGALT00000018707	OSBPL10
ENSGALT00000032856		ENSGALT00000018764	
ENSGALT00000032871	DIMT1	ENSGALT00000018770	ANO10
ENSGALT00000033197	BTRC	ENSGALT00000018773	
ENSGALT00000033354		ENSGALT00000018812	EAF1
ENSGALT00000033492		ENSGALT00000018822	
ENSGALT00000033813		ENSGALT00000018857	PARK2
ENSGALT00000033873		ENSGALT00000018933	PTPN4
ENSGALT00000034008		ENSGALT00000019041	
ENSGALT00000034796		ENSGALT00000019050	AHNAK2
ENSGALT00000034856	QSOX2	ENSGALT00000019065	FNDC1
ENSGALT00000035033	ANGPTL7	ENSGALT00000019105	SEMA5B
ENSGALT00000035194		ENSGALT00000019108	
ENSGALT00000035212		ENSGALT00000019133	ACKR4
ENSGALT00000035232		ENSGALT00000019134	
ENSGALT00000035373		ENSGALT00000019136	
ENSGALT00000036007		ENSGALT00000019144	
ENSGALT00000036281		ENSGALT00000019167	
ENSGALT00000036294		ENSGALT00000019224	
ENSGALT00000036295		ENSGALT00000019246	
ENSGALT00000036623	MTDH	ENSGALT00000019273	LZTFL1
ENSGALT00000036642		ENSGALT00000019277	SYNE2
ENSGALT00000036926		ENSGALT00000019286	HEG1

ENSGALT00000037229		ENSGALT00000019307	ANKRD50
ENSGALT00000037436		ENSGALT00000019329	SACM1L
ENSGALT00000037581	AKAP12	ENSGALT00000019330	TTC26
ENSGALT00000037654	SSBP2	ENSGALT00000019340	
ENSGALT00000037747	PRPF40A	ENSGALT00000019393	ZDHHC3
ENSGALT00000037951		ENSGALT00000019409	SLC38A6
ENSGALT00000037963		ENSGALT00000019464	DESI1
ENSGALT00000038163		ENSGALT00000019482	PMM1
ENSGALT00000038198		ENSGALT00000019514	ZC3H7B
ENSGALT00000038228	FXN	ENSGALT00000019581	SEC24D
ENSGALT00000038342		ENSGALT00000019611	
ENSGALT00000038633		ENSGALT00000019620	
ENSGALT00000038829		ENSGALT00000019637	SGSM3
ENSGALT00000039021	AGPAT2	ENSGALT00000019676	ARPP21
ENSGALT00000039073	UPF3B	ENSGALT00000019679	LARP7
ENSGALT00000039093	MLF1	ENSGALT00000019695	TRANK1
ENSGALT00000039136	PCIF1	ENSGALT00000019698	OSBPL11
ENSGALT00000039194	C11orf89	ENSGALT00000019817	
ENSGALT00000039213	CLDN19	ENSGALT00000019837	MIEF1
ENSGALT00000039585		ENSGALT00000019895	PLA2G12A
ENSGALT00000039672		ENSGALT00000019898	MGAT5
ENSGALT00000039878		ENSGALT00000019931	CCNT2
ENSGALT00000039922		ENSGALT00000019966	
ENSGALT00000040133	PLXDC1	ENSGALT00000020038	LRRFIP2
ENSGALT00000040414		ENSGALT00000020068	PPP3CA
ENSGALT00000040421	RALY	ENSGALT00000020115	MANBA
ENSGALT00000040443	RPUSD2	ENSGALT00000020121	
ENSGALT00000040502		ENSGALT00000020165	SASH1
ENSGALT00000040562		ENSGALT00000020216	
ENSGALT00000040574		ENSGALT00000020285	MTHFD1L
ENSGALT00000040680	SCN4A	ENSGALT00000020337	SBF1
ENSGALT00000041120	FSTL3	ENSGALT00000020353	
ENSGALT00000041155	FAM46B	ENSGALT00000020354	MIS18BP1
ENSGALT00000041341		ENSGALT00000020410	
ENSGALT00000041392	POGZ	ENSGALT00000020417	
ENSGALT00000041398		ENSGALT00000020442	BRD9
		ENSGALT00000020444	TPPP
		ENSGALT00000020462	PRPF40A
		ENSGALT00000020486	GPD2
		ENSGALT00000020490	
		ENSGALT00000020497	
		ENSGALT00000020504	HMGXB4

ENSGALT00000020544	FRMD3
ENSGALT00000020552	GKAP1
ENSGALT00000020569	AGTPBP1
ENSGALT00000020597	COL15A1
ENSGALT00000020623	BTBD11
ENSGALT00000020625	
ENSGALT00000020631	
ENSGALT00000020647	CTDP1
ENSGALT00000020679	NRSN1
ENSGALT00000020695	
ENSGALT00000020740	
ENSGALT00000020743	
ENSGALT00000020831	SYCP3
ENSGALT00000020885	
ENSGALT00000020904	PRPF4B
ENSGALT00000020947	MYLK4
ENSGALT00000020949	
ENSGALT00000021064	MCM4
ENSGALT00000021086	
ENSGALT00000021101	KIAA1551
ENSGALT00000021202	B4GALNT3
ENSGALT00000021213	ERC1
ENSGALT00000021258	MARCH6
ENSGALT00000021277	
ENSGALT00000021280	SBK2
ENSGALT00000021282	BCL2L13
ENSGALT00000021284	
ENSGALT00000021306	MTRR
ENSGALT00000021320	ADCY2
ENSGALT00000021439	PLEKHA5
ENSGALT00000021448	TMEM245
ENSGALT00000021521	BCAT1
ENSGALT00000021578	SLC39A6
ENSGALT00000021611	
ENSGALT00000021647	LOXL3
ENSGALT00000021658	ARHGEF11
ENSGALT00000021722	
ENSGALT00000021812	
ENSGALT00000021823	CMA1
ENSGALT00000021871	TMF1
ENSGALT00000021898	SORBS2
ENSGALT00000022062	

ENSGALT00000022114	
ENSGALT00000022138	FBXO5
ENSGALT00000022147	MTUS1
ENSGALT00000022180	LRRC2
ENSGALT00000022219	MICU3
ENSGALT00000022257	UROS
ENSGALT00000022325	TMEM181
ENSGALT00000022352	GFPT2
ENSGALT00000022353	
ENSGALT00000022435	HECA
ENSGALT00000022499	
ENSGALT00000022505	PCYOX1
ENSGALT00000022607	AHI1
ENSGALT00000022662	EYA4
ENSGALT00000022698	WASF2
ENSGALT00000022704	LAMA2
ENSGALT00000022726	
ENSGALT00000022738	SSPN
ENSGALT00000022808	TM7SF3
ENSGALT00000022820	
ENSGALT00000022856	CYB5R3
ENSGALT00000022891	FCGBP
ENSGALT00000022893	SUV420H1
ENSGALT00000022946	
ENSGALT00000022979	GPR155
ENSGALT00000023043	TMEM33
ENSGALT00000023090	
ENSGALT00000023095	N4BP2
ENSGALT00000023097	
ENSGALT00000023131	
ENSGALT00000023144	
ENSGALT00000023145	SMIM14
ENSGALT00000023157	NECAP1
ENSGALT00000023225	
ENSGALT00000023231	ZCCHC4
ENSGALT00000023246	
ENSGALT00000023325	EVI5L
ENSGALT00000023354	
ENSGALT00000023478	
ENSGALT00000023495	
ENSGALT00000023630	NR2F6
ENSGALT00000023647	RHOBTB3

ENSGALT00000023680	
ENSGALT00000023694	MEGF10
ENSGALT00000023700	DHX29
ENSGALT00000023723	SLC38A9
ENSGALT00000023728	
ENSGALT00000023753	
ENSGALT00000023780	CWC27
ENSGALT00000023833	
ENSGALT00000023834	WARS2
ENSGALT00000023839	SREK1
ENSGALT00000023868	PIK3R1
ENSGALT00000023918	HOMER1
ENSGALT00000023921	EMILIN2
ENSGALT00000023962	PLCXD3
ENSGALT00000023965	TRDN
ENSGALT00000023976	
ENSGALT00000023982	
ENSGALT00000023984	SMPDL3A
ENSGALT00000023995	
ENSGALT00000024023	CEP85L
ENSGALT00000024049	HSPB3
ENSGALT00000024082	ENC1
ENSGALT00000024112	GTF2E1
ENSGALT00000024149	LRRC58
ENSGALT00000024162	
ENSGALT00000024169	RBBP8
ENSGALT00000024191	LYAR
ENSGALT00000024248	KIAA1919
ENSGALT00000024267	
ENSGALT00000024268	KIAA2026
ENSGALT00000024321	ARHGAP31
ENSGALT00000024341	
ENSGALT00000024346	ZBTB20
ENSGALT00000024372	
ENSGALT00000024382	ZNF521
ENSGALT00000024398	
ENSGALT00000024400	
ENSGALT00000024483	
ENSGALT00000024492	
ENSGALT00000024497	GAREM
ENSGALT00000024590	CD164
ENSGALT00000024600	GPR161

ENSGALT00000024605	
ENSGALT00000024611	COL8A1
ENSGALT00000024628	RB1CC1
ENSGALT00000024648	FILIP1L
ENSGALT00000024669	RGMB
ENSGALT00000024704	ABI3BP
ENSGALT00000024769	
ENSGALT00000024772	CBLB
ENSGALT00000024777	BBX
ENSGALT00000024811	PHLDB2
ENSGALT00000024828	HOOK3
ENSGALT00000024855	PROS1
ENSGALT00000024861	ARL13B
ENSGALT00000024871	PENK
ENSGALT00000024901	MPZL1
ENSGALT00000024931	PTGFRN
ENSGALT00000024944	IGSF3
ENSGALT00000024962	
ENSGALT00000024965	ASPH
ENSGALT00000024970	
ENSGALT00000024998	GGH
ENSGALT00000025000	
ENSGALT00000025002	GBE1
ENSGALT00000025048	CSPP1
ENSGALT00000025106	
ENSGALT00000025114	HTRA3
ENSGALT00000025119	
ENSGALT00000025131	SLCO5A1
ENSGALT00000025157	ZCCHC9
ENSGALT00000025170	
ENSGALT00000025199	EDIL3
ENSGALT00000025264	FAM193A
ENSGALT00000025294	NRIP1
ENSGALT00000025295	UGCG
ENSGALT00000025296	
ENSGALT00000025302	
ENSGALT00000025330	
ENSGALT00000025350	SVEP1
ENSGALT00000025361	
ENSGALT00000025428	
ENSGALT00000025446	PM20D2
ENSGALT00000025447	

ENSGALT00000025494	USP16
ENSGALT00000025499	LRRCC1
ENSGALT00000025504	CA13
ENSGALT00000025521	
ENSGALT00000025528	NT5E
ENSGALT00000025551	PRSS35
ENSGALT00000025559	PGM3
ENSGALT00000025595	HUNK
ENSGALT00000025610	PHIP
ENSGALT00000025624	SENP6
ENSGALT00000025628	FILIP1
ENSGALT00000025653	
ENSGALT00000025680	PAXBP1
ENSGALT00000025688	THNSL2
ENSGALT00000025692	
ENSGALT00000025742	DPY19L4
ENSGALT00000025775	FASTKD5
ENSGALT00000025788	ITSN1
ENSGALT00000025829	ATRN
ENSGALT00000025839	
ENSGALT00000025841	
ENSGALT00000025855	RRM2B
ENSGALT00000025859	TTC3
ENSGALT00000025873	
ENSGALT00000025882	BRWD1
ENSGALT00000025913	ABRA
ENSGALT00000025927	RAB11FIP5
ENSGALT00000026001	RHPN1
ENSGALT00000026021	C2CD2
ENSGALT00000026026	PSCA
ENSGALT00000026044	GPR20
ENSGALT00000026088	WDR4
ENSGALT00000026104	
ENSGALT00000026133	
ENSGALT00000026194	
ENSGALT00000026211	RPGR
ENSGALT00000026230	PHF3
ENSGALT00000026245	
ENSGALT00000026250	
ENSGALT00000026253	BEND6
ENSGALT00000026264	DST
ENSGALT00000026321	

ENSGALT00000026331	ICK
ENSGALT00000026385	
ENSGALT00000026390	FBXO25
ENSGALT00000026404	
ENSGALT00000026441	CNKSR2
ENSGALT00000026483	CXorf23
ENSGALT00000026486	ASAP2
ENSGALT00000026492	TAF2
ENSGALT00000026498	MAP3K15
ENSGALT00000026525	RRM2
ENSGALT00000026569	SMC6
ENSGALT00000026609	ATAD2B
ENSGALT00000026630	ZNF512
ENSGALT00000026711	
ENSGALT00000026743	
ENSGALT00000026856	MXRA5
ENSGALT00000026872	XKR6
ENSGALT00000026878	
ENSGALT00000026905	TRAM2
ENSGALT00000026909	
ENSGALT00000026922	
ENSGALT00000026925	
ENSGALT00000026992	JADE3
ENSGALT00000026993	
ENSGALT00000027052	
ENSGALT00000027129	GCC2
ENSGALT00000027168	
ENSGALT00000027171	CUL4A
ENSGALT00000027208	
ENSGALT00000027231	
ENSGALT00000027237	
ENSGALT00000027243	METTL21C
ENSGALT00000027245	TPP2
ENSGALT00000027273	DOCK9
ENSGALT00000027292	RAP2A
ENSGALT00000027391	
ENSGALT00000027416	EPSTI1
ENSGALT00000027439	ZC3H13
ENSGALT00000027454	NUDT15
ENSGALT00000027462	RCBTB2
ENSGALT00000027478	TRIM13
ENSGALT00000027488	INTS6

ENSGALT00000027514	
ENSGALT00000027526	NHLRC3
ENSGALT00000027566	STARD13
ENSGALT00000027571	
ENSGALT00000027575	N4BP2L1
ENSGALT00000027616	LNX2
ENSGALT00000027620	
ENSGALT00000027648	MTMR6
ENSGALT00000027657	
ENSGALT00000027658	SACS
ENSGALT00000027676	
ENSGALT00000027690	
ENSGALT00000027709	
ENSGALT00000027716	NPAT
ENSGALT00000027722	
ENSGALT00000027735	CWF19L2
ENSGALT00000027799	MAML2
ENSGALT00000027858	FOLH1
ENSGALT00000027863	NOX4
ENSGALT00000027884	PCF11
ENSGALT00000027945	RAD51AP1
ENSGALT00000027985	
ENSGALT00000027995	FAM168A
ENSGALT00000028001	
ENSGALT00000028002	P2RY2
ENSGALT00000028034	
ENSGALT00000028035	STIM1
ENSGALT00000028064	LURAP1
ENSGALT00000028268	GPD1
ENSGALT00000028271	ANKRD46
ENSGALT00000029552	
ENSGALT00000029632	DDI2
ENSGALT00000029798	KNOP1
ENSGALT00000029912	
ENSGALT00000030355	HLCS
ENSGALT00000030405	
ENSGALT00000030447	DPT
ENSGALT00000030521	
ENSGALT00000030592	PICK1
ENSGALT00000030811	
ENSGALT00000030862	PAG1
ENSGALT00000030930	

ENSGALT00000031095	
ENSGALT00000031116	RP9
ENSGALT00000031228	ACBD7
ENSGALT00000031337	GLDN
ENSGALT00000031388	CHAD
ENSGALT00000031518	
ENSGALT00000031528	CLIC5
ENSGALT00000031751	KIAA0408
ENSGALT00000031776	
ENSGALT00000031915	PIGF
ENSGALT00000031945	TGFB2
ENSGALT00000032107	PDGFRL
ENSGALT00000032235	
ENSGALT00000032288	MAP9
ENSGALT00000032565	
ENSGALT00000032623	
ENSGALT00000032650	MYBPC3
ENSGALT00000032683	
ENSGALT00000032690	EFCAB4A
ENSGALT00000032768	TOPORS
ENSGALT00000032816	NMRK1
ENSGALT00000032836	KDM4C
ENSGALT00000032856	
ENSGALT00000032871	DIMT1
ENSGALT00000033197	BTRC
ENSGALT00000033265	
ENSGALT00000033345	LRRC8D
ENSGALT00000033354	
ENSGALT00000033492	
ENSGALT00000033524	AURKA
ENSGALT00000033559	RAB31
ENSGALT00000033666	S100B
ENSGALT00000033697	
ENSGALT00000033717	
ENSGALT00000034212	
ENSGALT00000034383	
ENSGALT00000034402	RNF26
ENSGALT00000034432	B3GNT9
ENSGALT00000034621	
ENSGALT00000034695	
ENSGALT00000034743	
ENSGALT00000034751	

ENSGALT00000034784	TMEM8C
ENSGALT00000034796	
ENSGALT00000034799	PRR11
ENSGALT00000034856	QSOX2
ENSGALT00000035033	ANGPTL7
ENSGALT00000035194	
ENSGALT00000035245	
ENSGALT00000035373	
ENSGALT00000035378	
ENSGALT00000036037	
ENSGALT00000036371	
ENSGALT00000036503	
ENSGALT00000036592	
ENSGALT00000036623	MTDH
ENSGALT00000036640	
ENSGALT00000036642	
ENSGALT00000036897	HHLA2
ENSGALT00000037148	
ENSGALT00000037170	
ENSGALT00000037229	
ENSGALT00000037476	
ENSGALT00000037581	AKAP12
ENSGALT00000037604	
ENSGALT00000037615	CDV3
ENSGALT00000037654	SSBP2
ENSGALT00000037674	
ENSGALT00000037747	PRPF40A
ENSGALT00000037892	
ENSGALT00000037963	
ENSGALT00000038145	
ENSGALT00000038163	
ENSGALT00000038198	
ENSGALT00000038252	
ENSGALT00000038316	
ENSGALT00000038660	
ENSGALT00000038751	
ENSGALT00000039073	UPF3B
ENSGALT00000039082	
ENSGALT00000039194	C11orf89
ENSGALT00000039213	CLDN19
ENSGALT00000039224	
ENSGALT00000039585	

ENSGALT00000039617	
ENSGALT00000039619	
ENSGALT00000039676	IER5
ENSGALT00000039690	
ENSGALT00000039703	
ENSGALT00000039833	
ENSGALT00000039989	MEGF9
ENSGALT00000040104	RCN1
ENSGALT00000040133	PLXDC1
ENSGALT00000040136	
ENSGALT00000040176	PLEKHG4
ENSGALT00000040414	
ENSGALT00000040502	
ENSGALT00000040550	PI4KB
ENSGALT00000040562	
ENSGALT00000040574	
ENSGALT00000040656	SMCR8
ENSGALT00000040660	
ENSGALT00000040996	STC2
ENSGALT00000041120	FSTL3
ENSGALT00000041141	
ENSGALT00000041341	
ENSGALT00000041392	POGZ
ENSGALT00000041398	

DEGs between RJF and WL		DEGs between CH and AA	
**Ensembl Transcript ID**	**HGNC symbol**	**Ensembl Transcript ID**	**HGNC symbol**
ENSGALT00000029647		ENSGALT00000000136	ANTXR1
ENSGALT00000000407		ENSGALT00000000188	
ENSGALT00000040207		ENSGALT00000000518	SLC25A37
ENSGALT00000000555		ENSGALT00000000898	
ENSGALT00000000587		ENSGALT00000001398	
ENSGALT00000014902		ENSGALT00000001427	
ENSGALT00000014829	DPP7	ENSGALT00000001603	ELN
ENSGALT00000000671	LONP1	ENSGALT00000001871	
ENSGALT00000014774	MAN1B1	ENSGALT00000002172	YDJC
ENSGALT00000001420		ENSGALT00000002291	
ENSGALT00000014744		ENSGALT00000002490	
ENSGALT00000000806	RANBP3	ENSGALT00000002566	SETBP1
ENSGALT00000014675		ENSGALT00000002567	
ENSGALT00000014665	TRAF2	ENSGALT00000002828	SH2B2

ENSGALT00000014657		ENSGALT00000003038	
ENSGALT00000001029		ENSGALT00000003157	AHCY
ENSGALT00000014614	RABL6	ENSGALT00000003449	
ENSGALT00000001477	MAP2K4	ENSGALT00000003570	
ENSGALT00000023397		ENSGALT00000003649	
ENSGALT00000002041		ENSGALT00000004015	PCDH12
ENSGALT00000002095		ENSGALT00000004115	
ENSGALT00000014372	SLC31A1	ENSGALT00000004499	SARDH
ENSGALT00000002349		ENSGALT00000004573	ALPK2
ENSGALT00000002374		ENSGALT00000004876	
ENSGALT00000002490		ENSGALT00000005088	TSPO2
ENSGALT00000002733	MFSD11	ENSGALT00000005219	ADAMTS 12
ENSGALT00000002759		ENSGALT00000005294	
ENSGALT00000002784		ENSGALT00000005581	ARRDC2
ENSGALT00000002885	PRPSAP1	ENSGALT00000005664	
ENSGALT00000021823	CMA1	ENSGALT00000005829	
ENSGALT00000014039		ENSGALT00000005844	PAPSS2
ENSGALT00000021812		ENSGALT00000005940	
ENSGALT00000003381		ENSGALT00000006575	
ENSGALT00000003433		ENSGALT00000006617	SLC16A6
ENSGALT00000003475		ENSGALT00000006667	
ENSGALT00000011643		ENSGALT00000007467	
ENSGALT00000002831		ENSGALT00000007566	
ENSGALT00000003804	SAP30BP	ENSGALT00000007643	TNFAIP8 L3
ENSGALT00000004306		ENSGALT00000007669	EPB42
ENSGALT00000004555		ENSGALT00000007728	APLNR
ENSGALT00000004579	NOTUM	ENSGALT00000008756	SDR42E1
ENSGALT00000034713	TOM1L1	ENSGALT00000009328	SEPT2
ENSGALT00000004865		ENSGALT00000009337	BCAR3
ENSGALT00000004896	PCTP	ENSGALT00000009416	ALOX5
ENSGALT00000005054		ENSGALT00000009723	
ENSGALT00000034695		ENSGALT00000009864	
ENSGALT00000005180	SUZ12	ENSGALT00000010002	
ENSGALT00000001193		ENSGALT00000010763	
ENSGALT00000008122		ENSGALT00000010875	
ENSGALT00000001236	DOT1L	ENSGALT00000010963	KLHDC8 B
ENSGALT00000001472	AP3D1	ENSGALT00000011206	BAIAP2
ENSGALT00000005503	ERN1	ENSGALT00000011644	
ENSGALT00000034662	TEX2	ENSGALT00000011897	MAP3K8

ENSGALT00000007962	SLC27A4	ENSGALT00000012048	
ENSGALT00000006110	MKNK2	ENSGALT00000012483	
ENSGALT00000007881	COQ4	ENSGALT00000012861	PRR5L
ENSGALT00000001502	BTBD2	ENSGALT00000013006	
ENSGALT00000005647	SMURF2	ENSGALT00000013519	OBFC1
ENSGALT00000005672		ENSGALT00000013524	
ENSGALT00000005785		ENSGALT00000013679	MPP4
ENSGALT00000023521		ENSGALT00000013683	ANKRD12
ENSGALT00000029695	ABHD17A	ENSGALT00000013844	
ENSGALT00000001557		ENSGALT00000013978	
ENSGALT00000007637		ENSGALT00000014372	SLC31A1
ENSGALT00000007631	PTGES2	ENSGALT00000014545	NEXN
ENSGALT00000007603		ENSGALT00000014694	CCDC141
ENSGALT00000005864		ENSGALT00000014902	
ENSGALT00000006288		ENSGALT00000015142	
ENSGALT00000001723	UBXN6	ENSGALT00000015210	TRIM63
ENSGALT00000006617	SLC16A6	ENSGALT00000015253	
ENSGALT00000006701	WIPI1	ENSGALT00000015414	
ENSGALT00000035511	PLIN3	ENSGALT00000015566	
ENSGALT00000038499	CDC42EP4	ENSGALT00000015790	
ENSGALT00000002009		ENSGALT00000016017	
ENSGALT00000029664		ENSGALT00000016908	
ENSGALT00000007424	ZER1	ENSGALT00000016985	TM4SF18
ENSGALT00000002098	POLRMT	ENSGALT00000017109	BEND5
ENSGALT00000007292	NPLOC4	ENSGALT00000017560	
ENSGALT00000007279	LRRC8A	ENSGALT00000017724	
ENSGALT00000011145	SLC38A10	ENSGALT00000018296	SLC25A29
ENSGALT00000007386		ENSGALT00000018392	
ENSGALT00000007251		ENSGALT00000018404	EEA1
ENSGALT00000007108	CRAT	ENSGALT00000018472	TMCC3
ENSGALT00000003154	TMEM259	ENSGALT00000019065	FNDC1
ENSGALT00000006623		ENSGALT00000019137	TMEM108
ENSGALT00000006504	FNBP1	ENSGALT00000019167	
ENSGALT00000032738		ENSGALT00000019187	
ENSGALT00000019966		ENSGALT00000019230	APOLD1
ENSGALT00000005350	SPTB	ENSGALT00000019766	
ENSGALT00000006004	PPAPDC3	ENSGALT00000020038	LRRFIP2
ENSGALT00000005992	PRRC2B	ENSGALT00000020121	

ENSGALT00000011703		ENSGALT00000020184	
ENSGALT00000013753		ENSGALT00000020417	
ENSGALT00000011734	PCYT2	ENSGALT00000020490	
ENSGALT00000011818		ENSGALT00000020623	BTBD11
ENSGALT00000004127	SLC25A42	ENSGALT00000020631	
ENSGALT00000005537	TSC1	ENSGALT00000020714	
ENSGALT00000038280	EPN3	ENSGALT00000020759	
ENSGALT00000004779	ARMC6	ENSGALT00000020791	
ENSGALT00000004856	HOMER3	ENSGALT00000021101	KIAA155<br>1
ENSGALT00000031388	CHAD	ENSGALT00000021403	RERGL
ENSGALT00000005397	RALGDS	ENSGALT00000021880	
ENSGALT00000012523	NAT9	ENSGALT00000021898	SORBS2
ENSGALT00000005285		ENSGALT00000022087	NR4A3
ENSGALT00000012560		ENSGALT00000022454	
ENSGALT00000005056		ENSGALT00000022662	EYA4
ENSGALT00000012890		ENSGALT00000023131	
ENSGALT00000005016	REXO4	ENSGALT00000023965	TRDN
ENSGALT00000005192	COMP	ENSGALT00000024023	CEP85L
ENSGALT00000012930		ENSGALT00000024049	HSPB3
ENSGALT00000005309		ENSGALT00000024095	ABHD3
ENSGALT00000007496		ENSGALT00000024577	NME7
ENSGALT00000007466		ENSGALT00000024673	SESN1
ENSGALT00000005348	LSM4	ENSGALT00000024871	PENK
ENSGALT00000013002	SLC25A19	ENSGALT00000024905	
ENSGALT00000005355		ENSGALT00000024931	PTGFRN
ENSGALT00000006156		ENSGALT00000024944	IGSF3
ENSGALT00000040443	RPUSD2	ENSGALT00000025106	
ENSGALT00000013018	KIAA0195	ENSGALT00000025338	
ENSGALT00000013031	CASKIN2	ENSGALT00000025383	
ENSGALT00000001658		ENSGALT00000025610	PHIP
ENSGALT00000005424		ENSGALT00000025628	FILIP1
ENSGALT00000006955		ENSGALT00000025999	
ENSGALT00000023312	RETSAT	ENSGALT00000026026	PSCA
ENSGALT00000004226	BRD3	ENSGALT00000026040	TSNARE<br>1
ENSGALT00000004139	RXRA	ENSGALT00000026104	
ENSGALT00000005655		ENSGALT00000026117	
ENSGALT00000039021	AGPAT2	ENSGALT00000026343	
ENSGALT00000006685		ENSGALT00000026525	RRM2
ENSGALT00000006531		ENSGALT00000026909	
ENSGALT00000040276	NAV2	ENSGALT00000026922	

ENSGALT00000006317		ENSGALT00000027071	AFF3
ENSGALT00000003726	SEC16A	ENSGALT00000027168	
ENSGALT00000005994		ENSGALT00000027194	IRS2
ENSGALT00000006018	SLC35E1	ENSGALT00000027403	RGCC
ENSGALT00000003018		ENSGALT00000027738	GUCY1A2
ENSGALT00000006230	EPS15L1	ENSGALT00000027822	PANX1
ENSGALT00000040172		ENSGALT00000027985	
ENSGALT00000019825		ENSGALT00000028002	P2RY2
ENSGALT00000031814		ENSGALT00000029461	
ENSGALT00000019813	ELP4	ENSGALT00000029552	
ENSGALT00000040104	RCN1	ENSGALT00000031372	ACER3
ENSGALT00000006310		ENSGALT00000031388	CHAD
ENSGALT00000002629		ENSGALT00000031518	
ENSGALT00000039281	TPM4	ENSGALT00000032336	NSDHL
ENSGALT00000019109		ENSGALT00000032587	
ENSGALT00000008620		ENSGALT00000032951	P2RY14
ENSGALT00000008698		ENSGALT00000033524	AURKA
ENSGALT00000008731	PARVA	ENSGALT00000033697	
ENSGALT00000008888	MICAL2	ENSGALT00000034001	
ENSGALT00000006684	DPP9	ENSGALT00000034078	
ENSGALT00000008932		ENSGALT00000034317	
ENSGALT00000008962		ENSGALT00000034319	
ENSGALT00000039859		ENSGALT00000034713	TOM1L1
ENSGALT00000006779		ENSGALT00000035373	
ENSGALT00000009054		ENSGALT00000035848	
ENSGALT00000009088	ADM	ENSGALT00000036018	EVA1B
ENSGALT00000009326	IPO7	ENSGALT00000036371	
ENSGALT00000009336		ENSGALT00000038194	
ENSGALT00000002236		ENSGALT00000038916	
ENSGALT00000009504		ENSGALT00000039082	
ENSGALT00000034894	STOM	ENSGALT00000039301	PDZD7
ENSGALT00000009649		ENSGALT00000039703	
ENSGALT00000034898		ENSGALT00000039878	
ENSGALT00000009745	CYP2R1	ENSGALT00000040564	
ENSGALT00000009835		ENSGALT00000040996	STC2
ENSGALT00000001877			
ENSGALT00000001117	PLA2G15		
ENSGALT00000001294	GINS3		
ENSGALT00000032718	KCNJ11		
ENSGALT00000001304			
ENSGALT00000001320			

ENSGALT00000001489	MMP15
ENSGALT00000001657	
ENSGALT00000001570	OLFML2A
ENSGALT00000010128	HPS5
ENSGALT00000022076	CCDC102A
ENSGALT00000001496	ARPC5L
ENSGALT00000001476	
ENSGALT00000010207	
ENSGALT00000001402	
ENSGALT00000001330	RALGPS1
ENSGALT00000010405	
ENSGALT00000001362	
ENSGALT00000002191	RANBP10
ENSGALT00000002429	
ENSGALT00000002498	EDC4
ENSGALT00000010585	
ENSGALT00000010620	
ENSGALT00000039194	C11orf89
ENSGALT00000010676	
ENSGALT00000010774	MOB2
ENSGALT00000010998	AP2A2
ENSGALT00000003282	TPPP3
ENSGALT00000003500	
ENSGALT00000003653	
ENSGALT00000003894	MTSS1L
ENSGALT00000040057	
ENSGALT00000011181	
ENSGALT00000011466	
ENSGALT00000011547	
ENSGALT00000011621	
ENSGALT00000004497	
ENSGALT00000017615	DTX4
ENSGALT00000011877	MED19
ENSGALT00000038761	RTN4RL2
ENSGALT00000004875	
ENSGALT00000004906	AMFR
ENSGALT00000005176	
ENSGALT00000005216	MBTPS1
ENSGALT00000010684	FBXO3
ENSGALT00000005262	OSGIN1
ENSGALT00000005367	NFATC3
ENSGALT00000023364	CAT

ENSGALT00000012705	
ENSGALT00000012829	COMMD9
ENSGALT00000012888	TRAF6
ENSGALT00000005659	
ENSGALT00000005666	
ENSGALT00000013052	
ENSGALT00000022785	
ENSGALT00000030003	
ENSGALT00000013121	
ENSGALT00000034245	
ENSGALT00000006188	ZNF423
ENSGALT00000006236	SIAH1
ENSGALT00000006378	
ENSGALT00000013147	
ENSGALT00000013183	
ENSGALT00000013191	
ENSGALT00000032650	MYBPC3
ENSGALT00000006611	GPT2
ENSGALT00000013356	
ENSGALT00000006871	MYLK3
ENSGALT00000006929	
ENSGALT00000013396	
ENSGALT00000013425	
ENSGALT00000006976	
ENSGALT00000007136	
ENSGALT00000007149	
ENSGALT00000007159	
ENSGALT00000013551	
ENSGALT00000013587	ATG13
ENSGALT00000035181	PFKFB1
ENSGALT00000007751	LRP3
ENSGALT00000007771	
ENSGALT00000013748	
ENSGALT00000007832	
ENSGALT00000013933	
ENSGALT00000013981	NDUFAF1
ENSGALT00000007938	KIAA0355
ENSGALT00000014145	MAPKBP1
ENSGALT00000008241	FAM96B
ENSGALT00000008251	
ENSGALT00000014672	TMEM87A
ENSGALT00000039221	

ENSGALT00000008465	
ENSGALT00000039170	
ENSGALT00000014809	
ENSGALT00000015102	ELMSAN1
ENSGALT00000038149	
ENSGALT00000015198	
ENSGALT00000029669	HOXC10
ENSGALT00000015283	
ENSGALT00000022454	
ENSGALT00000015312	
ENSGALT00000015350	KIAA0247
ENSGALT00000015368	
ENSGALT00000028069	
ENSGALT00000008792	
ENSGALT00000008794	
ENSGALT00000008799	
ENSGALT00000015576	
ENSGALT00000008821	
ENSGALT00000015588	
ENSGALT00000033760	
ENSGALT00000015687	
ENSGALT00000015691	EMC7
ENSGALT00000015856	FAM98B
ENSGALT00000015965	ZNF770
ENSGALT00000016005	
ENSGALT00000016127	
ENSGALT00000028346	
ENSGALT00000016156	AP4S1
ENSGALT00000009186	MTHFSD
ENSGALT00000009243	
ENSGALT00000009231	
ENSGALT00000016285	
ENSGALT00000016289	BAZ1A
ENSGALT00000016327	SRP54
ENSGALT00000016326	
ENSGALT00000016334	PPP2R3C
ENSGALT00000016344	
ENSGALT00000009439	
ENSGALT00000016417	
ENSGALT00000016500	SEC23A
ENSGALT00000016505	
ENSGALT00000016512	

ENSGALT00000016576	COQ6
ENSGALT00000016585	
ENSGALT00000016604	ALDH6A1
ENSGALT00000022441	TOMM40L
ENSGALT00000016641	
ENSGALT00000016690	LTBP2
ENSGALT00000016704	AREL1
ENSGALT00000010046	ANKRD11
ENSGALT00000016756	
ENSGALT00000016763	MLH3
ENSGALT00000016792	NEK9
ENSGALT00000016804	BATF
ENSGALT00000037707	
ENSGALT00000016918	IRF2BPL
ENSGALT00000017033	AHSA1
ENSGALT00000000074	
ENSGALT00000017069	
ENSGALT00000004641	
ENSGALT00000017211	
ENSGALT00000017221	SEL1L
ENSGALT00000004911	BFAR
ENSGALT00000000947	
ENSGALT00000017348	TTC8
ENSGALT00000017465	SMEK1
ENSGALT00000017485	
ENSGALT00000037601	
ENSGALT00000005479	
ENSGALT00000017577	LGMN
ENSGALT00000005546	TECPR1
ENSGALT00000001181	IST1
ENSGALT00000028074	
ENSGALT00000017630	BTBD7
ENSGALT00000017687	ASB2
ENSGALT00000001325	
ENSGALT00000018063	ATG2B
ENSGALT00000018066	
ENSGALT00000005765	FAM20C
ENSGALT00000005824	PRKAR1B
ENSGALT00000018196	
ENSGALT00000018280	
ENSGALT00000018316	WARS
ENSGALT00000018374	

ENSGALT00000034197	C7orf50
ENSGALT00000006456	
ENSGALT00000006473	MICALL2
ENSGALT00000018521	HSP90AA1
ENSGALT00000018539	WDR20
ENSGALT00000018544	ZNF839
ENSGALT00000018551	
ENSGALT00000006646	GAA
ENSGALT00000006656	
ENSGALT00000032501	
ENSGALT00000006743	
ENSGALT00000018666	TNFAIP2
ENSGALT00000018759	MARK3
ENSGALT00000006863	TTYH3
ENSGALT00000018795	APOPT1
ENSGALT00000037448	
ENSGALT00000037443	
ENSGALT00000019138	BTBD6
ENSGALT00000007098	FOXK1
ENSGALT00000019340	
ENSGALT00000019364	
ENSGALT00000007215	MMD2
ENSGALT00000007252	
ENSGALT00000008219	SWT1
ENSGALT00000019443	PPM1A
ENSGALT00000034001	
ENSGALT00000037347	
ENSGALT00000007417	SMURF1
ENSGALT00000000238	
ENSGALT00000019655	
ENSGALT00000000139	
ENSGALT00000019761	NAA30
ENSGALT00000019771	
ENSGALT00000019806	PELI2
ENSGALT00000007493	BUD31
ENSGALT00000019886	
ENSGALT00000019926	SAMD4A
ENSGALT00000019930	CGRRF1
ENSGALT00000007505	
ENSGALT00000019945	
ENSGALT00000007510	
ENSGALT00000007592	DHRS7B

ENSGALT00000020050	SOS2
ENSGALT00000020133	MAP4K5
ENSGALT00000007690	
ENSGALT00000007721	
ENSGALT00000007745	
ENSGALT00000020162	ATL1
ENSGALT00000007838	
ENSGALT00000020164	SAV1
ENSGALT00000007980	ALKBH5
ENSGALT00000020241	NID2
ENSGALT00000008014	MIEF2
ENSGALT00000008093	SHMT1
ENSGALT00000000149	
ENSGALT00000008119	
ENSGALT00000019575	
ENSGALT00000031517	
ENSGALT00000003794	
ENSGALT00000008468	LMF1
ENSGALT00000040739	LAS1L
ENSGALT00000008614	
ENSGALT00000008671	
ENSGALT00000007313	
ENSGALT00000007294	YIPF6
ENSGALT00000008816	
ENSGALT00000007137	EDA
ENSGALT00000008954	GFER
ENSGALT00000009002	
ENSGALT00000006532	
ENSGALT00000006500	
ENSGALT00000006376	
ENSGALT00000022940	
ENSGALT00000023322	
ENSGALT00000006314	ZDHHC9
ENSGALT00000009467	
ENSGALT00000009734	
ENSGALT00000009868	
ENSGALT00000007708	HDAC8
ENSGALT00000007851	
ENSGALT00000000161	
ENSGALT00000010366	KCTD5
ENSGALT00000007959	TIMM8A
ENSGALT00000010394	

ENSGALT00000010426	
ENSGALT00000010464	KIAA0430
ENSGALT00000010556	
ENSGALT00000008098	
ENSGALT00000008105	
ENSGALT00000010888	
ENSGALT00000008128	
ENSGALT00000010969	
ENSGALT00000011038	SMG1
ENSGALT00000011107	
ENSGALT00000011312	EEF2K
ENSGALT00000011412	
ENSGALT00000009079	FOXO4
ENSGALT00000011481	
ENSGALT00000011534	
ENSGALT00000009239	
ENSGALT00000039834	C16orf72
ENSGALT00000000404	
ENSGALT00000009488	
ENSGALT00000009540	
ENSGALT00000012315	MGRN1
ENSGALT00000012323	UBALD1
ENSGALT00000012346	
ENSGALT00000012358	HMOX2
ENSGALT00000009812	GPC4
ENSGALT00000012459	
ENSGALT00000009861	FAM122B
ENSGALT00000009895	MOSPD1
ENSGALT00000022056	MCOLN1
ENSGALT00000012620	
ENSGALT00000012623	
ENSGALT00000009988	FHL1
ENSGALT00000010348	HTATSF1
ENSGALT00000012696	DNAJA3
ENSGALT00000021945	
ENSGALT00000010117	KIRREL
ENSGALT00000000945	ETV3
ENSGALT00000010487	
ENSGALT00000021621	PRCC
ENSGALT00000004228	DECR2
ENSGALT00000023483	
ENSGALT00000001529	

ENSGALT00000003862	
ENSGALT00000040760	
ENSGALT00000003641	
ENSGALT00000011011	
ENSGALT00000003054	ABCA3
ENSGALT00000011184	
ENSGALT00000022043	TPM3
ENSGALT00000011226	
ENSGALT00000011305	
ENSGALT00000015109	RNPS1
ENSGALT00000015018	
ENSGALT00000015002	
ENSGALT00000032362	
ENSGALT00000023620	
ENSGALT00000017111	
ENSGALT00000001109	
ENSGALT00000003152	GPRC5B
ENSGALT00000019489	
ENSGALT00000032336	NSDHL
ENSGALT00000012141	
ENSGALT00000012247	
ENSGALT00000012279	
ENSGALT00000012370	
ENSGALT00000012409	
ENSGALT00000012454	
ENSGALT00000039451	
ENSGALT00000012569	
ENSGALT00000012638	
ENSGALT00000012629	ABCB7
ENSGALT00000012759	
ENSGALT00000012893	
ENSGALT00000013077	PAK3
ENSGALT00000013221	COL4A5
ENSGALT00000013217	COL4A5
ENSGALT00000013447	COL4A6
ENSGALT00000013485	
ENSGALT00000013668	VBP1
ENSGALT00000013866	
ENSGALT00000013871	
ENSGALT00000013962	
ENSGALT00000014012	TMEM255A
ENSGALT00000014085	

ENSGALT00000014088	
ENSGALT00000039050	
ENSGALT00000014414	FAM199X
ENSGALT00000014716	TBC1D8B
ENSGALT00000014753	
ENSGALT00000014767	CD99L2
ENSGALT00000014782	MTMR1
ENSGALT00000014807	MTM1
ENSGALT00000009805	
ENSGALT00000029631	ATF1
ENSGALT00000009909	DIP2B
ENSGALT00000014951	FMR1
ENSGALT00000015038	
ENSGALT00000015067	PLRG1
ENSGALT00000015084	
ENSGALT00000032288	MAP9
ENSGALT00000015246	GUCY1B3
ENSGALT00000028268	GPD1
ENSGALT00000015275	
ENSGALT00000015333	FAM198B
ENSGALT00000015372	
ENSGALT00000015382	PPID
ENSGALT00000015418	FNIP2
ENSGALT00000009619	
ENSGALT00000015511	TMEM192
ENSGALT00000015566	
ENSGALT00000015714	PALLD
ENSGALT00000015738	C4orf27
ENSGALT00000019115	ARHGAP35
ENSGALT00000015813	CBR4
ENSGALT00000015841	PCDH18
ENSGALT00000015887	
ENSGALT00000015926	
ENSGALT00000016039	RNF150
ENSGALT00000016070	USP38
ENSGALT00000016217	
ENSGALT00000016226	LSM6
ENSGALT00000038572	
ENSGALT00000016263	
ENSGALT00000016278	PRMT10
ENSGALT00000016293	ARHGAP10
ENSGALT00000016385	SH3D19

ENSGALT00000016422	PET112
ENSGALT00000016433	
ENSGALT00000016475	
ENSGALT00000016483	HGSNAT
ENSGALT00000016490	
ENSGALT00000016555	
ENSGALT00000016708	
ENSGALT00000016727	
ENSGALT00000016846	CCNG2
ENSGALT00000016908	
ENSGALT00000017134	ETNPPL
ENSGALT00000017138	
ENSGALT00000025976	
ENSGALT00000017158	PAPSS1
ENSGALT00000017210	INTS12
ENSGALT00000017222	PPA2
ENSGALT00000017251	
ENSGALT00000017263	SNX25
ENSGALT00000017270	
ENSGALT00000017294	
ENSGALT00000017342	RWDD4
ENSGALT00000017346	CDKN2AIP
ENSGALT00000017488	SAP30
ENSGALT00000017505	
ENSGALT00000017617	ASB5
ENSGALT00000017629	VEGFC
ENSGALT00000017688	G3BP2
ENSGALT00000017693	BMP2K
ENSGALT00000017703	ANTXR2
ENSGALT00000017784	NUDT9
ENSGALT00000018144	
ENSGALT00000018225	ENOPH1
ENSGALT00000021658	ARHGEF11
ENSGALT00000029722	RNF41
ENSGALT00000018298	
ENSGALT00000022282	
ENSGALT00000018500	TECRL
ENSGALT00000032173	NOA1
ENSGALT00000001580	
ENSGALT00000018588	
ENSGALT00000041395	LEPROTL1
ENSGALT00000018652	

ENSGALT00000001556	MTMR4
ENSGALT00000002265	NDRG3
ENSGALT00000018681	CCNI
ENSGALT00000002335	
ENSGALT00000018738	SCARB2
ENSGALT00000002384	
ENSGALT00000001603	ELN
ENSGALT00000021722	
ENSGALT00000018860	RUFY3
ENSGALT00000018869	GRSF1
ENSGALT00000001731	AUTS2
ENSGALT00000002775	
ENSGALT00000002799	
ENSGALT00000003157	AHCY
ENSGALT00000001926	GTF2IRD1
ENSGALT00000019202	UBA6
ENSGALT00000002942	MAP1LC3A
ENSGALT00000002964	DYNLRB1
ENSGALT00000040421	RALY
ENSGALT00000004812	PXMP4
ENSGALT00000019309	
ENSGALT00000019311	NUDT6
ENSGALT00000040447	
ENSGALT00000002223	ANKFY1
ENSGALT00000002242	CYB5D2
ENSGALT00000019519	
ENSGALT00000002517	DERL2
ENSGALT00000002525	C1QBP
ENSGALT00000019642	
ENSGALT00000019661	
ENSGALT00000035106	RABL5
ENSGALT00000006265	
ENSGALT00000019851	
ENSGALT00000006537	TOMM34
ENSGALT00000019895	PLA2G12A
ENSGALT00000019897	
ENSGALT00000037662	
ENSGALT00000019968	TSPAN5
ENSGALT00000006588	
ENSGALT00000006631	
ENSGALT00000006710	
ENSGALT00000019988	

ENSGALT00000006749	
ENSGALT00000002944	DTX2
ENSGALT00000040248	
ENSGALT00000003016	MDH2
ENSGALT00000007309	SULF2
ENSGALT00000007499	CSE1L
ENSGALT00000003069	TMEM120A
ENSGALT00000020032	TRMT10A
ENSGALT00000034233	KCNB1
ENSGALT00000000070	
ENSGALT00000003192	
ENSGALT00000028340	
ENSGALT00000020064	EMCN
ENSGALT00000007724	CCNDBP1
ENSGALT00000020119	
ENSGALT00000008180	
ENSGALT00000020161	BDH2
ENSGALT00000008232	LSM14B
ENSGALT00000008294	MTG2
ENSGALT00000008344	
ENSGALT00000041383	
ENSGALT00000023517	
ENSGALT00000003748	
ENSGALT00000009067	
ENSGALT00000022137	
ENSGALT00000003757	
ENSGALT00000009144	
ENSGALT00000003775	
ENSGALT00000003859	
ENSGALT00000022171	
ENSGALT00000009286	
ENSGALT00000009312	
ENSGALT00000022194	VPS37A
ENSGALT00000022198	
ENSGALT00000009385	
ENSGALT00000033928	
ENSGALT00000009494	PCMTD2
ENSGALT00000022308	
ENSGALT00000022312	
ENSGALT00000003944	
ENSGALT00000004011	
ENSGALT00000004087	TPST1

ENSGALT00000004122	
ENSGALT00000009779	ZBTB46
ENSGALT00000004144	TMEM248
ENSGALT00000036310	
ENSGALT00000032094	SRD5A3
ENSGALT00000004146	FAM211A
ENSGALT00000004183	
ENSGALT00000022578	CHIC2
ENSGALT00000004394	PITPNA
ENSGALT00000022602	SCFD2
ENSGALT00000032088	USP46
ENSGALT00000022640	
ENSGALT00000022792	DCUN1D4
ENSGALT00000009996	SLC52A3
ENSGALT00000009999	
ENSGALT00000022799	OCIAD1
ENSGALT00000010129	
ENSGALT00000004762	
ENSGALT00000010607	TM9SF4
ENSGALT00000022997	
ENSGALT00000004849	
ENSGALT00000023042	SLC30A9
ENSGALT00000023043	TMEM33
ENSGALT00000005087	POLDIP2
ENSGALT00000023102	
ENSGALT00000023136	LIAS
ENSGALT00000010897	CDK5RAP1
ENSGALT00000029529	KLHL5
ENSGALT00000039224	
ENSGALT00000010979	
ENSGALT00000003560	
ENSGALT00000003630	
ENSGALT00000003701	
ENSGALT00000003720	
ENSGALT00000021744	PCBP2
ENSGALT00000003781	
ENSGALT00000003954	COL5A2
ENSGALT00000023206	RBPJ
ENSGALT00000023214	SEL1L3
ENSGALT00000004032	
ENSGALT00000004147	
ENSGALT00000006318	RPL23A

ENSGALT00000004253	DUSP19
ENSGALT00000004344	NCKAP1
ENSGALT00000011732	
ENSGALT00000012067	CTSZ
ENSGALT00000023256	SOD3
ENSGALT00000006365	
ENSGALT00000023259	
ENSGALT00000023263	
ENSGALT00000039685	
ENSGALT00000012194	
ENSGALT00000006417	PHF12
ENSGALT00000004363	
ENSGALT00000041120	FSTL3
ENSGALT00000004522	KANSL1L
ENSGALT00000004557	
ENSGALT00000012397	
ENSGALT00000012648	ZNF217
ENSGALT00000023420	
ENSGALT00000023428	C1QTNF7
ENSGALT00000028050	DPM1
ENSGALT00000024191	LYAR
ENSGALT00000038844	
ENSGALT00000013022	
ENSGALT00000006434	NUFIP2
ENSGALT00000024208	
ENSGALT00000005664	
ENSGALT00000004660	TXNL1
ENSGALT00000006045	
ENSGALT00000025037	WFS1
ENSGALT00000006652	NSRP1
ENSGALT00000039716	
ENSGALT00000025045	MAN2B2
ENSGALT00000006250	
ENSGALT00000006838	CPD
ENSGALT00000004384	
ENSGALT00000025066	
ENSGALT00000006274	
ENSGALT00000006341	IQCA1
ENSGALT00000025088	ABLIM2
ENSGALT00000025114	HTRA3
ENSGALT00000025139	ACOX3
ENSGALT00000025174	DOK7

ENSGALT00000006583	SH3BP4
ENSGALT00000006741	TRAF3IP1
ENSGALT00000007018	SPECC1
ENSGALT00000033730	
ENSGALT00000003868	AQP3
ENSGALT00000002555	
ENSGALT00000025262	
ENSGALT00000002382	NT5DC2
ENSGALT00000009315	
ENSGALT00000039669	
ENSGALT00000009887	
ENSGALT00000002508	
ENSGALT00000009335	
ENSGALT00000025335	
ENSGALT00000025338	
ENSGALT00000034480	
ENSGALT00000007195	TRPV2
ENSGALT00000007345	
ENSGALT00000007351	TAX1BP3
ENSGALT00000007373	CTNS
ENSGALT00000002971	DNAJB5
ENSGALT00000007457	AKAP10
ENSGALT00000025439	PTCD3
ENSGALT00000025450	
ENSGALT00000003330	NPRL2
ENSGALT00000003543	
ENSGALT00000003585	MAPKAPK3
ENSGALT00000025484	
ENSGALT00000007525	ULK2
ENSGALT00000003737	
ENSGALT00000012579	TMEFF2
ENSGALT00000025489	
ENSGALT00000012866	
ENSGALT00000007598	
ENSGALT00000007650	NXN
ENSGALT00000013057	COQ10B
ENSGALT00000013137	
ENSGALT00000023128	
ENSGALT00000013192	
ENSGALT00000035848	
ENSGALT00000025715	EIF2AK3
ENSGALT00000013202	

ENSGALT00000032015	
ENSGALT00000003917	RNF123
ENSGALT00000033513	C2orf47
ENSGALT00000007833	FAM101B
ENSGALT00000025773	
ENSGALT00000020313	
ENSGALT00000033496	
ENSGALT00000003976	CREB3
ENSGALT00000013327	
ENSGALT00000004064	
ENSGALT00000008181	GDPD1
ENSGALT00000013409	
ENSGALT00000013633	TRAK2
ENSGALT00000013642	STRADB
ENSGALT00000013654	TMEM237
ENSGALT00000008250	
ENSGALT00000013679	MPP4
ENSGALT00000025829	ATRN
ENSGALT00000034226	
ENSGALT00000006075	TEX264
ENSGALT00000013707	ALS2
ENSGALT00000008348	
ENSGALT00000036280	
ENSGALT00000008405	MED13
ENSGALT00000008456	
ENSGALT00000001250	TMEM173
ENSGALT00000013828	CCNYL1
ENSGALT00000005943	OSMR
ENSGALT00000029609	
ENSGALT00000004187	
ENSGALT00000023947	C7
ENSGALT00000008605	CA4
ENSGALT00000023976	
ENSGALT00000023983	C5orf28
ENSGALT00000000046	
ENSGALT00000007519	ASPN
ENSGALT00000023990	NNT
ENSGALT00000007684	IARS
ENSGALT00000003921	
ENSGALT00000024048	ARL15
ENSGALT00000014015	
ENSGALT00000023719	

ENSGALT00000000221	CDS2
ENSGALT00000003725	
ENSGALT00000003545	ETF1
ENSGALT00000014120	RAPH1
ENSGALT00000023728	
ENSGALT00000007969	TIMP4
ENSGALT00000007976	
ENSGALT00000003489	
ENSGALT00000014220	
ENSGALT00000014333	IDH1
ENSGALT00000014370	PIKFYVE
ENSGALT00000008859	DYNLL2
ENSGALT00000002683	MAT2B
ENSGALT00000002649	NUDCD2
ENSGALT00000002636	CCNG1
ENSGALT00000023799	PPWD1
ENSGALT00000008944	AKAP1
ENSGALT00000008222	FBLN2
ENSGALT00000023839	SREK1
ENSGALT00000002157	ADRA1B
ENSGALT00000008313	FAM120A
ENSGALT00000008368	WNK2
ENSGALT00000001972	CLIP2
ENSGALT00000023888	SERINC5
ENSGALT00000033324	
ENSGALT00000023918	HOMER1
ENSGALT00000007194	
ENSGALT00000007047	
ENSGALT00000008433	BICD2
ENSGALT00000006998	AP3B1
ENSGALT00000014791	
ENSGALT00000038562	F2RL2
ENSGALT00000024118	COL4A3BP
ENSGALT00000022788	CPEB4
ENSGALT00000024097	GFM2
ENSGALT00000024086	HEXB
ENSGALT00000008882	ASB14
ENSGALT00000029777	
ENSGALT00000009143	NLK
ENSGALT00000009146	TMEM97
ENSGALT00000024194	MRPS27
ENSGALT00000001663	GTSF1

ENSGALT00000024183	PGM5
ENSGALT00000023329	AK3
ENSGALT00000009021	ARF4
ENSGALT00000000557	
ENSGALT00000024230	RCL1
ENSGALT00000024242	CD274
ENSGALT00000024250	KIAA1432
ENSGALT00000009368	
ENSGALT00000024268	KIAA2026
ENSGALT00000032836	KDM4C
ENSGALT00000002766	
ENSGALT00000015032	AGPS
ENSGALT00000033290	
ENSGALT00000002628	
ENSGALT00000015075	MTX2
ENSGALT00000009300	UQCRC1
ENSGALT00000009663	MED31
ENSGALT00000015103	KIAA1715
ENSGALT00000000004	
ENSGALT00000015106	
ENSGALT00000038862	FBXO39
ENSGALT00000038228	FXN
ENSGALT00000009792	SMTNL2
ENSGALT00000002495	DUSP26
ENSGALT00000024387	MAMDC2
ENSGALT00000000469	PSMC5
ENSGALT00000024435	
ENSGALT00000015187	
ENSGALT00000015215	
ENSGALT00000021613	
ENSGALT00000000694	
ENSGALT00000032821	
ENSGALT00000015508	
ENSGALT00000032816	NMRK1
ENSGALT00000015535	SLC25A12
ENSGALT00000000541	
ENSGALT00000004898	PROSC
ENSGALT00000009495	RAB7A
ENSGALT00000009501	RPN1
ENSGALT00000000891	DNAJC11
ENSGALT00000020544	FRMD3
ENSGALT00000020546	IDNK

ENSGALT00000030746	
ENSGALT00000009510	EEFSEC
ENSGALT00000009542	
ENSGALT00000020549	
ENSGALT00000005307	NOP16
ENSGALT00000009591	SEC61A1
ENSGALT00000000901	KLHL21
ENSGALT00000005328	
ENSGALT00000009651	
ENSGALT00000005077	EIF4EBP1
ENSGALT00000040847	
ENSGALT00000009723	
ENSGALT00000015660	UBR3
ENSGALT00000020563	
ENSGALT00000009765	
ENSGALT00000005847	RNF145
ENSGALT00000029074	
ENSGALT00000009993	
ENSGALT00000005867	
ENSGALT00000010098	KLF15
ENSGALT00000010142	
ENSGALT00000015749	METTL5
ENSGALT00000020572	
ENSGALT00000023340	CHD5
ENSGALT00000015751	
ENSGALT00000020582	
ENSGALT00000015791	KLHL23
ENSGALT00000010231	
ENSGALT00000000783	CCDC47
ENSGALT00000010255	
ENSGALT00000015996	PPIG
ENSGALT00000020588	
ENSGALT00000020591	
ENSGALT00000001513	TPRG1L
ENSGALT00000016007	
ENSGALT00000006159	MED7
ENSGALT00000006244	MRPL22
ENSGALT00000006374	LARP1
ENSGALT00000010371	
ENSGALT00000010440	CCDC51
ENSGALT00000006432	
ENSGALT00000000842	KANSL1

ENSGALT00000040399	SLC25A39
ENSGALT00000022180	LRRC2
ENSGALT00000006615	
ENSGALT00000035387	
ENSGALT00000001855	
ENSGALT00000021979	SPTLC1
ENSGALT00000006945	CCDC69
ENSGALT00000000584	
ENSGALT00000035182	
ENSGALT00000007522	
ENSGALT00000037896	
ENSGALT00000010572	SRPK3
ENSGALT00000001986	TMEM52
ENSGALT00000010655	MTMR14
ENSGALT00000001955	ZNF652
ENSGALT00000001957	
ENSGALT00000001966	ABI3
ENSGALT00000035169	
ENSGALT00000001975	
ENSGALT00000017724	
ENSGALT00000000332	
ENSGALT00000000365	FBXL17
ENSGALT00000024619	PAM
ENSGALT00000002003	UBE2Z
ENSGALT00000007219	DCTN4
ENSGALT00000002317	CALCOCO2
ENSGALT00000001947	HOXB9
ENSGALT00000005499	
ENSGALT00000005511	
ENSGALT00000000373	
ENSGALT00000007249	
ENSGALT00000028252	
ENSGALT00000007290	NDST1
ENSGALT00000005561	AGPAT6
ENSGALT00000009192	PDGFRB
ENSGALT00000009190	CSF1R
ENSGALT00000005683	
ENSGALT00000024737	
ENSGALT00000036239	
ENSGALT00000024799	
ENSGALT00000002321	VWA1
ENSGALT00000002423	PSMB3

ENSGALT00000018083	
ENSGALT00000002452	PIP4K2B
ENSGALT00000011121	QRICH1
ENSGALT00000009349	RNF130
ENSGALT00000005799	AP3M2
ENSGALT00000036016	
ENSGALT00000009410	TBC1D9B
ENSGALT00000011269	
ENSGALT00000005886	PLAT
ENSGALT00000011358	PRKAR2A
ENSGALT00000024832	
ENSGALT00000024838	
ENSGALT00000037774	
ENSGALT00000029612	VDAC3
ENSGALT00000011505	
ENSGALT00000024902	SLC44A1
ENSGALT00000002573	FBXL20
ENSGALT00000002657	CDK12
ENSGALT00000023556	STARD3
ENSGALT00000002864	UBE2J2
ENSGALT00000011518	KCTD6
ENSGALT00000011555	ACOX2
ENSGALT00000025051	RAD23B
ENSGALT00000002907	
ENSGALT00000023458	KANSL3
ENSGALT00000000352	
ENSGALT00000029130	
ENSGALT00000023663	ERAP1
ENSGALT00000011799	
ENSGALT00000023651	
ENSGALT00000033125	
ENSGALT00000011817	
ENSGALT00000023641	TTC37
ENSGALT00000009699	CNOT6
ENSGALT00000033111	
ENSGALT00000018302	SLC4A3
ENSGALT00000012074	
ENSGALT00000003902	GPR157
ENSGALT00000012394	
ENSGALT00000003951	SPSB1
ENSGALT00000005478	
ENSGALT00000005463	

ENSGALT00000004118	
ENSGALT00000032786	
ENSGALT00000005294	
ENSGALT00000010215	
ENSGALT00000028126	
ENSGALT00000012448	
ENSGALT00000022505	PCYOX1
ENSGALT00000018446	DES
ENSGALT00000034107	TXNDC15
ENSGALT00000010356	SEC24A
ENSGALT00000012660	GXYLT2
ENSGALT00000004300	KIF1B
ENSGALT00000033329	PPP4R2
ENSGALT00000032789	
ENSGALT00000004426	
ENSGALT00000004476	PEX14
ENSGALT00000018477	
ENSGALT00000029510	
ENSGALT00000025065	
ENSGALT00000022896	NUDCD3
ENSGALT00000010437	
ENSGALT00000010461	
ENSGALT00000010508	
ENSGALT00000010517	
ENSGALT00000005284	MTOR
ENSGALT00000005311	
ENSGALT00000005447	PLEKHM2
ENSGALT00000025254	HSDL2
ENSGALT00000025295	UGCG
ENSGALT00000025297	
ENSGALT00000025304	KIAA0368
ENSGALT00000025326	
ENSGALT00000013532	
ENSGALT00000013623	
ENSGALT00000018495	FAM134A
ENSGALT00000004510	RUNDC1
ENSGALT00000002747	PLAA
ENSGALT00000002845	TEK
ENSGALT00000036208	
ENSGALT00000004453	VAT1
ENSGALT00000013700	
ENSGALT00000013715	

ENSGALT00000037566	
ENSGALT00000003135	
ENSGALT00000013770	IFT122
ENSGALT00000013808	PLXND1
ENSGALT00000013831	TMCC1
ENSGALT00000013922	FGD5
ENSGALT00000003386	
ENSGALT00000003423	
ENSGALT00000011833	
ENSGALT00000011880	
ENSGALT00000003696	ATG12
ENSGALT00000011976	
ENSGALT00000034531	
ENSGALT00000003983	MCCC2
ENSGALT00000018650	TMBIM1
ENSGALT00000018675	
ENSGALT00000006425	
ENSGALT00000039619	
ENSGALT00000038163	
ENSGALT00000004909	PINK1
ENSGALT00000018709	SMARCAL1
ENSGALT00000013288	
ENSGALT00000013361	FEM1C
ENSGALT00000018820	
ENSGALT00000018933	PTPN4
ENSGALT00000029784	
ENSGALT00000008572	PPIC
ENSGALT00000019039	NIFK
ENSGALT00000019041	
ENSGALT00000019090	DIRC2
ENSGALT00000019110	PDIA5
ENSGALT00000019216	
ENSGALT00000019286	HEG1
ENSGALT00000007199	MFN2
ENSGALT00000007216	
ENSGALT00000019698	OSBPL11
ENSGALT00000019704	
ENSGALT00000019721	
ENSGALT00000007342	MTHFR
ENSGALT00000032941	
ENSGALT00000007609	C1QB
ENSGALT00000007608	C1QA

ENSGALT00000019870	SLC35F5
ENSGALT00000034897	
ENSGALT00000019931	CCNT2
ENSGALT00000039213	CLDN19
ENSGALT00000007741	
ENSGALT00000007793	PPCS
ENSGALT00000020181	
ENSGALT00000020351	MBD5
ENSGALT00000020382	
ENSGALT00000029274	
ENSGALT00000020391	
ENSGALT00000020417	
ENSGALT00000020434	ARL5A
ENSGALT00000020486	GPD2
ENSGALT00000020497	
ENSGALT00000020547	MARCH7
ENSGALT00000001983	
ENSGALT00000001997	
ENSGALT00000002184	
ENSGALT00000002361	
ENSGALT00000003148	COMT
ENSGALT00000003199	
ENSGALT00000003339	PGAM5
ENSGALT00000003414	CHFR
ENSGALT00000003784	ULK1
ENSGALT00000003883	MMP17
ENSGALT00000034743	
ENSGALT00000004249	SLC15A4
ENSGALT00000004577	
ENSGALT00000004598	
ENSGALT00000004770	SCARB1
ENSGALT00000004933	NCOR2
ENSGALT00000005240	
ENSGALT00000005393	
ENSGALT00000006066	ANAPC7
ENSGALT00000026506	HSF1
ENSGALT00000007010	MLXIP
ENSGALT00000000380	HMBS
ENSGALT00000007031	DIABLO
ENSGALT00000000393	VPS11
ENSGALT00000000417	SIK2
ENSGALT00000007244	PPTC7

ENSGALT00000007277	
ENSGALT00000035085	
ENSGALT00000001383	PANX3
ENSGALT00000000486	
ENSGALT00000000525	DHDDS
ENSGALT00000001501	
ENSGALT00000007523	
ENSGALT00000001579	
ENSGALT00000000760	CNKSR1
ENSGALT00000000781	LAPTM5
ENSGALT00000001710	
ENSGALT00000001717	
ENSGALT00000001790	KCNJ5
ENSGALT00000000876	
ENSGALT00000001897	
ENSGALT00000000878	
ENSGALT00000038999	
ENSGALT00000000957	CTPS1
ENSGALT00000001016	
ENSGALT00000002056	
ENSGALT00000001026	STX12
ENSGALT00000001032	
ENSGALT00000023447	HECTD4
ENSGALT00000001127	
ENSGALT00000002230	JAM3
ENSGALT00000002312	VPS26B
ENSGALT00000002324	
ENSGALT00000002439	
ENSGALT00000002903	
ENSGALT00000001253	ZMPSTE24
ENSGALT00000003079	LDB3
ENSGALT00000003144	
ENSGALT00000003257	
ENSGALT00000007773	ISCU
ENSGALT00000001788	
ENSGALT00000001834	PPP2R3A
ENSGALT00000001317	
ENSGALT00000041155	FAM46B
ENSGALT00000001392	WDTC1
ENSGALT00000001847	PCCB
ENSGALT00000010780	ARHGEF12
ENSGALT00000034383	

ENSGALT00000010901	PVRL1
ENSGALT00000003908	
ENSGALT00000040822	
ENSGALT00000004014	CSGALNACT2
ENSGALT00000034212	
ENSGALT00000004154	
ENSGALT00000041354	
ENSGALT00000034167	
ENSGALT00000004265	
ENSGALT00000001474	MTFR1L
ENSGALT00000040809	
ENSGALT00000001481	
ENSGALT00000011217	
ENSGALT00000001539	MAN1C1
ENSGALT00000004359	
ENSGALT00000011363	
ENSGALT00000001870	
ENSGALT00000023514	CADM1
ENSGALT00000001916	CLIC4
ENSGALT00000001994	TRNAU1AP
ENSGALT00000004680	
ENSGALT00000011570	
ENSGALT00000004876	
ENSGALT00000002149	SRSF4
ENSGALT00000005033	
ENSGALT00000034008	
ENSGALT00000011831	CEP164
ENSGALT00000008853	ADRBK2
ENSGALT00000002306	FHL3
ENSGALT00000003103	LIMS2
ENSGALT00000003200	
ENSGALT00000011985	SCN4B
ENSGALT00000034294	SCN2B
ENSGALT00000003399	
ENSGALT00000011998	
ENSGALT00000000544	
ENSGALT00000010877	
ENSGALT00000012507	SLC37A4
ENSGALT00000005757	PTEN
ENSGALT00000005787	ATAD1
ENSGALT00000006268	
ENSGALT00000009327	KREMEN1

ENSGALT00000006467	SLC25A16
ENSGALT00000006533	CCAR1
ENSGALT00000009461	DGCR14
ENSGALT00000009474	DGCR2
ENSGALT00000006608	
ENSGALT00000036018	EVA1B
ENSGALT00000010597	DNAJB11
ENSGALT00000006635	VPS26A
ENSGALT00000012830	DLAT
ENSGALT00000003587	
ENSGALT00000012907	
ENSGALT00000040712	
ENSGALT00000001980	
ENSGALT00000009622	
ENSGALT00000006946	MCU
ENSGALT00000007067	PPA1
ENSGALT00000010418	
ENSGALT00000010254	
ENSGALT00000010325	WDR53
ENSGALT00000010258	D2HGDH
ENSGALT00000007605	
ENSGALT00000007678	
ENSGALT00000007743	ZMIZ1
ENSGALT00000010319	CRKL
ENSGALT00000010150	THAP4
ENSGALT00000010118	KLHL30
ENSGALT00000005168	PTP4A2
ENSGALT00000010647	
ENSGALT00000010666	GUCD1
ENSGALT00000005249	
ENSGALT00000005281	
ENSGALT00000008009	COMTD1
ENSGALT00000008018	
ENSGALT00000008036	
ENSGALT00000008109	
ENSGALT00000008126	
ENSGALT00000021437	
ENSGALT00000008159	CAMK2G
ENSGALT00000009365	
ENSGALT00000009355	
ENSGALT00000008188	
ENSGALT00000005575	

ENSGALT00000008280	
ENSGALT00000035005	
ENSGALT00000008299	
ENSGALT00000005662	RNF19B
ENSGALT00000010902	
ENSGALT00000005857	
ENSGALT00000008397	MYOZ1
ENSGALT00000008603	SORBS1
ENSGALT00000008903	
ENSGALT00000006028	PABPC4
ENSGALT00000008618	MRPS22
ENSGALT00000008877	TM9SF3
ENSGALT00000011250	PIK3IP1
ENSGALT00000008940	NOLC1
ENSGALT00000009008	PITX3
ENSGALT00000009044	GBF1
ENSGALT00000011276	
ENSGALT00000011283	C12orf43
ENSGALT00000009122	CUEDC2
ENSGALT00000006228	CAP1
ENSGALT00000003064	PRPF38B
ENSGALT00000009141	HIF1AN
ENSGALT00000003258	GPSM2
ENSGALT00000011502	MLEC
ENSGALT00000008412	
ENSGALT00000008360	ACSL3
ENSGALT00000003286	CLCC1
ENSGALT00000011568	RNF10
ENSGALT00000003326	WDR47
ENSGALT00000006375	
ENSGALT00000008282	
ENSGALT00000011588	
ENSGALT00000008246	
ENSGALT00000008191	CUL3
ENSGALT00000006539	FUCA1
ENSGALT00000008057	DOCK10
ENSGALT00000003625	ZBTB41
ENSGALT00000011731	
ENSGALT00000009862	MAPK8
ENSGALT00000003831	
ENSGALT00000003897	
ENSGALT00000004005	

ENSGALT00000004201	OLFML2B
ENSGALT00000004312	UHMK1
ENSGALT00000010304	LIPA
ENSGALT00000004870	MFF
ENSGALT00000011828	
ENSGALT00000004824	AGFG1
ENSGALT00000010382	PANK1
ENSGALT00000004623	TRIP12
ENSGALT00000033697	
ENSGALT00000011938	HSPB8
ENSGALT00000010542	NOC3L
ENSGALT00000004729	PHGDH
ENSGALT00000011975	
ENSGALT00000033985	
ENSGALT00000004307	
ENSGALT00000004266	XRN1
ENSGALT00000010939	
ENSGALT00000011152	
ENSGALT00000011201	CPEB3
ENSGALT00000011235	BTAF1
ENSGALT00000004131	PCOLCE2
ENSGALT00000003699	C3orf58
ENSGALT00000012100	
ENSGALT00000005458	
ENSGALT00000012892	PNPT1
ENSGALT00000012835	EFEMP1
ENSGALT00000011008	
ENSGALT00000005530	ALDH9A1
ENSGALT00000012531	PRODH
ENSGALT00000012708	
ENSGALT00000005721	DARS2
ENSGALT00000012192	ANKRD2
ENSGALT00000005829	
ENSGALT00000011075	
ENSGALT00000006190	XPR1
ENSGALT00000011195	DLG1
ENSGALT00000011255	
ENSGALT00000006981	
ENSGALT00000039466	
ENSGALT00000013047	
ENSGALT00000006306	CEP350
ENSGALT00000013063	MTMR3

ENSGALT00000013195	
ENSGALT00000006691	
ENSGALT00000038026	
ENSGALT00000033698	
ENSGALT00000006824	
ENSGALT00000013265	
ENSGALT00000011583	
ENSGALT00000021800	CHPF2
ENSGALT00000012505	FBXW4
ENSGALT00000021776	ASB10
ENSGALT00000011646	HRASLS
ENSGALT00000007170	RFWD2
ENSGALT00000013435	ANAPC1
ENSGALT00000011822	
ENSGALT00000007248	CACYBP
ENSGALT00000011898	
ENSGALT00000012719	KAZALD1
ENSGALT00000039302	SFXN3
ENSGALT00000007329	HEBP2
ENSGALT00000028328	
ENSGALT00000039413	
ENSGALT00000012083	PCYT1A
ENSGALT00000013379	
ENSGALT00000013406	MED13L
ENSGALT00000001591	
ENSGALT00000013781	RIN2
ENSGALT00000013021	FAM178A
ENSGALT00000013124	
ENSGALT00000013893	
ENSGALT00000007904	MAP4
ENSGALT00000013911	KLHDC3
ENSGALT00000013919	
ENSGALT00000031983	KLC4
ENSGALT00000033576	C1orf21
ENSGALT00000013188	
ENSGALT00000031981	
ENSGALT00000008265	
ENSGALT00000008354	OLFM3
ENSGALT00000014784	
ENSGALT00000008401	
ENSGALT00000033492	
ENSGALT00000008484	KLHL40

ENSGALT00000008494	
ENSGALT00000008441	VCAM1
ENSGALT00000008467	
ENSGALT00000008474	LRRC39
ENSGALT00000033254	PDE6D
ENSGALT00000012571	COPS7B
ENSGALT00000014742	
ENSGALT00000014738	
ENSGALT00000008548	
ENSGALT00000008549	HIAT1
ENSGALT00000008683	AGL
ENSGALT00000014682	CLIP4
ENSGALT00000039166	
ENSGALT00000014608	TRMT61B
ENSGALT00000008744	
ENSGALT00000008906	PTBP2
ENSGALT00000014542	FBXO11
ENSGALT00000008961	ALG14
ENSGALT00000014390	
ENSGALT00000039077	CNN3
ENSGALT00000014386	
ENSGALT00000009113	
ENSGALT00000008983	TBRG4
ENSGALT00000039079	
ENSGALT00000014275	SPRED2
ENSGALT00000012824	
ENSGALT00000009078	
ENSGALT00000014299	GPAM
ENSGALT00000009422	
ENSGALT00000014409	ZDHHC6
ENSGALT00000014431	
ENSGALT00000014249	
ENSGALT00000014245	
ENSGALT00000031978	FBXO48
ENSGALT00000009287	
ENSGALT00000031979	
ENSGALT00000009337	BCAR3
ENSGALT00000014214	PCSK2
ENSGALT00000014777	
ENSGALT00000038916	
ENSGALT00000009713	
ENSGALT00000014731	

ENSGALT00000009417	
ENSGALT00000014689	BTBD3
ENSGALT00000014663	
ENSGALT00000009497	
ENSGALT00000009966	XYLB
ENSGALT00000014324	
ENSGALT00000014317	
ENSGALT00000014238	
ENSGALT00000013645	EIF2B5
ENSGALT00000013739	
ENSGALT00000013818	ABCF3
ENSGALT00000014091	
ENSGALT00000033354	
ENSGALT00000010164	
ENSGALT00000014035	
ENSGALT00000009984	CCBL2
ENSGALT00000015262	PRDX3
ENSGALT00000028082	
ENSGALT00000010087	PKN2
ENSGALT00000033333	
ENSGALT00000010099	
ENSGALT00000010388	NOM1
ENSGALT00000010467	
ENSGALT00000010570	ESYT2
ENSGALT00000015412	SEC23IP
ENSGALT00000015447	WDR11
ENSGALT00000010623	
ENSGALT00000033012	
ENSGALT00000014128	SYDE2
ENSGALT00000013868	ATL2
ENSGALT00000010875	
ENSGALT00000013852	HNRNPLL
ENSGALT00000011472	
ENSGALT00000038945	
ENSGALT00000011564	
ENSGALT00000014381	TTLL7
ENSGALT00000013878	EIF4G1
ENSGALT00000011573	
ENSGALT00000014453	ELTD1
ENSGALT00000015724	
ENSGALT00000001098	TBC1D22B
ENSGALT00000014497	DNAJB4

ENSGALT00000015848	
ENSGALT00000010681	BCCIP
ENSGALT00000014667	
ENSGALT00000001035	CACNA1S
ENSGALT00000040899	
ENSGALT00000021600	SOS1
ENSGALT00000011888	MTPAP
ENSGALT00000011932	WAC
ENSGALT00000011983	MPP7
ENSGALT00000012033	
ENSGALT00000012112	
ENSGALT00000016231	
ENSGALT00000004009	
ENSGALT00000014863	
ENSGALT00000016376	KDM4A
ENSGALT00000014998	CRLS1
ENSGALT00000017035	
ENSGALT00000015001	
ENSGALT00000012598	KIAA1217
ENSGALT00000012794	
ENSGALT00000012825	COMMD3
ENSGALT00000038548	ST3GAL3
ENSGALT00000000542	IPO9
ENSGALT00000017059	BNIP3
ENSGALT00000015061	SDE2
ENSGALT00000017087	INPP5A
ENSGALT00000012901	
ENSGALT00000012922	PLXDC2
ENSGALT00000013939	
ENSGALT00000014060	
ENSGALT00000003690	ZNF609
ENSGALT00000003455	SNX22
ENSGALT00000015194	
ENSGALT00000014097	STAM
ENSGALT00000014104	PTPLA
ENSGALT00000014117	
ENSGALT00000016418	
ENSGALT00000015319	DISP1
ENSGALT00000015332	BROX
ENSGALT00000014205	PTER
ENSGALT00000014135	
ENSGALT00000014256	FAM171A1

ENSGALT00000015385	
ENSGALT00000014327	CROT
ENSGALT00000015441	MARK1
ENSGALT00000002999	
ENSGALT00000000195	
ENSGALT00000031945	TGFB2
ENSGALT00000033152	
ENSGALT00000015752	KCTD3
ENSGALT00000015924	
ENSGALT00000031937	RPS6KC1
ENSGALT00000015943	
ENSGALT00000031934	VASH2
ENSGALT00000016648	
ENSGALT00000015969	NENF
ENSGALT00000015970	TMEM206
ENSGALT00000015980	PPP2R5A
ENSGALT00000014487	
ENSGALT00000022855	COX5A
ENSGALT00000016009	
ENSGALT00000002612	MPI
ENSGALT00000016037	
ENSGALT00000016049	RCOR3
ENSGALT00000015304	ANKIB1
ENSGALT00000002583	SCAMP2
ENSGALT00000014768	
ENSGALT00000016717	TMEM69
ENSGALT00000016788	MAST2
ENSGALT00000000779	PI16
ENSGALT00000016077	
ENSGALT00000015329	PEX1
ENSGALT00000015427	
ENSGALT00000016989	FAAH
ENSGALT00000000864	PPP1R15B
ENSGALT00000016111	UBR2
ENSGALT00000015485	TFPI2
ENSGALT00000028081	PKDCC
ENSGALT00000015498	
ENSGALT00000041398	
ENSGALT00000014612	GNB4
ENSGALT00000017008	
ENSGALT00000000908	MDM4
ENSGALT00000017068	

ENSGALT00000002511	SIN3A
ENSGALT00000016201	LRPPRC
ENSGALT00000004220	CSPG4
ENSGALT00000014937	NCEH1
ENSGALT00000031920	
ENSGALT00000004357	
ENSGALT00000004380	TSPAN3
ENSGALT00000000950	TMCC2
ENSGALT00000038337	
ENSGALT00000016234	SRBD1
ENSGALT00000004485	RCN2
ENSGALT00000004731	UBE2Q2
ENSGALT00000000994	ELK4
ENSGALT00000016253	
ENSGALT00000001018	SLC41A1
ENSGALT00000017199	TXNDC12
ENSGALT00000017200	
ENSGALT00000016264	
ENSGALT00000017269	ZFYVE9
ENSGALT00000016269	
ENSGALT00000016270	
ENSGALT00000015414	
ENSGALT00000015416	
ENSGALT00000031908	
ENSGALT00000015429	
ENSGALT00000016306	
ENSGALT00000016321	RBKS
ENSGALT00000015740	SGCE
ENSGALT00000001255	
ENSGALT00000017302	
ENSGALT00000017321	ECHDC2
ENSGALT00000001297	MAPKAPK2
ENSGALT00000016469	GLO1
ENSGALT00000015772	ASB4
ENSGALT00000015790	
ENSGALT00000017326	SCP2
ENSGALT00000034634	
ENSGALT00000040356	
ENSGALT00000005141	DNAJA4
ENSGALT00000016480	ZFAND3
ENSGALT00000005233	
ENSGALT00000039672	

ENSGALT00000015869	
ENSGALT00000005378	FBXL22
ENSGALT00000017402	MIOS
ENSGALT00000016499	CMTR1
ENSGALT00000017329	PODN
ENSGALT00000017404	RPA3
ENSGALT00000037997	RPA3-AS1
ENSGALT00000001786	CD34
ENSGALT00000015516	NMD3
ENSGALT00000015523	PPM1L
ENSGALT00000015544	
ENSGALT00000039192	
ENSGALT00000038176	RNF8
ENSGALT00000015632	
ENSGALT00000015634	
ENSGALT00000016540	SLC35B2
ENSGALT00000015688	GFM1
ENSGALT00000038145	
ENSGALT00000017449	
ENSGALT00000039093	MLF1
ENSGALT00000016725	TMEM63B
ENSGALT00000017469	NDC1
ENSGALT00000017477	
ENSGALT00000002207	ATP5F1
ENSGALT00000002238	RAP1A
ENSGALT00000032981	C3orf55
ENSGALT00000017480	LRRC42
ENSGALT00000017484	
ENSGALT00000017487	
ENSGALT00000016736	MRPL14
ENSGALT00000017745	RAPGEF5
ENSGALT00000017540	ACOT11
ENSGALT00000016751	
ENSGALT00000002661	LRIG2
ENSGALT00000017560	
ENSGALT00000006011	BNIP2
ENSGALT00000017568	USP24
ENSGALT00000017589	
ENSGALT00000016730	
ENSGALT00000016761	SLC33A1
ENSGALT00000003002	HIPK1
ENSGALT00000017683	FGGY

ENSGALT00000017829	MALSU1
ENSGALT00000016815	
ENSGALT00000017877	
ENSGALT00000017882	CCDC126
ENSGALT00000034521	MCEE
ENSGALT00000017975	
ENSGALT00000006347	TARSL2
ENSGALT00000017979	
ENSGALT00000032964	TM2D1
ENSGALT00000018006	
ENSGALT00000016864	
ENSGALT00000018034	
ENSGALT00000018085	
ENSGALT00000017909	EFCAB7
ENSGALT00000018131	
ENSGALT00000018199	CHN2
ENSGALT00000017953	
ENSGALT00000003247	
ENSGALT00000018212	WIPF3
ENSGALT00000018231	FKBP14
ENSGALT00000018282	
ENSGALT00000034417	
ENSGALT00000018291	
ENSGALT00000018312	ANKRD28
ENSGALT00000018327	DPH3
ENSGALT00000016977	ZNF318
ENSGALT00000016982	
ENSGALT00000018329	
ENSGALT00000018331	
ENSGALT00000017995	
ENSGALT00000040028	
ENSGALT00000040026	
ENSGALT00000018348	PLCL2
ENSGALT00000018357	TBC1D5
ENSGALT00000018402	KAT2B
ENSGALT00000001028	RFX7
ENSGALT00000017165	
ENSGALT00000038194	
ENSGALT00000018407	ZNF385D
ENSGALT00000007059	
ENSGALT00000018418	NKIRAS1
ENSGALT00000016952	WWTR1

ENSGALT00000007264	
ENSGALT00000018328	
ENSGALT00000018364	
ENSGALT00000018434	
ENSGALT00000018443	
ENSGALT00000018455	ANKRD13C
ENSGALT00000016995	HPS3
ENSGALT00000017002	
ENSGALT00000017274	QPCT
ENSGALT00000017280	
ENSGALT00000017288	
ENSGALT00000018456	OXSM
ENSGALT00000018458	LRRC3B
ENSGALT00000017328	TFB2M
ENSGALT00000004387	
ENSGALT00000018638	AZI2
ENSGALT00000017370	
ENSGALT00000017414	ZBTB18
ENSGALT00000018718	GPD1L
ENSGALT00000017618	ERO1LB
ENSGALT00000017890	NID1
ENSGALT00000018736	TCAIM
ENSGALT00000004102	GGPS1
ENSGALT00000000385	ARID4B
ENSGALT00000007734	SEMA6D
ENSGALT00000018770	ANO10
ENSGALT00000022567	TOMM20
ENSGALT00000018780	
ENSGALT00000018812	EAF1
ENSGALT00000017944	
ENSGALT00000018839	
ENSGALT00000018846	CAPN7
ENSGALT00000018026	
ENSGALT00000018064	
ENSGALT00000034280	SECISBP2L
ENSGALT00000018081	TAF5L
ENSGALT00000005108	APOBEC2
ENSGALT00000005111	
ENSGALT00000018903	ATP2C1
ENSGALT00000018940	
ENSGALT00000018217	
ENSGALT00000018251	

ENSGALT00000019087	DNAJC13
ENSGALT00000018269	SMOC2
ENSGALT00000009244	
ENSGALT00000005500	
ENSGALT00000018633	FGFR1OP
ENSGALT00000018720	
ENSGALT00000018857	PARK2
ENSGALT00000018893	MAP3K4
ENSGALT00000009637	PDE8A
ENSGALT00000009646	FSD2
ENSGALT00000009670	
ENSGALT00000009692	
ENSGALT00000009702	
ENSGALT00000019010	
ENSGALT00000005617	PRELP
ENSGALT00000019329	SACM1L
ENSGALT00000010337	
ENSGALT00000019061	WTAP
ENSGALT00000019062	
ENSGALT00000019065	FNDC1
ENSGALT00000031806	
ENSGALT00000020066	FBXO30
ENSGALT00000019393	ZDHHC3
ENSGALT00000020182	TAB2
ENSGALT00000020197	
ENSGALT00000010523	
ENSGALT00000010528	
ENSGALT00000020225	NUP43
ENSGALT00000037591	
ENSGALT00000031799	LRP11
ENSGALT00000020285	MTHFD1L
ENSGALT00000021152	
ENSGALT00000019473	TRAK1
ENSGALT00000019478	EIF1B
ENSGALT00000022168	
ENSGALT00000022182	SCAF8
ENSGALT00000019495	
ENSGALT00000022259	TMEM242
ENSGALT00000022265	ZDHHC14
ENSGALT00000019569	
ENSGALT00000022311	SYNJ2
ENSGALT00000031788	DYNLT1

ENSGALT00000010834	
ENSGALT00000022346	PLAGL1
ENSGALT00000022358	LTV1
ENSGALT00000019702	MLH1
ENSGALT00000022374	PEX3
ENSGALT00000022379	AIG1
ENSGALT00000022415	VTA1
ENSGALT00000019716	EPDR1
ENSGALT00000011156	FAM174B
ENSGALT00000011260	CHD2
ENSGALT00000022522	MAP3K5
ENSGALT00000019843	HERPUD2
ENSGALT00000022595	PDE7B
ENSGALT00000022607	AHI1
ENSGALT00000022632	
ENSGALT00000019858	
ENSGALT00000022653	SLC2A12
ENSGALT00000022662	EYA4
ENSGALT00000019894	
ENSGALT00000022671	
ENSGALT00000019908	
ENSGALT00000037476	
ENSGALT00000004596	
ENSGALT00000031116	RP9
ENSGALT00000019924	KBTBD2
ENSGALT00000019935	LSM5
ENSGALT00000022702	ARHGAP18
ENSGALT00000022704	LAMA2
ENSGALT00000031751	KIAA0408
ENSGALT00000023965	TRDN
ENSGALT00000023989	
ENSGALT00000020113	YAE1D1
ENSGALT00000011601	
ENSGALT00000011612	
ENSGALT00000020152	C7orf25
ENSGALT00000031107	
ENSGALT00000020177	
ENSGALT00000020178	BLVRA
ENSGALT00000024033	NUS1
ENSGALT00000031104	VOPP1
ENSGALT00000020187	LANCL2
ENSGALT00000037413	

ENSGALT00000024042	DCBLD1
ENSGALT00000020200	TPK1
ENSGALT00000024059	
ENSGALT00000020295	
ENSGALT00000011754	
ENSGALT00000020338	
ENSGALT00000020352	
ENSGALT00000024093	KPNA5
ENSGALT00000031721	
ENSGALT00000024128	
ENSGALT00000024132	NT5DC1
ENSGALT00000020597	COL15A1
ENSGALT00000024267	
ENSGALT00000011900	CILP
ENSGALT00000020646	
ENSGALT00000012036	DPP8
ENSGALT00000020648	NFATC1
ENSGALT00000020670	ALDH5A1
ENSGALT00000020676	MRS2
ENSGALT00000020688	CDKAL1
ENSGALT00000024687	FOXO3
ENSGALT00000024698	LACE1
ENSGALT00000024702	
ENSGALT00000012111	
ENSGALT00000024713	
ENSGALT00000024718	BEND3
ENSGALT00000024723	
ENSGALT00000012122	DENND4A
ENSGALT00000024849	
ENSGALT00000037256	
ENSGALT00000024935	
ENSGALT00000024942	TSTD1
ENSGALT00000020725	RANBP9
ENSGALT00000012312	
ENSGALT00000012462	
ENSGALT00000031683	
ENSGALT00000025147	MAP3K7
ENSGALT00000020743	
ENSGALT00000020760	CAP2
ENSGALT00000025442	
ENSGALT00000020851	
ENSGALT00000020852	

ENSGALT00000020859	
ENSGALT00000020862	BMP6
ENSGALT00000025446	PM20D2
ENSGALT00000025464	
ENSGALT00000031045	
ENSGALT00000020881	
ENSGALT00000020885	
ENSGALT00000020890	FARS2
ENSGALT00000020897	ECI2
ENSGALT00000025551	PRSS35
ENSGALT00000020905	PXDC1
ENSGALT00000020909	SLC22A23
ENSGALT00000025575	IBTK
ENSGALT00000020914	
ENSGALT00000020918	BPHL
ENSGALT00000020945	GMDS
ENSGALT00000020983	
ENSGALT00000025624	SENP6
ENSGALT00000025629	
ENSGALT00000012959	
ENSGALT00000021151	FAM105B
ENSGALT00000021178	TRIO
ENSGALT00000021258	MARCH6
ENSGALT00000021280	SBK2
ENSGALT00000021302	SEMA5A
ENSGALT00000026237	
ENSGALT00000021306	MTRR
ENSGALT00000026251	ZNF451
ENSGALT00000021320	ADCY2
ENSGALT00000021443	
ENSGALT00000026298	MLIP
ENSGALT00000026310	KLHL31
ENSGALT00000021488	
ENSGALT00000026320	GCLC
ENSGALT00000021491	
ENSGALT00000026321	
ENSGALT00000021505	
ENSGALT00000026329	
ENSGALT00000026335	
ENSGALT00000021546	
ENSGALT00000021552	NDUFS6
ENSGALT00000026340	AGPAT5

ENSGALT00000026360	
ENSGALT00000021558	GALNT12
ENSGALT00000031625	
ENSGALT00000026395	
ENSGALT00000013352	
ENSGALT00000013365	
ENSGALT00000026413	PXDN
ENSGALT00000021854	PDCD6
ENSGALT00000021930	ERP44
ENSGALT00000026450	
ENSGALT00000015891	
ENSGALT00000022087	NR4A3
ENSGALT00000022115	BLOC1S4
ENSGALT00000022165	
ENSGALT00000026493	CPSF3
ENSGALT00000015890	
ENSGALT00000022178	ACOT13
ENSGALT00000022206	ZNF236
ENSGALT00000026523	
ENSGALT00000026524	
ENSGALT00000022232	TSHZ1
ENSGALT00000036936	ZADH2
ENSGALT00000013464	TMED3
ENSGALT00000022289	
ENSGALT00000021988	PPP6R2
ENSGALT00000020337	SBF1
ENSGALT00000022290	TIMM21
ENSGALT00000040414	
ENSGALT00000025383	
ENSGALT00000022349	TMX3
ENSGALT00000026538	PDIA6
ENSGALT00000022432	TNPO3
ENSGALT00000013005	KLHDC10
ENSGALT00000033574	
ENSGALT00000022519	RNMT
ENSGALT00000022517	
ENSGALT00000022497	SEH1L
ENSGALT00000013037	TMEM209
ENSGALT00000013516	
ENSGALT00000022485	PSMG2
ENSGALT00000033572	
ENSGALT00000022445	

ENSGALT00000031591	RDH14
ENSGALT00000026589	SDC1
ENSGALT00000029656	
ENSGALT00000028442	
ENSGALT00000013703	PPP4R1
ENSGALT00000013581	
ENSGALT00000013684	
ENSGALT00000013683	ANKRD12
ENSGALT00000026614	
ENSGALT00000023134	NDUFV2
ENSGALT00000031579	C2orf44
ENSGALT00000026617	
ENSGALT00000013589	
ENSGALT00000010489	CHCHD3
ENSGALT00000014003	IQGAP1
ENSGALT00000011162	ITIH5
ENSGALT00000010913	
ENSGALT00000036879	
ENSGALT00000026632	FNDC4
ENSGALT00000010889	
ENSGALT00000010817	UPF2
ENSGALT00000005822	DHTKD1
ENSGALT00000022642	SEC61A2
ENSGALT00000023489	PHYH
ENSGALT00000023921	EMILIN2
ENSGALT00000029187	
ENSGALT00000036791	
ENSGALT00000026684	
ENSGALT00000010596	
ENSGALT00000026714	HADHB
ENSGALT00000026723	
ENSGALT00000039749	
ENSGALT00000023980	
ENSGALT00000026763	SCARA5
ENSGALT00000013557	
ENSGALT00000013555	RSBN1L
ENSGALT00000013546	PTPN12
ENSGALT00000013522	
ENSGALT00000013515	FAM185A
ENSGALT00000013512	
ENSGALT00000013482	
ENSGALT00000013473	

ENSGALT00000013428	
ENSGALT00000013382	
ENSGALT00000024064	USP14
ENSGALT00000013290	
ENSGALT00000013245	SRPK2
ENSGALT00000013172	ATXN7L1
ENSGALT00000026845	EXTL3
ENSGALT00000026848	
ENSGALT00000013062	
ENSGALT00000039460	DUS4L
ENSGALT00000026878	
ENSGALT00000013000	
ENSGALT00000024095	ABHD3
ENSGALT00000024152	MIB1
ENSGALT00000026894	
ENSGALT00000031549	
ENSGALT00000012832	LAMB1
ENSGALT00000026905	TRAM2
ENSGALT00000024186	CABLES1
ENSGALT00000024197	RIOK3
ENSGALT00000011632	
ENSGALT00000026909	
ENSGALT00000024206	C18orf8
ENSGALT00000011493	KIF21A
ENSGALT00000024240	NPC1
ENSGALT00000026965	RCAN2
ENSGALT00000013914	
ENSGALT00000013929	
ENSGALT00000027001	
ENSGALT00000024335	OSBPL1A
ENSGALT00000021457	PLXNB2
ENSGALT00000024382	ZNF521
ENSGALT00000024398	
ENSGALT00000014328	ASB15
ENSGALT00000014337	
ENSGALT00000014340	IQUB
ENSGALT00000014357	
ENSGALT00000014559	AASS
ENSGALT00000014601	
ENSGALT00000014649	CPED1
ENSGALT00000015281	
ENSGALT00000015293	

ENSGALT00000024463	TRAPPC8
ENSGALT00000015373	TMEM168
ENSGALT00000030698	LSMEM1
ENSGALT00000015454	
ENSGALT00000015456	
ENSGALT00000015471	
ENSGALT00000015558	PRICKLE1
ENSGALT00000015593	ADAMTS20
ENSGALT00000015636	TMEM117
ENSGALT00000015735	ARID2
ENSGALT00000015837	
ENSGALT00000024545	DTNA
ENSGALT00000015861	
ENSGALT00000015878	
ENSGALT00000015976	
ENSGALT00000015981	RPL18A
ENSGALT00000015988	
ENSGALT00000016002	
ENSGALT00000016025	
ENSGALT00000016059	
ENSGALT00000016099	CAND1
ENSGALT00000016139	
ENSGALT00000016170	
ENSGALT00000021064	MCM4
ENSGALT00000016330	PTPRB
ENSGALT00000041400	C8orf22
ENSGALT00000016553	RAB21
ENSGALT00000024605	
ENSGALT00000016566	
ENSGALT00000022162	
ENSGALT00000024757	
ENSGALT00000029932	
ENSGALT00000024867	
ENSGALT00000024871	PENK
ENSGALT00000024873	IMPAD1
ENSGALT00000024899	
ENSGALT00000018268	
ENSGALT00000018392	
ENSGALT00000018400	
ENSGALT00000018413	
ENSGALT00000030642	
ENSGALT00000024998	GGH

ENSGALT00000018472	TMCC3
ENSGALT00000018479	
ENSGALT00000022941	ADHFE1
ENSGALT00000018639	
ENSGALT00000025029	
ENSGALT00000018679	CDK17
ENSGALT00000018716	
ENSGALT00000038256	
ENSGALT00000023424	
ENSGALT00000025048	CSPP1
ENSGALT00000018870	
ENSGALT00000025181	
ENSGALT00000030877	
ENSGALT00000019040	
ENSGALT00000019063	NUP205
ENSGALT00000025372	
ENSGALT00000025427	
ENSGALT00000025468	
ENSGALT00000019173	
ENSGALT00000030620	
ENSGALT00000025563	
ENSGALT00000030617	
ENSGALT00000019356	
ENSGALT00000019412	
ENSGALT00000025647	DECR1
ENSGALT00000019461	NHP2L1
ENSGALT00000019464	DESI1
ENSGALT00000019494	
ENSGALT00000019497	
ENSGALT00000037963	
ENSGALT00000025676	
ENSGALT00000025692	
ENSGALT00000025711	
ENSGALT00000019589	XPNPEP3
ENSGALT00000025714	RNF151
ENSGALT00000025742	DPY19L4
ENSGALT00000030837	NDUFAF6
ENSGALT00000025772	
ENSGALT00000025777	
ENSGALT00000023439	MATN2
ENSGALT00000030604	
ENSGALT00000019954	

ENSGALT00000030825	POLR2K
ENSGALT00000025842	RNF19A
ENSGALT00000023333	
ENSGALT00000020100	ANKRD54
ENSGALT00000025883	ATP6V1C1
ENSGALT00000020453	
ENSGALT00000025898	
ENSGALT00000020490	
ENSGALT00000037765	
ENSGALT00000020504	HMGXB4
ENSGALT00000025906	LRP12
ENSGALT00000025913	ABRA
ENSGALT00000025930	
ENSGALT00000025937	
ENSGALT00000020590	RTCB
ENSGALT00000030816	
ENSGALT00000025971	
ENSGALT00000025969	UTP23
ENSGALT00000025965	
ENSGALT00000020629	
ENSGALT00000020663	CKAP4
ENSGALT00000020668	NUAK1
ENSGALT00000026475	
ENSGALT00000026431	
ENSGALT00000020683	
ENSGALT00000026325	KIAA0196
ENSGALT00000026313	TRIB1
ENSGALT00000026309	
ENSGALT00000020769	
ENSGALT00000030784	FAM49B
ENSGALT00000020773	
ENSGALT00000020821	
ENSGALT00000020822	CCDC53
ENSGALT00000020826	GNPTAB
ENSGALT00000020835	
ENSGALT00000020864	PARP12
ENSGALT00000020873	HIPK2
ENSGALT00000026044	GPR20
ENSGALT00000026043	PTP4A3
ENSGALT00000026025	
ENSGALT00000020934	
ENSGALT00000020953	KDM7A

ENSGALT00000020996	AGK
ENSGALT00000021056	SYT10
ENSGALT00000021073	YARS2
ENSGALT00000021086	
ENSGALT00000021134	TMTC1
ENSGALT00000021203	NINJ2
ENSGALT00000021281	
ENSGALT00000021332	
ENSGALT00000021337	
ENSGALT00000021340	
ENSGALT00000021374	
ENSGALT00000021380	
ENSGALT00000021517	
ENSGALT00000021594	
ENSGALT00000021626	ABCC9
ENSGALT00000022801	ASUN
ENSGALT00000022820	
ENSGALT00000022856	CYB5R3
ENSGALT00000022912	
ENSGALT00000022945	SULT4A1
ENSGALT00000022959	
ENSGALT00000022966	PARVB
ENSGALT00000022998	
ENSGALT00000023003	KIAA0930
ENSGALT00000022888	
ENSGALT00000027944	
ENSGALT00000030043	
ENSGALT00000023174	TSPAN9
ENSGALT00000021894	TULP3
ENSGALT00000023158	
ENSGALT00000023157	NECAP1
ENSGALT00000023197	MAN1A2
ENSGALT00000023287	
ENSGALT00000023289	FAM162A
ENSGALT00000022680	FBXO40
ENSGALT00000023323	
ENSGALT00000023360	
ENSGALT00000023361	
ENSGALT00000023442	
ENSGALT00000023485	
ENSGALT00000023492	
ENSGALT00000023496	LPCAT3

ENSGALT00000023545	
ENSGALT00000023646	CLSTN3
ENSGALT00000023710	
ENSGALT00000023808	STYK1
ENSGALT00000023883	GDAP2
ENSGALT00000023902	
ENSGALT00000024021	
ENSGALT00000024035	
ENSGALT00000036952	
ENSGALT00000024316	B4GALT3
ENSGALT00000024333	IGSF11
ENSGALT00000024530	
ENSGALT00000024570	BLZF1
ENSGALT00000024577	NME7
ENSGALT00000030447	DPT
ENSGALT00000024599	TIPRL
ENSGALT00000024644	CMSS1
ENSGALT00000024648	FILIP1L
ENSGALT00000024670	TOMM70A
ENSGALT00000024704	ABI3BP
ENSGALT00000024756	RPL24
ENSGALT00000036897	HHLA2
ENSGALT00000024797	PVRL3
ENSGALT00000024837	
ENSGALT00000024843	C3orf38
ENSGALT00000024855	PROS1
ENSGALT00000024895	DCAF6
ENSGALT00000024900	
ENSGALT00000030405	
ENSGALT00000024975	
ENSGALT00000024990	
ENSGALT00000025002	GBE1
ENSGALT00000036824	
ENSGALT00000025349	CHODL
ENSGALT00000025402	ATP5J
ENSGALT00000025479	
ENSGALT00000025487	RWDD2B
ENSGALT00000025511	MAP3K7CL
ENSGALT00000025518	BACH1
ENSGALT00000025609	
ENSGALT00000025738	
ENSGALT00000025792	

ENSGALT00000025800	
ENSGALT00000025863	DSCR3
ENSGALT00000025866	
ENSGALT00000025873	
ENSGALT00000025891	
ENSGALT00000036704	
ENSGALT00000026100	PKNOX1
ENSGALT00000026124	
ENSGALT00000026125	AGPAT3
ENSGALT00000030337	
ENSGALT00000026137	CXorf36
ENSGALT00000026167	
ENSGALT00000026174	
ENSGALT00000026207	
ENSGALT00000026220	
ENSGALT00000026255	TAB3
ENSGALT00000026256	
ENSGALT00000026334	
ENSGALT00000026357	
ENSGALT00000026363	
ENSGALT00000026366	APOO
ENSGALT00000026387	PRDX4
ENSGALT00000030302	SMPX
ENSGALT00000026456	RPS6KA3
ENSGALT00000026483	CXorf23
ENSGALT00000026498	MAP3K15
ENSGALT00000026507	
ENSGALT00000026711	
ENSGALT00000026728	ASB11
ENSGALT00000026753	
ENSGALT00000026761	
ENSGALT00000026762	
ENSGALT00000026770	
ENSGALT00000026803	CLCN4
ENSGALT00000026917	DHRSX
ENSGALT00000026928	ASMTL
ENSGALT00000026955	PPP2R3B
ENSGALT00000026993	
ENSGALT00000027031	ATP10A
ENSGALT00000027040	UBE3A
ENSGALT00000036592	
ENSGALT00000027049	INPP4A

ENSGALT00000027060	MITD1
ENSGALT00000036589	TXNDC9
ENSGALT00000027078	
ENSGALT00000027087	CNOT11
ENSGALT00000027090	RNF149
ENSGALT00000027111	MFSD9
ENSGALT00000027114	
ENSGALT00000027119	FHL2
ENSGALT00000027121	
ENSGALT00000027123	UXS1
ENSGALT00000036568	
ENSGALT00000027149	
ENSGALT00000027160	
ENSGALT00000027189	TUBGCP3
ENSGALT00000027208	
ENSGALT00000027212	CARKD
ENSGALT00000027229	
ENSGALT00000027236	
ENSGALT00000027240	KDELC1
ENSGALT00000027243	METTL21C
ENSGALT00000027247	ITGBL1
ENSGALT00000027264	CLYBL
ENSGALT00000027267	TM9SF2
ENSGALT00000027273	DOCK9
ENSGALT00000027289	
ENSGALT00000027314	GPR180
ENSGALT00000027349	CLN5
ENSGALT00000027356	
ENSGALT00000027358	COMMD6
ENSGALT00000027364	KLF5
ENSGALT00000027373	
ENSGALT00000027404	VWA8
ENSGALT00000027417	
ENSGALT00000027432	SLC25A30
ENSGALT00000027443	
ENSGALT00000027446	KIAA0226L
ENSGALT00000027451	
ENSGALT00000027453	
ENSGALT00000030149	
ENSGALT00000036484	
ENSGALT00000027476	
ENSGALT00000027505	

ENSGALT00000027509	
ENSGALT00000027522	
ENSGALT00000027523	COG6
ENSGALT00000027525	LHFP
ENSGALT00000027527	PROSER1
ENSGALT00000027531	
ENSGALT00000027540	
ENSGALT00000027584	B3GALTL
ENSGALT00000027586	
ENSGALT00000027597	
ENSGALT00000030102	POMP
ENSGALT00000027648	MTMR6
ENSGALT00000027655	MIPEP
ENSGALT00000027657	
ENSGALT00000027661	SGCG
ENSGALT00000027664	MICU2
ENSGALT00000027671	
ENSGALT00000027676	
ENSGALT00000027677	
ENSGALT00000027680	
ENSGALT00000027690	
ENSGALT00000021058	
ENSGALT00000027711	KDELC2
ENSGALT00000027718	
ENSGALT00000027725	CUL5
ENSGALT00000027757	DCUN1D5
ENSGALT00000036427	
ENSGALT00000027810	SESN3
ENSGALT00000027816	
ENSGALT00000027818	ANKRD49
ENSGALT00000027831	
ENSGALT00000027869	CTSC
ENSGALT00000027880	ME3
ENSGALT00000027882	RAB30
ENSGALT00000027884	PCF11
ENSGALT00000027888	CCDC90B
ENSGALT00000022773	
ENSGALT00000002817	
ENSGALT00000027904	NARS2
ENSGALT00000002458	AQP11
ENSGALT00000001192	LRRC32
ENSGALT00000036387	

ENSGALT00000001279	RNF121
ENSGALT00000028034	
ENSGALT00000028035	STIM1
ENSGALT00000022062	
ENSGALT00000014840	DGAT2
ENSGALT00000018287	
ENSGALT00000031276	
ENSGALT00000027989	
ENSGALT00000026436	KLHL34
ENSGALT00000026765	TCEANC
ENSGALT00000027059	
ENSGALT00000027144	CHAMP1
ENSGALT00000027875	PRSS23
ENSGALT00000007813	TMEM119
ENSGALT00000010788	PPP1R3C
ENSGALT00000024049	HSPB3
ENSGALT00000024541	
ENSGALT00000001984	
ENSGALT00000011424	FAM43A
ENSGALT00000017230	FLRT2
ENSGALT00000019144	
ENSGALT00000022862	A4GALT
ENSGALT00000006628	FEM1A
ENSGALT00000002482	UTS2R
ENSGALT00000011909	
ENSGALT00000014235	
ENSGALT00000001876	
ENSGALT00000030942	LRRC30
ENSGALT00000026439	ZHX2
ENSGALT00000026354	
ENSGALT00000007889	
ENSGALT00000014752	
ENSGALT00000018781	
ENSGALT00000013613	
ENSGALT00000013616	THBD
ENSGALT00000017169	CDC42EP3
ENSGALT00000017907	IRF2BP2
ENSGALT00000008264	PDP2
ENSGALT00000008793	
ENSGALT00000007518	
ENSGALT00000040564	
ENSGALT00000006563	

ENSGALT00000009554	AGTR2
ENSGALT00000014023	
ENSGALT00000014971	SLITRK2
ENSGALT00000033813	
ENSGALT00000013983	GPR1
ENSGALT00000015011	
ENSGALT00000018926	TMEM177
ENSGALT00000004272	
ENSGALT00000011035	KLHL25
ENSGALT00000011869	KBTBD13
ENSGALT00000013924	
ENSGALT00000018475	SNAPC3
ENSGALT00000029098	
ENSGALT00000029084	
ENSGALT00000029081	

DEGs between CH and WL		DEGs between AA and WL	
**Ensembl Transcript ID**	**HGNC symbol**	**Ensembl Transcript ID**	**HGNC symbol**
ENSGALT00000029647		ENSGALT00000029647	
ENSGALT00000000407		ENSGALT00000000407	
ENSGALT00000000510		ENSGALT00000040207	
ENSGALT00000040207		ENSGALT00000000555	
ENSGALT00000000555		ENSGALT00000040563	ANKMY1
ENSGALT00000040563	ANKMY1	ENSGALT00000000556	
ENSGALT00000000587		ENSGALT00000000587	
ENSGALT00000014902		ENSGALT00000001184	MYH13
ENSGALT00000001184	MYH13	ENSGALT00000014829	DPP7
ENSGALT00000014829	DPP7	ENSGALT00000000671	LONP1
ENSGALT00000000671	LONP1	ENSGALT00000014774	MAN1B1
ENSGALT00000014774	MAN1B1	ENSGALT00000014744	
ENSGALT00000014744		ENSGALT00000000806	RANBP3
ENSGALT00000000806	RANBP3	ENSGALT00000000837	MARCH2
ENSGALT00000001427		ENSGALT00000000866	
ENSGALT00000041277		ENSGALT00000014712	NPDC1
ENSGALT00000014675		ENSGALT00000014675	
ENSGALT00000014665	TRAF2	ENSGALT00000014665	TRAF2
ENSGALT00000014657		ENSGALT00000014657	
ENSGALT00000001029		ENSGALT00000001029	
ENSGALT00000014614	RABL6	ENSGALT00000014614	RABL6
ENSGALT00000001137	PIP5K1C	ENSGALT00000001137	PIP5K1C
ENSGALT00000001477	MAP2K4	ENSGALT00000001477	MAP2K4
ENSGALT00000001507		ENSGALT00000001507	

ENSGALT00000001534	ELAC2	ENSGALT00000001534	ELAC2
ENSGALT00000014440	ZNF618	ENSGALT00000014440	ZNF618
ENSGALT00000002041		ENSGALT00000002041	
ENSGALT00000002095		ENSGALT00000002095	
ENSGALT00000002182	B3GNTL1	ENSGALT00000014372	SLC31A1
ENSGALT00000014372	SLC31A1	ENSGALT00000002349	
ENSGALT00000002349		ENSGALT00000028221	
ENSGALT00000014359	FKBP15	ENSGALT00000014359	FKBP15
ENSGALT00000002374		ENSGALT00000002374	
ENSGALT00000002396	FOXK2	ENSGALT00000002416	
ENSGALT00000002433	NARF	ENSGALT00000002433	NARF
ENSGALT00000002490		ENSGALT00000002445	C17orf62
ENSGALT00000014314		ENSGALT00000002469	OGFOD3
ENSGALT00000002733	MFSD11	ENSGALT00000002490	
ENSGALT00000002759		ENSGALT00000002528	
ENSGALT00000002784		ENSGALT00000002733	MFSD11
ENSGALT00000034851		ENSGALT00000002759	
ENSGALT00000014239	DPH7	ENSGALT00000002784	
ENSGALT00000003062	UBE2O	ENSGALT00000002789	MXRA7
ENSGALT00000002885	PRPSAP1	ENSGALT00000034851	
ENSGALT00000014053	ANAPC2	ENSGALT00000014239	DPH7
ENSGALT00000021823	CMA1	ENSGALT00000003062	UBE2O
ENSGALT00000014039		ENSGALT00000002885	PRPSAP 1
ENSGALT00000021812		ENSGALT00000014053	ANAPC2
ENSGALT00000003210		ENSGALT00000021823	CMA1
ENSGALT00000013811		ENSGALT00000014039	
ENSGALT00000021807		ENSGALT00000021812	
ENSGALT00000003433		ENSGALT00000003210	
ENSGALT00000003475		ENSGALT00000013811	
ENSGALT00000003571	UNK	ENSGALT00000003381	
ENSGALT00000011643		ENSGALT00000021807	
ENSGALT00000002831		ENSGALT00000003433	
ENSGALT00000003634	GALK1	ENSGALT00000003475	
ENSGALT00000003804	SAP30BP	ENSGALT00000003571	UNK
ENSGALT00000004306		ENSGALT00000011643	
ENSGALT00000004482		ENSGALT00000002831	
ENSGALT00000004555		ENSGALT00000003634	GALK1
ENSGALT00000004579	NOTUM	ENSGALT00000003804	SAP30BP
ENSGALT00000004723	UTP18	ENSGALT00000004306	
ENSGALT00000034713	TOM1L1	ENSGALT00000004482	
ENSGALT00000011421		ENSGALT00000004555	

ENSGALT00000011333		ENSGALT00000004579	NOTUM
ENSGALT00000004865		ENSGALT00000004723	UTP18
ENSGALT00000004896	PCTP	ENSGALT00000011421	
ENSGALT00000002422	MLLT1	ENSGALT00000011292	CDK5RAP2
ENSGALT00000004998	COIL	ENSGALT00000004865	
ENSGALT00000005054		ENSGALT00000002682	
ENSGALT00000034695		ENSGALT00000004896	PCTP
ENSGALT00000005180	SUZ12	ENSGALT00000002422	MLLT1
ENSGALT00000008172	SLC25A25	ENSGALT00000004971	TRIM25
ENSGALT00000005437	C17orf75	ENSGALT00000005054	
ENSGALT00000008132		ENSGALT00000039989	MEGF9
ENSGALT00000001193		ENSGALT00000034695	
ENSGALT00000008122		ENSGALT00000005180	SUZ12
ENSGALT00000008495	ST6GALNAC6	ENSGALT00000008172	SLC25A25
ENSGALT00000001236	DOT1L	ENSGALT00000005437	C17orf75
ENSGALT00000001472	AP3D1	ENSGALT00000008132	
ENSGALT00000005503	ERN1	ENSGALT00000001193	
ENSGALT00000007990	CERCAM	ENSGALT00000008122	
ENSGALT00000034662	TEX2	ENSGALT00000008495	ST6GALNAC6
ENSGALT00000007962	SLC27A4	ENSGALT00000041212	
ENSGALT00000006110	MKNK2	ENSGALT00000001236	DOT1L
ENSGALT00000007881	COQ4	ENSGALT00000001472	AP3D1
ENSGALT00000001502	BTBD2	ENSGALT00000005503	ERN1
ENSGALT00000005586		ENSGALT00000007990	CERCAM
ENSGALT00000005647	SMURF2	ENSGALT00000034662	TEX2
ENSGALT00000005672		ENSGALT00000007962	SLC27A4
ENSGALT00000001527	SCAMP4	ENSGALT00000017003	MOB3A
ENSGALT00000005785		ENSGALT00000006110	MKNK2
ENSGALT00000023521		ENSGALT00000007881	COQ4
ENSGALT00000029695	ABHD17A	ENSGALT00000001502	BTBD2
ENSGALT00000001557		ENSGALT00000005586	
ENSGALT00000007637		ENSGALT00000005647	SMURF2
ENSGALT00000007631	PTGES2	ENSGALT00000005672	
ENSGALT00000007603		ENSGALT00000034629	C17orf58
ENSGALT00000007546	GLE1	ENSGALT00000001527	SCAMP4
ENSGALT00000005813		ENSGALT00000005785	
ENSGALT00000005864		ENSGALT00000023521	
ENSGALT00000006197		ENSGALT00000029695	ABHD17A

ENSGALT00000006288		ENSGALT00000001557	
ENSGALT00000001662	MIER2	ENSGALT00000007637	
ENSGALT00000001723	UBXN6	ENSGALT00000007631	PTGES2
ENSGALT00000006617	SLC16A6	ENSGALT00000007603	
ENSGALT00000006701	WIPI1	ENSGALT00000007546	GLE1
ENSGALT00000035511	PLIN3	ENSGALT00000005813	
ENSGALT00000001774	SEMA6B	ENSGALT00000005864	
ENSGALT00000006909		ENSGALT00000006197	
ENSGALT00000006907	ABCA5	ENSGALT00000006288	
ENSGALT00000006971	MAP2K6	ENSGALT00000001662	MIER2
ENSGALT00000001934		ENSGALT00000001723	UBXN6
ENSGALT00000007063	ATP5J2	ENSGALT00000006617	SLC16A6
ENSGALT00000038499	CDC42EP4	ENSGALT00000006701	WIPI1
ENSGALT00000002009		ENSGALT00000035511	PLIN3
ENSGALT00000029664		ENSGALT00000001774	SEMA6B
ENSGALT00000002022		ENSGALT00000006907	ABCA5
ENSGALT00000007431	ZDHHC12	ENSGALT00000006971	MAP2K6
ENSGALT00000007424	ZER1	ENSGALT00000001934	
ENSGALT00000002098	POLRMT	ENSGALT00000007063	ATP5J2
ENSGALT00000002107	FGF22	ENSGALT00000038499	CDC42EP4
ENSGALT00000002126		ENSGALT00000002009	
ENSGALT00000007398		ENSGALT00000029664	
ENSGALT00000007292	NPLOC4	ENSGALT00000002022	
ENSGALT00000007279	LRRC8A	ENSGALT00000007431	ZDHHC12
ENSGALT00000011145	SLC38A10	ENSGALT00000007424	ZER1
ENSGALT00000007386		ENSGALT00000002098	POLRMT
ENSGALT00000007251		ENSGALT00000002126	
ENSGALT00000035079		ENSGALT00000007398	
ENSGALT00000007150	FAM73B	ENSGALT00000007292	NPLOC4
ENSGALT00000011206	BAIAP2	ENSGALT00000007326	CCBL1
ENSGALT00000011238	RPTOR	ENSGALT00000007279	LRRC8A
ENSGALT00000007143		ENSGALT00000011145	SLC38A10
ENSGALT00000007108	CRAT	ENSGALT00000007386	
ENSGALT00000003154	TMEM259	ENSGALT00000007251	
ENSGALT00000034684	TOR1B	ENSGALT00000011176	AATK
ENSGALT00000006623		ENSGALT00000035079	
ENSGALT00000011427	TBC1D16	ENSGALT00000007150	FAM73B
ENSGALT00000011440		ENSGALT00000011238	RPTOR
ENSGALT00000006585	USP20	ENSGALT00000007143	

ENSGALT00000006504	FNBP1	ENSGALT00000007108	CRAT
ENSGALT00000040552		ENSGALT00000003154	TMEM259
ENSGALT00000032738		ENSGALT00000002579	
ENSGALT00000006449		ENSGALT00000006623	
ENSGALT00000006164		ENSGALT00000011427	TBC1D16
ENSGALT00000008293		ENSGALT00000006585	USP20
ENSGALT00000035078		ENSGALT00000019296	
ENSGALT00000004211	SBNO2	ENSGALT00000006504	FNBP1
ENSGALT00000019966		ENSGALT00000040552	
ENSGALT00000006070	NUP214	ENSGALT00000032738	
ENSGALT00000005350	SPTB	ENSGALT00000032737	
ENSGALT00000011630		ENSGALT00000021548	
ENSGALT00000000158	AFMID	ENSGALT00000006449	
ENSGALT00000006013	FAM78A	ENSGALT00000004080	POLR2E
ENSGALT00000006004	PPAPDC3	ENSGALT00000006164	
ENSGALT00000005992	PRRC2B	ENSGALT00000008293	
ENSGALT00000011644		ENSGALT00000035078	
ENSGALT00000011691	HGS	ENSGALT00000004211	SBNO2
ENSGALT00000005921	UCK1	ENSGALT00000019966	
ENSGALT00000035065		ENSGALT00000006070	NUP214
ENSGALT00000021732	DAGLA	ENSGALT00000005350	SPTB
ENSGALT00000011703		ENSGALT00000011630	
ENSGALT00000013753		ENSGALT00000006013	FAM78A
ENSGALT00000011710		ENSGALT00000006004	PPAPDC3
ENSGALT00000008416		ENSGALT00000005992	PRRC2B
ENSGALT00000011734	PCYT2	ENSGALT00000011644	
ENSGALT00000021713	CPSF7	ENSGALT00000011691	HGS
ENSGALT00000005588	DDX31	ENSGALT00000005921	UCK1
ENSGALT00000011813		ENSGALT00000005734	
ENSGALT00000011818		ENSGALT00000032890	SLC39A3
ENSGALT00000004127	SLC25A42	ENSGALT00000035065	
ENSGALT00000011101	SPATA20	ENSGALT00000021732	DAGLA
ENSGALT00000005537	TSC1	ENSGALT00000011703	
ENSGALT00000038280	EPN3	ENSGALT00000013753	
ENSGALT00000004779	ARMC6	ENSGALT00000011710	
ENSGALT00000004817	SUGP2	ENSGALT00000005834	
ENSGALT00000009269		ENSGALT00000008416	
ENSGALT00000004856	HOMER3	ENSGALT00000011734	PCYT2
ENSGALT00000008347	VPS37C	ENSGALT00000005588	DDX31
ENSGALT00000031388	CHAD	ENSGALT00000011813	

ENSGALT00000005397	RALGDS	ENSGALT00000011818	
ENSGALT00000012479		ENSGALT00000004127	SLC25A42
ENSGALT00000032736	MS4A15	ENSGALT00000038316	
ENSGALT00000005000		ENSGALT00000011101	SPATA20
ENSGALT00000005285		ENSGALT00000005537	TSC1
ENSGALT00000012560		ENSGALT00000038280	EPN3
ENSGALT00000005085		ENSGALT00000004779	ARMC6
ENSGALT00000012890		ENSGALT00000004817	SUGP2
ENSGALT00000005016	REXO4	ENSGALT00000009269	
ENSGALT00000005192	COMP	ENSGALT00000004856	HOMER3
ENSGALT00000005236	CRTC1	ENSGALT00000008347	VPS37C
ENSGALT00000012930		ENSGALT00000031388	CHAD
ENSGALT00000005309		ENSGALT00000005397	RALGDS
ENSGALT00000007496		ENSGALT00000012479	
ENSGALT00000007466		ENSGALT00000032736	MS4A15
ENSGALT00000005348	LSM4	ENSGALT00000004991	
ENSGALT00000004669		ENSGALT00000005285	
ENSGALT00000007402		ENSGALT00000012560	
ENSGALT00000013002	SLC25A19	ENSGALT00000012666	HID1
ENSGALT00000005355		ENSGALT00000005085	
ENSGALT00000029662		ENSGALT00000005056	
ENSGALT00000006156		ENSGALT00000012890	
ENSGALT00000040443	RPUSD2	ENSGALT00000005016	REXO4
ENSGALT00000013018	KIAA0195	ENSGALT00000005192	COMP
ENSGALT00000013031	CASKIN2	ENSGALT00000005236	CRTC1
ENSGALT00000001658		ENSGALT00000012930	
ENSGALT00000005424		ENSGALT00000012934	
ENSGALT00000040420		ENSGALT00000008160	
ENSGALT00000006955		ENSGALT00000004792	ADAMTS13
ENSGALT00000004255		ENSGALT00000005309	
ENSGALT00000023312	RETSAT	ENSGALT00000005330	
ENSGALT00000004226	BRD3	ENSGALT00000007496	
ENSGALT00000006852		ENSGALT00000007466	
ENSGALT00000005581	ARRDC2	ENSGALT00000005348	LSM4
ENSGALT00000004139	RXRA	ENSGALT00000004669	
ENSGALT00000005655		ENSGALT00000007402	
ENSGALT00000039021	AGPAT2	ENSGALT00000013002	SLC25A19
ENSGALT00000006685		ENSGALT00000005355	
ENSGALT00000006531		ENSGALT00000029662	

ENSGALT00000005938	CPAMD8	ENSGALT00000006156	
ENSGALT00000006436		ENSGALT00000040443	RPUSD2
ENSGALT00000005982	SIN3B	ENSGALT00000013018	KIAA0195
ENSGALT00000040276	NAV2	ENSGALT00000013031	CASKIN2
ENSGALT00000006317		ENSGALT00000004499	SARDH
ENSGALT00000003726	SEC16A	ENSGALT00000001658	
ENSGALT00000005994		ENSGALT00000005424	
ENSGALT00000006018	SLC35E1	ENSGALT00000006955	
ENSGALT00000006060		ENSGALT00000004255	
ENSGALT00000003045	INPP5E	ENSGALT00000023312	RETSAT
ENSGALT00000003018		ENSGALT00000006901	IGHMBP2
ENSGALT00000006230	EPS15L1	ENSGALT00000004226	BRD3
ENSGALT00000019904	LGR4	ENSGALT00000006852	
ENSGALT00000019825		ENSGALT00000004139	RXRA
ENSGALT00000031814		ENSGALT00000005655	
ENSGALT00000019813	ELP4	ENSGALT00000040352	PSMD13
ENSGALT00000002770	GPSM1	ENSGALT00000003844	FAM69B
ENSGALT00000040104	RCN1	ENSGALT00000039021	AGPAT2
ENSGALT00000034856	QSOX2	ENSGALT00000006685	
ENSGALT00000006310		ENSGALT00000006633	
ENSGALT00000002629		ENSGALT00000006531	
ENSGALT00000002593	CAMSAP1	ENSGALT00000005938	CPAMD8
ENSGALT00000039281	TPM4	ENSGALT00000005982	SIN3B
ENSGALT00000019117		ENSGALT00000040276	NAV2
ENSGALT00000008620		ENSGALT00000006317	
ENSGALT00000020812		ENSGALT00000003726	SEC16A
ENSGALT00000008698		ENSGALT00000005994	
ENSGALT00000008731	PARVA	ENSGALT00000006018	SLC35E1
ENSGALT00000008888	MICAL2	ENSGALT00000006060	
ENSGALT00000006684	DPP9	ENSGALT00000003045	INPP5E
ENSGALT00000008904		ENSGALT00000003018	
ENSGALT00000008932		ENSGALT00000006230	EPS15L1
ENSGALT00000039859		ENSGALT00000019904	LGR4
ENSGALT00000006779		ENSGALT00000019825	
ENSGALT00000006783		ENSGALT00000031814	
ENSGALT00000009054		ENSGALT00000019813	ELP4
ENSGALT00000009084		ENSGALT00000002770	GPSM1
ENSGALT00000009088	ADM	ENSGALT00000040104	RCN1
ENSGALT00000002299	CNTRL	ENSGALT00000034856	QSOX2
ENSGALT00000009266		ENSGALT00000006310	
ENSGALT00000009326	IPO7	ENSGALT00000002629	
ENSGALT00000009336		ENSGALT00000002593	CAMSAP

			1
ENSGALT00000002236		ENSGALT00000039281	TPM4
ENSGALT00000009504		ENSGALT00000019109	
ENSGALT00000034894	STOM	ENSGALT00000006575	
ENSGALT00000002162	DAB2IP	ENSGALT00000008620	
ENSGALT00000009626	TUB	ENSGALT00000020812	
ENSGALT00000009649		ENSGALT00000008698	
ENSGALT00000034898		ENSGALT00000008731	PARVA
ENSGALT00000001902	RC3H2	ENSGALT00000008888	MICAL2
ENSGALT00000009835		ENSGALT00000006684	DPP9
ENSGALT00000001877		ENSGALT00000008904	
ENSGALT00000001117	PLA2G15	ENSGALT00000039859	
ENSGALT00000001165	SLC7A6OS	ENSGALT00000006779	
ENSGALT00000001259	SLC7A6	ENSGALT00000006783	
ENSGALT00000001294	GINS3	ENSGALT00000003081	MED16
ENSGALT00000032718	KCNJ11	ENSGALT00000009037	MRVI1
ENSGALT00000001304		ENSGALT00000009054	
ENSGALT00000001320		ENSGALT00000009084	
ENSGALT00000001489	MMP15	ENSGALT00000009088	ADM
ENSGALT00000001326	USB1	ENSGALT00000002299	CNTRL
ENSGALT00000001687		ENSGALT00000009266	
ENSGALT00000010128	HPS5	ENSGALT00000009326	IPO7
ENSGALT00000001535	SCAI	ENSGALT00000009336	
ENSGALT00000001768	POLR2C	ENSGALT00000002236	
ENSGALT00000001476		ENSGALT00000009504	
ENSGALT00000010207		ENSGALT00000034894	STOM
ENSGALT00000010272		ENSGALT00000002162	DAB2IP
ENSGALT00000001436		ENSGALT00000009626	TUB
ENSGALT00000001402		ENSGALT00000009649	
ENSGALT00000001372	ZBTB43	ENSGALT00000034898	
ENSGALT00000001330	RALGPS1	ENSGALT00000001902	RC3H2
ENSGALT00000001810		ENSGALT00000009835	
ENSGALT00000010333	OSBPL5	ENSGALT00000001877	
ENSGALT00000010405		ENSGALT00000001117	PLA2G15
ENSGALT00000001852	HERPUD1	ENSGALT00000001165	SLC7A6OS
ENSGALT00000001362		ENSGALT00000001294	GINS3
ENSGALT00000001871		ENSGALT00000032718	KCNJ11
ENSGALT00000001908	NLRC5	ENSGALT00000001304	
ENSGALT00000002065	RSPRY1	ENSGALT00000001320	
ENSGALT00000002191	RANBP10	ENSGALT00000001489	MMP15
ENSGALT00000002429		ENSGALT00000001326	USB1

ENSGALT00000002498	EDC4	ENSGALT00000001687	
ENSGALT00000002514		ENSGALT00000010128	HPS5
ENSGALT00000010619		ENSGALT00000001514	GOLGA1
ENSGALT00000010620		ENSGALT00000001768	POLR2C
ENSGALT00000039194	C11orf89	ENSGALT00000001496	ARPC5L
ENSGALT00000010641		ENSGALT00000001476	
ENSGALT00000010676		ENSGALT00000010207	
ENSGALT00000010751	DUSP8	ENSGALT00000010251	
ENSGALT00000010774	MOB2	ENSGALT00000010272	
ENSGALT00000010823		ENSGALT00000001436	
ENSGALT00000003047		ENSGALT00000001402	
ENSGALT00000010998	AP2A2	ENSGALT00000001372	ZBTB43
ENSGALT00000040176	PLEKHG4	ENSGALT00000038660	
ENSGALT00000003500		ENSGALT00000001810	
ENSGALT00000003653		ENSGALT00000010333	OSBPL5
ENSGALT00000011039		ENSGALT00000010376	
ENSGALT00000011062	TSPAN4	ENSGALT00000010405	
ENSGALT00000039051		ENSGALT00000001852	HERPUD1
ENSGALT00000003894	MTSS1L	ENSGALT00000001362	
ENSGALT00000032690	EFCAB4A	ENSGALT00000010554	
ENSGALT00000023495		ENSGALT00000002065	RSPRY1
ENSGALT00000003987		ENSGALT00000002191	RANBP10
ENSGALT00000004241	PDPR	ENSGALT00000002429	
ENSGALT00000011181		ENSGALT00000002498	EDC4
ENSGALT00000022893	SUV420H1	ENSGALT00000002514	
ENSGALT00000032683		ENSGALT00000010619	
ENSGALT00000011466		ENSGALT00000039194	C11orf89
ENSGALT00000011547		ENSGALT00000010641	
ENSGALT00000011621		ENSGALT00000010676	
ENSGALT00000004497		ENSGALT00000010774	MOB2
ENSGALT00000011657		ENSGALT00000010823	
ENSGALT00000004515		ENSGALT00000003047	
ENSGALT00000017615	DTX4	ENSGALT00000010998	AP2A2
ENSGALT00000011857		ENSGALT00000003282	TPPP3
ENSGALT00000022891	FCGBP	ENSGALT00000040176	PLEKHG4
ENSGALT00000011921		ENSGALT00000003500	
ENSGALT00000007737	TIMM10	ENSGALT00000003653	
ENSGALT00000038761	RTN4RL2	ENSGALT00000039051	
ENSGALT00000004875		ENSGALT00000003894	MTSS1L

ENSGALT00000012340	FADD	ENSGALT00000032690	EFCAB4A
ENSGALT00000004906	AMFR	ENSGALT00000023495	
ENSGALT00000005216	MBTPS1	ENSGALT00000003987	
ENSGALT00000012655		ENSGALT00000004100	
ENSGALT00000010684	FBXO3	ENSGALT00000004241	PDPR
ENSGALT00000005262	OSGIN1	ENSGALT00000011181	
ENSGALT00000005367	NFATC3	ENSGALT00000022893	SUV420H1
ENSGALT00000022035		ENSGALT00000032683	
ENSGALT00000005597	SLC12A4	ENSGALT00000011547	
ENSGALT00000012705		ENSGALT00000011621	
ENSGALT00000012746		ENSGALT00000004497	
ENSGALT00000012805	PAMR1	ENSGALT00000011657	
ENSGALT00000012829	COMMD9	ENSGALT00000017615	DTX4
ENSGALT00000012888	TRAF6	ENSGALT00000011857	
ENSGALT00000005666		ENSGALT00000022891	FCGBP
ENSGALT00000005731		ENSGALT00000011921	
ENSGALT00000021154		ENSGALT00000007737	TIMM10
ENSGALT00000016123	CYLD	ENSGALT00000038761	RTN4RL2
ENSGALT00000013052		ENSGALT00000004875	
ENSGALT00000022785		ENSGALT00000012208	TPCN2
ENSGALT00000030003		ENSGALT00000012340	FADD
ENSGALT00000013121		ENSGALT00000004906	AMFR
ENSGALT00000034245		ENSGALT00000005064	TRADD
ENSGALT00000006188	ZNF423	ENSGALT00000005070	
ENSGALT00000013128		ENSGALT00000005176	
ENSGALT00000006236	SIAH1	ENSGALT00000005216	MBTPS1
ENSGALT00000006283		ENSGALT00000012655	
ENSGALT00000006378		ENSGALT00000010684	FBXO3
ENSGALT00000013183		ENSGALT00000005262	OSGIN1
ENSGALT00000013191		ENSGALT00000005367	NFATC3
ENSGALT00000013356		ENSGALT00000022035	
ENSGALT00000006871	MYLK3	ENSGALT00000005388	DUS2
ENSGALT00000013368		ENSGALT00000005597	SLC12A4
ENSGALT00000013396		ENSGALT00000023364	CAT
ENSGALT00000013425		ENSGALT00000012705	
ENSGALT00000006976		ENSGALT00000012805	PAMR1
ENSGALT00000007136		ENSGALT00000012829	COMMD9
ENSGALT00000007149		ENSGALT00000012861	PRR5L
ENSGALT00000007159		ENSGALT00000012888	TRAF6
ENSGALT00000013551		ENSGALT00000012953	

ENSGALT00000013587	ATG13	ENSGALT00000005666	
ENSGALT00000035181	PFKFB1	ENSGALT00000005731	
ENSGALT00000007751	LRP3	ENSGALT00000005902	CHD9
ENSGALT00000007771		ENSGALT00000016123	CYLD
ENSGALT00000013748		ENSGALT00000013011	EXT2
ENSGALT00000038342		ENSGALT00000013052	
ENSGALT00000013841		ENSGALT00000022785	
ENSGALT00000013867	DLL4	ENSGALT00000030003	
ENSGALT00000013933		ENSGALT00000006125	PAPD5
ENSGALT00000013981	NDUFAF1	ENSGALT00000013121	
ENSGALT00000007911	SS18L2	ENSGALT00000034245	
ENSGALT00000007938	KIAA0355	ENSGALT00000006188	ZNF423
ENSGALT00000014000		ENSGALT00000013128	
ENSGALT00000014145	MAPKBP1	ENSGALT00000006283	
ENSGALT00000008241	FAM96B	ENSGALT00000013143	
ENSGALT00000008251		ENSGALT00000006378	
ENSGALT00000014555	EHD4	ENSGALT00000013183	
ENSGALT00000014672	TMEM87A	ENSGALT00000013356	
ENSGALT00000039221		ENSGALT00000006871	MYLK3
ENSGALT00000008447		ENSGALT00000013368	
ENSGALT00000008452		ENSGALT00000006929	
ENSGALT00000008465		ENSGALT00000013425	
ENSGALT00000014708		ENSGALT00000006976	
ENSGALT00000014809		ENSGALT00000007136	
ENSGALT00000015071	TMEM62	ENSGALT00000007149	
ENSGALT00000015102	ELMSAN1	ENSGALT00000007159	
ENSGALT00000038149		ENSGALT00000013551	
ENSGALT00000015117		ENSGALT00000013587	ATG13
ENSGALT00000033808		ENSGALT00000013673	CREB3L1
ENSGALT00000015198		ENSGALT00000035181	PFKFB1
ENSGALT00000029669	HOXC10	ENSGALT00000007751	LRP3
ENSGALT00000021972	ATMIN	ENSGALT00000007771	
ENSGALT00000015206		ENSGALT00000013748	
ENSGALT00000015283		ENSGALT00000007832	
ENSGALT00000000388	PCNX	ENSGALT00000013841	
ENSGALT00000022454		ENSGALT00000013867	DLL4
ENSGALT00000015296		ENSGALT00000013933	
ENSGALT00000015312		ENSGALT00000032642	
ENSGALT00000015350	KIAA0247	ENSGALT00000013978	
ENSGALT00000015368		ENSGALT00000013981	NDUFAF 1
ENSGALT00000008821		ENSGALT00000007938	KIAA0355

ENSGALT00000015588		ENSGALT00000034006	
ENSGALT00000033760		ENSGALT00000014000	
ENSGALT00000008832		ENSGALT00000014067	
ENSGALT00000021647	LOXL3	ENSGALT00000014145	MAPKBP 1
ENSGALT00000023454		ENSGALT00000008241	FAM96B
ENSGALT00000015678		ENSGALT00000008251	
ENSGALT00000015691	EMC7	ENSGALT00000014555	EHD4
ENSGALT00000015856	FAM98B	ENSGALT00000014672	TMEM87 A
ENSGALT00000015940		ENSGALT00000039221	
ENSGALT00000016005		ENSGALT00000008447	
ENSGALT00000028346		ENSGALT00000008459	
ENSGALT00000009065	KLHL36	ENSGALT00000008465	
ENSGALT00000009174	ZDHHC7	ENSGALT00000014708	
ENSGALT00000009186	MTHFSD	ENSGALT00000039170	
ENSGALT00000009243		ENSGALT00000014809	
ENSGALT00000009240		ENSGALT00000032634	
ENSGALT00000009231		ENSGALT00000014837	
ENSGALT00000016236	ARHGAP5	ENSGALT00000015071	TMEM62
ENSGALT00000016285		ENSGALT00000015102	ELMSAN 1
ENSGALT00000016289	BAZ1A	ENSGALT00000038149	
ENSGALT00000016327	SRP54	ENSGALT00000015117	
ENSGALT00000009283		ENSGALT00000015198	
ENSGALT00000016326		ENSGALT00000029669	HOXC10
ENSGALT00000016334	PPP2R3C	ENSGALT00000021972	ATMIN
ENSGALT00000016344		ENSGALT00000015206	
ENSGALT00000009408		ENSGALT00000015283	
ENSGALT00000009439		ENSGALT00000000388	PCNX
ENSGALT00000016500	SEC23A	ENSGALT00000022454	
ENSGALT00000016505		ENSGALT00000015312	
ENSGALT00000016512		ENSGALT00000015350	KIAA0247
ENSGALT00000016532	PNN	ENSGALT00000015368	
ENSGALT00000016569	FBXO33	ENSGALT00000028069	
ENSGALT00000016576	COQ6	ENSGALT00000015383	GALNT16
ENSGALT00000016585		ENSGALT00000008756	SDR42E1
ENSGALT00000016604	ALDH6A1	ENSGALT00000008821	
ENSGALT00000032565		ENSGALT00000015588	
ENSGALT00000022441	TOMM40L	ENSGALT00000033760	
ENSGALT00000016641		ENSGALT00000008832	
ENSGALT00000016690	LTBP2	ENSGALT00000021647	LOXL3

ENSGALT00000010149	GALNS	ENSGALT00000000596	AP2S1
ENSGALT00000016704	AREL1	ENSGALT00000015631	
ENSGALT00000010048	ACSF3	ENSGALT00000015663	EIF2AK4
ENSGALT00000010046	ANKRD11	ENSGALT00000015691	EMC7
ENSGALT00000016756		ENSGALT00000015856	FAM98B
ENSGALT00000016763	MLH3	ENSGALT00000015940	
ENSGALT00000016792	NEK9	ENSGALT00000015948	
ENSGALT00000037707		ENSGALT00000015965	ZNF770
ENSGALT00000016850	GPATCH2L	ENSGALT00000016005	
ENSGALT00000016888	ESRRB	ENSGALT00000016127	
ENSGALT00000016892	VASH1	ENSGALT00000028346	
ENSGALT00000016899		ENSGALT00000016210	HEATR5A
ENSGALT00000016918	IRF2BPL	ENSGALT00000009174	ZDHHC7
ENSGALT00000016923		ENSGALT00000009186	MTHFSD
ENSGALT00000017033	AHSA1	ENSGALT00000009243	
ENSGALT00000000074		ENSGALT00000009240	
ENSGALT00000017069		ENSGALT00000009231	
ENSGALT00000034401		ENSGALT00000009226	EMC8
ENSGALT00000000910	CIRH1A	ENSGALT00000016236	ARHGAP5
ENSGALT00000004641		ENSGALT00000016285	
ENSGALT00000004784	MKL2	ENSGALT00000016289	BAZ1A
ENSGALT00000017215	STON2	ENSGALT00000016327	SRP54
ENSGALT00000017221	SEL1L	ENSGALT00000009283	
ENSGALT00000004911	BFAR	ENSGALT00000016326	
ENSGALT00000000947		ENSGALT00000016334	PPP2R3C
ENSGALT00000004992	TXNDC11	ENSGALT00000016344	
ENSGALT00000017348	TTC8	ENSGALT00000016353	
ENSGALT00000005338		ENSGALT00000009408	
ENSGALT00000017411	C14orf159	ENSGALT00000009439	
ENSGALT00000017485		ENSGALT00000016500	SEC23A
ENSGALT00000017502	TRIP11	ENSGALT00000016505	
ENSGALT00000037601		ENSGALT00000016512	
ENSGALT00000005479		ENSGALT00000016532	PNN
ENSGALT00000017577	LGMN	ENSGALT00000016569	FBXO33
ENSGALT00000005507	LMTK2	ENSGALT00000010154	PIEZO1
ENSGALT00000005546	TECPR1	ENSGALT00000016576	COQ6
ENSGALT00000001181	IST1	ENSGALT00000016585	
ENSGALT00000001245		ENSGALT00000022384	CDT1
ENSGALT00000001289	PHLPP2	ENSGALT00000016604	ALDH6A1

ENSGALT00000028074		ENSGALT00000032565	
ENSGALT00000017630	BTBD7	ENSGALT00000022441	TOMM40L
ENSGALT00000001325		ENSGALT00000016690	LTBP2
ENSGALT00000018063	ATG2B	ENSGALT00000010149	GALNS
ENSGALT00000037556		ENSGALT00000016704	AREL1
ENSGALT00000005765	FAM20C	ENSGALT00000016710	FCF1
ENSGALT00000005824	PRKAR1B	ENSGALT00000010046	ANKRD11
ENSGALT00000018196		ENSGALT00000016756	
ENSGALT00000005837	HEATR2	ENSGALT00000016763	MLH3
ENSGALT00000018280		ENSGALT00000016792	NEK9
ENSGALT00000018296	SLC25A29	ENSGALT00000016804	BATF
ENSGALT00000006001	SUN1	ENSGALT00000037707	
ENSGALT00000018316	WARS	ENSGALT00000016850	GPATCH2L
ENSGALT00000018342		ENSGALT00000016888	ESRRB
ENSGALT00000034244	ADAP1	ENSGALT00000016892	VASH1
ENSGALT00000034197	C7orf50	ENSGALT00000016899	
ENSGALT00000006456		ENSGALT00000016918	IRF2BPL
ENSGALT00000006473	MICALL2	ENSGALT00000016923	
ENSGALT00000006529	INTS1	ENSGALT00000016945	TMEM63C
ENSGALT00000018521	HSP90AA1	ENSGALT00000016973	POMT2
ENSGALT00000018539	WDR20	ENSGALT00000016986	
ENSGALT00000018544	ZNF839	ENSGALT00000017033	AHSA1
ENSGALT00000018551		ENSGALT00000000074	
ENSGALT00000018553	TECPR2	ENSGALT00000017069	
ENSGALT00000018558	ANKRD9	ENSGALT00000034401	
ENSGALT00000018569	RCOR1	ENSGALT00000000898	
ENSGALT00000006646	GAA	ENSGALT00000000910	CIRH1A
ENSGALT00000006656		ENSGALT00000004641	
ENSGALT00000023325	EVI5L	ENSGALT00000017215	STON2
ENSGALT00000032501		ENSGALT00000017221	SEL1L
ENSGALT00000006743		ENSGALT00000004911	BFAR
ENSGALT00000040967		ENSGALT00000000947	
ENSGALT00000006768	SNX8	ENSGALT00000004992	TXNDC11
ENSGALT00000018666	TNFAIP2	ENSGALT00000017359	TDP1
ENSGALT00000018671		ENSGALT00000005338	
ENSGALT00000018692		ENSGALT00000017411	C14orf159

ENSGALT00000018759	MARK3	ENSGALT00000017485	
ENSGALT00000006809		ENSGALT00000017502	TRIP11
ENSGALT00000006863	TTYH3	ENSGALT00000037601	
ENSGALT00000018795	APOPT1	ENSGALT00000005479	
ENSGALT00000018805		ENSGALT00000017577	LGMN
ENSGALT00000018983	ADSSL1	ENSGALT00000005546	TECPR1
ENSGALT00000006969	GNA12	ENSGALT00000001181	IST1
ENSGALT00000037448		ENSGALT00000001245	
ENSGALT00000037443		ENSGALT00000001289	PHLPP2
ENSGALT00000023630	NR2F6	ENSGALT00000028074	
ENSGALT00000019108		ENSGALT00000017630	BTBD7
ENSGALT00000037436		ENSGALT00000001325	
ENSGALT00000019114		ENSGALT00000018063	ATG2B
ENSGALT00000019132	BRF1	ENSGALT00000018066	
ENSGALT00000019138	BTBD6	ENSGALT00000001342	
ENSGALT00000007098	FOXK1	ENSGALT00000037556	
ENSGALT00000019340		ENSGALT00000005765	FAM20C
ENSGALT00000019364		ENSGALT00000005824	PRKAR1B
ENSGALT00000007215	MMD2	ENSGALT00000018181	HHIPL1
ENSGALT00000007252		ENSGALT00000018196	
ENSGALT00000019409	SLC38A6	ENSGALT00000005837	HEATR2
ENSGALT00000008230	IVNS1ABP	ENSGALT00000018280	
ENSGALT00000007259		ENSGALT00000006001	SUN1
ENSGALT00000019443	PPM1A	ENSGALT00000018316	WARS
ENSGALT00000019451	PCNXL4	ENSGALT00000018342	
ENSGALT00000015673		ENSGALT00000034197	C7orf50
ENSGALT00000019598		ENSGALT00000006456	
ENSGALT00000007374		ENSGALT00000004936	ZFAND2A
ENSGALT00000037347		ENSGALT00000006473	MICALL2
ENSGALT00000007417	SMURF1	ENSGALT00000006529	INTS1
ENSGALT00000000238		ENSGALT00000018521	HSP90AA1
ENSGALT00000019620		ENSGALT00000018551	
ENSGALT00000019657		ENSGALT00000018553	TECPR2
ENSGALT00000000139		ENSGALT00000018569	RCOR1
ENSGALT00000019761	NAA30	ENSGALT00000006646	GAA
ENSGALT00000007484	PDAP1	ENSGALT00000006656	
ENSGALT00000019771		ENSGALT00000023325	EVI5L
ENSGALT00000007493	BUD31	ENSGALT00000032501	
ENSGALT00000019886		ENSGALT00000006743	

ENSGALT00000019926	SAMD4A	ENSGALT00000040967	
ENSGALT00000019930	CGRRF1	ENSGALT00000006768	SNX8
ENSGALT00000007505		ENSGALT00000018666	TNFAIP2
ENSGALT00000019945		ENSGALT00000018671	
ENSGALT00000007510		ENSGALT00000018692	
ENSGALT00000019972		ENSGALT00000018759	MARK3
ENSGALT00000007592	DHRS7B	ENSGALT00000006809	
ENSGALT00000020029		ENSGALT00000006863	TTYH3
ENSGALT00000007656	MPRIP	ENSGALT00000018785	TRMT61A
ENSGALT00000020041	VCPKMT	ENSGALT00000018795	APOPT1
ENSGALT00000020050	SOS2	ENSGALT00000032496	
ENSGALT00000020072	L2HGDH	ENSGALT00000018983	ADSSL1
ENSGALT00000020080		ENSGALT00000006938	BRAT1
ENSGALT00000020133	MAP4K5	ENSGALT00000006969	GNA12
ENSGALT00000007690		ENSGALT00000037448	
ENSGALT00000007721		ENSGALT00000037443	
ENSGALT00000007729	NT5M	ENSGALT00000037442	ZBTB42
ENSGALT00000007745		ENSGALT00000023630	NR2F6
ENSGALT00000007783		ENSGALT00000019108	
ENSGALT00000020162	ATL1	ENSGALT00000037436	
ENSGALT00000007838		ENSGALT00000019114	
ENSGALT00000000234		ENSGALT00000019132	BRF1
ENSGALT00000020164	SAV1	ENSGALT00000019138	BTBD6
ENSGALT00000007886	GID4	ENSGALT00000019249	
ENSGALT00000020205	FRMD6	ENSGALT00000007098	FOXK1
ENSGALT00000007980	ALKBH5	ENSGALT00000019277	SYNE2
ENSGALT00000020241	NID2	ENSGALT00000019302	
ENSGALT00000020294	GNPNAT1	ENSGALT00000019340	
ENSGALT00000020309	FERMT2	ENSGALT00000019364	
ENSGALT00000020354	MIS18BP1	ENSGALT00000007215	MMD2
ENSGALT00000008014	MIEF2	ENSGALT00000007252	
ENSGALT00000020376	PRPF39	ENSGALT00000019409	SLC38A6
ENSGALT00000020402	KLHL28	ENSGALT00000019427	TRMT5
ENSGALT00000040656	SMCR8	ENSGALT00000007259	
ENSGALT00000008093	SHMT1	ENSGALT00000019443	PPM1A
ENSGALT00000000084		ENSGALT00000019449	DHRS7
ENSGALT00000000149		ENSGALT00000015673	
ENSGALT00000008119		ENSGALT00000034001	
ENSGALT00000019575		ENSGALT00000019598	
ENSGALT00000031517		ENSGALT00000007374	
ENSGALT00000003794		ENSGALT00000037347	

ENSGALT00000031518		ENSGALT00000007417	SMURF1
ENSGALT00000008174	B9D1	ENSGALT00000000238	
ENSGALT00000000081		ENSGALT00000019620	
ENSGALT00000041141		ENSGALT00000019657	
ENSGALT00000008468	LMF1	ENSGALT00000000139	
ENSGALT00000040739	LAS1L	ENSGALT00000019761	NAA30
ENSGALT00000008614		ENSGALT00000007484	PDAP1
ENSGALT00000032414	VSIG4	ENSGALT00000019771	
ENSGALT00000008671		ENSGALT00000007493	BUD31
ENSGALT00000007320		ENSGALT00000019842	ATG14
ENSGALT00000008694		ENSGALT00000019869	DLGAP5
ENSGALT00000007313		ENSGALT00000019886	
ENSGALT00000007301	OPHN1	ENSGALT00000019926	SAMD4A
ENSGALT00000008758		ENSGALT00000019930	CGRRF1
ENSGALT00000007294	YIPF6	ENSGALT00000007505	
ENSGALT00000008816		ENSGALT00000019945	
ENSGALT00000007282	STARD8	ENSGALT00000007510	
ENSGALT00000007137	EDA	ENSGALT00000007566	
ENSGALT00000008954	GFER	ENSGALT00000007592	DHRS7B
ENSGALT00000009002		ENSGALT00000019990	KLHDC1
ENSGALT00000009013		ENSGALT00000032448	
ENSGALT00000040650		ENSGALT00000020029	
ENSGALT00000006629		ENSGALT00000007656	MPRIP
ENSGALT00000006532		ENSGALT00000020041	VCPKMT
ENSGALT00000006500		ENSGALT00000020050	SOS2
ENSGALT00000006376		ENSGALT00000020072	L2HGDH
ENSGALT00000015015		ENSGALT00000020133	MAP4K5
ENSGALT00000022940		ENSGALT00000007690	
ENSGALT00000040562		ENSGALT00000007721	
ENSGALT00000009446	MLST8	ENSGALT00000007729	NT5M
ENSGALT00000006314	ZDHHC9	ENSGALT00000007745	
ENSGALT00000009467		ENSGALT00000007783	
ENSGALT00000000216	ZNF638	ENSGALT00000000237	
ENSGALT00000009586	ARHGAP17	ENSGALT00000020162	ATL1
ENSGALT00000006167	PIN4	ENSGALT00000007838	
ENSGALT00000006133		ENSGALT00000020164	SAV1
ENSGALT00000009734		ENSGALT00000020196	
ENSGALT00000009821		ENSGALT00000020205	FRMD6
ENSGALT00000009868		ENSGALT00000007980	ALKBH5
ENSGALT00000007708	HDAC8	ENSGALT00000020241	NID2
ENSGALT00000007756		ENSGALT00000020248	TXNDC16

ENSGALT00000007851		ENSGALT00000020294	GNPNAT1
ENSGALT00000000161		ENSGALT00000020309	FERMT2
ENSGALT00000007950		ENSGALT00000020311	DDHD1
ENSGALT00000010366	KCTD5	ENSGALT00000020354	MIS18BP1
ENSGALT00000007959	TIMM8A	ENSGALT00000008014	MIEF2
ENSGALT00000010394		ENSGALT00000020376	PRPF39
ENSGALT00000010414	UNKL	ENSGALT00000020402	KLHL28
ENSGALT00000010426		ENSGALT00000040656	SMCR8
ENSGALT00000010464	KIAA0430	ENSGALT00000008093	SHMT1
ENSGALT00000010556		ENSGALT00000000084	
ENSGALT00000008098		ENSGALT00000000149	
ENSGALT00000008105		ENSGALT00000008119	
ENSGALT00000010888		ENSGALT00000019575	
ENSGALT00000008128		ENSGALT00000031517	
ENSGALT00000010969		ENSGALT00000003794	
ENSGALT00000011038	SMG1	ENSGALT00000031518	
ENSGALT00000011107		ENSGALT00000008174	B9D1
ENSGALT00000008838		ENSGALT00000041141	
ENSGALT00000011312	EEF2K	ENSGALT00000008468	LMF1
ENSGALT00000011343	POLR3E	ENSGALT00000007513	ZC4H2
ENSGALT00000011412		ENSGALT00000040739	LAS1L
ENSGALT00000009079	FOXO4	ENSGALT00000008614	
ENSGALT00000011481		ENSGALT00000032414	VSIG4
ENSGALT00000009239		ENSGALT00000008671	
ENSGALT00000039834	C16orf72	ENSGALT00000008694	
ENSGALT00000032399		ENSGALT00000007313	
ENSGALT00000011867	ABAT	ENSGALT00000007301	OPHN1
ENSGALT00000000404		ENSGALT00000007294	YIPF6
ENSGALT00000009488		ENSGALT00000008816	
ENSGALT00000009540		ENSGALT00000007282	STARD8
ENSGALT00000009621		ENSGALT00000007137	EDA
ENSGALT00000012090		ENSGALT00000008918	
ENSGALT00000009683	DOCK11	ENSGALT00000008954	GFER
ENSGALT00000012148		ENSGALT00000009002	
ENSGALT00000012273	C7orf26	ENSGALT00000006667	
ENSGALT00000012315	MGRN1	ENSGALT00000006629	
ENSGALT00000012323	UBALD1	ENSGALT00000006532	
ENSGALT00000012346		ENSGALT00000006500	
ENSGALT00000012358	HMOX2	ENSGALT00000023131	
ENSGALT00000009795		ENSGALT00000006401	MARS2

ENSGALT00000009812	GPC4	ENSGALT00000006376	
ENSGALT00000012459		ENSGALT00000015015	
ENSGALT00000009861	FAM122B	ENSGALT00000022940	
ENSGALT00000009889		ENSGALT00000040562	
ENSGALT00000009895	MOSPD1	ENSGALT00000023322	
ENSGALT00000022056	MCOLN1	ENSGALT00000009446	MLST8
ENSGALT00000012620		ENSGALT00000006314	ZDHHC9
ENSGALT00000012623		ENSGALT00000009467	
ENSGALT00000009976	SLC9A6	ENSGALT00000000216	ZNF638
ENSGALT00000009030	RARA	ENSGALT00000006167	PIN4
ENSGALT00000012696	DNAJA3	ENSGALT00000006133	
ENSGALT00000014661	SMG5	ENSGALT00000009734	
ENSGALT00000010117	KIRREL	ENSGALT00000009821	
ENSGALT00000000945	ETV3	ENSGALT00000009864	
ENSGALT00000010487		ENSGALT00000009868	
ENSGALT00000021621	PRCC	ENSGALT00000009913	GGA2
ENSGALT00000010594	MCF2	ENSGALT00000007708	HDAC8
ENSGALT00000021620	MRPL24	ENSGALT00000007756	
ENSGALT00000028290		ENSGALT00000007851	
ENSGALT00000004228	DECR2	ENSGALT00000007937	GLA
ENSGALT00000023483		ENSGALT00000010199	NSMCE1
ENSGALT00000004086	MSS51	ENSGALT00000007950	
ENSGALT00000021963	S100A13	ENSGALT00000010366	KCTD5
ENSGALT00000001529		ENSGALT00000007959	TIMM8A
ENSGALT00000003862		ENSGALT00000010394	
ENSGALT00000040760		ENSGALT00000010426	
ENSGALT00000034473		ENSGALT00000010464	KIAA0430
ENSGALT00000014892		ENSGALT00000010556	
ENSGALT00000003641		ENSGALT00000008098	
ENSGALT00000003589	SPSB3	ENSGALT00000008105	
ENSGALT00000003298	SEC14L5	ENSGALT00000010888	
ENSGALT00000004258	ATP8B2	ENSGALT00000008128	
ENSGALT00000011011		ENSGALT00000010969	
ENSGALT00000003142	CAPN15	ENSGALT00000011038	SMG1
ENSGALT00000011029		ENSGALT00000011107	
ENSGALT00000003054	ABCA3	ENSGALT00000008838	
ENSGALT00000022043	TPM3	ENSGALT00000011230	C16orf52
ENSGALT00000023591	ASH1L	ENSGALT00000011312	EEF2K
ENSGALT00000015109	RNPS1	ENSGALT00000011343	POLR3E
ENSGALT00000015029		ENSGALT00000011412	
ENSGALT00000023613		ENSGALT00000009079	FOXO4
ENSGALT00000015002		ENSGALT00000011481	

ENSGALT00000032362		ENSGALT00000009229	
ENSGALT00000023620		ENSGALT00000009239	
ENSGALT00000029552		ENSGALT00000039834	C16orf72
ENSGALT00000017111		ENSGALT00000011867	ABAT
ENSGALT00000001109		ENSGALT00000000404	
ENSGALT00000001122		ENSGALT00000009488	
ENSGALT00000002849	ROGDI	ENSGALT00000009540	
ENSGALT00000040574		ENSGALT00000009621	
ENSGALT00000003152	GPRC5B	ENSGALT00000009683	DOCK11
ENSGALT00000029798	KNOP1	ENSGALT00000036188	
ENSGALT00000003559	DCUN1D3	ENSGALT00000012148	
ENSGALT00000011713		ENSGALT00000012543	
ENSGALT00000019489		ENSGALT00000012273	C7orf26
ENSGALT00000040550	PI4KB	ENSGALT00000039617	
ENSGALT00000001276		ENSGALT00000012315	MGRN1
ENSGALT00000015681	KCTD12	ENSGALT00000012323	UBALD1
ENSGALT00000000505		ENSGALT00000012346	
ENSGALT00000012247		ENSGALT00000012358	HMOX2
ENSGALT00000003096	PRUNE	ENSGALT00000009795	
ENSGALT00000012271		ENSGALT00000009812	GPC4
ENSGALT00000012291	AMER1	ENSGALT00000012459	
ENSGALT00000012370		ENSGALT00000009861	FAM122B
ENSGALT00000012409		ENSGALT00000009889	
ENSGALT00000012454		ENSGALT00000009895	MOSPD1
ENSGALT00000041392	POGZ	ENSGALT00000022056	MCOLN1
ENSGALT00000012493		ENSGALT00000012620	
ENSGALT00000039451		ENSGALT00000012623	
ENSGALT00000012569		ENSGALT00000009976	SLC9A6
ENSGALT00000012638		ENSGALT00000012696	DNAJA3
ENSGALT00000012629	ABCB7	ENSGALT00000014661	SMG5
ENSGALT00000012737	ATRX	ENSGALT00000021945	
ENSGALT00000012759		ENSGALT00000010117	KIRREL
ENSGALT00000012893		ENSGALT00000010428	
ENSGALT00000012996	CAPN6	ENSGALT00000000945	ETV3
ENSGALT00000013077	PAK3	ENSGALT00000010487	
ENSGALT00000013105		ENSGALT00000021621	PRCC
ENSGALT00000013107	AMMECR1	ENSGALT00000010594	MCF2
ENSGALT00000013109		ENSGALT00000028290	
ENSGALT00000013126	ACSL4	ENSGALT00000004228	DECR2
ENSGALT00000013221	COL4A5	ENSGALT00000023483	
ENSGALT00000013217	COL4A5	ENSGALT00000004086	MSS51
ENSGALT00000012265	NIT1	ENSGALT00000021963	S100A13

ENSGALT00000013447	COL4A6	ENSGALT00000001529	
ENSGALT00000013485		ENSGALT00000003862	
ENSGALT00000013668	VBP1	ENSGALT00000034473	
ENSGALT00000013858	THOC2	ENSGALT00000014892	
ENSGALT00000013866		ENSGALT00000003641	
ENSGALT00000013871		ENSGALT00000003589	SPSB3
ENSGALT00000013942	CUL4B	ENSGALT00000011109	SHC1
ENSGALT00000013962		ENSGALT00000003298	SEC14L5
ENSGALT00000014012	TMEM255A	ENSGALT00000003249	NAGPA
ENSGALT00000039073	UPF3B	ENSGALT00000004258	ATP8B2
ENSGALT00000039072		ENSGALT00000011011	
ENSGALT00000014085		ENSGALT00000003142	CAPN15
ENSGALT00000014088		ENSGALT00000011029	
ENSGALT00000014093	CXorf56	ENSGALT00000003054	ABCA3
ENSGALT00000014132	SLC25A43	ENSGALT00000022043	TPM3
ENSGALT00000039050		ENSGALT00000023591	ASH1L
ENSGALT00000014414	FAM199X	ENSGALT00000011214	POF1B
ENSGALT00000014623	CXorf57	ENSGALT00000011305	
ENSGALT00000014716	TBC1D8B	ENSGALT00000015124	TELO2
ENSGALT00000014753		ENSGALT00000015109	RNPS1
ENSGALT00000014767	CD99L2	ENSGALT00000023607	SYT11
ENSGALT00000014782	MTMR1	ENSGALT00000015029	
ENSGALT00000014807	MTM1	ENSGALT00000023613	
ENSGALT00000009805		ENSGALT00000015002	
ENSGALT00000029631	ATF1	ENSGALT00000032362	
ENSGALT00000009909	DIP2B	ENSGALT00000023620	
ENSGALT00000014996		ENSGALT00000017111	
ENSGALT00000009980	CERS5	ENSGALT00000001109	
ENSGALT00000015038		ENSGALT00000002849	ROGDI
ENSGALT00000015067	PLRG1	ENSGALT00000040574	
ENSGALT00000015084		ENSGALT00000003152	GPRC5B
ENSGALT00000032288	MAP9	ENSGALT00000029798	KNOP1
ENSGALT00000015246	GUCY1B3	ENSGALT00000003334	THUMPD1
ENSGALT00000028268	GPD1	ENSGALT00000003559	DCUN1D3
ENSGALT00000010024	SMARCD1	ENSGALT00000011713	
ENSGALT00000015275		ENSGALT00000019489	
ENSGALT00000015333	FAM198B	ENSGALT00000003923	UQCRC2
ENSGALT00000010260	RAPGEF3	ENSGALT00000040550	PI4KB
ENSGALT00000015372		ENSGALT00000032336	NSDHL
ENSGALT00000015382	PPID	ENSGALT00000001276	

ENSGALT00000015423	RAPGEF2	ENSGALT00000012141	
ENSGALT00000009619		ENSGALT00000012173	
ENSGALT00000015511	TMEM192	ENSGALT00000015681	KCTD12
ENSGALT00000015566		ENSGALT00000012247	
ENSGALT00000015714	PALLD	ENSGALT00000003096	PRUNE
ENSGALT00000019766		ENSGALT00000012271	
ENSGALT00000019115	ARHGAP35	ENSGALT00000012279	
ENSGALT00000015774	SH3RF1	ENSGALT00000012291	AMER1
ENSGALT00000015813	CBR4	ENSGALT00000012370	
ENSGALT00000015841	PCDH18	ENSGALT00000012409	
ENSGALT00000015887		ENSGALT00000012454	
ENSGALT00000015926		ENSGALT00000041392	POGZ
ENSGALT00000015941	SETD7	ENSGALT00000012483	
ENSGALT00000032252		ENSGALT00000012493	
ENSGALT00000016039	RNF150	ENSGALT00000039451	
ENSGALT00000016070	USP38	ENSGALT00000012569	
ENSGALT00000016095	GAB1	ENSGALT00000012638	
ENSGALT00000016203	OTUD4	ENSGALT00000012629	ABCB7
ENSGALT00000016214	MMAA	ENSGALT00000012737	ATRX
ENSGALT00000016226	LSM6	ENSGALT00000012759	
ENSGALT00000038572		ENSGALT00000012893	
ENSGALT00000016278	PRMT10	ENSGALT00000012996	CAPN6
ENSGALT00000016302		ENSGALT00000013077	PAK3
ENSGALT00000016385	SH3D19	ENSGALT00000013105	
ENSGALT00000016422	PET112	ENSGALT00000013107	AMMECR 1
ENSGALT00000016433		ENSGALT00000013109	
ENSGALT00000016446	SCLT1	ENSGALT00000013126	ACSL4
ENSGALT00000016475		ENSGALT00000013221	COL4A5
ENSGALT00000016483	HGSNAT	ENSGALT00000013217	COL4A5
ENSGALT00000016490		ENSGALT00000013447	COL4A6
ENSGALT00000016578	MFSD8	ENSGALT00000013485	
ENSGALT00000016607	JADE1	ENSGALT00000013668	VBP1
ENSGALT00000016708		ENSGALT00000013858	THOC2
ENSGALT00000016727		ENSGALT00000013866	
ENSGALT00000016820	CNOT6L	ENSGALT00000013871	
ENSGALT00000016846	CCNG2	ENSGALT00000013942	CUL4B
ENSGALT00000016897	FAM13A	ENSGALT00000013962	
ENSGALT00000016908		ENSGALT00000039082	
ENSGALT00000017134	ETNPPL	ENSGALT00000014012	TMEM25 5A
ENSGALT00000017138		ENSGALT00000039073	UPF3B

ENSGALT00000017142		ENSGALT00000039072	
ENSGALT00000025976		ENSGALT00000014085	
ENSGALT00000017158	PAPSS1	ENSGALT00000014088	
ENSGALT00000017210	INTS12	ENSGALT00000014093	CXorf56
ENSGALT00000017222	PPA2	ENSGALT00000014132	SLC25A4 3
ENSGALT00000017251		ENSGALT00000039050	
ENSGALT00000017270		ENSGALT00000014414	FAM199X
ENSGALT00000017294		ENSGALT00000014447	IL1RAPL 2
ENSGALT00000017342	RWDD4	ENSGALT00000014623	CXorf57
ENSGALT00000017346	CDKN2AIP	ENSGALT00000014716	TBC1D8B
ENSGALT00000017366		ENSGALT00000014753	
ENSGALT00000017488	SAP30	ENSGALT00000014763	
ENSGALT00000017505		ENSGALT00000014767	CD99L2
ENSGALT00000017617	ASB5	ENSGALT00000014782	MTMR1
ENSGALT00000017629	VEGFC	ENSGALT00000014807	MTM1
ENSGALT00000017682		ENSGALT00000009805	
ENSGALT00000017688	G3BP2	ENSGALT00000029631	ATF1
ENSGALT00000017693	BMP2K	ENSGALT00000029963	
ENSGALT00000017703	ANTXR2	ENSGALT00000009909	DIP2B
ENSGALT00000017734	RASGEF1B	ENSGALT00000009960	LIMA1
ENSGALT00000017784	NUDT9	ENSGALT00000009980	CERS5
ENSGALT00000017992	AFF1	ENSGALT00000015038	
ENSGALT00000018134	ARHGAP24	ENSGALT00000015067	PLRG1
ENSGALT00000018144		ENSGALT00000015084	
ENSGALT00000018147	LIN54	ENSGALT00000032288	MAP9
ENSGALT00000018201		ENSGALT00000015233	GUCY1A 3
ENSGALT00000018225	ENOPH1	ENSGALT00000015246	GUCY1B 3
ENSGALT00000038016		ENSGALT00000028268	GPD1
ENSGALT00000021658	ARHGEF11	ENSGALT00000010024	SMARCD 1
ENSGALT00000018245		ENSGALT00000015275	
ENSGALT00000001368		ENSGALT00000010135	RACGAP 1
ENSGALT00000029722	RNF41	ENSGALT00000010204	
ENSGALT00000023097		ENSGALT00000015333	FAM198B
ENSGALT00000018298		ENSGALT00000010260	RAPGEF 3
ENSGALT00000022282		ENSGALT00000032282	
ENSGALT00000022286	SOCS7	ENSGALT00000015372	

ENSGALT00000018386		ENSGALT00000015382	PPID
ENSGALT00000018500	TECRL	ENSGALT00000009619	
ENSGALT00000040660		ENSGALT00000015511	TMEM19 2
ENSGALT00000032173	NOA1	ENSGALT00000015566	
ENSGALT00000018580	REST	ENSGALT00000015714	PALLD
ENSGALT00000001580		ENSGALT00000015738	C4orf27
ENSGALT00000018588		ENSGALT00000019115	ARHGAP 35
ENSGALT00000001793	NCOA6	ENSGALT00000015813	CBR4
ENSGALT00000001850	PIGU	ENSGALT00000015841	PCDH18
ENSGALT00000041395	LEPROTL1	ENSGALT00000015887	
ENSGALT00000018652		ENSGALT00000015926	
ENSGALT00000018662	MFHAS1	ENSGALT00000015941	SETD7
ENSGALT00000018669		ENSGALT00000015954	MAML3
ENSGALT00000002165		ENSGALT00000032252	
ENSGALT00000001556	MTMR4	ENSGALT00000016039	RNF150
ENSGALT00000002265	NDRG3	ENSGALT00000016070	USP38
ENSGALT00000018681	CCNI	ENSGALT00000016095	GAB1
ENSGALT00000002314	PHF20	ENSGALT00000016203	OTUD4
ENSGALT00000002335		ENSGALT00000016214	MMAA
ENSGALT00000018738	SCARB2	ENSGALT00000038572	
ENSGALT00000002384		ENSGALT00000016278	PRMT10
ENSGALT00000001603	ELN	ENSGALT00000016293	ARHGAP 10
ENSGALT00000002696		ENSGALT00000016302	
ENSGALT00000001666		ENSGALT00000016385	SH3D19
ENSGALT00000001706	WBSCR17	ENSGALT00000016422	PET112
ENSGALT00000021722		ENSGALT00000016433	
ENSGALT00000021729	NXNL1	ENSGALT00000016446	SCLT1
ENSGALT00000021883		ENSGALT00000016475	
ENSGALT00000018826	SDAD1	ENSGALT00000016483	HGSNAT
ENSGALT00000018860	RUFY3	ENSGALT00000016490	
ENSGALT00000018869	GRSF1	ENSGALT00000032235	
ENSGALT00000029792		ENSGALT00000016555	
ENSGALT00000021915	ATP13A1	ENSGALT00000016572	PLK4
ENSGALT00000002729	CEP250	ENSGALT00000016578	MFSD8
ENSGALT00000001766		ENSGALT00000016607	JADE1
ENSGALT00000002775		ENSGALT00000016708	
ENSGALT00000001925	GTF2I	ENSGALT00000016727	
ENSGALT00000019023	ANKRD17	ENSGALT00000016753	
ENSGALT00000002799		ENSGALT00000016846	CCNG2

ENSGALT00000003157	AHCY	ENSGALT00000016897	FAM13A
ENSGALT00000001926	GTF2IRD1	ENSGALT00000017134	ETNPPL
ENSGALT00000019202	UBA6	ENSGALT00000017138	
ENSGALT00000002942	MAP1LC3A	ENSGALT00000017142	
ENSGALT00000002964	DYNLRB1	ENSGALT00000025976	
ENSGALT00000003027	ITCH	ENSGALT00000017158	PAPSS1
ENSGALT00000003180		ENSGALT00000017190	
ENSGALT00000040421	RALY	ENSGALT00000017210	INTS12
ENSGALT00000004812	PXMP4	ENSGALT00000017222	PPA2
ENSGALT00000004975		ENSGALT00000017251	
ENSGALT00000005318	ACSS2	ENSGALT00000017270	
ENSGALT00000019307	ANKRD50	ENSGALT00000017294	
ENSGALT00000019309		ENSGALT00000017317	ENPP6
ENSGALT00000019311	NUDT6	ENSGALT00000017342	RWDD4
ENSGALT00000040447		ENSGALT00000017346	CDKN2AIP
ENSGALT00000002223	ANKFY1	ENSGALT00000017366	
ENSGALT00000005391	SOGA1	ENSGALT00000017483	
ENSGALT00000002242	CYB5D2	ENSGALT00000017488	SAP30
ENSGALT00000002350	ZZEF1	ENSGALT00000017617	ASB5
ENSGALT00000019560		ENSGALT00000017629	VEGFC
ENSGALT00000019566	SYNPO2	ENSGALT00000017682	
ENSGALT00000019581	SEC24D	ENSGALT00000017688	G3BP2
ENSGALT00000002517	DERL2	ENSGALT00000017693	BMP2K
ENSGALT00000002525	C1QBP	ENSGALT00000017695	PAQR3
ENSGALT00000037701		ENSGALT00000017703	ANTXR2
ENSGALT00000019661		ENSGALT00000017784	NUDT9
ENSGALT00000005832		ENSGALT00000017992	AFF1
ENSGALT00000006112		ENSGALT00000018134	ARHGAP24
ENSGALT00000006220	PIGT	ENSGALT00000018144	
ENSGALT00000019679	LARP7	ENSGALT00000018147	LIN54
ENSGALT00000006261		ENSGALT00000018201	
ENSGALT00000006265		ENSGALT00000018225	ENOPH1
ENSGALT00000002828	SH2B2	ENSGALT00000038016	
ENSGALT00000006521		ENSGALT00000021658	ARHGEF11
ENSGALT00000006537	TOMM34	ENSGALT00000001368	
ENSGALT00000019895	PLA2G12A	ENSGALT00000029722	RNF41
ENSGALT00000037662		ENSGALT00000023097	
ENSGALT00000002880		ENSGALT00000018298	
ENSGALT00000019968	TSPAN5	ENSGALT00000022282	

ENSGALT00000006588		ENSGALT00000022286	SOCS7
ENSGALT00000006710		ENSGALT00000018386	
ENSGALT00000019988		ENSGALT00000018500	TECRL
ENSGALT00000006749		ENSGALT00000040660	
ENSGALT00000039955	JPH2	ENSGALT00000032173	NOA1
ENSGALT00000002944	DTX2	ENSGALT00000001580	
ENSGALT00000003016	MDH2	ENSGALT00000018588	
ENSGALT00000007275	NCOA3	ENSGALT00000001793	NCOA6
ENSGALT00000007499	CSE1L	ENSGALT00000001850	PIGU
ENSGALT00000003069	TMEM120A	ENSGALT00000041395	LEPROT L1
ENSGALT00000020032	TRMT10A	ENSGALT00000002032	RBL1
ENSGALT00000034233	KCNB1	ENSGALT00000018652	
ENSGALT00000000071		ENSGALT00000018662	MFHAS1
ENSGALT00000000070		ENSGALT00000018669	
ENSGALT00000003192		ENSGALT00000002165	
ENSGALT00000020064	EMCN	ENSGALT00000001556	MTMR4
ENSGALT00000007724	CCNDBP1	ENSGALT00000002265	NDRG3
ENSGALT00000020115	MANBA	ENSGALT00000018681	CCNI
ENSGALT00000020119		ENSGALT00000002314	PHF20
ENSGALT00000008180		ENSGALT00000002335	
ENSGALT00000020161	BDH2	ENSGALT00000002485	
ENSGALT00000003372	PEX12	ENSGALT00000018738	SCARB2
ENSGALT00000008232	LSM14B	ENSGALT00000002384	
ENSGALT00000021880		ENSGALT00000001603	ELN
ENSGALT00000008294	MTG2	ENSGALT00000002696	
ENSGALT00000003484		ENSGALT00000018760	NUP54
ENSGALT00000008344		ENSGALT00000001666	
ENSGALT00000008535	LAMA5	ENSGALT00000001706	WBSCR1 7
ENSGALT00000003612	TMEM132E	ENSGALT00000021722	
ENSGALT00000022040		ENSGALT00000021729	NXNL1
ENSGALT00000041383		ENSGALT00000021883	
ENSGALT00000023517		ENSGALT00000018860	RUFY3
ENSGALT00000022100	FAT1	ENSGALT00000018869	GRSF1
ENSGALT00000009067		ENSGALT00000001731	AUTS2
ENSGALT00000022137		ENSGALT00000021915	ATP13A1
ENSGALT00000003757		ENSGALT00000002729	CEP250
ENSGALT00000009144		ENSGALT00000001766	
ENSGALT00000003859		ENSGALT00000001779	
ENSGALT00000037573		ENSGALT00000002775	
ENSGALT00000032107	PDGFRL	ENSGALT00000001925	GTF2I

ENSGALT00000022171		ENSGALT00000019023	ANKRD17
ENSGALT00000009286		ENSGALT00000002799	
ENSGALT00000009312		ENSGALT00000003157	AHCY
ENSGALT00000022194	VPS37A	ENSGALT00000001926	GTF2IRD1
ENSGALT00000009385		ENSGALT00000019202	UBA6
ENSGALT00000033928		ENSGALT00000002942	MAP1LC3A
ENSGALT00000009494	PCMTD2	ENSGALT00000019246	
ENSGALT00000022308		ENSGALT00000002964	DYNLRB1
ENSGALT00000009656	PRPF6	ENSGALT00000003027	ITCH
ENSGALT00000003916		ENSGALT00000040421	RALY
ENSGALT00000003944		ENSGALT00000004812	PXMP4
ENSGALT00000004011		ENSGALT00000004815	
ENSGALT00000004075	CRCP	ENSGALT00000004840	
ENSGALT00000004087	TPST1	ENSGALT00000004975	
ENSGALT00000004122		ENSGALT00000005318	ACSS2
ENSGALT00000009779	ZBTB46	ENSGALT00000019307	ANKRD50
ENSGALT00000004144	TMEM248	ENSGALT00000019309	
ENSGALT00000036310		ENSGALT00000019311	NUDT6
ENSGALT00000032094	SRD5A3	ENSGALT00000040447	
ENSGALT00000004146	FAM211A	ENSGALT00000002223	ANKFY1
ENSGALT00000004183		ENSGALT00000005391	SOGA1
ENSGALT00000022578	CHIC2	ENSGALT00000002242	CYB5D2
ENSGALT00000004394	PITPNA	ENSGALT00000002350	ZZEF1
ENSGALT00000022602	SCFD2	ENSGALT00000019519	
ENSGALT00000032088	USP46	ENSGALT00000019560	
ENSGALT00000022640		ENSGALT00000019566	SYNPO2
ENSGALT00000022792	DCUN1D4	ENSGALT00000019581	SEC24D
ENSGALT00000009996	SLC52A3	ENSGALT00000019584	
ENSGALT00000000023		ENSGALT00000002517	DERL2
ENSGALT00000009999		ENSGALT00000002525	C1QBP
ENSGALT00000004467	SCARF1	ENSGALT00000037701	
ENSGALT00000022799	OCIAD1	ENSGALT00000019661	
ENSGALT00000010006	TBC1D20	ENSGALT00000005832	
ENSGALT00000004650	PRPF8	ENSGALT00000006112	
ENSGALT00000010020	HM13	ENSGALT00000006220	PIGT
ENSGALT00000022847	SLAIN2	ENSGALT00000035106	RABL5
ENSGALT00000010025		ENSGALT00000019679	LARP7

ENSGALT00000010073		ENSGALT00000006265	
ENSGALT00000010129		ENSGALT00000006521	
ENSGALT00000010138		ENSGALT00000006537	TOMM34
ENSGALT00000010406		ENSGALT00000019895	PLA2G12A
ENSGALT00000004762		ENSGALT00000019897	
ENSGALT00000010607	TM9SF4	ENSGALT00000037662	
ENSGALT00000022980		ENSGALT00000002880	
ENSGALT00000022997		ENSGALT00000019968	TSPAN5
ENSGALT00000004849		ENSGALT00000006588	
ENSGALT00000023042	SLC30A9	ENSGALT00000006631	
ENSGALT00000023043	TMEM33	ENSGALT00000006710	
ENSGALT00000004908	SMG6	ENSGALT00000019988	
ENSGALT00000010635	PLAGL2	ENSGALT00000006749	
ENSGALT00000010675	ASXL1	ENSGALT00000039955	JPH2
ENSGALT00000005087	POLDIP2	ENSGALT00000002944	DTX2
ENSGALT00000023145	SMIM14	ENSGALT00000040248	
ENSGALT00000023144		ENSGALT00000003016	MDH2
ENSGALT00000023136	LIAS	ENSGALT00000007180	
ENSGALT00000010897	CDK5RAP1	ENSGALT00000007309	SULF2
ENSGALT00000010917		ENSGALT00000007499	CSE1L
ENSGALT00000029529	KLHL5	ENSGALT00000003069	TMEM120A
ENSGALT00000000233	KLF3	ENSGALT00000020032	TRMT10A
ENSGALT00000022020	TBC1D1	ENSGALT00000007543	
ENSGALT00000039224		ENSGALT00000034233	KCNB1
ENSGALT00000003519		ENSGALT00000000070	
ENSGALT00000010979		ENSGALT00000003192	
ENSGALT00000003581	MFSD6	ENSGALT00000028340	
ENSGALT00000003630		ENSGALT00000020064	EMCN
ENSGALT00000003720		ENSGALT00000003246	MMP19
ENSGALT00000003753	OSGEPL1	ENSGALT00000007669	EPB42
ENSGALT00000021744	PCBP2	ENSGALT00000007724	CCNDBP1
ENSGALT00000000200	SUPT6H	ENSGALT00000020115	MANBA
ENSGALT00000003781		ENSGALT00000020119	
ENSGALT00000011061	SNX21	ENSGALT00000008180	
ENSGALT00000023186	STIM2	ENSGALT00000020161	BDH2
ENSGALT00000011074	ACOT8	ENSGALT00000003372	PEX12
ENSGALT00000003954	COL5A2	ENSGALT00000008232	LSM14B
ENSGALT00000011138		ENSGALT00000021898	SORBS2

ENSGALT00000023214	SEL1L3	ENSGALT00000008294	MTG2
ENSGALT00000039136	PCIF1	ENSGALT00000008344	
ENSGALT00000004032		ENSGALT00000008535	LAMA5
ENSGALT00000034022	TFPI	ENSGALT00000003612	TMEM13<br>2E
ENSGALT00000004147		ENSGALT00000022040	
ENSGALT00000006318	RPL23A	ENSGALT00000041383	
ENSGALT00000011227	SLC12A5	ENSGALT00000023517	
ENSGALT00000011344	NCOA5	ENSGALT00000022114	
ENSGALT00000004253	DUSP19	ENSGALT00000003748	
ENSGALT00000004344	NCKAP1	ENSGALT00000009067	
ENSGALT00000011732		ENSGALT00000022137	
ENSGALT00000012067	CTSZ	ENSGALT00000003757	
ENSGALT00000023256	SOD3	ENSGALT00000009144	
ENSGALT00000006365		ENSGALT00000003859	
ENSGALT00000023259		ENSGALT00000037573	
ENSGALT00000023263		ENSGALT00000032107	PDGFRL
ENSGALT00000039685		ENSGALT00000022171	
ENSGALT00000006386	DHRS13	ENSGALT00000009286	
ENSGALT00000012184	NPEPL1	ENSGALT00000009312	
ENSGALT00000012194		ENSGALT00000022194	VPS37A
ENSGALT00000006417	PHF12	ENSGALT00000022198	
ENSGALT00000004363		ENSGALT00000009385	
ENSGALT00000041120	FSTL3	ENSGALT00000033928	
ENSGALT00000004522	KANSL1L	ENSGALT00000009494	PCMTD2
ENSGALT00000012215		ENSGALT00000022308	
ENSGALT00000004557		ENSGALT00000022312	
ENSGALT00000004587		ENSGALT00000009656	PRPF6
ENSGALT00000040054		ENSGALT00000003944	
ENSGALT00000012397		ENSGALT00000004011	
ENSGALT00000004610		ENSGALT00000004075	CRCP
ENSGALT00000012648	ZNF217	ENSGALT00000004087	TPST1
ENSGALT00000023419		ENSGALT00000004122	
ENSGALT00000012669	TSHZ2	ENSGALT00000009779	ZBTB46
ENSGALT00000023420		ENSGALT00000004144	TMEM24<br>8
ENSGALT00000023421	FBXL5	ENSGALT00000036322	
ENSGALT00000028050	DPM1	ENSGALT00000036310	
ENSGALT00000024191	LYAR	ENSGALT00000009828	
ENSGALT00000038844		ENSGALT00000032094	SRD5A3
ENSGALT00000013022		ENSGALT00000004146	FAM211A
ENSGALT00000013071	B4GALT5	ENSGALT00000004183	

ENSGALT00000006434	NUFIP2	ENSGALT00000022578	CHIC2
ENSGALT00000024208		ENSGALT00000004394	PITPNA
ENSGALT00000005653		ENSGALT00000022602	SCFD2
ENSGALT00000005664		ENSGALT00000032088	USP46
ENSGALT00000004660	TXNL1	ENSGALT00000022640	
ENSGALT00000004573	ALPK2	ENSGALT00000022792	DCUN1D4
ENSGALT00000004529	MALT1	ENSGALT00000009996	SLC52A3
ENSGALT00000034909	SSH2	ENSGALT00000009999	
ENSGALT00000025037	WFS1	ENSGALT00000022799	OCIAD1
ENSGALT00000006652	NSRP1	ENSGALT00000010006	TBC1D20
ENSGALT00000025045	MAN2B2	ENSGALT00000004650	PRPF8
ENSGALT00000006250		ENSGALT00000010020	HM13
ENSGALT00000006838	CPD	ENSGALT00000022828	FRYL
ENSGALT00000004384		ENSGALT00000022847	SLAIN2
ENSGALT00000004279	LIPG	ENSGALT00000010025	
ENSGALT00000025066		ENSGALT00000010073	
ENSGALT00000006274		ENSGALT00000010129	
ENSGALT00000006341	IQCA1	ENSGALT00000004728	SERPINF2
ENSGALT00000006438		ENSGALT00000004762	
ENSGALT00000025088	ABLIM2	ENSGALT00000010607	TM9SF4
ENSGALT00000025114	HTRA3	ENSGALT00000022980	
ENSGALT00000025139	ACOX3	ENSGALT00000022997	
ENSGALT00000006898		ENSGALT00000004849	
ENSGALT00000025174	DOK7	ENSGALT00000023042	SLC30A9
ENSGALT00000029361	C18orf32	ENSGALT00000023043	TMEM33
ENSGALT00000029461		ENSGALT00000004908	SMG6
ENSGALT00000039658		ENSGALT00000010635	PLAGL2
ENSGALT00000006583	SH3BP4	ENSGALT00000010675	ASXL1
ENSGALT00000006741	TRAF3IP1	ENSGALT00000005087	POLDIP2
ENSGALT00000025226		ENSGALT00000039767	TMEM199
ENSGALT00000002706		ENSGALT00000023095	N4BP2
ENSGALT00000007018	SPECC1	ENSGALT00000023145	SMIM14
ENSGALT00000002271	NISCH	ENSGALT00000023144	
ENSGALT00000002333	STAB1	ENSGALT00000023136	LIAS
ENSGALT00000025259		ENSGALT00000010897	CDK5RAP1
ENSGALT00000002566	SETBP1	ENSGALT00000010917	
ENSGALT00000033730		ENSGALT00000005809	PIGS
ENSGALT00000003868	AQP3	ENSGALT00000029529	KLHL5

ENSGALT00000002555		ENSGALT00000022105	
ENSGALT00000007130	NCOR1	ENSGALT00000000233	KLF3
ENSGALT00000025264	FAM193A	ENSGALT00000022020	TBC1D1
ENSGALT00000002382	NT5DC2	ENSGALT00000039224	
ENSGALT00000009315		ENSGALT00000003519	
ENSGALT00000025319		ENSGALT00000010979	
ENSGALT00000039669		ENSGALT00000003560	
ENSGALT00000025330		ENSGALT00000003581	MFSD6
ENSGALT00000034499	UBAP1	ENSGALT00000003630	
ENSGALT00000009887		ENSGALT00000003720	
ENSGALT00000007174	CENPV	ENSGALT00000021744	PCBP2
ENSGALT00000002508		ENSGALT00000000200	SUPT6H
ENSGALT00000009335		ENSGALT00000003781	
ENSGALT00000009350	FAM219A	ENSGALT00000003836	
ENSGALT00000025335		ENSGALT00000011061	SNX21
ENSGALT00000034480		ENSGALT00000003954	COL5A2
ENSGALT00000006957	MCM3AP	ENSGALT00000011138	
ENSGALT00000007195	TRPV2	ENSGALT00000037357	
ENSGALT00000007345		ENSGALT00000023214	SEL1L3
ENSGALT00000025361		ENSGALT00000039136	PCIF1
ENSGALT00000007351	TAX1BP3	ENSGALT00000004032	
ENSGALT00000002971	DNAJB5	ENSGALT00000004147	
ENSGALT00000003093		ENSGALT00000023229	ANAPC4
ENSGALT00000036295		ENSGALT00000023231	ZCCHC4
ENSGALT00000002638		ENSGALT00000006318	RPL23A
ENSGALT00000003276	PFKFB4	ENSGALT00000011227	SLC12A5
ENSGALT00000007457	AKAP10	ENSGALT00000011344	NCOA5
ENSGALT00000025439	PTCD3	ENSGALT00000004236	NUP35
ENSGALT00000003341	HYAL2	ENSGALT00000004253	DUSP19
ENSGALT00000012144		ENSGALT00000004344	NCKAP1
ENSGALT00000025455	REEP1	ENSGALT00000011732	
ENSGALT00000003330	NPRL2	ENSGALT00000023246	
ENSGALT00000003543		ENSGALT00000012067	CTSZ
ENSGALT00000003585	MAPKAPK3	ENSGALT00000012140	
ENSGALT00000025484		ENSGALT00000023256	SOD3
ENSGALT00000007525	ULK2	ENSGALT00000006365	
ENSGALT00000003737		ENSGALT00000023259	
ENSGALT00000012579	TMEFF2	ENSGALT00000023263	
ENSGALT00000025489		ENSGALT00000023270	GPR125
ENSGALT00000012866		ENSGALT00000012184	NPEPL1
ENSGALT00000007598		ENSGALT00000012194	
ENSGALT00000007650	NXN	ENSGALT00000006417	PHF12

ENSGALT00000023124		ENSGALT00000004363	
ENSGALT00000013057	COQ10B	ENSGALT00000041120	FSTL3
ENSGALT00000023126	PAK4	ENSGALT00000004522	KANSL1L
ENSGALT00000015642		ENSGALT00000012215	
ENSGALT00000023128		ENSGALT00000004557	
ENSGALT00000035848		ENSGALT00000004587	
ENSGALT00000003768		ENSGALT00000012397	
ENSGALT00000007703	FAM57A	ENSGALT00000004610	
ENSGALT00000025715	EIF2AK3	ENSGALT00000033524	AURKA
ENSGALT00000007801		ENSGALT00000012648	ZNF217
ENSGALT00000032015		ENSGALT00000012669	TSHZ2
ENSGALT00000003917	RNF123	ENSGALT00000023420	
ENSGALT00000033513	C2orf47	ENSGALT00000028050	DPM1
ENSGALT00000007833	FAM101B	ENSGALT00000012995	FAM65C
ENSGALT00000025750	PANK2	ENSGALT00000024191	LYAR
ENSGALT00000025773		ENSGALT00000038844	
ENSGALT00000020313		ENSGALT00000013022	
ENSGALT00000013252	KCTD18	ENSGALT00000013071	B4GALT5
ENSGALT00000033496		ENSGALT00000006434	NUFIP2
ENSGALT00000003976	CREB3	ENSGALT00000024208	
ENSGALT00000013327		ENSGALT00000009906	HELZ2
ENSGALT00000008115	PPM1E	ENSGALT00000005653	
ENSGALT00000004064		ENSGALT00000005664	
ENSGALT00000008166		ENSGALT00000004660	TXNL1
ENSGALT00000008181	GDPD1	ENSGALT00000006045	
ENSGALT00000034416	CA9	ENSGALT00000004529	MALT1
ENSGALT00000025784	UBOX5	ENSGALT00000025037	WFS1
ENSGALT00000013409		ENSGALT00000006652	NSRP1
ENSGALT00000025775	FASTKD5	ENSGALT00000025045	MAN2B2
ENSGALT00000025781		ENSGALT00000006773	
ENSGALT00000025785	DDRGK1	ENSGALT00000006250	
ENSGALT00000008234	DHX40	ENSGALT00000006838	CPD
ENSGALT00000013633	TRAK2	ENSGALT00000004384	
ENSGALT00000013642	STRADB	ENSGALT00000025066	
ENSGALT00000005129	RAD54L2	ENSGALT00000006274	
ENSGALT00000013654	TMEM237	ENSGALT00000006341	IQCA1
ENSGALT00000005219	ADAMTS12	ENSGALT00000006438	
ENSGALT00000008250		ENSGALT00000025088	ABLIM2
ENSGALT00000013679	MPP4	ENSGALT00000025114	HTRA3
ENSGALT00000005305	RAI14	ENSGALT00000025139	ACOX3
ENSGALT00000008286	VMP1	ENSGALT00000006898	
ENSGALT00000025818	C20orf194	ENSGALT00000025174	DOK7

ENSGALT00000025829	ATRN	ENSGALT00000039658	
ENSGALT00000005931		ENSGALT00000006583	SH3BP4
ENSGALT00000034226		ENSGALT00000006741	TRAF3IP1
ENSGALT00000006075	TEX264	ENSGALT00000006793	ASB1
ENSGALT00000013707	ALS2	ENSGALT00000034546	ST8SIA5
ENSGALT00000013744		ENSGALT00000025226	
ENSGALT00000013776	NOP58	ENSGALT00000002706	
ENSGALT00000008348		ENSGALT00000002271	NISCH
ENSGALT00000006336		ENSGALT00000002333	STAB1
ENSGALT00000006367		ENSGALT00000025259	
ENSGALT00000008358	RNFT1	ENSGALT00000033730	
ENSGALT00000036280		ENSGALT00000003868	AQP3
ENSGALT00000008405	MED13	ENSGALT00000002555	
ENSGALT00000025839		ENSGALT00000007130	NCOR1
ENSGALT00000008456		ENSGALT00000006991	
ENSGALT00000005708		ENSGALT00000025262	
ENSGALT00000025939	EXOC6B	ENSGALT00000025264	FAM193A
ENSGALT00000008492	BCAS3	ENSGALT00000025265	
ENSGALT00000007193	PARP3	ENSGALT00000002382	NT5DC2
ENSGALT00000013828	CCNYL1	ENSGALT00000009315	
ENSGALT00000008505	PPM1D	ENSGALT00000041399	C4orf48
ENSGALT00000008517		ENSGALT00000025319	
ENSGALT00000008580		ENSGALT00000039669	
ENSGALT00000005943	OSMR	ENSGALT00000025330	
ENSGALT00000029609		ENSGALT00000034499	UBAP1
ENSGALT00000023934	TTC33	ENSGALT00000009887	
ENSGALT00000004187		ENSGALT00000007174	CENPV
ENSGALT00000023947	C7	ENSGALT00000002508	
ENSGALT00000023962	PLCXD3	ENSGALT00000009335	
ENSGALT00000000026	AAK1	ENSGALT00000009350	FAM219A
ENSGALT00000023976		ENSGALT00000025335	
ENSGALT00000007426		ENSGALT00000025338	
ENSGALT00000023983	C5orf28	ENSGALT00000034480	
ENSGALT00000000046		ENSGALT00000006957	MCM3AP
ENSGALT00000041239	ECSCR	ENSGALT00000007195	TRPV2
ENSGALT00000007519	ASPN	ENSGALT00000025361	
ENSGALT00000007571	NOL8	ENSGALT00000007351	TAX1BP3
ENSGALT00000000136	ANTXR1	ENSGALT00000007373	CTNS
ENSGALT00000023988	PAIP1	ENSGALT00000002971	DNAJB5
ENSGALT00000023990	NNT	ENSGALT00000003093	
ENSGALT00000023997		ENSGALT00000036295	

ENSGALT00000032856		ENSGALT00000007388	SHPK
ENSGALT00000007684	IARS	ENSGALT00000002638	
ENSGALT00000024048	ARL15	ENSGALT00000003010	
ENSGALT00000014015		ENSGALT00000003276	PFKFB4
ENSGALT00000003856	CTNNA1	ENSGALT00000007457	AKAP10
ENSGALT00000014048	NRP2	ENSGALT00000025439	PTCD3
ENSGALT00000023700	DHX29	ENSGALT00000025450	
ENSGALT00000023719		ENSGALT00000012144	
ENSGALT00000000221	CDS2	ENSGALT00000003330	NPRL2
ENSGALT00000023723	SLC38A9	ENSGALT00000003543	
ENSGALT00000003725		ENSGALT00000003585	MAPKAP K3
ENSGALT00000003545	ETF1	ENSGALT00000025484	
ENSGALT00000014120	RAPH1	ENSGALT00000007525	ULK2
ENSGALT00000023728		ENSGALT00000003737	
ENSGALT00000014182	ABI2	ENSGALT00000025489	
ENSGALT00000000227		ENSGALT00000012866	
ENSGALT00000003489		ENSGALT00000007598	
ENSGALT00000007996		ENSGALT00000007650	NXN
ENSGALT00000014220		ENSGALT00000023124	
ENSGALT00000014289	NBEAL1	ENSGALT00000013057	COQ10B
ENSGALT00000032871	DIMT1	ENSGALT00000025688	THNSL2
ENSGALT00000014333	IDH1	ENSGALT00000003723	MANF
ENSGALT00000014370	PIKFYVE	ENSGALT00000023128	
ENSGALT00000002866		ENSGALT00000007703	FAM57A
ENSGALT00000008859	DYNLL2	ENSGALT00000025715	EIF2AK3
ENSGALT00000039052		ENSGALT00000013202	
ENSGALT00000023780	CWC27	ENSGALT00000007801	
ENSGALT00000002683	MAT2B	ENSGALT00000032015	
ENSGALT00000002649	NUDCD2	ENSGALT00000003917	RNF123
ENSGALT00000002636	CCNG1	ENSGALT00000033513	C2orf47
ENSGALT00000023799	PPWD1	ENSGALT00000007833	FAM101B
ENSGALT00000023814	TRIM23	ENSGALT00000025750	PANK2
ENSGALT00000008944	AKAP1	ENSGALT00000025773	
ENSGALT00000008081	IQSEC1	ENSGALT00000003959	IP6K1
ENSGALT00000008142	NUP210	ENSGALT00000020313	
ENSGALT00000008222	FBLN2	ENSGALT00000033496	
ENSGALT00000023839	SREK1	ENSGALT00000003976	CREB3
ENSGALT00000023868	PIK3R1	ENSGALT00000013327	
ENSGALT00000008313	FAM120A	ENSGALT00000008115	PPM1E
ENSGALT00000023876	CDK7	ENSGALT00000004292	
ENSGALT00000001892	PPARGC1B	ENSGALT00000034799	PRR11

ENSGALT00000002082		ENSGALT00000013401	
ENSGALT00000000362		ENSGALT00000004064	
ENSGALT00000008368	WNK2	ENSGALT00000008166	
ENSGALT00000004213	GRPEL2	ENSGALT00000008181	GDPD1
ENSGALT00000001972	CLIP2	ENSGALT00000034416	CA9
ENSGALT00000008413	FGD3	ENSGALT00000025784	UBOX5
ENSGALT00000023888	SERINC5	ENSGALT00000013409	
ENSGALT00000004451	STK10	ENSGALT00000025775	FASTKD5
ENSGALT00000033324		ENSGALT00000025781	
ENSGALT00000014643		ENSGALT00000025785	DDRGK1
ENSGALT00000023911	PAPD4	ENSGALT00000008234	DHX40
ENSGALT00000007194		ENSGALT00000013633	TRAK2
ENSGALT00000007151	DMGDH	ENSGALT00000013642	STRADB
ENSGALT00000004469	SH3PXD2B	ENSGALT00000005129	RAD54L2
ENSGALT00000007061	LHFPL2	ENSGALT00000013654	TMEM237
ENSGALT00000007047		ENSGALT00000005018	MTMR12
ENSGALT00000004489	NEURL1B	ENSGALT00000025803	SLC4A11
ENSGALT00000004517	ERGIC1	ENSGALT00000028093	NPR3
ENSGALT00000008433	BICD2	ENSGALT00000005219	ADAMTS12
ENSGALT00000006998	AP3B1	ENSGALT00000008250	
ENSGALT00000014791		ENSGALT00000013679	MPP4
ENSGALT00000008538	DCP1A	ENSGALT00000005305	RAI14
ENSGALT00000009090		ENSGALT00000005353	DNAJC21
ENSGALT00000024118	COL4A3BP	ENSGALT00000008286	VMP1
ENSGALT00000040996	STC2	ENSGALT00000025818	C20orf194
ENSGALT00000022788	CPEB4	ENSGALT00000025829	ATRN
ENSGALT00000024097	GFM2	ENSGALT00000005931	
ENSGALT00000024086	HEXB	ENSGALT00000034226	
ENSGALT00000008687		ENSGALT00000006075	TEX264
ENSGALT00000008746	CCDC66	ENSGALT00000013707	ALS2
ENSGALT00000008882	ASB14	ENSGALT00000013744	
ENSGALT00000029725		ENSGALT00000008348	
ENSGALT00000029777		ENSGALT00000006367	
ENSGALT00000009143	NLK	ENSGALT00000008358	RNFT1
ENSGALT00000000518	SLC25A37	ENSGALT00000036280	
ENSGALT00000000531	ENTPD4	ENSGALT00000008405	MED13
ENSGALT00000009146	TMEM97	ENSGALT00000001111	
ENSGALT00000040958		ENSGALT00000025839	
ENSGALT00000024194	MRPS27	ENSGALT00000025924	PRADC1

ENSGALT00000024183	PGM5	ENSGALT00000008456	
ENSGALT00000000548	LOXL2	ENSGALT00000025927	RAB11FIP5
ENSGALT00000016528	SMARCA2	ENSGALT00000005708	
ENSGALT00000016531		ENSGALT00000025939	EXOC6B
ENSGALT00000023329	AK3	ENSGALT00000008492	BCAS3
ENSGALT00000009018	PDE12	ENSGALT00000007193	PARP3
ENSGALT00000009021	ARF4	ENSGALT00000013828	CCNYL1
ENSGALT00000040952		ENSGALT00000005878	
ENSGALT00000024242	CD274	ENSGALT00000008517	
ENSGALT00000024250	KIAA1432	ENSGALT00000005924	
ENSGALT00000009362	MNT	ENSGALT00000008580	
ENSGALT00000009368		ENSGALT00000005943	OSMR
ENSGALT00000009407	CLUH	ENSGALT00000001647	
ENSGALT00000009165	NCKIPSD	ENSGALT00000029609	
ENSGALT00000000956	MYO1D	ENSGALT00000023934	TTC33
ENSGALT00000040727	PSMD11	ENSGALT00000004187	
ENSGALT00000024268	KIAA2026	ENSGALT00000023947	C7
ENSGALT00000002766		ENSGALT00000023962	PLCXD3
ENSGALT00000015032	AGPS	ENSGALT00000000026	AAK1
ENSGALT00000033290		ENSGALT00000023976	
ENSGALT00000004794		ENSGALT00000007426	
ENSGALT00000024372		ENSGALT00000007467	
ENSGALT00000040680	SCN4A	ENSGALT00000023983	C5orf28
ENSGALT00000015075	MTX2	ENSGALT00000000046	
ENSGALT00000009300	UQCRC1	ENSGALT00000004015	PCDH12
ENSGALT00000009663	MED31	ENSGALT00000041239	ECSCR
ENSGALT00000024341		ENSGALT00000007480	ECM2
ENSGALT00000009741	FAM64A	ENSGALT00000007519	ASPN
ENSGALT00000015103	KIAA1715	ENSGALT00000007571	NOL8
ENSGALT00000015106		ENSGALT00000000136	ANTXR1
ENSGALT00000002520	TTI2	ENSGALT00000023988	PAIP1
ENSGALT00000038228	FXN	ENSGALT00000023990	NNT
ENSGALT00000009792	SMTNL2	ENSGALT00000023997	
ENSGALT00000024378	FAM189A2	ENSGALT00000032856	
ENSGALT00000002495	DUSP26	ENSGALT00000007684	IARS
ENSGALT00000024387	MAMDC2	ENSGALT00000003921	
ENSGALT00000022979	GPR155	ENSGALT00000024048	ARL15
ENSGALT00000001720	ZNF346	ENSGALT00000014015	
ENSGALT00000000469	PSMC5	ENSGALT00000003856	CTNNA1
ENSGALT00000024435		ENSGALT00000023700	DHX29
ENSGALT00000015187		ENSGALT00000023719	

ENSGALT00000015190		ENSGALT00000000221	CDS2
ENSGALT00000015215		ENSGALT00000023723	SLC38A9
ENSGALT00000021611		ENSGALT00000003725	
ENSGALT00000021613		ENSGALT00000003545	ETF1
ENSGALT00000000694		ENSGALT00000014120	RAPH1
ENSGALT00000000731		ENSGALT00000023728	
ENSGALT00000032821		ENSGALT00000007902	
ENSGALT00000000739	ERRFI1	ENSGALT00000014182	ABI2
ENSGALT00000015508		ENSGALT00000000227	
ENSGALT00000032816	NMRK1	ENSGALT00000023732	MAP3K1
ENSGALT00000015535	SLC25A12	ENSGALT00000003489	
ENSGALT00000000541		ENSGALT00000007996	
ENSGALT00000000797	PER3	ENSGALT00000003449	
ENSGALT00000000559	TLK2	ENSGALT00000014220	
ENSGALT00000009434		ENSGALT00000014289	NBEAL1
ENSGALT00000004898	PROSC	ENSGALT00000032871	DIMT1
ENSGALT00000009495	RAB7A	ENSGALT00000014333	IDH1
ENSGALT00000009501	RPN1	ENSGALT00000008038	
ENSGALT00000000891	DNAJC11	ENSGALT00000014370	PIKFYVE
ENSGALT00000000627	MRC2	ENSGALT00000002866	
ENSGALT00000015547		ENSGALT00000008859	DYNLL2
ENSGALT00000015562	DCAF17	ENSGALT00000039052	
ENSGALT00000020544	FRMD3	ENSGALT00000039046	
ENSGALT00000020546	IDNK	ENSGALT00000023780	CWC27
ENSGALT00000030746		ENSGALT00000002683	MAT2B
ENSGALT00000009510	EEFSEC	ENSGALT00000002649	NUDCD2
ENSGALT00000009542		ENSGALT00000002636	CCNG1
ENSGALT00000020549		ENSGALT00000023799	PPWD1
ENSGALT00000004947	RAB11FIP1	ENSGALT00000008930	MSI2
ENSGALT00000020552	GKAP1	ENSGALT00000000305	
ENSGALT00000009591	SEC61A1	ENSGALT00000008944	AKAP1
ENSGALT00000000901	KLHL21	ENSGALT00000002291	
ENSGALT00000009602	KBTBD12	ENSGALT00000008081	IQSEC1
ENSGALT00000005328		ENSGALT00000008222	FBLN2
ENSGALT00000009651		ENSGALT00000023839	SREK1
ENSGALT00000005077	EIF4EBP1	ENSGALT00000023868	PIK3R1
ENSGALT00000005337	SIMC1	ENSGALT00000002157	ADRA1B
ENSGALT00000000697		ENSGALT00000008313	FAM120A
ENSGALT00000040847		ENSGALT00000023876	CDK7
ENSGALT00000009723		ENSGALT00000001892	PPARGC 1B
ENSGALT00000020559	C9orf64	ENSGALT00000002082	

ENSGALT00000015660	UBR3	ENSGALT00000000362	
ENSGALT00000009740		ENSGALT00000008368	WNK2
ENSGALT00000020563		ENSGALT00000004213	GRPEL2
ENSGALT00000009765		ENSGALT00000001972	CLIP2
ENSGALT00000005847	RNF145	ENSGALT00000008413	FGD3
ENSGALT00000029074		ENSGALT00000023888	SERINC5
ENSGALT00000009993		ENSGALT00000004443	UBTD2
ENSGALT00000010098	KLF15	ENSGALT00000004451	STK10
ENSGALT00000010142		ENSGALT00000033324	
ENSGALT00000015749	METTL5	ENSGALT00000014643	
ENSGALT00000020564		ENSGALT00000007151	DMGDH
ENSGALT00000020572		ENSGALT00000004469	SH3PXD2B
ENSGALT00000001006	RNF207	ENSGALT00000007061	LHFPL2
ENSGALT00000023340	CHD5	ENSGALT00000007047	
ENSGALT00000020577	GOLM1	ENSGALT00000004489	NEURL1B
ENSGALT00000020582		ENSGALT00000004517	ERGIC1
ENSGALT00000015791	KLHL23	ENSGALT00000008433	BICD2
ENSGALT00000001441	C1orf174	ENSGALT00000014694	CCDC141
ENSGALT00000010231		ENSGALT00000006998	AP3B1
ENSGALT00000015973	CCDC173	ENSGALT00000006867	AGGF1
ENSGALT00000001448	LRRC47	ENSGALT00000008538	DCP1A
ENSGALT00000000783	CCDC47	ENSGALT00000009090	
ENSGALT00000010255		ENSGALT00000038562	F2RL2
ENSGALT00000015996	PPIG	ENSGALT00000004538	
ENSGALT00000020588		ENSGALT00000024118	COL4A3BP
ENSGALT00000020591		ENSGALT00000008651	ACTR8
ENSGALT00000040509		ENSGALT00000040996	STC2
ENSGALT00000001513	TPRG1L	ENSGALT00000022788	CPEB4
ENSGALT00000016007		ENSGALT00000024097	GFM2
ENSGALT00000006182	SGCD	ENSGALT00000024086	HEXB
ENSGALT00000006244	MRPL22	ENSGALT00000008687	
ENSGALT00000006272	GEMIN5	ENSGALT00000008746	CCDC66
ENSGALT00000006374	LARP1	ENSGALT00000008805	
ENSGALT00000010371		ENSGALT00000005114	
ENSGALT00000010411		ENSGALT00000008882	ASB14
ENSGALT00000010440	CCDC51	ENSGALT00000029725	
ENSGALT00000006432		ENSGALT00000029777	
ENSGALT00000020613		ENSGALT00000009143	NLK

ENSGALT00000000842	KANSL1	ENSGALT00000000518	SLC25A3<br>7
ENSGALT00000034078		ENSGALT00000000531	ENTPD4
ENSGALT00000040399	SLC25A39	ENSGALT00000009146	TMEM97
ENSGALT00000017380	S1PR3	ENSGALT00000040958	
ENSGALT00000016166		ENSGALT00000024194	MRPS27
ENSGALT00000016165	FAM117A	ENSGALT00000009153	IFT20
ENSGALT00000006615		ENSGALT00000001663	GTSF1
ENSGALT00000035387		ENSGALT00000024183	PGM5
ENSGALT00000005308	TM2D2	ENSGALT00000000548	LOXL2
ENSGALT00000001855		ENSGALT00000016528	SMARCA<br>2
ENSGALT00000021979	SPTLC1	ENSGALT00000016531	
ENSGALT00000000584		ENSGALT00000016548	RFX3
ENSGALT00000000207	ADAMTS19	ENSGALT00000023329	AK3
ENSGALT00000005373		ENSGALT00000009018	PDE12
ENSGALT00000010811	TADA3	ENSGALT00000009021	ARF4
ENSGALT00000001940	C17orf105	ENSGALT00000024230	RCL1
ENSGALT00000037896		ENSGALT00000024242	CD274
ENSGALT00000010572	SRPK3	ENSGALT00000024250	KIAA1432
ENSGALT00000001986	TMEM52	ENSGALT00000009368	
ENSGALT00000006949		ENSGALT00000009407	CLUH
ENSGALT00000006950		ENSGALT00000001199	
ENSGALT00000010618		ENSGALT00000014983	
ENSGALT00000001801	DHX8	ENSGALT00000000956	MYO1D
ENSGALT00000010655	MTMR14	ENSGALT00000040727	PSMD11
ENSGALT00000001955	ZNF652	ENSGALT00000024268	KIAA2026
ENSGALT00000001957		ENSGALT00000002766	
ENSGALT00000001966	ABI3	ENSGALT00000015032	AGPS
ENSGALT00000035169		ENSGALT00000033290	
ENSGALT00000002094	SLC35E2B	ENSGALT00000004794	
ENSGALT00000007164	TNIP1	ENSGALT00000024372	
ENSGALT00000001975		ENSGALT00000040680	SCN4A
ENSGALT00000000332		ENSGALT00000015075	MTX2
ENSGALT00000000334	MAN2A1	ENSGALT00000009300	UQCRC1
ENSGALT00000000365	FBXL17	ENSGALT00000009663	MED31
ENSGALT00000024619	PAM	ENSGALT00000024341	
ENSGALT00000002003	UBE2Z	ENSGALT00000009741	FAM64A
ENSGALT00000007219	DCTN4	ENSGALT00000015103	KIAA1715
ENSGALT00000010798	CAMK1	ENSGALT00000000004	
ENSGALT00000002119	MMP23B	ENSGALT00000015106	
ENSGALT00000005511		ENSGALT00000038862	FBXO39

ENSGALT00000007249		ENSGALT00000038228	FXN
ENSGALT00000007258	MYOZ3	ENSGALT00000040669	RCC2
ENSGALT00000007290	NDST1	ENSGALT00000009792	SMTNL2
ENSGALT00000007299		ENSGALT00000024378	FAM189A2
ENSGALT00000007311		ENSGALT00000002495	DUSP26
ENSGALT00000040533		ENSGALT00000024387	MAMDC2
ENSGALT00000005561	AGPAT6	ENSGALT00000022979	GPR155
ENSGALT00000000312	SP2	ENSGALT00000001720	ZNF346
ENSGALT00000002248		ENSGALT00000000469	PSMC5
ENSGALT00000024669	RGMB	ENSGALT00000023090	
ENSGALT00000009192	PDGFRB	ENSGALT00000024435	
ENSGALT00000009190	CSF1R	ENSGALT00000015187	
ENSGALT00000009267	HMGXB3	ENSGALT00000015190	
ENSGALT00000010963	KLHDC8B	ENSGALT00000015215	
ENSGALT00000024694	LNPEP	ENSGALT00000021611	
ENSGALT00000024714	RNF38	ENSGALT00000021613	
ENSGALT00000005683		ENSGALT00000000694	
ENSGALT00000024737		ENSGALT00000000731	
ENSGALT00000036239		ENSGALT00000015487	METAP1D
ENSGALT00000024770	PIGG	ENSGALT00000032821	
ENSGALT00000024799		ENSGALT00000000739	ERRFI1
ENSGALT00000002392	MLLT6	ENSGALT00000015508	
ENSGALT00000011032	USP19	ENSGALT00000015535	SLC25A12
ENSGALT00000002321	VWA1	ENSGALT00000000541	
ENSGALT00000002423	PSMB3	ENSGALT00000000797	PER3
ENSGALT00000018083		ENSGALT00000009434	
ENSGALT00000002452	PIP4K2B	ENSGALT00000004898	PROSC
ENSGALT00000011121	QRICH1	ENSGALT00000009495	RAB7A
ENSGALT00000009349	RNF130	ENSGALT00000009501	RPN1
ENSGALT00000002480	CWC25	ENSGALT00000000891	DNAJC11
ENSGALT00000002426		ENSGALT00000000627	MRC2
ENSGALT00000005799	AP3M2	ENSGALT00000015547	
ENSGALT00000017943		ENSGALT00000024492	
ENSGALT00000036016		ENSGALT00000020544	FRMD3
ENSGALT00000009410	TBC1D9B	ENSGALT00000020546	IDNK
ENSGALT00000011269		ENSGALT00000030746	
ENSGALT00000005886	PLAT	ENSGALT00000009510	EEFSEC
ENSGALT00000002516		ENSGALT00000009542	
ENSGALT00000011358	PRKAR2A	ENSGALT00000020549	

ENSGALT00000024832		ENSGALT00000004947	RAB11FIP1
ENSGALT00000024838		ENSGALT00000020552	GKAP1
ENSGALT00000040133	PLXDC1	ENSGALT00000005307	NOP16
ENSGALT00000037774		ENSGALT00000009591	SEC61A1
ENSGALT00000024886	PSD3	ENSGALT00000000901	KLHL21
ENSGALT00000033434	RPP14	ENSGALT00000009602	KBTBD12
ENSGALT00000029612	VDAC3	ENSGALT00000005328	
ENSGALT00000009500	SQSTM1	ENSGALT00000009651	
ENSGALT00000011505		ENSGALT00000005077	EIF4EBP1
ENSGALT00000024902	SLC44A1	ENSGALT00000005337	SIMC1
ENSGALT00000002573	FBXL20	ENSGALT00000000697	
ENSGALT00000002657	CDK12	ENSGALT00000040847	
ENSGALT00000023556	STARD3	ENSGALT00000009723	
ENSGALT00000024907	FKTN	ENSGALT00000020559	C9orf64
ENSGALT00000002864	UBE2J2	ENSGALT00000015660	UBR3
ENSGALT00000011518	KCTD6	ENSGALT00000020563	
ENSGALT00000011555	ACOX2	ENSGALT00000009765	
ENSGALT00000025051	RAD23B	ENSGALT00000005847	RNF145
ENSGALT00000009539	MAML1	ENSGALT00000009993	
ENSGALT00000002907		ENSGALT00000010098	KLF15
ENSGALT00000023458	KANSL3	ENSGALT00000010121	CCDC37
ENSGALT00000000352		ENSGALT00000010142	
ENSGALT00000009585		ENSGALT00000015749	METTL5
ENSGALT00000011619		ENSGALT00000005940	
ENSGALT00000029130		ENSGALT00000020569	AGTPBP1
ENSGALT00000023663	ERAP1	ENSGALT00000020572	
ENSGALT00000033125		ENSGALT00000023340	CHD5
ENSGALT00000011817		ENSGALT00000020577	GOLM1
ENSGALT00000003315	PLEKHN1	ENSGALT00000020582	
ENSGALT00000023641	TTC37	ENSGALT00000001441	C1orf174
ENSGALT00000009653	ADAMTS2	ENSGALT00000010231	
ENSGALT00000009661		ENSGALT00000015973	CCDC173
ENSGALT00000009699	CNOT6	ENSGALT00000001448	LRRC47
ENSGALT00000022352	GFPT2	ENSGALT00000000783	CCDC47
ENSGALT00000033111		ENSGALT00000010255	
ENSGALT00000009806		ENSGALT00000015996	PPIG
ENSGALT00000012074		ENSGALT00000020588	
ENSGALT00000023233		ENSGALT00000020591	

ENSGALT00000003902	GPR157	ENSGALT00000001513	TPRG1L
ENSGALT00000012239	KBTBD8	ENSGALT00000016000	FASTKD1
ENSGALT00000012394		ENSGALT00000016007	
ENSGALT00000028102		ENSGALT00000006159	MED7
ENSGALT00000003951	SPSB1	ENSGALT00000028046	
ENSGALT00000003969	SLC25A33	ENSGALT00000006182	SGCD
ENSGALT00000005592	KLHL11	ENSGALT00000006244	MRPL22
ENSGALT00000005478		ENSGALT00000006272	GEMIN5
ENSGALT00000005463		ENSGALT00000006297	
ENSGALT00000003980	TMEM201	ENSGALT00000006374	LARP1
ENSGALT00000025199	EDIL3	ENSGALT00000005246	
ENSGALT00000005381	ZNF385C	ENSGALT00000010371	
ENSGALT00000021871	TMF1	ENSGALT00000010411	
ENSGALT00000005327	KAT2A	ENSGALT00000010440	CCDC51
ENSGALT00000037654	SSBP2	ENSGALT00000006422	GALNT10
ENSGALT00000025157	ZCCHC9	ENSGALT00000006432	
ENSGALT00000004118		ENSGALT00000000842	KANSL1
ENSGALT00000032786		ENSGALT00000040399	SLC25A39
ENSGALT00000003806	GMPPA	ENSGALT00000017380	S1PR3
ENSGALT00000010215		ENSGALT00000016166	
ENSGALT00000004175	UBE4B	ENSGALT00000016165	FAM117A
ENSGALT00000022506		ENSGALT00000006615	
ENSGALT00000028126		ENSGALT00000006650	
ENSGALT00000023578	FRMD4B	ENSGALT00000035387	
ENSGALT00000040187		ENSGALT00000005308	TM2D2
ENSGALT00000012448		ENSGALT00000001855	
ENSGALT00000022505	PCYOX1	ENSGALT00000021979	SPTLC1
ENSGALT00000010230		ENSGALT00000000584	
ENSGALT00000010280		ENSGALT00000000207	ADAMTS19
ENSGALT00000025119		ENSGALT00000005373	
ENSGALT00000005164		ENSGALT00000010811	TADA3
ENSGALT00000018464		ENSGALT00000001940	C17orf105
ENSGALT00000025117	ZFYVE16	ENSGALT00000000276	MCC
ENSGALT00000013374	ANXA4	ENSGALT00000037896	
ENSGALT00000018446	DES	ENSGALT00000010572	SRPK3
ENSGALT00000034107	TXNDC15	ENSGALT00000001986	TMEM52
ENSGALT00000010339	DDX46	ENSGALT00000006949	
ENSGALT00000010356	SEC24A	ENSGALT00000006950	
ENSGALT00000012660	GXYLT2	ENSGALT00000010618	

ENSGALT00000004300	KIF1B	ENSGALT00000001801	DHX8
ENSGALT00000033329	PPP4R2	ENSGALT00000010655	MTMR14
ENSGALT00000032789		ENSGALT00000001955	ZNF652
ENSGALT00000037640		ENSGALT00000001957	
ENSGALT00000004476	PEX14	ENSGALT00000001966	ABI3
ENSGALT00000004554	CASZ1	ENSGALT00000035169	
ENSGALT00000018477		ENSGALT00000002094	SLC35E2B
ENSGALT00000029510		ENSGALT00000007164	TNIP1
ENSGALT00000025065		ENSGALT00000001975	
ENSGALT00000022896	NUDCD3	ENSGALT00000000323	CAMK4
ENSGALT00000025056		ENSGALT00000017724	
ENSGALT00000010437		ENSGALT00000000332	
ENSGALT00000004808	SRM	ENSGALT00000000334	MAN2A1
ENSGALT00000010461		ENSGALT00000000365	FBXL17
ENSGALT00000025241		ENSGALT00000024619	PAM
ENSGALT00000010508		ENSGALT00000002003	UBE2Z
ENSGALT00000010517		ENSGALT00000007219	DCTN4
ENSGALT00000005284	MTOR	ENSGALT00000002119	MMP23B
ENSGALT00000035033	ANGPTL7	ENSGALT00000002317	CALCOCO2
ENSGALT00000004844	EZH1	ENSGALT00000001947	HOXB9
ENSGALT00000005311		ENSGALT00000005511	
ENSGALT00000005447	PLEKHM2	ENSGALT00000007249	
ENSGALT00000025254	HSDL2	ENSGALT00000028252	
ENSGALT00000025295	UGCG	ENSGALT00000007258	MYOZ3
ENSGALT00000025297		ENSGALT00000007290	NDST1
ENSGALT00000025304	KIAA0368	ENSGALT00000007299	
ENSGALT00000028052	TRNT1	ENSGALT00000005561	AGPAT6
ENSGALT00000013466	CRBN	ENSGALT00000000312	SP2
ENSGALT00000005919	FBXO42	ENSGALT00000002304	
ENSGALT00000025350	SVEP1	ENSGALT00000024669	RGMB
ENSGALT00000005941		ENSGALT00000009192	PDGFRB
ENSGALT00000013524		ENSGALT00000009190	CSF1R
ENSGALT00000013532		ENSGALT00000009267	HMGXB3
ENSGALT00000010763		ENSGALT00000024694	LNPEP
ENSGALT00000025366		ENSGALT00000024714	RNF38
ENSGALT00000032768	TOPORS	ENSGALT00000005683	
ENSGALT00000013590	LMCD1	ENSGALT00000024737	
ENSGALT00000013623		ENSGALT00000024747	PCGF3
ENSGALT00000018495	FAM134A	ENSGALT00000036239	
ENSGALT00000002709	CAAP1	ENSGALT00000024770	PIGG

ENSGALT00000004510	RUNDC1	ENSGALT00000024799	
ENSGALT00000002747	PLAA	ENSGALT00000002392	MLLT6
ENSGALT00000002845	TEK	ENSGALT00000011032	USP19
ENSGALT00000039748		ENSGALT00000002321	VWA1
ENSGALT00000002894	C9orf72	ENSGALT00000002423	PSMB3
ENSGALT00000036208		ENSGALT00000018083	
ENSGALT00000004453	VAT1	ENSGALT00000002452	PIP4K2B
ENSGALT00000010935		ENSGALT00000011121	QRICH1
ENSGALT00000004366	NBR1	ENSGALT00000009349	RNF130
ENSGALT00000013700		ENSGALT00000002480	CWC25
ENSGALT00000013715		ENSGALT00000002426	
ENSGALT00000037566		ENSGALT00000005799	AP3M2
ENSGALT00000003135		ENSGALT00000036016	
ENSGALT00000003899	CYP27A1	ENSGALT00000009410	TBC1D9B
ENSGALT00000013770	IFT122	ENSGALT00000011269	
ENSGALT00000006036		ENSGALT00000005886	PLAT
ENSGALT00000013808	PLXND1	ENSGALT00000002516	
ENSGALT00000006223	UBR4	ENSGALT00000011358	PRKAR2A
ENSGALT00000013831	TMCC1	ENSGALT00000024832	
ENSGALT00000036642		ENSGALT00000040133	PLXDC1
ENSGALT00000003255		ENSGALT00000002630	
ENSGALT00000011543	AFF4	ENSGALT00000037774	
ENSGALT00000013922	FGD5	ENSGALT00000002716	ACAP3
ENSGALT00000003386		ENSGALT00000024886	PSD3
ENSGALT00000003423		ENSGALT00000033434	RPP14
ENSGALT00000011833		ENSGALT00000029612	VDAC3
ENSGALT00000006321		ENSGALT00000009500	SQSTM1
ENSGALT00000003649		ENSGALT00000011505	
ENSGALT00000011880		ENSGALT00000024902	SLC44A1
ENSGALT00000003696	ATG12	ENSGALT00000002573	FBXL20
ENSGALT00000011962		ENSGALT00000002623	MED1
ENSGALT00000011976		ENSGALT00000002657	CDK12
ENSGALT00000003718	CDO1	ENSGALT00000023556	STARD3
ENSGALT00000012177	TCERG1	ENSGALT00000024907	FKTN
ENSGALT00000003821		ENSGALT00000002864	UBE2J2
ENSGALT00000034531		ENSGALT00000011518	KCTD6
ENSGALT00000003846	BDP1	ENSGALT00000011555	ACOX2
ENSGALT00000003983	MCCC2	ENSGALT00000025051	RAD23B
ENSGALT00000018650	TMBIM1	ENSGALT00000009539	MAML1
ENSGALT00000012363		ENSGALT00000002907	
ENSGALT00000018675		ENSGALT00000023458	KANSL3

ENSGALT00000006425		ENSGALT00000023680	
ENSGALT00000012431		ENSGALT00000009585	
ENSGALT00000039619		ENSGALT00000023694	MEGF10
ENSGALT00000038163		ENSGALT00000011619	
ENSGALT00000004909	PINK1	ENSGALT00000029130	
ENSGALT00000022237		ENSGALT00000023663	ERAP1
ENSGALT00000013288		ENSGALT00000006746	STAMBP
ENSGALT00000013361	FEM1C	ENSGALT00000033125	
ENSGALT00000013392	ALDH7A1	ENSGALT00000011817	
ENSGALT00000018820		ENSGALT00000023641	TTC37
ENSGALT00000018822		ENSGALT00000003479	
ENSGALT00000018925		ENSGALT00000011836	PSMD6
ENSGALT00000008552	SNCAIP	ENSGALT00000009653	ADAMTS2
ENSGALT00000029784		ENSGALT00000009699	CNOT6
ENSGALT00000008572	PPIC	ENSGALT00000022352	GFPT2
ENSGALT00000008658	DCAF10	ENSGALT00000033111	
ENSGALT00000008663	POLR1E	ENSGALT00000009806	
ENSGALT00000019039	NIFK	ENSGALT00000018214	
ENSGALT00000008587	ZBTB5	ENSGALT00000012074	
ENSGALT00000019041		ENSGALT00000023233	
ENSGALT00000019048	IQCB1	ENSGALT00000003902	GPR157
ENSGALT00000019090	DIRC2	ENSGALT00000012239	KBTBD8
ENSGALT00000019105	SEMA5B	ENSGALT00000028102	
ENSGALT00000019110	PDIA5	ENSGALT00000003951	SPSB1
ENSGALT00000019136		ENSGALT00000003969	SLC25A33
ENSGALT00000019216		ENSGALT00000018346	
ENSGALT00000019224		ENSGALT00000005592	KLHL11
ENSGALT00000019286	HEG1	ENSGALT00000005478	
ENSGALT00000007199	MFN2	ENSGALT00000005463	
ENSGALT00000007216		ENSGALT00000003980	TMEM201
ENSGALT00000019689	SNX4	ENSGALT00000005381	ZNF385C
ENSGALT00000007303	CLCN6	ENSGALT00000021871	TMF1
ENSGALT00000019698	OSBPL11	ENSGALT00000005327	KAT2A
ENSGALT00000007342	MTHFR	ENSGALT00000037654	SSBP2
ENSGALT00000007392		ENSGALT00000025157	ZCCHC9
ENSGALT00000032941		ENSGALT00000004118	
ENSGALT00000007609	C1QB	ENSGALT00000032786	
ENSGALT00000007608	C1QA	ENSGALT00000005294	
ENSGALT00000019870	SLC35F5	ENSGALT00000003806	GMPPA

ENSGALT00000019898	MGAT5	ENSGALT00000005275	GHDC
ENSGALT00000034897		ENSGALT00000010215	
ENSGALT00000019996	R3HDM1	ENSGALT00000004175	UBE4B
ENSGALT00000007741		ENSGALT00000022506	
ENSGALT00000020021	UBXN4	ENSGALT00000028126	
ENSGALT00000007793	PPCS	ENSGALT00000023578	FRMD4B
ENSGALT00000008337	ANKRD13A	ENSGALT00000040187	
ENSGALT00000030001		ENSGALT00000012448	
ENSGALT00000020181		ENSGALT00000022505	PCYOX1
ENSGALT00000020209		ENSGALT00000010230	
ENSGALT00000032903		ENSGALT00000010280	
ENSGALT00000022876		ENSGALT00000025119	
ENSGALT00000016382		ENSGALT00000005164	
ENSGALT00000029632	DDI2	ENSGALT00000018464	
ENSGALT00000020351	MBD5	ENSGALT00000025117	ZFYVE16
ENSGALT00000020382		ENSGALT00000013374	ANXA4
ENSGALT00000029274		ENSGALT00000018446	DES
ENSGALT00000020391		ENSGALT00000034107	TXNDC15
ENSGALT00000020417		ENSGALT00000010339	DDX46
ENSGALT00000004115		ENSGALT00000010356	SEC24A
ENSGALT00000020434	ARL5A	ENSGALT00000004988	
ENSGALT00000037747	PRPF40A	ENSGALT00000012660	GXYLT2
ENSGALT00000020486	GPD2	ENSGALT00000004300	KIF1B
ENSGALT00000020497		ENSGALT00000033329	PPP4R2
ENSGALT00000020547	MARCH7	ENSGALT00000032789	
ENSGALT00000001983		ENSGALT00000004426	
ENSGALT00000001997		ENSGALT00000004455	DFFA
ENSGALT00000023409		ENSGALT00000004476	PEX14
ENSGALT00000002123	SERPIND1	ENSGALT00000004554	CASZ1
ENSGALT00000002184		ENSGALT00000018477	
ENSGALT00000002361		ENSGALT00000029510	
ENSGALT00000001634		ENSGALT00000025065	
ENSGALT00000003148	COMT	ENSGALT00000022896	NUDCD3
ENSGALT00000003199		ENSGALT00000025056	
ENSGALT00000003320		ENSGALT00000010437	
ENSGALT00000003339	PGAM5	ENSGALT00000004808	SRM
ENSGALT00000003358	ANKLE2	ENSGALT00000010461	
ENSGALT00000003414	CHFR	ENSGALT00000010508	
ENSGALT00000003714	PUS1	ENSGALT00000010517	
ENSGALT00000003784	ULK1	ENSGALT00000025246	SNX30
ENSGALT00000003883	MMP17	ENSGALT00000005284	MTOR

ENSGALT00000003918	SFSWAP	ENSGALT00000035033	ANGPTL 7
ENSGALT00000004249	SLC15A4	ENSGALT00000004844	EZH1
ENSGALT00000004598		ENSGALT00000005311	
ENSGALT00000004770	SCARB1	ENSGALT00000005447	PLEKHM 2
ENSGALT00000004933	NCOR2	ENSGALT00000025254	HSDL2
ENSGALT00000005063		ENSGALT00000025295	UGCG
ENSGALT00000005224		ENSGALT00000025296	
ENSGALT00000005240		ENSGALT00000025297	
ENSGALT00000005250		ENSGALT00000025304	KIAA0368
ENSGALT00000005393		ENSGALT00000028052	TRNT1
ENSGALT00000005860		ENSGALT00000013466	CRBN
ENSGALT00000005939	VPS37B	ENSGALT00000025324	
ENSGALT00000006066	ANAPC7	ENSGALT00000005919	FBXO42
ENSGALT00000006657		ENSGALT00000025350	SVEP1
ENSGALT00000001861	PIK3R6	ENSGALT00000005941	
ENSGALT00000026506	HSF1	ENSGALT00000013524	
ENSGALT00000000353	C2CD2L	ENSGALT00000013532	
ENSGALT00000007010	MLXIP	ENSGALT00000010797	
ENSGALT00000000380	HMBS	ENSGALT00000025366	
ENSGALT00000007031	DIABLO	ENSGALT00000032768	TOPORS
ENSGALT00000000393	VPS11	ENSGALT00000013590	LMCD1
ENSGALT00000000417	SIK2	ENSGALT00000013599	
ENSGALT00000007244	PPTC7	ENSGALT00000013623	
ENSGALT00000007277		ENSGALT00000018495	FAM134A
ENSGALT00000035085		ENSGALT00000004510	RUNDC1
ENSGALT00000000486		ENSGALT00000002747	PLAA
ENSGALT00000001417	PUS3	ENSGALT00000002781	IFT74
ENSGALT00000000525	DHDDS	ENSGALT00000002845	TEK
ENSGALT00000001501		ENSGALT00000002894	C9orf72
ENSGALT00000007523		ENSGALT00000036208	
ENSGALT00000001579		ENSGALT00000004453	VAT1
ENSGALT00000000760	CNKSR1	ENSGALT00000010935	
ENSGALT00000001619	DCPS	ENSGALT00000004366	NBR1
ENSGALT00000000781	LAPTM5	ENSGALT00000013700	
ENSGALT00000001710		ENSGALT00000013715	
ENSGALT00000001717		ENSGALT00000037566	
ENSGALT00000007562		ENSGALT00000003135	
ENSGALT00000001790	KCNJ5	ENSGALT00000003899	CYP27A1
ENSGALT00000001814		ENSGALT00000013770	IFT122
ENSGALT00000000876		ENSGALT00000029706	

ENSGALT00000001897		ENSGALT00000006036	
ENSGALT00000035194		ENSGALT00000013808	PLXND1
ENSGALT00000001951	PRDM10	ENSGALT00000006223	UBR4
ENSGALT00000001988		ENSGALT00000013831	TMCC1
ENSGALT00000000949	SCMH1	ENSGALT00000036642	
ENSGALT00000002027	ST14	ENSGALT00000003255	
ENSGALT00000038999		ENSGALT00000011543	AFF4
ENSGALT00000000957	CTPS1	ENSGALT00000003296	TSTD2
ENSGALT00000001016		ENSGALT00000013922	FGD5
ENSGALT00000010016		ENSGALT00000018542	TTLL4
ENSGALT00000002047	ZBTB44	ENSGALT00000003386	
ENSGALT00000002056		ENSGALT00000003423	
ENSGALT00000002090	ADAMTS8	ENSGALT00000011833	
ENSGALT00000022093	SENP1	ENSGALT00000003628	
ENSGALT00000001026	STX12	ENSGALT00000006321	
ENSGALT00000002140	ADAMTS15	ENSGALT00000018598	
ENSGALT00000001032		ENSGALT00000003696	ATG12
ENSGALT00000023447	HECTD4	ENSGALT00000011962	
ENSGALT00000001127		ENSGALT00000011976	
ENSGALT00000002230	JAM3	ENSGALT00000012114	LARS
ENSGALT00000002312	VPS26B	ENSGALT00000012177	TCERG1
ENSGALT00000002324		ENSGALT00000003821	
ENSGALT00000001233	KDM1A	ENSGALT00000034531	
ENSGALT00000002863		ENSGALT00000003846	BDP1
ENSGALT00000002903		ENSGALT00000003983	MCCC2
ENSGALT00000001253	ZMPSTE24	ENSGALT00000018650	TMBIM1
ENSGALT00000007760	FICD	ENSGALT00000012363	
ENSGALT00000003079	LDB3	ENSGALT00000018675	
ENSGALT00000010494	GRAMD1B	ENSGALT00000006425	
ENSGALT00000003119		ENSGALT00000006424	
ENSGALT00000010518	CLMP	ENSGALT00000039619	
ENSGALT00000003144		ENSGALT00000038163	
ENSGALT00000003257		ENSGALT00000004909	PINK1
ENSGALT00000010602	UBASH3B	ENSGALT00000022237	
ENSGALT00000007773	ISCU	ENSGALT00000018709	SMARCAL1
ENSGALT00000001788		ENSGALT00000013288	
ENSGALT00000001834	PPP2R3A	ENSGALT00000018729	XRCC5
ENSGALT00000001317		ENSGALT00000013349	PGGT1B
ENSGALT00000041155	FAM46B	ENSGALT00000013361	FEM1C
ENSGALT00000001355		ENSGALT00000013392	ALDH7A1
ENSGALT00000001392	WDTC1	ENSGALT00000018820	

ENSGALT00000010780	ARHGEF12	ENSGALT00000018822	
ENSGALT00000034383		ENSGALT00000008519	SRFBP1
ENSGALT00000001409	TMEM222	ENSGALT00000008552	SNCAIP
ENSGALT00000003816		ENSGALT00000029784	
ENSGALT00000010901	PVRL1	ENSGALT00000008572	PPIC
ENSGALT00000003908		ENSGALT00000008658	DCAF10
ENSGALT00000040822		ENSGALT00000018997	RALB
ENSGALT00000010949		ENSGALT00000008663	POLR1E
ENSGALT00000022698	WASF2	ENSGALT00000019039	NIFK
ENSGALT00000004014	CSGALNACT2	ENSGALT00000008587	ZBTB5
ENSGALT00000004133	BMS1	ENSGALT00000019041	
ENSGALT00000034212		ENSGALT00000008724	
ENSGALT00000004154		ENSGALT00000019090	DIRC2
ENSGALT00000041354		ENSGALT00000019105	SEMA5B
ENSGALT00000004232		ENSGALT00000019110	PDIA5
ENSGALT00000004246	FAM149B1	ENSGALT00000019136	
ENSGALT00000001414	YTHDF2	ENSGALT00000019216	
ENSGALT00000034167		ENSGALT00000019224	
ENSGALT00000004265		ENSGALT00000019286	HEG1
ENSGALT00000001474	MTFR1L	ENSGALT00000007199	MFN2
ENSGALT00000040809		ENSGALT00000007216	
ENSGALT00000001481		ENSGALT00000019689	SNX4
ENSGALT00000011217		ENSGALT00000007303	CLCN6
ENSGALT00000011287		ENSGALT00000019698	OSBPL11
ENSGALT00000001539	MAN1C1	ENSGALT00000007342	MTHFR
ENSGALT00000002654	DGKD	ENSGALT00000007392	
ENSGALT00000004359		ENSGALT00000032941	
ENSGALT00000001574	LDLRAP1	ENSGALT00000007609	C1QB
ENSGALT00000004372		ENSGALT00000007608	C1QA
ENSGALT00000011346	ZBTB16	ENSGALT00000019870	SLC35F5
ENSGALT00000011352		ENSGALT00000019884	NCKAP5
ENSGALT00000004487		ENSGALT00000019898	MGAT5
ENSGALT00000011363		ENSGALT00000034897	
ENSGALT00000004583		ENSGALT00000007659	C1orf50
ENSGALT00000001870		ENSGALT00000019996	R3HDM1
ENSGALT00000023514	CADM1	ENSGALT00000007741	
ENSGALT00000001916	CLIC4	ENSGALT00000020021	UBXN4
ENSGALT00000008249	KCTD10	ENSGALT00000007793	PPCS
ENSGALT00000001994	TRNAU1AP	ENSGALT00000008337	ANKRD1 3A
ENSGALT00000004680		ENSGALT00000020181	
ENSGALT00000002023		ENSGALT00000020184	

ENSGALT00000004693		ENSGALT00000020216	
ENSGALT00000011570		ENSGALT00000022876	
ENSGALT00000019416	SIDT2	ENSGALT00000020306	
ENSGALT00000002785	IWS1	ENSGALT00000016382	
ENSGALT00000004876		ENSGALT00000029632	DDI2
ENSGALT00000002149	SRSF4	ENSGALT00000020351	MBD5
ENSGALT00000005033		ENSGALT00000020382	
ENSGALT00000034008		ENSGALT00000029274	
ENSGALT00000011831	CEP164	ENSGALT00000020390	NMI
ENSGALT00000005241		ENSGALT00000020391	
ENSGALT00000002218	PDIK1L	ENSGALT00000020434	ARL5A
ENSGALT00000008853	ADRBK2	ENSGALT00000020462	PRPF40A
ENSGALT00000002281	UTP11L	ENSGALT00000037747	PRPF40A
ENSGALT00000002306	FHL3	ENSGALT00000020486	GPD2
ENSGALT00000003103	LIMS2	ENSGALT00000020497	
ENSGALT00000002444	INPP5B	ENSGALT00000020547	MARCH7
ENSGALT00000003177	WDR33	ENSGALT00000001983	
ENSGALT00000003200		ENSGALT00000001997	
ENSGALT00000011985	SCN4B	ENSGALT00000023409	
ENSGALT00000034294	SCN2B	ENSGALT00000002123	SERPIND<br>1
ENSGALT00000003399		ENSGALT00000002156	
ENSGALT00000003405	FAM168B	ENSGALT00000002172	YDJC
ENSGALT00000003431	ARHGEF4	ENSGALT00000002184	
ENSGALT00000011996		ENSGALT00000002268	PPM1F
ENSGALT00000011998		ENSGALT00000002361	
ENSGALT00000011874	TMPRSS5	ENSGALT00000002464	CDC45
ENSGALT00000003570		ENSGALT00000001634	
ENSGALT00000012038	IFT46	ENSGALT00000003148	COMT
ENSGALT00000000544		ENSGALT00000003199	
ENSGALT00000010877		ENSGALT00000003320	
ENSGALT00000012457	BCL9L	ENSGALT00000003339	PGAM5
ENSGALT00000010839	VPS8	ENSGALT00000003358	ANKLE2
ENSGALT00000003131	SNIP1	ENSGALT00000003414	CHFR
ENSGALT00000003145	ZC3H12A	ENSGALT00000003714	PUS1
ENSGALT00000012507	SLC37A4	ENSGALT00000003784	ULK1
ENSGALT00000009103	TTC28	ENSGALT00000003883	MMP17
ENSGALT00000005757	PTEN	ENSGALT00000003918	SFSWAP
ENSGALT00000005787	ATAD1	ENSGALT00000004041	GPR133
ENSGALT00000005844	PAPSS2	ENSGALT00000004249	SLC15A4
ENSGALT00000006037	PRKG1	ENSGALT00000004577	
ENSGALT00000009289	CCDC117	ENSGALT00000004598	

ENSGALT00000006268		ENSGALT00000004770	SCARB1
ENSGALT00000009327	KREMEN1	ENSGALT00000004933	NCOR2
ENSGALT00000006467	SLC25A16	ENSGALT00000005063	
ENSGALT00000006478	TET1	ENSGALT00000005160	EIF2B1
ENSGALT00000006533	CCAR1	ENSGALT00000005224	
ENSGALT00000006591		ENSGALT00000005240	
ENSGALT00000009461	DGCR14	ENSGALT00000005250	
ENSGALT00000009474	DGCR2	ENSGALT00000005393	
ENSGALT00000040808		ENSGALT00000005779	PITPNM2
ENSGALT00000006608		ENSGALT00000005860	
ENSGALT00000010597	DNAJB11	ENSGALT00000005939	VPS37B
ENSGALT00000035073	MAP7D1	ENSGALT00000006066	ANAPC7
ENSGALT00000006635	VPS26A	ENSGALT00000006388	CAMKK2
ENSGALT00000012830	DLAT	ENSGALT00000006657	
ENSGALT00000010575		ENSGALT00000001861	PIK3R6
ENSGALT00000003587		ENSGALT00000026506	HSF1
ENSGALT00000006771		ENSGALT00000000353	C2CD2L
ENSGALT00000003624		ENSGALT00000007010	MLXIP
ENSGALT00000006816	COL13A1	ENSGALT00000000380	HMBS
ENSGALT00000012907		ENSGALT00000007031	DIABLO
ENSGALT00000012936	ALG9	ENSGALT00000000393	VPS11
ENSGALT00000003529	AGO1	ENSGALT00000000417	SIK2
ENSGALT00000040712		ENSGALT00000007244	PPTC7
ENSGALT00000006896		ENSGALT00000007277	
ENSGALT00000012079		ENSGALT00000035085	
ENSGALT00000001980		ENSGALT00000000486	
ENSGALT00000009622		ENSGALT00000001417	PUS3
ENSGALT00000006946	MCU	ENSGALT00000001485	STT3A
ENSGALT00000010481	AMOTL2	ENSGALT00000000525	DHDDS
ENSGALT00000010418		ENSGALT00000001501	
ENSGALT00000010372	PAK2	ENSGALT00000007523	
ENSGALT00000035036	NFYC	ENSGALT00000001579	
ENSGALT00000008355		ENSGALT00000000760	CNKSR1
ENSGALT00000010254		ENSGALT00000001619	DCPS
ENSGALT00000010329	FBXO45	ENSGALT00000000781	LAPTM5
ENSGALT00000010325	WDR53	ENSGALT00000001710	
ENSGALT00000010312	UBXN7	ENSGALT00000001717	
ENSGALT00000010258	D2HGDH	ENSGALT00000007562	
ENSGALT00000007448	C10orf54	ENSGALT00000001790	KCNJ5
ENSGALT00000007605		ENSGALT00000001897	
ENSGALT00000007678		ENSGALT00000001951	PRDM10
ENSGALT00000007743	ZMIZ1	ENSGALT00000001988	

ENSGALT00000010319	CRKL	ENSGALT00000000949	SCMH1
ENSGALT00000010365	KLHL22	ENSGALT00000038999	
ENSGALT00000010179		ENSGALT00000000957	CTPS1
ENSGALT00000010381	MED15	ENSGALT00000010016	
ENSGALT00000010150	THAP4	ENSGALT00000002047	ZBTB44
ENSGALT00000010118	KLHL30	ENSGALT00000002056	
ENSGALT00000040502		ENSGALT00000002090	ADAMTS 8
ENSGALT00000007982		ENSGALT00000022093	SENP1
ENSGALT00000010504	GGT5	ENSGALT00000001026	STX12
ENSGALT00000005168	PTP4A2	ENSGALT00000002140	ADAMTS 15
ENSGALT00000010666	GUCD1	ENSGALT00000001032	
ENSGALT00000005230	FAM167B	ENSGALT00000023447	HECTD4
ENSGALT00000005249		ENSGALT00000001127	
ENSGALT00000008009	COMTD1	ENSGALT00000002230	JAM3
ENSGALT00000005346		ENSGALT00000002324	
ENSGALT00000008013	SAMD8	ENSGALT00000001233	KDM1A
ENSGALT00000008018		ENSGALT00000002439	
ENSGALT00000005386		ENSGALT00000002863	
ENSGALT00000008036		ENSGALT00000002903	
ENSGALT00000008109		ENSGALT00000001253	ZMPSTE 24
ENSGALT00000005407	ZBTB8A	ENSGALT00000007760	FICD
ENSGALT00000021437		ENSGALT00000003079	LDB3
ENSGALT00000008159	CAMK2G	ENSGALT00000010494	GRAMD1 B
ENSGALT00000004807		ENSGALT00000003119	
ENSGALT00000009365		ENSGALT00000010518	CLMP
ENSGALT00000009355		ENSGALT00000003144	
ENSGALT00000008188		ENSGALT00000003215	
ENSGALT00000009328	SEPT2	ENSGALT00000003257	
ENSGALT00000008243		ENSGALT00000010602	UBASH3 B
ENSGALT00000005575		ENSGALT00000007773	ISCU
ENSGALT00000008280		ENSGALT00000001788	
ENSGALT00000035005		ENSGALT00000001834	PPP2R3A
ENSGALT00000005643		ENSGALT00000001317	
ENSGALT00000008299		ENSGALT00000041155	FAM46B
ENSGALT00000008325	SEC24C	ENSGALT00000001355	
ENSGALT00000009299	FARP2	ENSGALT00000001392	WDTC1
ENSGALT00000005650	FNDC5	ENSGALT00000001977	

ENSGALT00000010902		ENSGALT00000010780	ARHGEF12
ENSGALT00000005857		ENSGALT00000034383	
ENSGALT00000008397	MYOZ1	ENSGALT00000001409	TMEM222
ENSGALT00000008407	USP54	ENSGALT00000034317	
ENSGALT00000008544	EIF3F	ENSGALT00000010901	PVRL1
ENSGALT00000008603	SORBS1	ENSGALT00000003908	
ENSGALT00000009101	PTTG1IP	ENSGALT00000010920	C1QTNF5
ENSGALT00000009089		ENSGALT00000040822	
ENSGALT00000033528		ENSGALT00000010949	
ENSGALT00000008913		ENSGALT00000022698	WASF2
ENSGALT00000008903		ENSGALT00000004014	CSGALNACT2
ENSGALT00000006028	PABPC4	ENSGALT00000004133	BMS1
ENSGALT00000008885		ENSGALT00000034212	
ENSGALT00000011126	PISD	ENSGALT00000004154	
ENSGALT00000006051	NT5C1A	ENSGALT00000041354	
ENSGALT00000008877	TM9SF3	ENSGALT00000004232	
ENSGALT00000011250	PIK3IP1	ENSGALT00000004246	FAM149B1
ENSGALT00000008940	NOLC1	ENSGALT00000001414	YTHDF2
ENSGALT00000002915		ENSGALT00000034167	
ENSGALT00000009008	PITX3	ENSGALT00000004265	
ENSGALT00000009044	GBF1	ENSGALT00000004276	IPMK
ENSGALT00000011276		ENSGALT00000001474	MTFR1L
ENSGALT00000009068		ENSGALT00000040809	
ENSGALT00000011283	C12orf43	ENSGALT00000001481	
ENSGALT00000003000	SLC25A24	ENSGALT00000011217	
ENSGALT00000009122	CUEDC2	ENSGALT00000011287	
ENSGALT00000011307	SPPL3	ENSGALT00000001539	MAN1C1
ENSGALT00000039703		ENSGALT00000002654	DGKD
ENSGALT00000006228	CAP1	ENSGALT00000001574	LDLRAP1
ENSGALT00000003064	PRPF38B	ENSGALT00000011352	
ENSGALT00000009141	HIF1AN	ENSGALT00000011363	
ENSGALT00000011502	MLEC	ENSGALT00000004583	
ENSGALT00000008412		ENSGALT00000023514	CADM1
ENSGALT00000034951		ENSGALT00000001916	CLIC4
ENSGALT00000008360	ACSL3	ENSGALT00000001959	
ENSGALT00000003286	CLCC1	ENSGALT00000008249	KCTD10
ENSGALT00000011568	RNF10	ENSGALT00000001994	TRNAU1

			AP
ENSGALT00000003326	WDR47	ENSGALT00000004680	
ENSGALT00000006375		ENSGALT00000002023	
ENSGALT00000008282		ENSGALT00000004693	
ENSGALT00000009416	ALOX5	ENSGALT00000011570	
ENSGALT00000028061	ZNF281	ENSGALT00000019416	SIDT2
ENSGALT00000011588		ENSGALT00000002785	IWS1
ENSGALT00000008246		ENSGALT00000004876	
ENSGALT00000008191	CUL3	ENSGALT00000002149	SRSF4
ENSGALT00000011648		ENSGALT00000034008	
ENSGALT00000006539	FUCA1	ENSGALT00000005241	
ENSGALT00000006584		ENSGALT00000002218	PDIK1L
ENSGALT00000003625	ZBTB41	ENSGALT00000008853	ADRBK2
ENSGALT00000011727		ENSGALT00000002256	
ENSGALT00000007951	IRS1	ENSGALT00000002281	UTP11L
ENSGALT00000011731		ENSGALT00000002306	FHL3
ENSGALT00000009862	MAPK8	ENSGALT00000003103	LIMS2
ENSGALT00000003831		ENSGALT00000002444	INPP5B
ENSGALT00000003897		ENSGALT00000003177	WDR33
ENSGALT00000004005		ENSGALT00000003200	
ENSGALT00000004201	OLFML2B	ENSGALT00000011985	SCN4B
ENSGALT00000004312	UHMK1	ENSGALT00000034294	SCN2B
ENSGALT00000010304	LIPA	ENSGALT00000003399	
ENSGALT00000004870	MFF	ENSGALT00000003405	FAM168B
ENSGALT00000011828		ENSGALT00000011996	
ENSGALT00000004824	AGFG1	ENSGALT00000011998	
ENSGALT00000010382	PANK1	ENSGALT00000003570	
ENSGALT00000004687		ENSGALT00000012038	IFT46
ENSGALT00000004623	TRIP12	ENSGALT00000000544	
ENSGALT00000010484	RPP30	ENSGALT00000010877	
ENSGALT00000010491		ENSGALT00000012457	BCL9L
ENSGALT00000033697		ENSGALT00000010839	VPS8
ENSGALT00000010507		ENSGALT00000003067	GNL2
ENSGALT00000011938	HSPB8	ENSGALT00000003145	ZC3H12A
ENSGALT00000010542	NOC3L	ENSGALT00000012507	SLC37A4
ENSGALT00000004729	PHGDH	ENSGALT00000009103	TTC28
ENSGALT00000011975		ENSGALT00000005757	PTEN
ENSGALT00000038257	VSIG10	ENSGALT00000005787	ATAD1
ENSGALT00000019223	PDE4DIP	ENSGALT00000041204	KY
ENSGALT00000004307		ENSGALT00000006037	PRKG1
ENSGALT00000002567		ENSGALT00000009289	CCDC11 7

ENSGALT00000004266	XRN1	ENSGALT00000006252	MYPN
ENSGALT00000004803	FAM78B	ENSGALT00000006268	
ENSGALT00000010939		ENSGALT00000009327	KREMEN1
ENSGALT00000011152		ENSGALT00000006467	SLC25A16
ENSGALT00000011201	CPEB3	ENSGALT00000006478	TET1
ENSGALT00000011235	BTAF1	ENSGALT00000006533	CCAR1
ENSGALT00000005208	PRRC2C	ENSGALT00000006591	
ENSGALT00000004131	PCOLCE2	ENSGALT00000003324	LSM10
ENSGALT00000004113	U2SURP	ENSGALT00000009461	DGCR14
ENSGALT00000003699	C3orf58	ENSGALT00000009474	DGCR2
ENSGALT00000005297		ENSGALT00000040808	
ENSGALT00000013157	CCDC88A	ENSGALT00000006608	
ENSGALT00000012100		ENSGALT00000036018	EVA1B
ENSGALT00000005458		ENSGALT00000010597	DNAJB11
ENSGALT00000012892	PNPT1	ENSGALT00000006635	VPS26A
ENSGALT00000012835	EFEMP1	ENSGALT00000012830	DLAT
ENSGALT00000011008		ENSGALT00000003587	
ENSGALT00000012809	VRK2	ENSGALT00000006771	
ENSGALT00000012797		ENSGALT00000006816	COL13A1
ENSGALT00000005530	ALDH9A1	ENSGALT00000012907	
ENSGALT00000012531	PRODH	ENSGALT00000012936	ALG9
ENSGALT00000039545	R3HCC1L	ENSGALT00000003529	AGO1
ENSGALT00000012578	OSBP2	ENSGALT00000040712	
ENSGALT00000005552		ENSGALT00000006896	
ENSGALT00000012708		ENSGALT00000012079	
ENSGALT00000005721	DARS2	ENSGALT00000001980	
ENSGALT00000009924		ENSGALT00000009622	
ENSGALT00000012192	ANKRD2	ENSGALT00000006946	MCU
ENSGALT00000005829		ENSGALT00000007055	SAR1A
ENSGALT00000006170		ENSGALT00000007067	PPA1
ENSGALT00000012894		ENSGALT00000010418	
ENSGALT00000012918		ENSGALT00000010372	PAK2
ENSGALT00000011075		ENSGALT00000035036	NFYC
ENSGALT00000006190	XPR1	ENSGALT00000008355	
ENSGALT00000011195	DLG1	ENSGALT00000010254	
ENSGALT00000006981		ENSGALT00000010286	CABIN1
ENSGALT00000033237	EXOSC1	ENSGALT00000010329	FBXO45
ENSGALT00000039466		ENSGALT00000010325	WDR53
ENSGALT00000013047		ENSGALT00000010312	UBXN7
ENSGALT00000006306	CEP350	ENSGALT00000010258	D2HGDH

ENSGALT00000013063	MTMR3	ENSGALT00000007448	C10orf54
ENSGALT00000013195		ENSGALT00000007605	
ENSGALT00000006691		ENSGALT00000007678	
ENSGALT00000011460	ATP13A3	ENSGALT00000007743	ZMIZ1
ENSGALT00000006699	ABL2	ENSGALT00000010319	CRKL
ENSGALT00000038026		ENSGALT00000010365	KLHL22
ENSGALT00000033698		ENSGALT00000010179	
ENSGALT00000012402	C10orf76	ENSGALT00000010381	MED15
ENSGALT00000013265		ENSGALT00000010150	THAP4
ENSGALT00000011583		ENSGALT00000010118	KLHL30
ENSGALT00000021800	CHPF2	ENSGALT00000010423	SMPD4
ENSGALT00000012505	FBXW4	ENSGALT00000040502	
ENSGALT00000021776	ASB10	ENSGALT00000007982	
ENSGALT00000011646	HRASLS	ENSGALT00000010504	GGT5
ENSGALT00000012535		ENSGALT00000005168	PTP4A2
ENSGALT00000011685	MB21D2	ENSGALT00000010666	GUCD1
ENSGALT00000011686		ENSGALT00000005230	FAM167B
ENSGALT00000007170	RFWD2	ENSGALT00000005249	
ENSGALT00000013435	ANAPC1	ENSGALT00000005281	
ENSGALT00000011822		ENSGALT00000033891	ADORA2A
ENSGALT00000013458		ENSGALT00000008009	COMTD1
ENSGALT00000033197	BTRC	ENSGALT00000005346	
ENSGALT00000011871		ENSGALT00000008013	SAMD8
ENSGALT00000011898		ENSGALT00000008018	
ENSGALT00000012719	KAZALD1	ENSGALT00000005386	
ENSGALT00000039302	SFXN3	ENSGALT00000008036	
ENSGALT00000007316	ZBTB37	ENSGALT00000008109	
ENSGALT00000007329	HEBP2	ENSGALT00000008126	
ENSGALT00000039298	LZTS2	ENSGALT00000021437	
ENSGALT00000028328		ENSGALT00000008159	CAMK2G
ENSGALT00000039413		ENSGALT00000004807	
ENSGALT00000012083	PCYT1A	ENSGALT00000009365	
ENSGALT00000013379		ENSGALT00000009355	
ENSGALT00000013406	MED13L	ENSGALT00000008188	
ENSGALT00000001591		ENSGALT00000009328	SEPT2
ENSGALT00000013781	RIN2	ENSGALT00000008243	
ENSGALT00000039933		ENSGALT00000005575	
ENSGALT00000013021	FAM178A	ENSGALT00000008280	
ENSGALT00000013045		ENSGALT00000035005	
ENSGALT00000013124		ENSGALT00000005601	KIAA1522
ENSGALT00000013893		ENSGALT00000005643	

ENSGALT00000013608	TPCN1	ENSGALT00000008299	
ENSGALT00000035381		ENSGALT00000008325	SEC24C
ENSGALT00000007904	MAP4	ENSGALT00000009299	FARP2
ENSGALT00000013911	KLHDC3	ENSGALT00000005650	FNDC5
ENSGALT00000013919		ENSGALT00000005690	AK2
ENSGALT00000031983	KLC4	ENSGALT00000010902	
ENSGALT00000033576	C1orf21	ENSGALT00000005857	
ENSGALT00000007675	EDEM3	ENSGALT00000008397	MYOZ1
ENSGALT00000012259		ENSGALT00000008407	USP54
ENSGALT00000007706	RNF2	ENSGALT00000008499	
ENSGALT00000012281	LRCH3	ENSGALT00000008544	EIF3F
ENSGALT00000013188		ENSGALT00000008603	SORBS1
ENSGALT00000014170		ENSGALT00000009101	PTTG1IP
ENSGALT00000031981		ENSGALT00000009089	
ENSGALT00000014195	KIF16B	ENSGALT00000021831	PPIE
ENSGALT00000008354	OLFM3	ENSGALT00000033528	
ENSGALT00000008454	SEC22C	ENSGALT00000008903	
ENSGALT00000015998	NKTR	ENSGALT00000006028	PABPC4
ENSGALT00000008401		ENSGALT00000008885	
ENSGALT00000033492		ENSGALT00000011126	PISD
ENSGALT00000008484	KLHL40	ENSGALT00000008618	MRPS22
ENSGALT00000008494		ENSGALT00000034973	HEYL
ENSGALT00000007577	OBSCN	ENSGALT00000006051	NT5C1A
ENSGALT00000008467		ENSGALT00000008877	TM9SF3
ENSGALT00000008474	LRRC39	ENSGALT00000011250	PIK3IP1
ENSGALT00000013503	SH3PXD2A	ENSGALT00000008940	NOLC1
ENSGALT00000012486		ENSGALT00000009008	PITX3
ENSGALT00000013539	SLK	ENSGALT00000009044	GBF1
ENSGALT00000033254	PDE6D	ENSGALT00000011276	
ENSGALT00000012571	COPS7B	ENSGALT00000009068	
ENSGALT00000014742		ENSGALT00000011283	C12orf43
ENSGALT00000014738		ENSGALT00000003000	SLC25A2<br>4
ENSGALT00000012599	DIS3L2	ENSGALT00000009122	CUEDC2
ENSGALT00000008548		ENSGALT00000011307	SPPL3
ENSGALT00000008553		ENSGALT00000006228	CAP1
ENSGALT00000008549	HIAT1	ENSGALT00000003064	PRPF38B
ENSGALT00000008683	AGL	ENSGALT00000009141	HIF1AN
ENSGALT00000014682	CLIP4	ENSGALT00000003258	GPSM2
ENSGALT00000039166		ENSGALT00000011502	MLEC
ENSGALT00000014608	TRMT61B	ENSGALT00000008412	
ENSGALT00000008691	SNAP47	ENSGALT00000034951	

ENSGALT00000008701		ENSGALT00000008360	ACSL3
ENSGALT00000008744		ENSGALT00000003286	CLCC1
ENSGALT00000008906	PTBP2	ENSGALT00000011568	RNF10
ENSGALT00000014542	FBXO11	ENSGALT00000003326	WDR47
ENSGALT00000008961	ALG14	ENSGALT00000006375	
ENSGALT00000014390		ENSGALT00000008282	
ENSGALT00000039077	CNN3	ENSGALT00000028061	ZNF281
ENSGALT00000008839	SETD2	ENSGALT00000011588	
ENSGALT00000014386		ENSGALT00000008246	
ENSGALT00000014378	VPS54	ENSGALT00000008191	CUL3
ENSGALT00000008983	TBRG4	ENSGALT00000011648	
ENSGALT00000014290	CEP68	ENSGALT00000006539	FUCA1
ENSGALT00000039079		ENSGALT00000006584	
ENSGALT00000009006		ENSGALT00000006787	NIPAL3
ENSGALT00000014275	SPRED2	ENSGALT00000003625	ZBTB41
ENSGALT00000012824		ENSGALT00000007951	IRS1
ENSGALT00000012833		ENSGALT00000011731	
ENSGALT00000012860	EIF4E2	ENSGALT00000009862	MAPK8
ENSGALT00000009078		ENSGALT00000003831	
ENSGALT00000014159		ENSGALT00000003897	
ENSGALT00000014258		ENSGALT00000004005	
ENSGALT00000014299	GPAM	ENSGALT00000004201	OLFML2B
ENSGALT00000009422		ENSGALT00000004223	
ENSGALT00000014409	ZDHHC6	ENSGALT00000004299	UAP1
ENSGALT00000021696	KDM2A	ENSGALT00000004312	UHMK1
ENSGALT00000014431		ENSGALT00000010304	LIPA
ENSGALT00000029637		ENSGALT00000004374	CRIP2
ENSGALT00000009565		ENSGALT00000004870	MFF
ENSGALT00000014249		ENSGALT00000011828	
ENSGALT00000033097		ENSGALT00000004824	AGFG1
ENSGALT00000009616	OXSR1	ENSGALT00000010311	
ENSGALT00000014566	C10orf118	ENSGALT00000010382	PANK1
ENSGALT00000014245		ENSGALT00000004687	
ENSGALT00000009277	TECR	ENSGALT00000004623	TRIP12
ENSGALT00000009287		ENSGALT00000010484	RPP30
ENSGALT00000031979		ENSGALT00000011938	HSPB8
ENSGALT00000009337	BCAR3	ENSGALT00000010562	TBC1D12
ENSGALT00000014639	AFAP1L2	ENSGALT00000010542	NOC3L
ENSGALT00000014777		ENSGALT00000010687	FRA10AC1
ENSGALT00000038916		ENSGALT00000004729	PHGDH

ENSGALT00000014757	ITPKB	ENSGALT00000011975	
ENSGALT00000009713		ENSGALT00000038257	VSIG10
ENSGALT00000014731		ENSGALT00000004494	
ENSGALT00000009417		ENSGALT00000019223	PDE4DIP
ENSGALT00000014779	FAM160B1	ENSGALT00000033985	
ENSGALT00000014689	BTBD3	ENSGALT00000004307	
ENSGALT00000014663		ENSGALT00000002567	
ENSGALT00000009497		ENSGALT00000004266	XRN1
ENSGALT00000009517	FAM69A	ENSGALT00000004803	FAM78B
ENSGALT00000014420	PLCB1	ENSGALT00000011152	
ENSGALT00000013545	RNPEPL1	ENSGALT00000011201	CPEB3
ENSGALT00000009966	XYLB	ENSGALT00000011235	BTAF1
ENSGALT00000014329	LRRN4	ENSGALT00000039690	
ENSGALT00000014324		ENSGALT00000005208	PRRC2C
ENSGALT00000014317		ENSGALT00000004131	PCOLCE 2
ENSGALT00000014238		ENSGALT00000004113	U2SURP
ENSGALT00000013645	EIF2B5	ENSGALT00000003699	C3orf58
ENSGALT00000013704	DVL3	ENSGALT00000005297	
ENSGALT00000013739		ENSGALT00000013157	CCDC88 A
ENSGALT00000013818	ABCF3	ENSGALT00000012474	CARD8
ENSGALT00000010074	GALNT11	ENSGALT00000012100	
ENSGALT00000009612	KIAA1107	ENSGALT00000005458	
ENSGALT00000014091		ENSGALT00000012892	PNPT1
ENSGALT00000014068	ACSS1	ENSGALT00000012835	EFEMP1
ENSGALT00000015143	RAB11FIP2	ENSGALT00000011008	
ENSGALT00000015155		ENSGALT00000012809	VRK2
ENSGALT00000033354		ENSGALT00000005530	ALDH9A1
ENSGALT00000010164		ENSGALT00000012531	PRODH
ENSGALT00000014035		ENSGALT00000039545	R3HCC1 L
ENSGALT00000009845	ZNF644	ENSGALT00000012578	OSBP2
ENSGALT00000015203		ENSGALT00000005552	
ENSGALT00000009867		ENSGALT00000012708	
ENSGALT00000009961	LRRC8C	ENSGALT00000005721	DARS2
ENSGALT00000009984	CCBL2	ENSGALT00000009924	
ENSGALT00000015262	PRDX3	ENSGALT00000005829	
ENSGALT00000028082		ENSGALT00000006170	
ENSGALT00000038716	RBM33	ENSGALT00000012894	
ENSGALT00000010087	PKN2	ENSGALT00000012918	
ENSGALT00000010099		ENSGALT00000011075	

ENSGALT00000010388	NOM1	ENSGALT00000006190	XPR1
ENSGALT00000010467		ENSGALT00000011195	DLG1
ENSGALT00000015358	BAG3	ENSGALT00000011255	
ENSGALT00000010570	ESYT2	ENSGALT00000006981	
ENSGALT00000013928		ENSGALT00000039466	
ENSGALT00000014106		ENSGALT00000013047	
ENSGALT00000015412	SEC23IP	ENSGALT00000033235	
ENSGALT00000015447	WDR11	ENSGALT00000013063	MTMR3
ENSGALT00000033012		ENSGALT00000006396	TOR1AIP 2
ENSGALT00000014128	SYDE2	ENSGALT00000013195	
ENSGALT00000010760	DIP2C	ENSGALT00000006691	
ENSGALT00000013868	ATL2	ENSGALT00000011460	ATP13A3
ENSGALT00000010875		ENSGALT00000038042	
ENSGALT00000001407	TEAD3	ENSGALT00000006699	ABL2
ENSGALT00000015475		ENSGALT00000038026	
ENSGALT00000014277	CTBS	ENSGALT00000033698	
ENSGALT00000001398		ENSGALT00000012402	C10orf76
ENSGALT00000013852	HNRNPLL	ENSGALT00000006824	
ENSGALT00000001203	MAPK14	ENSGALT00000013255	
ENSGALT00000011465	CCNY	ENSGALT00000013265	
ENSGALT00000011472		ENSGALT00000011583	
ENSGALT00000038945		ENSGALT00000013285	ERLEC1
ENSGALT00000011564		ENSGALT00000021800	CHPF2
ENSGALT00000013878	EIF4G1	ENSGALT00000012505	FBXW4
ENSGALT00000011573		ENSGALT00000021776	ASB10
ENSGALT00000014453	ELTD1	ENSGALT00000011646	HRASLS
ENSGALT00000015724		ENSGALT00000012535	
ENSGALT00000001098	TBC1D22B	ENSGALT00000011685	MB21D2
ENSGALT00000014497	DNAJB4	ENSGALT00000011686	
ENSGALT00000014545	NEXN	ENSGALT00000007170	RFWD2
ENSGALT00000015848		ENSGALT00000013400	
ENSGALT00000023376	DHX57	ENSGALT00000013435	ANAPC1
ENSGALT00000038643	ZRANB1	ENSGALT00000011822	
ENSGALT00000022257	UROS	ENSGALT00000013458	
ENSGALT00000010681	BCCIP	ENSGALT00000033197	BTRC
ENSGALT00000014667		ENSGALT00000011871	
ENSGALT00000001035	CACNA1S	ENSGALT00000011898	
ENSGALT00000040899		ENSGALT00000012719	KAZALD1
ENSGALT00000021600	SOS1	ENSGALT00000039302	SFXN3
ENSGALT00000011752		ENSGALT00000039301	PDZD7
ENSGALT00000011888	MTPAP	ENSGALT00000007316	ZBTB37

ENSGALT00000011932	WAC	ENSGALT00000007329	HEBP2
ENSGALT00000011983	MPP7	ENSGALT00000039298	LZTS2
ENSGALT00000012033		ENSGALT00000028328	
ENSGALT00000012112		ENSGALT00000039413	
ENSGALT00000014845	THUMPD2	ENSGALT00000012083	PCYT1A
ENSGALT00000016231		ENSGALT00000013379	
ENSGALT00000004009		ENSGALT00000013406	MED13L
ENSGALT00000016237		ENSGALT00000001591	
ENSGALT00000004002	SHF	ENSGALT00000007437	SMG7
ENSGALT00000003975		ENSGALT00000013781	RIN2
ENSGALT00000003910	ALPK3	ENSGALT00000013021	FAM178A
ENSGALT00000014863		ENSGALT00000013045	
ENSGALT00000003900	ZNF592	ENSGALT00000013124	
ENSGALT00000014913	CHGB	ENSGALT00000013893	
ENSGALT00000016376	KDM4A	ENSGALT00000013608	TPCN1
ENSGALT00000014998	CRLS1	ENSGALT00000039377	
ENSGALT00000017035		ENSGALT00000035381	
ENSGALT00000000712	KLHL12	ENSGALT00000007904	MAP4
ENSGALT00000015001		ENSGALT00000008045	
ENSGALT00000012598	KIAA1217	ENSGALT00000013899	PPP2R5 D
ENSGALT00000012794		ENSGALT00000013911	KLHDC3
ENSGALT00000038548	ST3GAL3	ENSGALT00000013919	
ENSGALT00000000542	IPO9	ENSGALT00000031983	KLC4
ENSGALT00000017059	BNIP3	ENSGALT00000033576	C1orf21
ENSGALT00000015061	SDE2	ENSGALT00000007675	EDEM3
ENSGALT00000017087	INPP5A	ENSGALT00000012259	
ENSGALT00000003644	SORD	ENSGALT00000007706	RNF2
ENSGALT00000015087	TMEM63A	ENSGALT00000012281	LRCH3
ENSGALT00000017131	ALDH18A1	ENSGALT00000008125	
ENSGALT00000003712		ENSGALT00000013188	
ENSGALT00000012922	PLXDC2	ENSGALT00000014170	
ENSGALT00000013939		ENSGALT00000013262	CNNM2
ENSGALT00000014060		ENSGALT00000031981	
ENSGALT00000003690	ZNF609	ENSGALT00000014195	KIF16B
ENSGALT00000015154	WDR26	ENSGALT00000008265	
ENSGALT00000016414		ENSGALT00000013413	
ENSGALT00000000450	PPP1R12B	ENSGALT00000008354	OLFM3
ENSGALT00000015194		ENSGALT00000008454	SEC22C
ENSGALT00000015195	FBXO28	ENSGALT00000015998	NKTR
ENSGALT00000014097	STAM	ENSGALT00000008401	
ENSGALT00000014104	PTPLA	ENSGALT00000033492	

ENSGALT00000014117		ENSGALT00000008484	KLHL40
ENSGALT00000015209	TP53BP2	ENSGALT00000008494	
ENSGALT00000016418		ENSGALT00000007577	OBSCN
ENSGALT00000016432	DMAP1	ENSGALT00000008467	
ENSGALT00000016435	ERI3	ENSGALT00000008481	TRMT13
ENSGALT00000016441		ENSGALT00000013503	SH3PXD2A
ENSGALT00000015319	DISP1	ENSGALT00000012486	
ENSGALT00000015332	BROX	ENSGALT00000013519	OBFC1
ENSGALT00000014205	PTER	ENSGALT00000008503	
ENSGALT00000014135		ENSGALT00000013539	SLK
ENSGALT00000014256	FAM171A1	ENSGALT00000033254	PDE6D
ENSGALT00000016472	PLK3	ENSGALT00000012571	COPS7B
ENSGALT00000015381		ENSGALT00000014742	
ENSGALT00000031228	ACBD7	ENSGALT00000014738	
ENSGALT00000015385		ENSGALT00000012599	DIS3L2
ENSGALT00000014327	CROT	ENSGALT00000008548	
ENSGALT00000015441	MARK1	ENSGALT00000008553	
ENSGALT00000015469		ENSGALT00000008549	HIAT1
ENSGALT00000034751		ENSGALT00000008683	AGL
ENSGALT00000040700		ENSGALT00000014682	CLIP4
ENSGALT00000002999		ENSGALT00000039166	
ENSGALT00000000195		ENSGALT00000014608	TRMT61B
ENSGALT00000031945	TGFB2	ENSGALT00000008691	SNAP47
ENSGALT00000015667	RRP15	ENSGALT00000008701	
ENSGALT00000033152		ENSGALT00000008744	
ENSGALT00000002757	ADPGK	ENSGALT00000008906	PTBP2
ENSGALT00000015752	KCTD3	ENSGALT00000014542	FBXO11
ENSGALT00000015916	PTPN14	ENSGALT00000008961	ALG14
ENSGALT00000015924		ENSGALT00000013697	
ENSGALT00000000132		ENSGALT00000014390	
ENSGALT00000015927		ENSGALT00000039077	CNN3
ENSGALT00000031937	RPS6KC1	ENSGALT00000008839	SETD2
ENSGALT00000015943		ENSGALT00000014386	
ENSGALT00000000123		ENSGALT00000014378	VPS54
ENSGALT00000031934	VASH2	ENSGALT00000013886	XPNPEP1
ENSGALT00000016627	TOE1	ENSGALT00000008983	TBRG4
ENSGALT00000031932	BATF3	ENSGALT00000014290	CEP68
ENSGALT00000016648		ENSGALT00000039079	
ENSGALT00000015969	NENF	ENSGALT00000009006	

ENSGALT00000007640	FGR	ENSGALT00000014275	SPRED2
ENSGALT00000016679	NASP	ENSGALT00000012824	
ENSGALT00000015980	PPP2R5A	ENSGALT00000031288	
ENSGALT00000014487		ENSGALT00000012833	
ENSGALT00000015176		ENSGALT00000013979	
ENSGALT00000022855	COX5A	ENSGALT00000012860	EIF4E2
ENSGALT00000016009		ENSGALT00000009078	
ENSGALT00000002612	MPI	ENSGALT00000014159	
ENSGALT00000016032	SLC30A1	ENSGALT00000014258	
ENSGALT00000016037		ENSGALT00000014299	GPAM
ENSGALT00000000700	KCTD20	ENSGALT00000009158	
ENSGALT00000000721	STK38	ENSGALT00000009422	
ENSGALT00000015304	ANKIB1	ENSGALT00000014409	ZDHHC6
ENSGALT00000002583	SCAMP2	ENSGALT00000021696	KDM2A
ENSGALT00000014768		ENSGALT00000014431	
ENSGALT00000016717	TMEM69	ENSGALT00000029637	
ENSGALT00000022351	MRPL47	ENSGALT00000009565	
ENSGALT00000016788	MAST2	ENSGALT00000014249	
ENSGALT00000016854	POMGNT1	ENSGALT00000009616	OXSR1
ENSGALT00000035250		ENSGALT00000014566	C10orf118
ENSGALT00000028064	LURAP1	ENSGALT00000014245	
ENSGALT00000000779	PI16	ENSGALT00000009287	
ENSGALT00000000243	MTCH1	ENSGALT00000031979	
ENSGALT00000015324		ENSGALT00000009337	BCAR3
ENSGALT00000016077		ENSGALT00000014639	AFAP1L2
ENSGALT00000015329	PEX1	ENSGALT00000014777	
ENSGALT00000028065		ENSGALT00000014757	ITPKB
ENSGALT00000015427		ENSGALT00000009713	
ENSGALT00000002097		ENSGALT00000014731	
ENSGALT00000016989	FAAH	ENSGALT00000009417	
ENSGALT00000002152		ENSGALT00000014779	FAM160B1
ENSGALT00000000864	PPP1R15B	ENSGALT00000014689	BTBD3
ENSGALT00000000884	PIK3C2B	ENSGALT00000014663	
ENSGALT00000016111	UBR2	ENSGALT00000009497	
ENSGALT00000015485	TFPI2	ENSGALT00000009517	FAM69A
ENSGALT00000016119	GLTSCR1L	ENSGALT00000014420	PLCB1
ENSGALT00000028081	PKDCC	ENSGALT00000013545	RNPEPL1
ENSGALT00000017005	ATPAF1	ENSGALT00000009966	XYLB
ENSGALT00000015498		ENSGALT00000014329	LRRN4

ENSGALT00000041398		ENSGALT00000014324	
ENSGALT00000014612	GNB4	ENSGALT00000014317	
ENSGALT00000017008		ENSGALT00000014238	
ENSGALT00000000908	MDM4	ENSGALT00000013645	EIF2B5
ENSGALT00000017068		ENSGALT00000013704	DVL3
ENSGALT00000000162	ZMAT3	ENSGALT00000013739	
ENSGALT00000002511	SIN3A	ENSGALT00000013818	ABCF3
ENSGALT00000017154		ENSGALT00000014157	
ENSGALT00000016201	LRPPRC	ENSGALT00000010074	GALNT11
ENSGALT00000004220	CSPG4	ENSGALT00000009612	KIAA1107
ENSGALT00000014937	NCEH1	ENSGALT00000014091	
ENSGALT00000031920		ENSGALT00000014068	ACSS1
ENSGALT00000004357		ENSGALT00000015143	RAB11FIP2
ENSGALT00000004380	TSPAN3	ENSGALT00000015155	
ENSGALT00000015009		ENSGALT00000033354	
ENSGALT00000015057	PLD1	ENSGALT00000010164	
ENSGALT00000035232		ENSGALT00000014035	
ENSGALT00000000950	TMCC2	ENSGALT00000009845	ZNF644
ENSGALT00000038337		ENSGALT00000015203	
ENSGALT00000016234	SRBD1	ENSGALT00000009867	
ENSGALT00000016247	PRKCE	ENSGALT00000009961	LRRC8C
ENSGALT00000004485	RCN2	ENSGALT00000009984	CCBL2
ENSGALT00000004731	UBE2Q2	ENSGALT00000015262	PRDX3
ENSGALT00000015276		ENSGALT00000010241	PAXIP1
ENSGALT00000000994	ELK4	ENSGALT00000028082	
ENSGALT00000001004		ENSGALT00000010087	PKN2
ENSGALT00000016253		ENSGALT00000010099	
ENSGALT00000001018	SLC41A1	ENSGALT00000010388	NOM1
ENSGALT00000017199	TXNDC12	ENSGALT00000015343	RGS10
ENSGALT00000017200		ENSGALT00000010467	
ENSGALT00000031915	PIGF	ENSGALT00000010570	ESYT2
ENSGALT00000016264		ENSGALT00000015375	INPP5F
ENSGALT00000017269	ZFYVE9	ENSGALT00000013928	
ENSGALT00000016269		ENSGALT00000015412	SEC23IP
ENSGALT00000016270		ENSGALT00000015447	WDR11
ENSGALT00000015328	MYNN	ENSGALT00000033012	
ENSGALT00000001027		ENSGALT00000014116	
ENSGALT00000031908		ENSGALT00000014128	SYDE2
ENSGALT00000017276	CC2D1B	ENSGALT00000010760	DIP2C
ENSGALT00000015429		ENSGALT00000013868	ATL2
ENSGALT00000016303		ENSGALT00000010875	

ENSGALT00000016306		ENSGALT00000015475	
ENSGALT00000016321	RBKS	ENSGALT00000014277	CTBS
ENSGALT00000016329	SUPT7L	ENSGALT00000001398	
ENSGALT00000017295	ZCCHC11	ENSGALT00000010908	WDR37
ENSGALT00000001189	SRGAP2	ENSGALT00000013852	HNRNPL L
ENSGALT00000015740	SGCE	ENSGALT00000001203	MAPK14
ENSGALT00000001255		ENSGALT00000011465	CCNY
ENSGALT00000001265	DYRK3	ENSGALT00000011472	
ENSGALT00000001297	MAPKAPK2	ENSGALT00000022442	GALM
ENSGALT00000016469	GLO1	ENSGALT00000038945	
ENSGALT00000015772	ASB4	ENSGALT00000011564	
ENSGALT00000015790		ENSGALT00000014411	
ENSGALT00000017326	SCP2	ENSGALT00000013878	EIF4G1
ENSGALT00000034634		ENSGALT00000011573	
ENSGALT00000040356		ENSGALT00000014453	ELTD1
ENSGALT00000005141	DNAJA4	ENSGALT00000015724	
ENSGALT00000015833		ENSGALT00000001098	TBC1D22 B
ENSGALT00000016480	ZFAND3	ENSGALT00000014545	NEXN
ENSGALT00000005233		ENSGALT00000015848	
ENSGALT00000015869		ENSGALT00000023376	DHX57
ENSGALT00000005261	TBC1D2B	ENSGALT00000022257	UROS
ENSGALT00000005279	DAPK2	ENSGALT00000010681	BCCIP
ENSGALT00000005378	FBXL22	ENSGALT00000014667	
ENSGALT00000005420	USP3	ENSGALT00000001035	CACNA1 S
ENSGALT00000005515	RAB8B	ENSGALT00000040899	
ENSGALT00000017402	MIOS	ENSGALT00000000815	PTPRH
ENSGALT00000016499	CMTR1	ENSGALT00000021600	SOS1
ENSGALT00000017329	PODN	ENSGALT00000011752	
ENSGALT00000001781		ENSGALT00000020791	
ENSGALT00000017404	RPA3	ENSGALT00000011883	KIAA1462
ENSGALT00000037997	RPA3-AS1	ENSGALT00000011888	MTPAP
ENSGALT00000001786	CD34	ENSGALT00000011932	WAC
ENSGALT00000015516	NMD3	ENSGALT00000011983	MPP7
ENSGALT00000015523	PPM1L	ENSGALT00000012033	
ENSGALT00000015544		ENSGALT00000012112	
ENSGALT00000039589		ENSGALT00000014845	THUMPD 2
ENSGALT00000039192		ENSGALT00000016208	
ENSGALT00000038176	RNF8	ENSGALT00000016231	

ENSGALT00000015632		ENSGALT00000004009	
ENSGALT00000016540	SLC35B2	ENSGALT00000016237	
ENSGALT00000015688	GFM1	ENSGALT00000004002	SHF
ENSGALT00000016551	SLC29A1	ENSGALT00000003975	
ENSGALT00000038145		ENSGALT00000003910	ALPK3
ENSGALT00000039093	MLF1	ENSGALT00000013907	
ENSGALT00000016725	TMEM63B	ENSGALT00000014863	
ENSGALT00000017557	ISPD	ENSGALT00000003900	ZNF592
ENSGALT00000017469	NDC1	ENSGALT00000014913	CHGB
ENSGALT00000017477		ENSGALT00000016376	KDM4A
ENSGALT00000002207	ATP5F1	ENSGALT00000014998	CRLS1
ENSGALT00000015739	RSRC1	ENSGALT00000029070	MGMT
ENSGALT00000002238	RAP1A	ENSGALT00000017035	
ENSGALT00000032981	C3orf55	ENSGALT00000000712	KLHL12
ENSGALT00000017661		ENSGALT00000012469	
ENSGALT00000017484		ENSGALT00000015001	
ENSGALT00000017487		ENSGALT00000012598	KIAA1217
ENSGALT00000016736	MRPL14	ENSGALT00000012794	
ENSGALT00000017736	SP4	ENSGALT00000038548	ST3GAL3
ENSGALT00000017745	RAPGEF5	ENSGALT00000000542	IPO9
ENSGALT00000017540	ACOT11	ENSGALT00000017059	BNIP3
ENSGALT00000002440	PPM1J	ENSGALT00000017087	INPP5A
ENSGALT00000005967		ENSGALT00000003644	SORD
ENSGALT00000016751		ENSGALT00000015087	TMEM63A
ENSGALT00000016682	TIPARP	ENSGALT00000017131	ALDH18A1
ENSGALT00000002661	LRIG2	ENSGALT00000003712	
ENSGALT00000006011	BNIP2	ENSGALT00000012922	PLXDC2
ENSGALT00000016793	GTPBP2	ENSGALT00000013939	
ENSGALT00000006179	MTMR10	ENSGALT00000003690	ZNF609
ENSGALT00000017568	USP24	ENSGALT00000003646	TRIP4
ENSGALT00000017589		ENSGALT00000016414	
ENSGALT00000016730		ENSGALT00000000450	PPP1R12B
ENSGALT00000016835		ENSGALT00000034726	SNX1
ENSGALT00000016761	SLC33A1	ENSGALT00000015194	
ENSGALT00000017826		ENSGALT00000015195	FBXO28
ENSGALT00000017301		ENSGALT00000014097	STAM
ENSGALT00000003002	HIPK1	ENSGALT00000014104	PTPLA
ENSGALT00000006206	MPHOSPH10	ENSGALT00000014117	
ENSGALT00000017683	FGGY	ENSGALT00000003234	MYO9A

ENSGALT00000017829	MALSU1	ENSGALT00000015209	TP53BP2
ENSGALT00000016815		ENSGALT00000016418	
ENSGALT00000003070	TRIM33	ENSGALT00000016432	DMAP1
ENSGALT00000017877		ENSGALT00000016435	ERI3
ENSGALT00000017882	CCDC126	ENSGALT00000016441	
ENSGALT00000034521	MCEE	ENSGALT00000015319	DISP1
ENSGALT00000017975		ENSGALT00000015332	BROX
ENSGALT00000006347	TARSL2	ENSGALT00000014205	PTER
ENSGALT00000006419	TM2D3	ENSGALT00000014135	
ENSGALT00000017979		ENSGALT00000003113	GRAMD2
ENSGALT00000032964	TM2D1	ENSGALT00000016462	KIF2C
ENSGALT00000018006		ENSGALT00000014256	FAM171A1
ENSGALT00000006440	LARP6	ENSGALT00000016472	PLK3
ENSGALT00000016864		ENSGALT00000015381	
ENSGALT00000006501	THSD4	ENSGALT00000015385	
ENSGALT00000018005		ENSGALT00000014327	CROT
ENSGALT00000017894	ATG4C	ENSGALT00000015441	MARK1
ENSGALT00000018034		ENSGALT00000015469	
ENSGALT00000018085		ENSGALT00000034751	
ENSGALT00000018131		ENSGALT00000040700	
ENSGALT00000016884		ENSGALT00000002999	
ENSGALT00000018137		ENSGALT00000000195	
ENSGALT00000017947		ENSGALT00000031945	TGFB2
ENSGALT00000018161	CREB5	ENSGALT00000033152	
ENSGALT00000018199	CHN2	ENSGALT00000002757	ADPGK
ENSGALT00000006663	SLTM	ENSGALT00000015752	KCTD3
ENSGALT00000017953		ENSGALT00000015916	PTPN14
ENSGALT00000017959	CACHD1	ENSGALT00000015924	
ENSGALT00000016916	TJAP1	ENSGALT00000000132	
ENSGALT00000017964	RAVER2	ENSGALT00000031937	RPS6KC1
ENSGALT00000003247		ENSGALT00000015943	
ENSGALT00000018212	WIPF3	ENSGALT00000000123	
ENSGALT00000018231	FKBP14	ENSGALT00000031934	VASH2
ENSGALT00000018282		ENSGALT00000014615	
ENSGALT00000034417		ENSGALT00000014450	
ENSGALT00000018291		ENSGALT00000016648	
ENSGALT00000018312	ANKRD28	ENSGALT00000015969	NENF
ENSGALT00000018324	GALNT15	ENSGALT00000016679	NASP
ENSGALT00000018327	DPH3	ENSGALT00000000439	
ENSGALT00000016977	ZNF318	ENSGALT00000015980	PPP2R5A

ENSGALT00000016982		ENSGALT00000014487	
ENSGALT00000018329		ENSGALT00000014750	CDK14
ENSGALT00000017012	LTBP3	ENSGALT00000015176	
ENSGALT00000018331		ENSGALT00000022855	COX5A
ENSGALT00000017995		ENSGALT00000015990	
ENSGALT00000040028		ENSGALT00000016009	
ENSGALT00000018348	PLCL2	ENSGALT00000002612	MPI
ENSGALT00000022526	MNS1	ENSGALT00000016017	
ENSGALT00000018402	KAT2B	ENSGALT00000015253	
ENSGALT00000016930	SIAH2	ENSGALT00000016032	SLC30A1
ENSGALT00000001028	RFX7	ENSGALT00000016037	
ENSGALT00000016934		ENSGALT00000000700	KCTD20
ENSGALT00000018125	SLC35D1	ENSGALT00000000721	STK38
ENSGALT00000017165		ENSGALT00000016049	RCOR3
ENSGALT00000006970		ENSGALT00000015304	ANKIB1
ENSGALT00000038194		ENSGALT00000002583	SCAMP2
ENSGALT00000018407	ZNF385D	ENSGALT00000014768	
ENSGALT00000018414	UBE2E1	ENSGALT00000016717	TMEM69
ENSGALT00000007059		ENSGALT00000022351	MRPL47
ENSGALT00000018418	NKIRAS1	ENSGALT00000016788	MAST2
ENSGALT00000016952	WWTR1	ENSGALT00000016854	POMGNT1
ENSGALT00000007264		ENSGALT00000035250	
ENSGALT00000017201	HEATR5B	ENSGALT00000028064	LURAP1
ENSGALT00000017218	STRN	ENSGALT00000000779	PI16
ENSGALT00000039081		ENSGALT00000000243	MTCH1
ENSGALT00000018328		ENSGALT00000015310	
ENSGALT00000018364		ENSGALT00000016072	
ENSGALT00000016991	CP	ENSGALT00000015324	
ENSGALT00000018434		ENSGALT00000016077	
ENSGALT00000037966		ENSGALT00000028065	
ENSGALT00000018443		ENSGALT00000015427	
ENSGALT00000018455	ANKRD13C	ENSGALT00000015428	
ENSGALT00000016995	HPS3	ENSGALT00000002097	
ENSGALT00000017268	PRKD3	ENSGALT00000016989	FAAH
ENSGALT00000017274	QPCT	ENSGALT00000000864	PPP1R15B
ENSGALT00000017275		ENSGALT00000000884	PIK3C2B
ENSGALT00000018537	TYW3	ENSGALT00000016111	UBR2
ENSGALT00000004361	UHRF1BP1	ENSGALT00000015485	TFPI2
ENSGALT00000017280		ENSGALT00000016119	GLTSCR1L

ENSGALT00000018563	SLC44A5	ENSGALT00000028081	PKDCC
ENSGALT00000017288		ENSGALT00000017005	ATPAF1
ENSGALT00000018575	RABGGTB	ENSGALT00000015498	
ENSGALT00000018582	MSH4	ENSGALT00000041398	
ENSGALT00000018458	LRRC3B	ENSGALT00000014612	GNB4
ENSGALT00000017325	CNST	ENSGALT00000017008	
ENSGALT00000017328	TFB2M	ENSGALT00000000908	MDM4
ENSGALT00000004387		ENSGALT00000017068	
ENSGALT00000037849		ENSGALT00000002409	
ENSGALT00000018603	SLC4A7	ENSGALT00000000162	ZMAT3
ENSGALT00000018638	AZI2	ENSGALT00000002511	SIN3A
ENSGALT00000018667	GADL1	ENSGALT00000017152	CDKN2C
ENSGALT00000017370		ENSGALT00000017154	
ENSGALT00000017414	ZBTB18	ENSGALT00000016201	LRPPRC
ENSGALT00000017430	SDCCAG8	ENSGALT00000004220	CSPG4
ENSGALT00000017461	CEP170	ENSGALT00000014937	NCEH1
ENSGALT00000017613		ENSGALT00000031920	
ENSGALT00000018718	GPD1L	ENSGALT00000004357	
ENSGALT00000000338	HEATR1	ENSGALT00000004380	TSPAN3
ENSGALT00000004468		ENSGALT00000015009	
ENSGALT00000017618	ERO1LB	ENSGALT00000015057	PLD1
ENSGALT00000017890	NID1	ENSGALT00000035232	
ENSGALT00000018725		ENSGALT00000000950	TMCC2
ENSGALT00000018727	CMTM6	ENSGALT00000038337	
ENSGALT00000004481	GRM4	ENSGALT00000016234	SRBD1
ENSGALT00000035045		ENSGALT00000004485	RCN2
ENSGALT00000004543		ENSGALT00000004731	UBE2Q2
ENSGALT00000018736	TCAIM	ENSGALT00000015276	
ENSGALT00000022610	B3GALNT2	ENSGALT00000000994	ELK4
ENSGALT00000004102	GGPS1	ENSGALT00000001004	
ENSGALT00000000385	ARID4B	ENSGALT00000016253	
ENSGALT00000007734	SEMA6D	ENSGALT00000001018	SLC41A1
ENSGALT00000018770	ANO10	ENSGALT00000017199	TXNDC12
ENSGALT00000022567	TOMM20	ENSGALT00000017200	
ENSGALT00000018780		ENSGALT00000031915	PIGF
ENSGALT00000018812	EAF1	ENSGALT00000016264	
ENSGALT00000017944		ENSGALT00000017269	ZFYVE9
ENSGALT00000018839		ENSGALT00000016269	
ENSGALT00000018846	CAPN7	ENSGALT00000016270	
ENSGALT00000018026		ENSGALT00000015328	MYNN
ENSGALT00000018060	RAB4A	ENSGALT00000001027	

ENSGALT00000009049	CEP152	ENSGALT00000015414	
ENSGALT00000018064		ENSGALT00000015416	
ENSGALT00000018070	NUP133	ENSGALT00000031908	
ENSGALT00000034280	SECISBP2L	ENSGALT00000017276	CC2D1B
ENSGALT00000018081	TAF5L	ENSGALT00000015429	
ENSGALT00000018087	URB2	ENSGALT00000016303	
ENSGALT00000005108	APOBEC2	ENSGALT00000016306	
ENSGALT00000018092	GALNT2	ENSGALT00000016321	RBKS
ENSGALT00000005111		ENSGALT00000016329	SUPT7L
ENSGALT00000018903	ATP2C1	ENSGALT00000001149	
ENSGALT00000031841	C1orf131	ENSGALT00000017295	ZCCHC1<br>1
ENSGALT00000018198	GNPAT	ENSGALT00000001189	SRGAP2
ENSGALT00000018210		ENSGALT00000015740	SGCE
ENSGALT00000018940		ENSGALT00000001255	
ENSGALT00000018217		ENSGALT00000017302	
ENSGALT00000018228	FAM120B	ENSGALT00000025940	
ENSGALT00000018250	PHF10	ENSGALT00000001265	DYRK3
ENSGALT00000018251		ENSGALT00000001297	MAPKAP<br>K2
ENSGALT00000019087	DNAJC13	ENSGALT00000016469	GLO1
ENSGALT00000018269	SMOC2	ENSGALT00000015772	ASB4
ENSGALT00000005461	FRS3	ENSGALT00000017326	SCP2
ENSGALT00000009208		ENSGALT00000034634	
ENSGALT00000009244		ENSGALT00000040356	
ENSGALT00000005500		ENSGALT00000005141	DNAJA4
ENSGALT00000005492	BYSL	ENSGALT00000015833	
ENSGALT00000009484	USP8	ENSGALT00000016480	ZFAND3
ENSGALT00000018633	FGFR1OP	ENSGALT00000005233	
ENSGALT00000037674		ENSGALT00000015869	
ENSGALT00000018857	PARK2	ENSGALT00000005261	TBC1D2B
ENSGALT00000018893	MAP3K4	ENSGALT00000005378	FBXL22
ENSGALT00000019134		ENSGALT00000005420	USP3
ENSGALT00000038740		ENSGALT00000005515	RAB8B
ENSGALT00000037615	CDV3	ENSGALT00000017402	MIOS
ENSGALT00000009646	FSD2	ENSGALT00000016499	CMTR1
ENSGALT00000009670		ENSGALT00000017329	PODN
ENSGALT00000037604		ENSGALT00000001781	
ENSGALT00000009692		ENSGALT00000017404	RPA3
ENSGALT00000009702		ENSGALT00000037997	RPA3-AS<br>1
ENSGALT00000018986		ENSGALT00000001786	CD34

ENSGALT00000019010		ENSGALT00000015516	NMD3
ENSGALT00000005608		ENSGALT00000015523	PPM1L
ENSGALT00000005617	PRELP	ENSGALT00000039589	
ENSGALT00000019329	SACM1L	ENSGALT00000039192	
ENSGALT00000010337		ENSGALT00000038176	RNF8
ENSGALT00000019332	LIMD1	ENSGALT00000015632	
ENSGALT00000019376	LARS2	ENSGALT00000015634	
ENSGALT00000019061	WTAP	ENSGALT00000016540	SLC35B2
ENSGALT00000019062		ENSGALT00000015688	GFM1
ENSGALT00000019065	FNDC1	ENSGALT00000016551	SLC29A1
ENSGALT00000031806		ENSGALT00000038145	
ENSGALT00000020066	FBXO30	ENSGALT00000039093	MLF1
ENSGALT00000019393	ZDHHC3	ENSGALT00000016725	TMEM63<br>B
ENSGALT00000020159	STXBP5	ENSGALT00000017557	ISPD
ENSGALT00000020165	SASH1	ENSGALT00000017469	NDC1
ENSGALT00000020182	TAB2	ENSGALT00000017477	
ENSGALT00000020197		ENSGALT00000002207	ATP5F1
ENSGALT00000010523		ENSGALT00000002238	RAP1A
ENSGALT00000010528		ENSGALT00000017661	
ENSGALT00000020225	NUP43	ENSGALT00000017484	
ENSGALT00000019426		ENSGALT00000017487	
ENSGALT00000037591		ENSGALT00000016736	MRPL14
ENSGALT00000031799	LRP11	ENSGALT00000017745	RAPGEF<br>5
ENSGALT00000020231	PPP1R14C	ENSGALT00000017540	ACOT11
ENSGALT00000020285	MTHFD1L	ENSGALT00000002440	PPM1J
ENSGALT00000037581	AKAP12	ENSGALT00000005967	
ENSGALT00000021152		ENSGALT00000016751	
ENSGALT00000019473	TRAK1	ENSGALT00000002661	LRIG2
ENSGALT00000022138	FBXO5	ENSGALT00000017560	
ENSGALT00000019478	EIF1B	ENSGALT00000006011	BNIP2
ENSGALT00000022168		ENSGALT00000016762	
ENSGALT00000022182	SCAF8	ENSGALT00000016793	GTPBP2
ENSGALT00000019495		ENSGALT00000006179	MTMR10
ENSGALT00000022196	TFB1M	ENSGALT00000017568	USP24
ENSGALT00000022259	TMEM242	ENSGALT00000017589	
ENSGALT00000022265	ZDHHC14	ENSGALT00000016730	
ENSGALT00000022291	SNX9	ENSGALT00000016835	
ENSGALT00000010812	ABHD2	ENSGALT00000016761	SLC33A1
ENSGALT00000019569		ENSGALT00000017659	OMA1
ENSGALT00000022311	SYNJ2	ENSGALT00000017826	

ENSGALT00000031788	DYNLT1	ENSGALT00000017301	
ENSGALT00000010834		ENSGALT00000003002	HIPK1
ENSGALT00000019676	ARPP21	ENSGALT00000003025	
ENSGALT00000034015	HAPLN3	ENSGALT00000006206	MPHOSP H10
ENSGALT00000022346	PLAGL1	ENSGALT00000017683	FGGY
ENSGALT00000022358	LTV1	ENSGALT00000017829	MALSU1
ENSGALT00000010909	DET1	ENSGALT00000016815	
ENSGALT00000022362	PHACTR2	ENSGALT00000003070	TRIM33
ENSGALT00000019702	MLH1	ENSGALT00000017877	
ENSGALT00000022374	PEX3	ENSGALT00000017882	CCDC12 6
ENSGALT00000031785	ADAT2	ENSGALT00000034521	MCEE
ENSGALT00000022379	AIG1	ENSGALT00000017975	
ENSGALT00000022399	HIVEP2	ENSGALT00000006347	TARSL2
ENSGALT00000022415	VTA1	ENSGALT00000006419	TM2D3
ENSGALT00000019716	EPDR1	ENSGALT00000017979	
ENSGALT00000022435	HECA	ENSGALT00000032964	TM2D1
ENSGALT00000011024	AGBL1	ENSGALT00000018006	
ENSGALT00000022483	TNFAIP3	ENSGALT00000016864	
ENSGALT00000031776		ENSGALT00000037820	
ENSGALT00000011148		ENSGALT00000017894	ATG4C
ENSGALT00000011156	FAM174B	ENSGALT00000018013	
ENSGALT00000011260	CHD2	ENSGALT00000018034	
ENSGALT00000022522	MAP3K5	ENSGALT00000018085	
ENSGALT00000019783	KIAA0895	ENSGALT00000017909	EFCAB7
ENSGALT00000019789	EEPD1	ENSGALT00000018131	
ENSGALT00000019828		ENSGALT00000016884	
ENSGALT00000019843	HERPUD2	ENSGALT00000018137	
ENSGALT00000022595	PDE7B	ENSGALT00000017947	
ENSGALT00000022607	AHI1	ENSGALT00000018161	CREB5
ENSGALT00000019858		ENSGALT00000018199	CHN2
ENSGALT00000022653	SLC2A12	ENSGALT00000006663	SLTM
ENSGALT00000022662	EYA4	ENSGALT00000017953	
ENSGALT00000019894		ENSGALT00000017959	CACHD1
ENSGALT00000022671		ENSGALT00000016916	TJAP1
ENSGALT00000019908		ENSGALT00000017964	RAVER2
ENSGALT00000037476		ENSGALT00000003247	
ENSGALT00000004596		ENSGALT00000018212	WIPF3
ENSGALT00000004590		ENSGALT00000018231	FKBP14
ENSGALT00000031116	RP9	ENSGALT00000018238	PLEKHA8
ENSGALT00000019924	KBTBD2	ENSGALT00000018282	

ENSGALT00000011432		ENSGALT00000034417	
ENSGALT00000019935	LSM5	ENSGALT00000018291	
ENSGALT00000011484	LYSMD4	ENSGALT00000018312	ANKRD28
ENSGALT00000022702	ARHGAP18	ENSGALT00000018324	GALNT15
ENSGALT00000022704	LAMA2	ENSGALT00000018327	DPH3
ENSGALT00000023965	TRDN	ENSGALT00000016977	ZNF318
ENSGALT00000023984	SMPDL3A	ENSGALT00000016982	
ENSGALT00000023989		ENSGALT00000018329	
ENSGALT00000020113	YAE1D1	ENSGALT00000017012	LTBP3
ENSGALT00000011601		ENSGALT00000017973	AK4
ENSGALT00000037441	MPLKIP	ENSGALT00000018331	
ENSGALT00000011612		ENSGALT00000017995	
ENSGALT00000020152	C7orf25	ENSGALT00000040028	
ENSGALT00000031107		ENSGALT00000040026	
ENSGALT00000011615	PCSK6	ENSGALT00000018348	PLCL2
ENSGALT00000011730		ENSGALT00000018357	TBC1D5
ENSGALT00000024023	CEP85L	ENSGALT00000022526	MNS1
ENSGALT00000020178	BLVRA	ENSGALT00000004037	TSPAN2
ENSGALT00000024033	NUS1	ENSGALT00000018402	KAT2B
ENSGALT00000031104	VOPP1	ENSGALT00000001028	RFX7
ENSGALT00000020187	LANCL2	ENSGALT00000016934	
ENSGALT00000037413		ENSGALT00000018125	SLC35D1
ENSGALT00000024042	DCBLD1	ENSGALT00000017165	
ENSGALT00000020200	TPK1	ENSGALT00000018407	ZNF385D
ENSGALT00000024059		ENSGALT00000018414	UBE2E1
ENSGALT00000011754		ENSGALT00000007043	PIGB
ENSGALT00000020338		ENSGALT00000007059	
ENSGALT00000020347		ENSGALT00000018418	NKIRAS1
ENSGALT00000020352		ENSGALT00000037758	RPL15
ENSGALT00000024093	KPNA5	ENSGALT00000016952	WWTR1
ENSGALT00000031095		ENSGALT00000007264	
ENSGALT00000024128		ENSGALT00000017201	HEATR5B
ENSGALT00000024130	DSE	ENSGALT00000017218	STRN
ENSGALT00000024132	NT5DC1	ENSGALT00000039081	
ENSGALT00000020442	BRD9	ENSGALT00000018328	
ENSGALT00000020444	TPPP	ENSGALT00000018364	
ENSGALT00000020449	LRRC14B	ENSGALT00000016985	TM4SF18
ENSGALT00000020597	COL15A1	ENSGALT00000018434	
ENSGALT00000024248	KIAA1919	ENSGALT00000037966	
ENSGALT00000024256	SLC16A10	ENSGALT00000018443	

ENSGALT00000024267		ENSGALT00000018455	ANKRD13C
ENSGALT00000020598		ENSGALT00000018492	CTH
ENSGALT00000011900	CILP	ENSGALT00000016995	HPS3
ENSGALT00000020622	ADNP2	ENSGALT00000017268	PRKD3
ENSGALT00000020625		ENSGALT00000017274	QPCT
ENSGALT00000020631		ENSGALT00000017275	
ENSGALT00000011910	PARP16	ENSGALT00000004361	UHRF1BP1
ENSGALT00000028084		ENSGALT00000017280	
ENSGALT00000020646		ENSGALT00000018563	SLC44A5
ENSGALT00000012036	DPP8	ENSGALT00000017288	
ENSGALT00000020648	NFATC1	ENSGALT00000018575	RABGGTB
ENSGALT00000024590	CD164	ENSGALT00000018582	MSH4
ENSGALT00000024673	SESN1	ENSGALT00000018456	OXSM
ENSGALT00000020670	ALDH5A1	ENSGALT00000018458	LRRC3B
ENSGALT00000020676	MRS2	ENSGALT00000004378	
ENSGALT00000020679	NRSN1	ENSGALT00000017328	TFB2M
ENSGALT00000020688	CDKAL1	ENSGALT00000004387	
ENSGALT00000024687	FOXO3	ENSGALT00000037849	
ENSGALT00000020693	RNF144B	ENSGALT00000018603	SLC4A7
ENSGALT00000024698	LACE1	ENSGALT00000018638	AZI2
ENSGALT00000024702		ENSGALT00000007643	TNFAIP8L3
ENSGALT00000012111		ENSGALT00000018667	GADL1
ENSGALT00000024713		ENSGALT00000017370	
ENSGALT00000024718	BEND3	ENSGALT00000017414	ZBTB18
ENSGALT00000024723		ENSGALT00000018707	OSBPL10
ENSGALT00000024761	RTN4IP1	ENSGALT00000023354	
ENSGALT00000012122	DENND4A	ENSGALT00000018718	GPD1L
ENSGALT00000024849		ENSGALT00000000338	HEATR1
ENSGALT00000020720		ENSGALT00000004468	
ENSGALT00000037256		ENSGALT00000017618	ERO1LB
ENSGALT00000024935		ENSGALT00000018721	
ENSGALT00000024970		ENSGALT00000017890	NID1
ENSGALT00000020725	RANBP9	ENSGALT00000018725	
ENSGALT00000012312		ENSGALT00000018727	CMTM6
ENSGALT00000024980	FBXL4	ENSGALT00000035045	
ENSGALT00000012379		ENSGALT00000004543	
ENSGALT00000012462		ENSGALT00000018736	TCAIM
ENSGALT00000031683		ENSGALT00000022610	B3GALN

			T2
ENSGALT00000025147	MAP3K7	ENSGALT00000004102	GGPS1
ENSGALT00000020740		ENSGALT00000000385	ARID4B
ENSGALT00000020742		ENSGALT00000018770	ANO10
ENSGALT00000020743		ENSGALT00000022567	TOMM20
ENSGALT00000020748	ATXN1	ENSGALT00000018780	
ENSGALT00000020760	CAP2	ENSGALT00000018812	EAF1
ENSGALT00000020766	NUP153	ENSGALT00000017944	
ENSGALT00000025160	BACH2	ENSGALT00000018839	
ENSGALT00000025170		ENSGALT00000018846	CAPN7
ENSGALT00000025373	MDN1	ENSGALT00000007944	
ENSGALT00000020785	EDN1	ENSGALT00000018026	
ENSGALT00000020801	NEDD9	ENSGALT00000018061	CCSAP
ENSGALT00000025442		ENSGALT00000009049	CEP152
ENSGALT00000020851		ENSGALT00000018064	
ENSGALT00000020852		ENSGALT00000018070	NUP133
ENSGALT00000020859		ENSGALT00000005088	TSPO2
ENSGALT00000020862	BMP6	ENSGALT00000034280	SECISBP2L
ENSGALT00000025447		ENSGALT00000018081	TAF5L
ENSGALT00000025457	RNGTT	ENSGALT00000018087	URB2
ENSGALT00000025464		ENSGALT00000005108	APOBEC2
ENSGALT00000025485		ENSGALT00000018092	GALNT2
ENSGALT00000025493		ENSGALT00000005134	
ENSGALT00000025521		ENSGALT00000018903	ATP2C1
ENSGALT00000031045		ENSGALT00000039833	
ENSGALT00000020881		ENSGALT00000039832	
ENSGALT00000037148		ENSGALT00000031841	C1orf131
ENSGALT00000020885		ENSGALT00000018198	GNPAT
ENSGALT00000037229		ENSGALT00000018210	
ENSGALT00000020890	FARS2	ENSGALT00000018940	
ENSGALT00000020897	ECI2	ENSGALT00000018217	
ENSGALT00000025551	PRSS35	ENSGALT00000018228	FAM120B
ENSGALT00000025559	PGM3	ENSGALT00000018250	PHF10
ENSGALT00000012808		ENSGALT00000018251	
ENSGALT00000020905	PXDC1	ENSGALT00000019087	DNAJC13
ENSGALT00000020909	SLC22A23	ENSGALT00000018269	SMOC2
ENSGALT00000025575	IBTK	ENSGALT00000005461	FRS3
ENSGALT00000020914		ENSGALT00000009208	
ENSGALT00000020918	BPHL	ENSGALT00000009244	
ENSGALT00000025610	PHIP	ENSGALT00000018417	DACT2

ENSGALT00000020938		ENSGALT00000005500	
ENSGALT00000020945	GMDS	ENSGALT00000005492	BYSL
ENSGALT00000020947	MYLK4	ENSGALT00000009484	USP8
ENSGALT00000020983		ENSGALT00000018633	FGFR1OP
ENSGALT00000025628	FILIP1	ENSGALT00000037674	
ENSGALT00000012883		ENSGALT00000018720	
ENSGALT00000025629		ENSGALT00000018857	PARK2
ENSGALT00000021025	TNFRSF11A	ENSGALT00000018893	MAP3K4
ENSGALT00000025651		ENSGALT00000005533	
ENSGALT00000025653		ENSGALT00000019134	
ENSGALT00000025678	KCNQ5	ENSGALT00000038740	
ENSGALT00000025705	ASRGL1	ENSGALT00000037615	CDV3
ENSGALT00000012959		ENSGALT00000009646	FSD2
ENSGALT00000021128	MYO10	ENSGALT00000009658	WHAMM
ENSGALT00000021151	FAM105B	ENSGALT00000009670	
ENSGALT00000021178	TRIO	ENSGALT00000037604	
ENSGALT00000021258	MARCH6	ENSGALT00000009692	
ENSGALT00000021280	SBK2	ENSGALT00000019273	LZTFL1
ENSGALT00000021302	SEMA5A	ENSGALT00000009702	
ENSGALT00000026230	PHF3	ENSGALT00000018986	
ENSGALT00000026237		ENSGALT00000019010	
ENSGALT00000021306	MTRR	ENSGALT00000005608	
ENSGALT00000026245		ENSGALT00000005617	PRELP
ENSGALT00000026251	ZNF451	ENSGALT00000019329	SACM1L
ENSGALT00000026264	DST	ENSGALT00000010337	
ENSGALT00000013227	UACA	ENSGALT00000019376	LARS2
ENSGALT00000021443		ENSGALT00000019061	WTAP
ENSGALT00000021447		ENSGALT00000019062	
ENSGALT00000021448	TMEM245	ENSGALT00000019065	FNDC1
ENSGALT00000026295		ENSGALT00000031806	
ENSGALT00000026298	MLIP	ENSGALT00000020066	FBXO30
ENSGALT00000026310	KLHL31	ENSGALT00000019393	ZDHHC3
ENSGALT00000021488		ENSGALT00000020159	STXBP5
ENSGALT00000026320	GCLC	ENSGALT00000020165	SASH1
ENSGALT00000021491		ENSGALT00000020182	TAB2
ENSGALT00000026321		ENSGALT00000020197	
ENSGALT00000021505		ENSGALT00000010528	
ENSGALT00000026329		ENSGALT00000020225	NUP43
ENSGALT00000026331	ICK	ENSGALT00000037591	
ENSGALT00000021546		ENSGALT00000031799	LRP11
ENSGALT00000021552	NDUFS6	ENSGALT00000020231	PPP1R14

			C
ENSGALT00000026340	AGPAT5	ENSGALT00000020285	MTHFD1L
ENSGALT00000026360		ENSGALT00000037581	AKAP12
ENSGALT00000021557	ANKS6	ENSGALT00000019473	TRAK1
ENSGALT00000021568	ELP2	ENSGALT00000022138	FBXO5
ENSGALT00000026373	ARHGEF10	ENSGALT00000019478	EIF1B
ENSGALT00000031625		ENSGALT00000022168	
ENSGALT00000026385		ENSGALT00000022182	SCAF8
ENSGALT00000026390	FBXO25	ENSGALT00000019495	
ENSGALT00000021578	SLC39A6	ENSGALT00000022196	TFB1M
ENSGALT00000026395		ENSGALT00000022259	TMEM242
ENSGALT00000013352		ENSGALT00000022265	ZDHHC14
ENSGALT00000013365		ENSGALT00000022291	SNX9
ENSGALT00000026413	PXDN	ENSGALT00000010812	ABHD2
ENSGALT00000021854	PDCD6	ENSGALT00000019569	
ENSGALT00000021913		ENSGALT00000022311	SYNJ2
ENSGALT00000021930	ERP44	ENSGALT00000022325	TMEM181
ENSGALT00000015891		ENSGALT00000031788	DYNLT1
ENSGALT00000021943		ENSGALT00000010834	
ENSGALT00000022087	NR4A3	ENSGALT00000019676	ARPP21
ENSGALT00000022096		ENSGALT00000034015	HAPLN3
ENSGALT00000022115	BLOC1S4	ENSGALT00000022346	PLAGL1
ENSGALT00000022120	PARD6G	ENSGALT00000022358	LTV1
ENSGALT00000022165		ENSGALT00000010909	DET1
ENSGALT00000026493	CPSF3	ENSGALT00000022362	PHACTR2
ENSGALT00000015890		ENSGALT00000022368	FUCA2
ENSGALT00000022178	ACOT13	ENSGALT00000019702	MLH1
ENSGALT00000026523		ENSGALT00000022374	PEX3
ENSGALT00000026524		ENSGALT00000031785	ADAT2
ENSGALT00000036936	ZADH2	ENSGALT00000022379	AIG1
ENSGALT00000013464	TMED3	ENSGALT00000022415	VTA1
ENSGALT00000022283	FAM69C	ENSGALT00000019716	EPDR1
ENSGALT00000021988	PPP6R2	ENSGALT00000022435	HECA
ENSGALT00000020337	SBF1	ENSGALT00000011024	AGBL1
ENSGALT00000022290	TIMM21	ENSGALT00000022483	TNFAIP3
ENSGALT00000040414		ENSGALT00000031776	
ENSGALT00000025383		ENSGALT00000011148	

ENSGALT00000026538	PDIA6	ENSGALT00000022499	
ENSGALT00000022432	TNPO3	ENSGALT00000011156	FAM174B
ENSGALT00000012929	AHCYL2	ENSGALT00000011260	CHD2
ENSGALT00000012955		ENSGALT00000022522	MAP3K5
ENSGALT00000013005	KLHDC10	ENSGALT00000038512	FAM169B
ENSGALT00000022519	RNMT	ENSGALT00000019843	HERPUD2
ENSGALT00000022497	SEH1L	ENSGALT00000022595	PDE7B
ENSGALT00000022508	CEP192	ENSGALT00000022607	AHI1
ENSGALT00000013037	TMEM209	ENSGALT00000019858	
ENSGALT00000026552		ENSGALT00000022653	SLC2A12
ENSGALT00000013516		ENSGALT00000019894	
ENSGALT00000022485	PSMG2	ENSGALT00000022671	
ENSGALT00000013533	UNC45A	ENSGALT00000019908	
ENSGALT00000013568	MAN2A2	ENSGALT00000037476	
ENSGALT00000026569	SMC6	ENSGALT00000004596	
ENSGALT00000022445		ENSGALT00000004590	
ENSGALT00000022420	IMPA2	ENSGALT00000031116	RP9
ENSGALT00000031591	RDH14	ENSGALT00000004401	MED23
ENSGALT00000026575		ENSGALT00000019924	KBTBD2
ENSGALT00000013284	FAM208B	ENSGALT00000011432	
ENSGALT00000026589	SDC1	ENSGALT00000019935	LSM5
ENSGALT00000022579	NAPG	ENSGALT00000011484	LYSMD4
ENSGALT00000028442		ENSGALT00000022702	ARHGAP18
ENSGALT00000033559	RAB31	ENSGALT00000022704	LAMA2
ENSGALT00000013703	PPP4R1	ENSGALT00000023937	ECHDC1
ENSGALT00000013581		ENSGALT00000020038	LRRFIP2
ENSGALT00000013684		ENSGALT00000023965	TRDN
ENSGALT00000013683	ANKRD12	ENSGALT00000023984	SMPDL3A
ENSGALT00000026614		ENSGALT00000023989	
ENSGALT00000023134	NDUFV2	ENSGALT00000020113	YAE1D1
ENSGALT00000031579	C2orf44	ENSGALT00000011601	
ENSGALT00000026617		ENSGALT00000037441	MPLKIP
ENSGALT00000013589		ENSGALT00000011612	
ENSGALT00000013794	SLC35B4	ENSGALT00000020152	C7orf25
ENSGALT00000013813		ENSGALT00000031107	
ENSGALT00000010489	CHCHD3	ENSGALT00000011615	PCSK6
ENSGALT00000013724	MAP1A	ENSGALT00000011730	
ENSGALT00000040160		ENSGALT00000020177	
ENSGALT00000014003	IQGAP1	ENSGALT00000024023	CEP85L

ENSGALT00000010225	PFKFB3	ENSGALT00000020178	BLVRA
ENSGALT00000040107	PRKCQ	ENSGALT00000024033	NUS1
ENSGALT00000011162	ITIH5	ENSGALT00000020187	LANCL2
ENSGALT00000010913		ENSGALT00000037413	
ENSGALT00000023972		ENSGALT00000024036	GOPC
ENSGALT00000036879		ENSGALT00000024042	DCBLD1
ENSGALT00000026630	ZNF512	ENSGALT00000020200	TPK1
ENSGALT00000026632	FNDC4	ENSGALT00000024059	
ENSGALT00000010889		ENSGALT00000020295	
ENSGALT00000010831	PROSER2	ENSGALT00000011754	
ENSGALT00000010817	UPF2	ENSGALT00000020338	
ENSGALT00000005822	DHTKD1	ENSGALT00000020352	
ENSGALT00000022642	SEC61A2	ENSGALT00000024093	KPNA5
ENSGALT00000026641		ENSGALT00000031095	
ENSGALT00000022732	CDC123	ENSGALT00000035373	
ENSGALT00000026651	SNX17	ENSGALT00000031721	
ENSGALT00000016041	CAMK1D	ENSGALT00000024128	
ENSGALT00000022327		ENSGALT00000024130	DSE
ENSGALT00000023489	PHYH	ENSGALT00000024132	NT5DC1
ENSGALT00000006362		ENSGALT00000020442	BRD9
ENSGALT00000023502		ENSGALT00000020444	TPPP
ENSGALT00000010765	FRMD4A	ENSGALT00000020449	LRRC14B
ENSGALT00000023921	EMILIN2	ENSGALT00000020597	COL15A1
ENSGALT00000029187		ENSGALT00000024248	KIAA1919
ENSGALT00000036791		ENSGALT00000024256	SLC16A10
ENSGALT00000030754		ENSGALT00000024267	
ENSGALT00000026684		ENSGALT00000020598	
ENSGALT00000010596		ENSGALT00000011900	CILP
ENSGALT00000026714	HADHB	ENSGALT00000020610	RECK
ENSGALT00000026723		ENSGALT00000020622	ADNP2
ENSGALT00000013777		ENSGALT00000020625	
ENSGALT00000039749		ENSGALT00000020631	
ENSGALT00000023980		ENSGALT00000024288	DDO
ENSGALT00000026748		ENSGALT00000011910	PARP16
ENSGALT00000026763	SCARA5	ENSGALT00000028084	
ENSGALT00000013557		ENSGALT00000020646	
ENSGALT00000013555	RSBN1L	ENSGALT00000012036	DPP8
ENSGALT00000013546	PTPN12	ENSGALT00000020648	NFATC1
ENSGALT00000026776	SLC35F6	ENSGALT00000024590	CD164
ENSGALT00000013530	GSAP	ENSGALT00000020670	ALDH5A1
ENSGALT00000013515	FAM185A	ENSGALT00000020676	MRS2

ENSGALT00000013482		ENSGALT00000020679	NRSN1
ENSGALT00000013473		ENSGALT00000020688	CDKAL1
ENSGALT00000013428		ENSGALT00000024687	FOXO3
ENSGALT00000013382		ENSGALT00000020695	
ENSGALT00000024064	USP14	ENSGALT00000024698	LACE1
ENSGALT00000013290		ENSGALT00000024702	
ENSGALT00000013259	KMT2E	ENSGALT00000012111	
ENSGALT00000026827	ZNF395	ENSGALT00000024713	
ENSGALT00000013245	SRPK2	ENSGALT00000024718	BEND3
ENSGALT00000013186		ENSGALT00000024723	
ENSGALT00000013172	ATXN7L1	ENSGALT00000024823	
ENSGALT00000026845	EXTL3	ENSGALT00000024849	
ENSGALT00000026848		ENSGALT00000020720	
ENSGALT00000013116		ENSGALT00000037256	
ENSGALT00000013062		ENSGALT00000024935	
ENSGALT00000026872	XKR6	ENSGALT00000024942	TSTD1
ENSGALT00000039460	DUS4L	ENSGALT00000024970	
ENSGALT00000026875		ENSGALT00000024973	COQ3
ENSGALT00000026878		ENSGALT00000020725	RANBP9
ENSGALT00000013000		ENSGALT00000012312	
ENSGALT00000024152	MIB1	ENSGALT00000024980	FBXL4
ENSGALT00000026894		ENSGALT00000012364	DIS3L
ENSGALT00000031549		ENSGALT00000031690	NDUFAF4
ENSGALT00000024169	RBBP8	ENSGALT00000025106	
ENSGALT00000012832	LAMB1	ENSGALT00000012379	
ENSGALT00000026905	TRAM2	ENSGALT00000012462	
ENSGALT00000024197	RIOK3	ENSGALT00000020734	MCUR1
ENSGALT00000011632		ENSGALT00000031683	
ENSGALT00000024206	C18orf8	ENSGALT00000025147	MAP3K7
ENSGALT00000011493	KIF21A	ENSGALT00000020740	
ENSGALT00000026946	MUT	ENSGALT00000012470	
ENSGALT00000024240	NPC1	ENSGALT00000020743	
ENSGALT00000026953		ENSGALT00000020748	ATXN1
ENSGALT00000031528	CLIC5	ENSGALT00000020752	
ENSGALT00000026965	RCAN2	ENSGALT00000020760	CAP2
ENSGALT00000026967	CYP39A1	ENSGALT00000020766	NUP153
ENSGALT00000013914		ENSGALT00000025170	
ENSGALT00000013929		ENSGALT00000025373	MDN1
ENSGALT00000026980	GPR116	ENSGALT00000020779	TBC1D7
ENSGALT00000039200	PIM3	ENSGALT00000025418	
ENSGALT00000014167		ENSGALT00000012541	ZWILCH

ENSGALT00000014151		ENSGALT00000020785	EDN1
ENSGALT00000024335	OSBPL1A	ENSGALT00000020801	NEDD9
ENSGALT00000021457	PLXNB2	ENSGALT00000025442	
ENSGALT00000024382	ZNF521	ENSGALT00000020851	
ENSGALT00000024407	KCTD1	ENSGALT00000020852	
ENSGALT00000014328	ASB15	ENSGALT00000020859	
ENSGALT00000014337		ENSGALT00000020862	BMP6
ENSGALT00000014340	IQUB	ENSGALT00000025447	
ENSGALT00000014357		ENSGALT00000025457	RNGTT
ENSGALT00000014559	AASS	ENSGALT00000037170	
ENSGALT00000014601		ENSGALT00000025464	
ENSGALT00000014754		ENSGALT00000025485	
ENSGALT00000014827		ENSGALT00000025521	
ENSGALT00000014836		ENSGALT00000020881	
ENSGALT00000015281		ENSGALT00000025528	NT5E
ENSGALT00000015293		ENSGALT00000037148	
ENSGALT00000024463	TRAPPC8	ENSGALT00000020885	
ENSGALT00000015373	TMEM168	ENSGALT00000037229	
ENSGALT00000030698	LSMEM1	ENSGALT00000020890	FARS2
ENSGALT00000015379		ENSGALT00000020897	ECI2
ENSGALT00000015454		ENSGALT00000025551	PRSS35
ENSGALT00000015471		ENSGALT00000025559	PGM3
ENSGALT00000015532		ENSGALT00000012808	
ENSGALT00000015558	PRICKLE1	ENSGALT00000020904	PRPF4B
ENSGALT00000015593	ADAMTS20	ENSGALT00000020905	PXDC1
ENSGALT00000015605	PUS7L	ENSGALT00000025575	IBTK
ENSGALT00000015635		ENSGALT00000020914	
ENSGALT00000015636	TMEM117	ENSGALT00000020918	BPHL
ENSGALT00000015694	ANO6	ENSGALT00000025610	PHIP
ENSGALT00000015735	ARID2	ENSGALT00000020938	
ENSGALT00000015787		ENSGALT00000020945	GMDS
ENSGALT00000015837		ENSGALT00000020947	MYLK4
ENSGALT00000024545	DTNA	ENSGALT00000020983	
ENSGALT00000015861		ENSGALT00000012856	C15orf61
ENSGALT00000015878		ENSGALT00000025628	FILIP1
ENSGALT00000015976		ENSGALT00000025629	
ENSGALT00000015981	RPL18A	ENSGALT00000031654	
ENSGALT00000016025		ENSGALT00000021007	
ENSGALT00000038638		ENSGALT00000021025	TNFRSF11A
ENSGALT00000016059		ENSGALT00000021033	KIAA1468
ENSGALT00000016099	CAND1	ENSGALT00000012956	

ENSGALT00000016139		ENSGALT00000025651	
ENSGALT00000016160		ENSGALT00000025653	
ENSGALT00000016192		ENSGALT00000025678	KCNQ5
ENSGALT00000021064	MCM4	ENSGALT00000025705	ASRGL1
ENSGALT00000016330	PTPRB	ENSGALT00000012959	
ENSGALT00000041400	C8orf22	ENSGALT00000021151	FAM105B
ENSGALT00000016545	ZFC3H1	ENSGALT00000021178	TRIO
ENSGALT00000016553	RAB21	ENSGALT00000013051	CORO2B
ENSGALT00000024628	RB1CC1	ENSGALT00000021258	MARCH6
ENSGALT00000022162		ENSGALT00000021277	
ENSGALT00000016625		ENSGALT00000021280	SBK2
ENSGALT00000016660	OSBPL8	ENSGALT00000021302	SEMA5A
ENSGALT00000017803	LIN7A	ENSGALT00000026230	PHF3
ENSGALT00000024757		ENSGALT00000026237	
ENSGALT00000029932		ENSGALT00000021306	MTRR
ENSGALT00000024867		ENSGALT00000021307	FASTKD3
ENSGALT00000024871	PENK	ENSGALT00000026245	
ENSGALT00000024873	IMPAD1	ENSGALT00000026251	ZNF451
ENSGALT00000024899		ENSGALT00000026253	BEND6
ENSGALT00000024908	NSMAF	ENSGALT00000026264	DST
ENSGALT00000018221	CEP290	ENSGALT00000013227	UACA
ENSGALT00000018392		ENSGALT00000033617	
ENSGALT00000018400		ENSGALT00000021443	
ENSGALT00000018404	EEA1	ENSGALT00000021448	TMEM24<br>5
ENSGALT00000018413		ENSGALT00000026298	MLIP
ENSGALT00000030642		ENSGALT00000026310	KLHL31
ENSGALT00000024965	ASPH	ENSGALT00000021488	
ENSGALT00000024998	GGH	ENSGALT00000026320	GCLC
ENSGALT00000030640	CCDC41	ENSGALT00000021491	
ENSGALT00000018472	TMCC3	ENSGALT00000026321	
ENSGALT00000018479		ENSGALT00000021505	
ENSGALT00000018529	VEZT	ENSGALT00000026329	
ENSGALT00000025000		ENSGALT00000026331	ICK
ENSGALT00000025023		ENSGALT00000021546	
ENSGALT00000022941	ADHFE1	ENSGALT00000021552	NDUFS6
ENSGALT00000018595	USP44	ENSGALT00000026340	AGPAT5
ENSGALT00000018639		ENSGALT00000026360	
ENSGALT00000025029		ENSGALT00000021557	ANKS6
ENSGALT00000018679	CDK17	ENSGALT00000021558	GALNT12
ENSGALT00000018716		ENSGALT00000021568	ELP2
ENSGALT00000023424		ENSGALT00000031625	

ENSGALT00000025048	CSPP1	ENSGALT00000026385	
ENSGALT00000025075	ARFGEF1	ENSGALT00000026390	FBXO25
ENSGALT00000018870		ENSGALT00000021578	SLC39A6
ENSGALT00000025131	SLCO5A1	ENSGALT00000026395	
ENSGALT00000025181		ENSGALT00000013336	
ENSGALT00000030884	MSC	ENSGALT00000013352	
ENSGALT00000019040		ENSGALT00000013365	
ENSGALT00000019063	NUP205	ENSGALT00000026413	PXDN
ENSGALT00000025372		ENSGALT00000021854	PDCD6
ENSGALT00000025374	ZC2HC1A	ENSGALT00000021913	
ENSGALT00000025468		ENSGALT00000021930	ERP44
ENSGALT00000025499	LRRCC1	ENSGALT00000015891	
ENSGALT00000025504	CA13	ENSGALT00000021943	
ENSGALT00000019173		ENSGALT00000022087	NR4A3
ENSGALT00000019245		ENSGALT00000022115	BLOC1S4
ENSGALT00000019256		ENSGALT00000022120	PARD6G
ENSGALT00000030620		ENSGALT00000022135	
ENSGALT00000025563		ENSGALT00000022165	
ENSGALT00000025572		ENSGALT00000022169	
ENSGALT00000019301	PLBD1	ENSGALT00000026493	CPSF3
ENSGALT00000019343	C7orf55	ENSGALT00000015890	
ENSGALT00000030617		ENSGALT00000022178	ACOT13
ENSGALT00000019356		ENSGALT00000015860	ARSA
ENSGALT00000019412		ENSGALT00000026523	
ENSGALT00000019419	FAM109B	ENSGALT00000026524	
ENSGALT00000019442	SREBF2	ENSGALT00000026525	RRM2
ENSGALT00000019444	CCDC134	ENSGALT00000036936	ZADH2
ENSGALT00000025647	DECR1	ENSGALT00000013464	TMED3
ENSGALT00000019461	NHP2L1	ENSGALT00000022283	FAM69C
ENSGALT00000019464	DESI1	ENSGALT00000021988	PPP6R2
ENSGALT00000019482	PMM1	ENSGALT00000020337	SBF1
ENSGALT00000019494		ENSGALT00000022290	TIMM21
ENSGALT00000019497		ENSGALT00000040414	
ENSGALT00000019502		ENSGALT00000026538	PDIA6
ENSGALT00000037963		ENSGALT00000022432	TNPO3
ENSGALT00000019514	ZC3H7B	ENSGALT00000012929	AHCYL2
ENSGALT00000025676		ENSGALT00000012992	
ENSGALT00000025692		ENSGALT00000013005	KLHDC10
ENSGALT00000025711		ENSGALT00000022519	RNMT
ENSGALT00000019565		ENSGALT00000022497	SEH1L
ENSGALT00000019589	XPNPEP3	ENSGALT00000022508	CEP192
ENSGALT00000019595		ENSGALT00000013037	TMEM20

			9
ENSGALT00000025714	RNF151	ENSGALT00000026552	
ENSGALT00000025742	DPY19L4	ENSGALT00000013516	
ENSGALT00000030837	NDUFAF6	ENSGALT00000022485	PSMG2
ENSGALT00000025765		ENSGALT00000013533	UNC45A
ENSGALT00000025772		ENSGALT00000013568	MAN2A2
ENSGALT00000025777		ENSGALT00000026569	SMC6
ENSGALT00000036623	MTDH	ENSGALT00000022445	
ENSGALT00000023439	MATN2	ENSGALT00000013228	COPG2
ENSGALT00000037892		ENSGALT00000022420	IMPA2
ENSGALT00000019837	MIEF1	ENSGALT00000031591	RDH14
ENSGALT00000019854		ENSGALT00000026575	
ENSGALT00000029404	HRSP12	ENSGALT00000026577	
ENSGALT00000025801		ENSGALT00000013284	FAM208B
ENSGALT00000030604		ENSGALT00000026582	TTC32
ENSGALT00000019957	JOSD1	ENSGALT00000026589	SDC1
ENSGALT00000030826		ENSGALT00000022385	
ENSGALT00000030825	POLR2K	ENSGALT00000022579	NAPG
ENSGALT00000025842	RNF19A	ENSGALT00000028442	
ENSGALT00000028271	ANKRD46	ENSGALT00000033559	RAB31
ENSGALT00000023333		ENSGALT00000013703	PPP4R1
ENSGALT00000020049		ENSGALT00000013581	
ENSGALT00000025855	RRM2B	ENSGALT00000013684	
ENSGALT00000030592	PICK1	ENSGALT00000013683	ANKRD1<br>2
ENSGALT00000025850	UBR5	ENSGALT00000026614	
ENSGALT00000020100	ANKRD54	ENSGALT00000023134	NDUFV2
ENSGALT00000020120		ENSGALT00000031579	C2orf44
ENSGALT00000020453		ENSGALT00000026617	
ENSGALT00000025898		ENSGALT00000013589	
ENSGALT00000020484		ENSGALT00000013664	PPIP5K1
ENSGALT00000037765		ENSGALT00000013794	SLC35B4
ENSGALT00000020498		ENSGALT00000013813	
ENSGALT00000020504	HMGXB4	ENSGALT00000010489	CHCHD3
ENSGALT00000025906	LRP12	ENSGALT00000013724	MAP1A
ENSGALT00000025930		ENSGALT00000040160	
ENSGALT00000025937		ENSGALT00000014003	IQGAP1
ENSGALT00000020590	RTCB	ENSGALT00000010225	PFKFB3
ENSGALT00000020600	PRDM4	ENSGALT00000040107	PRKCQ
ENSGALT00000030816		ENSGALT00000011162	ITIH5
ENSGALT00000025971		ENSGALT00000010913	
ENSGALT00000025969	UTP23	ENSGALT00000023972	

ENSGALT00000025965		ENSGALT00000023964	
ENSGALT00000020629		ENSGALT00000036879	
ENSGALT00000020633	RIC8B	ENSGALT00000026630	ZNF512
ENSGALT00000026501		ENSGALT00000026632	FNDC4
ENSGALT00000026492	TAF2	ENSGALT00000010889	
ENSGALT00000020668	NUAK1	ENSGALT00000010831	PROSER 2
ENSGALT00000026475		ENSGALT00000010817	UPF2
ENSGALT00000020714		ENSGALT00000005822	DHTKD1
ENSGALT00000026431		ENSGALT00000022642	SEC61A2
ENSGALT00000026425	TBC1D31	ENSGALT00000026641	
ENSGALT00000020683		ENSGALT00000022732	CDC123
ENSGALT00000026409	ATAD2	ENSGALT00000026651	SNX17
ENSGALT00000026383	FAM91A1	ENSGALT00000016041	CAMK1D
ENSGALT00000026325	KIAA0196	ENSGALT00000022327	
ENSGALT00000026313	TRIB1	ENSGALT00000023489	PHYH
ENSGALT00000020764	HCFC2	ENSGALT00000006362	
ENSGALT00000020765		ENSGALT00000023502	
ENSGALT00000026309		ENSGALT00000010765	FRMD4A
ENSGALT00000020769		ENSGALT00000023921	EMILIN2
ENSGALT00000030784	FAM49B	ENSGALT00000029187	
ENSGALT00000020773		ENSGALT00000036791	
ENSGALT00000020783	NT5DC3	ENSGALT00000026684	
ENSGALT00000026194		ENSGALT00000010596	
ENSGALT00000020821		ENSGALT00000026714	HADHB
ENSGALT00000020822	CCDC53	ENSGALT00000026723	
ENSGALT00000020826	GNPTAB	ENSGALT00000013777	
ENSGALT00000020835		ENSGALT00000039749	
ENSGALT00000026120	KHDRBS3	ENSGALT00000026740	
ENSGALT00000020864	PARP12	ENSGALT00000023980	
ENSGALT00000020873	HIPK2	ENSGALT00000024028	TYMS
ENSGALT00000026092	AGO2	ENSGALT00000026763	SCARA5
ENSGALT00000020879	KIAA1549	ENSGALT00000013567	
ENSGALT00000026043	PTP4A3	ENSGALT00000026767	PBK
ENSGALT00000026040	TSNARE1	ENSGALT00000013557	
ENSGALT00000026012		ENSGALT00000026773	CCDC25
ENSGALT00000026001	RHPN1	ENSGALT00000013555	RSBN1L
ENSGALT00000020934		ENSGALT00000013546	PTPN12
ENSGALT00000020949		ENSGALT00000026776	SLC35F6
ENSGALT00000020953	KDM7A	ENSGALT00000013530	GSAP
ENSGALT00000020957		ENSGALT00000013515	FAM185A
ENSGALT00000020962		ENSGALT00000013512	

ENSGALT00000020996	AGK	ENSGALT00000013482	
ENSGALT00000000104	NRBP2	ENSGALT00000013473	
ENSGALT00000021056	SYT10	ENSGALT00000013428	
ENSGALT00000021073	YARS2	ENSGALT00000013382	
ENSGALT00000021086		ENSGALT00000024064	USP14
ENSGALT00000021103	AMN1	ENSGALT00000013290	
ENSGALT00000021115		ENSGALT00000013259	KMT2E
ENSGALT00000021125	IPO8	ENSGALT00000026827	ZNF395
ENSGALT00000021134	TMTC1	ENSGALT00000013245	SRPK2
ENSGALT00000030549		ENSGALT00000026845	EXTL3
ENSGALT00000021202	B4GALNT3	ENSGALT00000026848	
ENSGALT00000021213	ERC1	ENSGALT00000013116	
ENSGALT00000021246	DCP1B	ENSGALT00000026852	HMBOX1
ENSGALT00000021270	CECR5	ENSGALT00000013062	
ENSGALT00000021281		ENSGALT00000026872	XKR6
ENSGALT00000021282	BCL2L13	ENSGALT00000039460	DUS4L
ENSGALT00000021284		ENSGALT00000026875	
ENSGALT00000021332		ENSGALT00000026878	
ENSGALT00000021337		ENSGALT00000013000	
ENSGALT00000005490		ENSGALT00000024095	ABHD3
ENSGALT00000021340		ENSGALT00000024152	MIB1
ENSGALT00000021369	EPS8	ENSGALT00000026894	
ENSGALT00000021374		ENSGALT00000031549	
ENSGALT00000021380		ENSGALT00000024169	RBBP8
ENSGALT00000021444	AEBP2	ENSGALT00000012832	LAMB1
ENSGALT00000021521	BCAT1	ENSGALT00000026905	TRAM2
ENSGALT00000021594		ENSGALT00000024186	CABLES1
ENSGALT00000021607	CMAS	ENSGALT00000024197	RIOK3
ENSGALT00000021626	ABCC9	ENSGALT00000011632	
ENSGALT00000022726		ENSGALT00000026909	
ENSGALT00000022736	RASSF8	ENSGALT00000024206	C18orf8
ENSGALT00000022738	SSPN	ENSGALT00000011493	KIF21A
ENSGALT00000022808	TM7SF3	ENSGALT00000026922	
ENSGALT00000022820		ENSGALT00000026946	MUT
ENSGALT00000022856	CYB5R3	ENSGALT00000024240	NPC1
ENSGALT00000022912		ENSGALT00000026953	
ENSGALT00000022959		ENSGALT00000031528	CLIC5
ENSGALT00000022966	PARVB	ENSGALT00000026965	RCAN2
ENSGALT00000022984		ENSGALT00000026967	CYP39A1
ENSGALT00000022998		ENSGALT00000013914	
ENSGALT00000023003	KIAA0930	ENSGALT00000013929	
ENSGALT00000023014		ENSGALT00000039200	PIM3

ENSGALT00000023016	ATXN10	ENSGALT00000027001	
ENSGALT00000015796		ENSGALT00000024304	TTC39C
ENSGALT00000018841	LRP6	ENSGALT00000014167	
ENSGALT00000027945	RAD51AP1	ENSGALT00000014151	
ENSGALT00000027937		ENSGALT00000024335	OSBPL1A
ENSGALT00000030043		ENSGALT00000021457	PLXNB2
ENSGALT00000021680	PRMT8	ENSGALT00000024382	ZNF521
ENSGALT00000023174	TSPAN9	ENSGALT00000024407	KCTD1
ENSGALT00000021894	TULP3	ENSGALT00000014313	
ENSGALT00000023158		ENSGALT00000014328	ASB15
ENSGALT00000023157	NECAP1	ENSGALT00000014337	
ENSGALT00000023154	FOXJ2	ENSGALT00000014340	IQUB
ENSGALT00000023197	MAN1A2	ENSGALT00000024417	
ENSGALT00000023287		ENSGALT00000014357	
ENSGALT00000035021		ENSGALT00000014559	AASS
ENSGALT00000022680	FBXO40	ENSGALT00000014601	
ENSGALT00000023323		ENSGALT00000014754	
ENSGALT00000023360		ENSGALT00000014827	
ENSGALT00000023361		ENSGALT00000015281	
ENSGALT00000023441	USP5	ENSGALT00000015293	
ENSGALT00000023442		ENSGALT00000024463	TRAPPC8
ENSGALT00000023485		ENSGALT00000015373	TMEM168
ENSGALT00000023492		ENSGALT00000030698	LSMEM1
ENSGALT00000023493		ENSGALT00000015454	
ENSGALT00000023496	LPCAT3	ENSGALT00000015456	
ENSGALT00000023646	CLSTN3	ENSGALT00000015471	
ENSGALT00000023686	CLCN1	ENSGALT00000015526	
ENSGALT00000023710		ENSGALT00000024497	GAREM
ENSGALT00000029277	YBX3	ENSGALT00000015532	
ENSGALT00000023808	STYK1	ENSGALT00000015593	ADAMTS20
ENSGALT00000023745	GABARAPL3	ENSGALT00000015635	
ENSGALT00000023883	GDAP2	ENSGALT00000015636	TMEM117
ENSGALT00000023902		ENSGALT00000015735	ARID2
ENSGALT00000023908		ENSGALT00000015787	
ENSGALT00000023929	CTC1	ENSGALT00000015837	
ENSGALT00000024021		ENSGALT00000015853	
ENSGALT00000024035		ENSGALT00000024545	DTNA

ENSGALT00000024112	GTF2E1	ENSGALT00000015861	
ENSGALT00000024149	LRRC58	ENSGALT00000015878	
ENSGALT00000030475	GSK3B	ENSGALT00000015976	
ENSGALT00000030474	COX17	ENSGALT00000015981	RPL18A
ENSGALT00000036952		ENSGALT00000015988	
ENSGALT00000024296		ENSGALT00000015999	
ENSGALT00000024306	TMEM39A	ENSGALT00000016025	
ENSGALT00000024316	B4GALT3	ENSGALT00000016059	
ENSGALT00000024321	ARHGAP31	ENSGALT00000016099	CAND1
ENSGALT00000024333	IGSF11	ENSGALT00000016139	
ENSGALT00000024400		ENSGALT00000016160	
ENSGALT00000024518	SLC35A5	ENSGALT00000016192	
ENSGALT00000024565	CCDC181	ENSGALT00000016187	
ENSGALT00000024570	BLZF1	ENSGALT00000021064	MCM4
ENSGALT00000030447	DPT	ENSGALT00000016330	PTPRB
ENSGALT00000024599	TIPRL	ENSGALT00000024594	SNAI2
ENSGALT00000024600	GPR161	ENSGALT00000041400	C8orf22
ENSGALT00000024611	COL8A1	ENSGALT00000016545	ZFC3H1
ENSGALT00000024648	FILIP1L	ENSGALT00000016553	RAB21
ENSGALT00000024670	TOMM70A	ENSGALT00000024628	RB1CC1
ENSGALT00000024692	TFG	ENSGALT00000022162	
ENSGALT00000024704	ABI3BP	ENSGALT00000016625	
ENSGALT00000024748	ZBTB11	ENSGALT00000016660	OSBPL8
ENSGALT00000024756	RPL24	ENSGALT00000024663	
ENSGALT00000024772	CBLB	ENSGALT00000024757	
ENSGALT00000024777	BBX	ENSGALT00000029932	
ENSGALT00000024797	PVRL3	ENSGALT00000024867	
ENSGALT00000024806	PLCXD2	ENSGALT00000024871	PENK
ENSGALT00000024813	ABHD10	ENSGALT00000024873	IMPAD1
ENSGALT00000024837		ENSGALT00000024899	
ENSGALT00000024843	C3orf38	ENSGALT00000018193	C12orf50
ENSGALT00000024855	PROS1	ENSGALT00000024908	NSMAF
ENSGALT00000024861	ARL13B	ENSGALT00000018208	C12orf29
ENSGALT00000024895	DCAF6	ENSGALT00000018221	CEP290
ENSGALT00000024900		ENSGALT00000018257	TMTC3
ENSGALT00000024901	MPZL1	ENSGALT00000018392	
ENSGALT00000030405		ENSGALT00000018400	
ENSGALT00000024931	PTGFRN	ENSGALT00000018404	EEA1
ENSGALT00000024944	IGSF3	ENSGALT00000018413	
ENSGALT00000041393		ENSGALT00000030642	
ENSGALT00000024990		ENSGALT00000018428	
ENSGALT00000025002	GBE1	ENSGALT00000024965	ASPH

ENSGALT00000025252		ENSGALT00000024998	GGH
ENSGALT00000036824		ENSGALT00000030640	CCDC41
ENSGALT00000030387		ENSGALT00000018479	
ENSGALT00000025349	CHODL	ENSGALT00000018529	VEZT
ENSGALT00000025402	ATP5J	ENSGALT00000025000	
ENSGALT00000025465	ADAMTS1	ENSGALT00000022941	ADHFE1
ENSGALT00000025470	ADAMTS5	ENSGALT00000018639	
ENSGALT00000025479		ENSGALT00000025029	
ENSGALT00000025494	USP16	ENSGALT00000018679	CDK17
ENSGALT00000025511	MAP3K7CL	ENSGALT00000018716	
ENSGALT00000025518	BACH1	ENSGALT00000018773	
ENSGALT00000025595	HUNK	ENSGALT00000023424	
ENSGALT00000025608	URB1	ENSGALT00000025048	CSPP1
ENSGALT00000025609		ENSGALT00000025075	ARFGEF<br>1
ENSGALT00000025637	SYNJ1	ENSGALT00000018870	
ENSGALT00000025691		ENSGALT00000025131	SLCO5A1
ENSGALT00000025738		ENSGALT00000025181	
ENSGALT00000025788	ITSN1	ENSGALT00000030884	MSC
ENSGALT00000025800		ENSGALT00000019040	
ENSGALT00000030355	HLCS	ENSGALT00000019063	NUP205
ENSGALT00000025859	TTC3	ENSGALT00000025372	
ENSGALT00000025863	DSCR3	ENSGALT00000025409	ZNF704
ENSGALT00000025866		ENSGALT00000030862	PAG1
ENSGALT00000025873		ENSGALT00000025468	
ENSGALT00000030348		ENSGALT00000025499	LRRCC1
ENSGALT00000025882	BRWD1	ENSGALT00000025504	CA13
ENSGALT00000025891		ENSGALT00000019173	
ENSGALT00000025999		ENSGALT00000019187	
ENSGALT00000026046	ABCG1	ENSGALT00000019245	
ENSGALT00000026078	SLC37A1	ENSGALT00000019256	
ENSGALT00000026088	WDR4	ENSGALT00000030620	
ENSGALT00000036704		ENSGALT00000025563	
ENSGALT00000026100	PKNOX1	ENSGALT00000019301	PLBD1
ENSGALT00000026110	CBS	ENSGALT00000036640	
ENSGALT00000026117		ENSGALT00000019356	
ENSGALT00000026124		ENSGALT00000019412	
ENSGALT00000026125	AGPAT3	ENSGALT00000019419	FAM109B
ENSGALT00000036686	TRAPPC10	ENSGALT00000025621	
ENSGALT00000030337		ENSGALT00000025631	
ENSGALT00000026133		ENSGALT00000019444	CCDC13<br>4

ENSGALT00000026167		ENSGALT00000025647	DECR1
ENSGALT00000026174		ENSGALT00000019461	NHP2L1
ENSGALT00000026181	MED14	ENSGALT00000019464	DESI1
ENSGALT00000026187		ENSGALT00000019482	PMM1
ENSGALT00000036653	BCOR	ENSGALT00000019494	
ENSGALT00000026207		ENSGALT00000019497	
ENSGALT00000026211	RPGR	ENSGALT00000037963	
ENSGALT00000026220		ENSGALT00000019514	ZC3H7B
ENSGALT00000026229		ENSGALT00000025676	
ENSGALT00000026255	TAB3	ENSGALT00000025692	
ENSGALT00000026256		ENSGALT00000025711	
ENSGALT00000026334		ENSGALT00000019565	
ENSGALT00000026357		ENSGALT00000019589	XPNPEP3
ENSGALT00000026363		ENSGALT00000019595	
ENSGALT00000026366	APOO	ENSGALT00000025714	RNF151
ENSGALT00000026387	PRDX4	ENSGALT00000025727	KIAA1429
ENSGALT00000030302	SMPX	ENSGALT00000019602	SLC25A17
ENSGALT00000026441	CNKSR2	ENSGALT00000025742	DPY19L4
ENSGALT00000026456	RPS6KA3	ENSGALT00000019637	SGSM3
ENSGALT00000026507		ENSGALT00000025759	
ENSGALT00000026711		ENSGALT00000030837	NDUFAF6
ENSGALT00000026728	ASB11	ENSGALT00000025765	
ENSGALT00000026750	GEMIN8	ENSGALT00000025772	
ENSGALT00000026753		ENSGALT00000025777	
ENSGALT00000026755	OFD1	ENSGALT00000036623	MTDH
ENSGALT00000026761		ENSGALT00000023439	MATN2
ENSGALT00000026762		ENSGALT00000037892	
ENSGALT00000026770		ENSGALT00000019837	MIEF1
ENSGALT00000026803	CLCN4	ENSGALT00000019854	
ENSGALT00000026817		ENSGALT00000029404	HRSP12
ENSGALT00000026856	MXRA5	ENSGALT00000025801	
ENSGALT00000026917	DHRSX	ENSGALT00000025802	OSR2
ENSGALT00000026925		ENSGALT00000030604	
ENSGALT00000026928	ASMTL	ENSGALT00000019957	JOSD1
ENSGALT00000026955	PPP2R3B	ENSGALT00000030602	TOMM22
ENSGALT00000026992	JADE3	ENSGALT00000019954	
ENSGALT00000026993		ENSGALT00000030826	
ENSGALT00000027008	CYFIP1	ENSGALT00000030825	POLR2K
ENSGALT00000027031	ATP10A	ENSGALT00000020002	DDX17

ENSGALT00000027040	UBE3A	ENSGALT00000025842	RNF19A
ENSGALT00000036592		ENSGALT00000028271	ANKRD46
ENSGALT00000027049	INPP4A	ENSGALT00000023333	
ENSGALT00000027060	MITD1	ENSGALT00000020009	
ENSGALT00000036589	TXNDC9	ENSGALT00000020049	
ENSGALT00000027071	AFF3	ENSGALT00000020069	
ENSGALT00000027073	LONRF2	ENSGALT00000025855	RRM2B
ENSGALT00000027078		ENSGALT00000030592	PICK1
ENSGALT00000027087	CNOT11	ENSGALT00000025850	UBR5
ENSGALT00000027090	RNF149	ENSGALT00000020100	ANKRD54
ENSGALT00000027110	SLC9A2	ENSGALT00000020120	
ENSGALT00000027111	MFSD9	ENSGALT00000025898	
ENSGALT00000027114		ENSGALT00000020485	RASD2
ENSGALT00000027121		ENSGALT00000020490	
ENSGALT00000027123	UXS1	ENSGALT00000037765	
ENSGALT00000027129	GCC2	ENSGALT00000020498	
ENSGALT00000027149		ENSGALT00000020504	HMGXB4
ENSGALT00000027160		ENSGALT00000025906	LRP12
ENSGALT00000027166	ADPRHL1	ENSGALT00000025930	
ENSGALT00000027172		ENSGALT00000025937	
ENSGALT00000027189	TUBGCP3	ENSGALT00000020590	RTCB
ENSGALT00000027194	IRS2	ENSGALT00000030816	
ENSGALT00000027208		ENSGALT00000020623	BTBD11
ENSGALT00000027212	CARKD	ENSGALT00000025971	
ENSGALT00000027229		ENSGALT00000025969	UTP23
ENSGALT00000027231		ENSGALT00000025968	
ENSGALT00000027236		ENSGALT00000025965	
ENSGALT00000027237		ENSGALT00000020629	
ENSGALT00000027238		ENSGALT00000020633	RIC8B
ENSGALT00000027240	KDELC1	ENSGALT00000026501	
ENSGALT00000027243	METTL21C	ENSGALT00000026492	TAF2
ENSGALT00000027257	TMTC4	ENSGALT00000026481	DSCC1
ENSGALT00000027264	CLYBL	ENSGALT00000020663	CKAP4
ENSGALT00000027267	TM9SF2	ENSGALT00000020668	NUAK1
ENSGALT00000027285		ENSGALT00000026475	
ENSGALT00000027289		ENSGALT00000026431	
ENSGALT00000030196		ENSGALT00000020683	
ENSGALT00000027314	GPR180	ENSGALT00000026409	ATAD2
ENSGALT00000027315	TGDS	ENSGALT00000026383	FAM91A1
ENSGALT00000027349	CLN5	ENSGALT00000026348	

ENSGALT00000027350		ENSGALT00000026343	
ENSGALT00000027356		ENSGALT00000026325	KIAA0196
ENSGALT00000027358	COMMD6	ENSGALT00000026313	TRIB1
ENSGALT00000027364	KLF5	ENSGALT00000020764	HCFC2
ENSGALT00000027373		ENSGALT00000020765	
ENSGALT00000023466		ENSGALT00000026309	
ENSGALT00000027397		ENSGALT00000020769	
ENSGALT00000027403	RGCC	ENSGALT00000030784	FAM49B
ENSGALT00000027404	VWA8	ENSGALT00000020773	
ENSGALT00000027432	SLC25A30	ENSGALT00000020783	NT5DC3
ENSGALT00000027434	COG3	ENSGALT00000026194	
ENSGALT00000027439	ZC3H13	ENSGALT00000026149	
ENSGALT00000027443		ENSGALT00000020821	
ENSGALT00000027446	KIAA0226L	ENSGALT00000020822	CCDC53
ENSGALT00000027451		ENSGALT00000020826	GNPTAB
ENSGALT00000027453		ENSGALT00000020831	SYCP3
ENSGALT00000030149		ENSGALT00000020835	
ENSGALT00000027460		ENSGALT00000026120	KHDRBS 3
ENSGALT00000027462	RCBTB2	ENSGALT00000020864	PARP12
ENSGALT00000027476		ENSGALT00000020873	HIPK2
ENSGALT00000030134		ENSGALT00000026092	AGO2
ENSGALT00000027511	THSD1	ENSGALT00000020879	KIAA1549
ENSGALT00000027522		ENSGALT00000026043	PTP4A3
ENSGALT00000027523	COG6	ENSGALT00000026001	RHPN1
ENSGALT00000027525	LHFP	ENSGALT00000020934	
ENSGALT00000027526	NHLRC3	ENSGALT00000020949	
ENSGALT00000027527	PROSER1	ENSGALT00000020953	KDM7A
ENSGALT00000027540		ENSGALT00000025983	PYCRL
ENSGALT00000027550	SPG20	ENSGALT00000020957	
ENSGALT00000027564		ENSGALT00000020962	
ENSGALT00000027566	STARD13	ENSGALT00000020996	AGK
ENSGALT00000027571		ENSGALT00000021001	KIAA1147
ENSGALT00000027575	N4BP2L1	ENSGALT00000000104	NRBP2
ENSGALT00000027584	B3GALTL	ENSGALT00000021056	SYT10
ENSGALT00000027586		ENSGALT00000021073	YARS2
ENSGALT00000036455		ENSGALT00000021086	
ENSGALT00000027597		ENSGALT00000021101	KIAA1551
ENSGALT00000027598		ENSGALT00000021115	
ENSGALT00000030102	POMP	ENSGALT00000021125	IPO8
ENSGALT00000027609	PAN3	ENSGALT00000021134	TMTC1
ENSGALT00000027616	LNX2	ENSGALT00000021202	B4GALN

			T3
ENSGALT00000027647		ENSGALT00000021213	ERC1
ENSGALT00000027648	MTMR6	ENSGALT00000021246	DCP1B
ENSGALT00000027655	MIPEP	ENSGALT00000021270	CECR5
ENSGALT00000027657		ENSGALT00000021281	
ENSGALT00000027661	SGCG	ENSGALT00000021282	BCL2L13
ENSGALT00000027664	MICU2	ENSGALT00000021284	
ENSGALT00000027671		ENSGALT00000021332	
ENSGALT00000027676		ENSGALT00000005490	
ENSGALT00000027677		ENSGALT00000021340	
ENSGALT00000027679	IFT88	ENSGALT00000021369	EPS8
ENSGALT00000027680		ENSGALT00000021374	
ENSGALT00000027690		ENSGALT00000021380	
ENSGALT00000021058		ENSGALT00000021439	PLEKHA5
ENSGALT00000027705	ZC3H12C	ENSGALT00000021517	
ENSGALT00000027709		ENSGALT00000021521	BCAT1
ENSGALT00000027711	KDELC2	ENSGALT00000021594	
ENSGALT00000027716	NPAT	ENSGALT00000021607	CMAS
ENSGALT00000027725	CUL5	ENSGALT00000021626	ABCC9
ENSGALT00000027729		ENSGALT00000022726	
ENSGALT00000027735	CWF19L2	ENSGALT00000022784	ITPR2
ENSGALT00000027738	GUCY1A2	ENSGALT00000022808	TM7SF3
ENSGALT00000027745	MSANTD4	ENSGALT00000022820	
ENSGALT00000027757	DCUN1D5	ENSGALT00000022856	CYB5R3
ENSGALT00000036427		ENSGALT00000022912	
ENSGALT00000027799	MAML2	ENSGALT00000022959	
ENSGALT00000027803	CEP57	ENSGALT00000022966	PARVB
ENSGALT00000027811	ENDOD1	ENSGALT00000022984	
ENSGALT00000027813		ENSGALT00000022998	
ENSGALT00000027816		ENSGALT00000023003	KIAA0930
ENSGALT00000027818	ANKRD49	ENSGALT00000023014	
ENSGALT00000027822	PANX1	ENSGALT00000023016	ATXN10
ENSGALT00000027831		ENSGALT00000015796	
ENSGALT00000036407		ENSGALT00000018841	LRP6
ENSGALT00000027863	NOX4	ENSGALT00000022888	
ENSGALT00000027869	CTSC	ENSGALT00000021942	
ENSGALT00000027880	ME3	ENSGALT00000027945	RAD51A P1
ENSGALT00000027882	RAB30	ENSGALT00000027944	
ENSGALT00000027884	PCF11	ENSGALT00000027937	
ENSGALT00000027888	CCDC90B	ENSGALT00000021680	PRMT8
ENSGALT00000022773		ENSGALT00000023174	TSPAN9

ENSGALT00000002817		ENSGALT00000021894	TULP3
ENSGALT00000027904	NARS2	ENSGALT00000021889	
ENSGALT00000000128	INTS4	ENSGALT00000023162	ITFG2
ENSGALT00000002458	AQP11	ENSGALT00000023158	
ENSGALT00000000963		ENSGALT00000023157	NECAP1
ENSGALT00000001192	LRRC32	ENSGALT00000023154	FOXJ2
ENSGALT00000001212	C11orf30	ENSGALT00000023070	
ENSGALT00000036387		ENSGALT00000023197	MAN1A2
ENSGALT00000001279	RNF121	ENSGALT00000023284	
ENSGALT00000001911		ENSGALT00000023287	
ENSGALT00000028034		ENSGALT00000035021	
ENSGALT00000028035	STIM1	ENSGALT00000022680	FBXO40
ENSGALT00000022062		ENSGALT00000023323	
ENSGALT00000014840	DGAT2	ENSGALT00000023360	
ENSGALT00000018287		ENSGALT00000023361	
ENSGALT00000036364		ENSGALT00000023441	USP5
ENSGALT00000028004	FCHSD2	ENSGALT00000023442	
ENSGALT00000028002	P2RY2	ENSGALT00000023476	ATN1
ENSGALT00000027997		ENSGALT00000023485	
ENSGALT00000027995	FAM168A	ENSGALT00000023492	
ENSGALT00000031276		ENSGALT00000023493	
ENSGALT00000027989		ENSGALT00000023496	LPCAT3
ENSGALT00000027978		ENSGALT00000023545	
ENSGALT00000027969	RNF169	ENSGALT00000023646	CLSTN3
ENSGALT00000027966		ENSGALT00000023672	
ENSGALT00000027961	SLCO2B1	ENSGALT00000023686	CLCN1
ENSGALT00000026436	KLHL34	ENSGALT00000023710	
ENSGALT00000027059		ENSGALT00000029277	YBX3
ENSGALT00000027144	CHAMP1	ENSGALT00000023808	STYK1
ENSGALT00000027875	PRSS23	ENSGALT00000023745	GABARAPL3
ENSGALT00000001106		ENSGALT00000023834	WARS2
ENSGALT00000028001		ENSGALT00000023883	GDAP2
ENSGALT00000007813	TMEM119	ENSGALT00000023902	
ENSGALT00000010788	PPP1R3C	ENSGALT00000023908	
ENSGALT00000033218	HPS6	ENSGALT00000023929	CTC1
ENSGALT00000021627		ENSGALT00000024112	GTF2E1
ENSGALT00000024049	HSPB3	ENSGALT00000024149	LRRC58
ENSGALT00000024082	ENC1	ENSGALT00000036952	
ENSGALT00000024541		ENSGALT00000024232	VANGL1
ENSGALT00000017083	PWWP2B	ENSGALT00000024296	
ENSGALT00000001841	MSL2	ENSGALT00000024306	TMEM39

			A
ENSGALT00000001984		ENSGALT00000024316	B4GALT3
ENSGALT00000028055	SPSB4	ENSGALT00000024321	ARHGAP31
ENSGALT00000004533	ZBTB38	ENSGALT00000024346	ZBTB20
ENSGALT00000015525	B3GALNT1	ENSGALT00000024400	
ENSGALT00000017230	FLRT2	ENSGALT00000024449	BOC
ENSGALT00000019144		ENSGALT00000024503	
ENSGALT00000019230	APOLD1	ENSGALT00000024530	
ENSGALT00000022862	A4GALT	ENSGALT00000024565	CCDC181
ENSGALT00000025294	NRIP1	ENSGALT00000024570	BLZF1
ENSGALT00000026015	ZBTB21	ENSGALT00000024577	NME7
ENSGALT00000006628	FEM1A	ENSGALT00000030447	DPT
ENSGALT00000002482	UTS2R	ENSGALT00000024599	TIPRL
ENSGALT00000011909		ENSGALT00000024600	GPR161
ENSGALT00000001876		ENSGALT00000024611	COL8A1
ENSGALT00000010978	CCDC71	ENSGALT00000024648	FILIP1L
ENSGALT00000009947	HNRNPA0	ENSGALT00000024670	TOMM70A
ENSGALT00000005527		ENSGALT00000024692	TFG
ENSGALT00000030942	LRRC30	ENSGALT00000024704	ABI3BP
ENSGALT00000023833		ENSGALT00000024748	ZBTB11
ENSGALT00000026439	ZHX2	ENSGALT00000024756	RPL24
ENSGALT00000026354		ENSGALT00000024769	
ENSGALT00000007889		ENSGALT00000024772	CBLB
ENSGALT00000014752		ENSGALT00000024777	BBX
ENSGALT00000018781		ENSGALT00000024797	PVRL3
ENSGALT00000013613		ENSGALT00000024806	PLCXD2
ENSGALT00000013616	THBD	ENSGALT00000024813	ABHD10
ENSGALT00000014184	FLRT3	ENSGALT00000024837	
ENSGALT00000017169	CDC42EP3	ENSGALT00000024841	
ENSGALT00000017907	IRF2BP2	ENSGALT00000024843	C3orf38
ENSGALT00000034432	B3GNT9	ENSGALT00000024855	PROS1
ENSGALT00000007124	PLEKHF1	ENSGALT00000024861	ARL13B
ENSGALT00000008264	PDP2	ENSGALT00000024895	DCAF6
ENSGALT00000008793		ENSGALT00000024900	
ENSGALT00000006800	CHST12	ENSGALT00000024901	MPZL1
ENSGALT00000007518		ENSGALT00000030405	
ENSGALT00000012664	VASN	ENSGALT00000024931	PTGFRN
ENSGALT00000006563		ENSGALT00000024944	IGSF3
ENSGALT00000009554	AGTR2	ENSGALT00000024984	

ENSGALT00000012049	
ENSGALT00000014023	
ENSGALT00000014971	SLITRK2
ENSGALT00000024087	HS3ST1
ENSGALT00000013006	
ENSGALT00000033813	
ENSGALT00000013983	GPR1
ENSGALT00000015011	
ENSGALT00000033212	PHOSPHO2
ENSGALT00000018926	TMEM177
ENSGALT00000034796	
ENSGALT00000004272	
ENSGALT00000011035	KLHL25
ENSGALT00000011869	KBTBD13
ENSGALT00000018475	SNAPC3
ENSGALT00000029098	
ENSGALT00000029086	
ENSGALT00000029081	
ENSGALT00000029080	
ENSGALT00000029077	
ENSGALT00000024828	HOOK3
ENSGALT00000024990	
ENSGALT00000024991	VGLL3
ENSGALT00000025002	GBE1
ENSGALT00000025252	
ENSGALT00000036824	
ENSGALT00000030387	
ENSGALT00000025349	CHODL
ENSGALT00000025402	ATP5J
ENSGALT00000025465	ADAMTS1
ENSGALT00000025470	ADAMTS5
ENSGALT00000025479	
ENSGALT00000025494	USP16
ENSGALT00000025511	MAP3K7CL
ENSGALT00000025518	BACH1
ENSGALT00000025595	HUNK
ENSGALT00000025608	URB1
ENSGALT00000025609	
ENSGALT00000025637	SYNJ1
ENSGALT00000025680	PAXBP1
ENSGALT00000025738	
ENSGALT00000025788	ITSN1
ENSGALT00000025800	
ENSGALT00000025844	
ENSGALT00000030355	HLCS
ENSGALT00000025859	TTC3
ENSGALT00000025863	DSCR3
ENSGALT00000025866	
ENSGALT00000025873	
ENSGALT00000030348	
ENSGALT00000025882	BRWD1
ENSGALT00000025891	
ENSGALT00000026021	C2CD2
ENSGALT00000026046	ABCG1
ENSGALT00000026078	SLC37A1
ENSGALT00000026088	WDR4
ENSGALT00000036704	
ENSGALT00000026100	PKNOX1
ENSGALT00000026104	
ENSGALT00000026110	CBS

ENSGALT00000026124	
ENSGALT00000026125	AGPAT3
ENSGALT00000030337	
ENSGALT00000026133	
ENSGALT00000026137	CXorf36
ENSGALT00000026167	
ENSGALT00000026174	
ENSGALT00000026181	MED14
ENSGALT00000026187	
ENSGALT00000026207	
ENSGALT00000026211	RPGR
ENSGALT00000026220	
ENSGALT00000026229	
ENSGALT00000026250	
ENSGALT00000026256	
ENSGALT00000026334	
ENSGALT00000026357	
ENSGALT00000026363	
ENSGALT00000026366	APOO
ENSGALT00000026387	PRDX4
ENSGALT00000030302	SMPX
ENSGALT00000026441	CNKSR2
ENSGALT00000026456	RPS6KA3
ENSGALT00000026507	
ENSGALT00000026711	
ENSGALT00000026728	ASB11
ENSGALT00000026750	GEMIN8
ENSGALT00000026753	
ENSGALT00000026755	OFD1
ENSGALT00000026761	
ENSGALT00000026770	
ENSGALT00000026790	MSL3
ENSGALT00000026803	CLCN4
ENSGALT00000026856	MXRA5
ENSGALT00000026917	DHRSX
ENSGALT00000026925	
ENSGALT00000026928	ASMTL
ENSGALT00000026955	PPP2R3B
ENSGALT00000026992	JADE3
ENSGALT00000026993	
ENSGALT00000027008	CYFIP1
ENSGALT00000027031	ATP10A

ENSGALT00000027040	UBE3A
ENSGALT00000036592	
ENSGALT00000027049	INPP4A
ENSGALT00000027060	MITD1
ENSGALT00000036589	TXNDC9
ENSGALT00000027073	LONRF2
ENSGALT00000027078	
ENSGALT00000027087	CNOT11
ENSGALT00000027090	RNF149
ENSGALT00000027110	SLC9A2
ENSGALT00000027111	MFSD9
ENSGALT00000027114	
ENSGALT00000027121	
ENSGALT00000027129	GCC2
ENSGALT00000027149	
ENSGALT00000027160	
ENSGALT00000027163	
ENSGALT00000027169	GRTP1
ENSGALT00000027172	
ENSGALT00000027180	
ENSGALT00000027189	TUBGCP3
ENSGALT00000027194	IRS2
ENSGALT00000027197	
ENSGALT00000027208	
ENSGALT00000027212	CARKD
ENSGALT00000027229	
ENSGALT00000027231	
ENSGALT00000027236	
ENSGALT00000027237	
ENSGALT00000027238	
ENSGALT00000027240	KDELC1
ENSGALT00000027245	TPP2
ENSGALT00000027257	TMTC4
ENSGALT00000027264	CLYBL
ENSGALT00000027267	TM9SF2
ENSGALT00000027285	
ENSGALT00000027289	
ENSGALT00000027292	RAP2A
ENSGALT00000030196	
ENSGALT00000027309	
ENSGALT00000027314	GPR180

ENSGALT00000027315	TGDS
ENSGALT00000027349	CLN5
ENSGALT00000027350	
ENSGALT00000027356	
ENSGALT00000027358	COMMD6
ENSGALT00000027364	KLF5
ENSGALT00000027373	
ENSGALT00000036503	
ENSGALT00000027391	
ENSGALT00000027397	
ENSGALT00000027403	RGCC
ENSGALT00000027404	VWA8
ENSGALT00000027432	SLC25A30
ENSGALT00000027434	COG3
ENSGALT00000027439	ZC3H13
ENSGALT00000027443	
ENSGALT00000027446	KIAA0226L
ENSGALT00000027451	
ENSGALT00000027453	
ENSGALT00000027454	NUDT15
ENSGALT00000030149	
ENSGALT00000036484	
ENSGALT00000027460	
ENSGALT00000027462	RCBTB2
ENSGALT00000027476	
ENSGALT00000027478	TRIM13
ENSGALT00000030134	
ENSGALT00000027488	INTS6
ENSGALT00000027505	
ENSGALT00000027509	
ENSGALT00000027510	VPS36
ENSGALT00000027511	THSD1
ENSGALT00000027516	
ENSGALT00000027519	
ENSGALT00000027522	
ENSGALT00000027523	COG6
ENSGALT00000027525	LHFP
ENSGALT00000027526	NHLRC3
ENSGALT00000027527	PROSER1

ENSGALT00000027550	SPG20
ENSGALT00000027564	
ENSGALT00000027566	STARD13
ENSGALT00000027571	
ENSGALT00000027575	N4BP2L1
ENSGALT00000027584	B3GALTL
ENSGALT00000036455	
ENSGALT00000027595	KATNAL1
ENSGALT00000027597	
ENSGALT00000030102	POMP
ENSGALT00000027616	LNX2
ENSGALT00000027647	
ENSGALT00000027648	MTMR6
ENSGALT00000027655	MIPEP
ENSGALT00000027657	
ENSGALT00000027658	SACS
ENSGALT00000027661	SGCG
ENSGALT00000027664	MICU2
ENSGALT00000027671	
ENSGALT00000027676	
ENSGALT00000027677	
ENSGALT00000027679	IFT88
ENSGALT00000027680	
ENSGALT00000027690	
ENSGALT00000021058	
ENSGALT00000027705	ZC3H12C
ENSGALT00000027709	
ENSGALT00000027711	KDELC2
ENSGALT00000027716	NPAT
ENSGALT00000027718	
ENSGALT00000027725	CUL5
ENSGALT00000027732	ALKBH8
ENSGALT00000027735	CWF19L2
ENSGALT00000027745	MSANTD4
ENSGALT00000030073	
ENSGALT00000027757	DCUN1D5
ENSGALT00000036427	
ENSGALT00000027799	MAML2
ENSGALT00000027803	CEP57
ENSGALT00000027807	FAM76B

ENSGALT00000027810	SESN3
ENSGALT00000027811	ENDOD1
ENSGALT00000027816	
ENSGALT00000027818	ANKRD4<br>9
ENSGALT00000027831	
ENSGALT00000027858	FOLH1
ENSGALT00000027863	NOX4
ENSGALT00000027869	CTSC
ENSGALT00000027880	ME3
ENSGALT00000027882	RAB30
ENSGALT00000027884	PCF11
ENSGALT00000027888	CCDC90<br>B
ENSGALT00000022773	
ENSGALT00000002817	
ENSGALT00000027904	NARS2
ENSGALT00000000128	INTS4
ENSGALT00000002458	AQP11
ENSGALT00000001093	CAPN5
ENSGALT00000031372	ACER3
ENSGALT00000001192	LRRC32
ENSGALT00000001212	C11orf30
ENSGALT00000036387	
ENSGALT00000001279	RNF121
ENSGALT00000001911	
ENSGALT00000036371	
ENSGALT00000028034	
ENSGALT00000028035	STIM1
ENSGALT00000022062	
ENSGALT00000014840	DGAT2
ENSGALT00000018287	
ENSGALT00000028004	FCHSD2
ENSGALT00000028002	P2RY2
ENSGALT00000027997	
ENSGALT00000027995	FAM168A
ENSGALT00000031276	
ENSGALT00000027989	
ENSGALT00000027985	
ENSGALT00000027978	
ENSGALT00000027969	RNF169
ENSGALT00000027966	

ENSGALT00000027961	SLCO2B1
ENSGALT00000026436	KLHL34
ENSGALT00000027059	
ENSGALT00000027144	CHAMP1
ENSGALT00000027875	PRSS23
ENSGALT00000001106	
ENSGALT00000028001	
ENSGALT00000007813	TMEM119
ENSGALT00000034402	RNF26
ENSGALT00000010788	PPP1R3C
ENSGALT00000021627	
ENSGALT00000024082	ENC1
ENSGALT00000028361	GAS1
ENSGALT00000017083	PWWP2B
ENSGALT00000001841	MSL2
ENSGALT00000001964	
ENSGALT00000001984	
ENSGALT00000004533	ZBTB38
ENSGALT00000014247	
ENSGALT00000015525	B3GALNT1
ENSGALT00000032951	P2RY14
ENSGALT00000017230	FLRT2
ENSGALT00000019144	
ENSGALT00000019840	
ENSGALT00000020121	
ENSGALT00000022862	A4GALT
ENSGALT00000024742	TRMT10C
ENSGALT00000025294	NRIP1
ENSGALT00000006628	FEM1A
ENSGALT00000039218	
ENSGALT00000002482	UTS2R
ENSGALT00000011909	
ENSGALT00000001876	
ENSGALT00000003739	RBM15B
ENSGALT00000010978	CCDC71
ENSGALT00000009947	HNRNPA0
ENSGALT00000005527	

ENSGALT00000030942	LRRC30
ENSGALT00000023833	
ENSGALT00000024880	FAM110B
ENSGALT00000026439	ZHX2
ENSGALT00000026354	
ENSGALT00000039163	PIGW
ENSGALT00000007889	
ENSGALT00000014752	
ENSGALT00000018177	
ENSGALT00000018781	
ENSGALT00000019167	
ENSGALT00000033345	LRRC8D
ENSGALT00000013613	
ENSGALT00000013616	THBD
ENSGALT00000014184	FLRT3
ENSGALT00000017169	CDC42EP3
ENSGALT00000017907	IRF2BP2
ENSGALT00000003716	CMTR2
ENSGALT00000034432	B3GNT9
ENSGALT00000007124	PLEKHF1
ENSGALT00000008264	PDP2
ENSGALT00000008793	
ENSGALT00000006800	CHST12
ENSGALT00000007518	
ENSGALT00000040564	
ENSGALT00000012664	VASN
ENSGALT00000006563	
ENSGALT00000009554	AGTR2
ENSGALT00000012049	
ENSGALT00000014023	
ENSGALT00000014971	SLITRK2
ENSGALT00000024087	HS3ST1
ENSGALT00000033813	
ENSGALT00000013983	GPR1
ENSGALT00000015011	
ENSGALT00000018926	TMEM177
ENSGALT00000034796	
ENSGALT00000004272	
ENSGALT00000011035	KLHL25
ENSGALT00000011869	KBTBD13

ENSGALT00000018475	SNAPC3
ENSGALT00000029086	
ENSGALT00000029077	
ENSGALT00000024822	
ENSGALT00000024821	THAP1
ENSGALT00000024828	HOOK3
